# Supplementary material for: mTert induction in p21-positive cells counteracts capillary rarefaction and pulmonary emphysema
Source: EMBO Rep. 2024 Feb 29;25(3):36. doi: 10.1038/s44319-023-00041-1 (PMC10933469; doi:10.1038/s44319-023-00041-1)
Supplement: Supplementary file 1 — Appendix [file 44319_2023_41_MOESM1_ESM.pdf]

Appendix

Table of Content

Appendix Figure S1 ……………………………………………………2-4

Appendix Figure S2 ……………………………………………………. 5

Appendix Figure S3 ……………………………………………………. 6

Appendix Figure S1

A

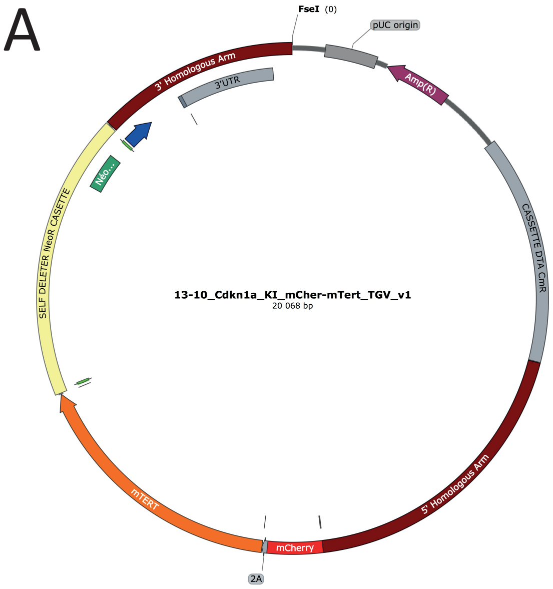

B

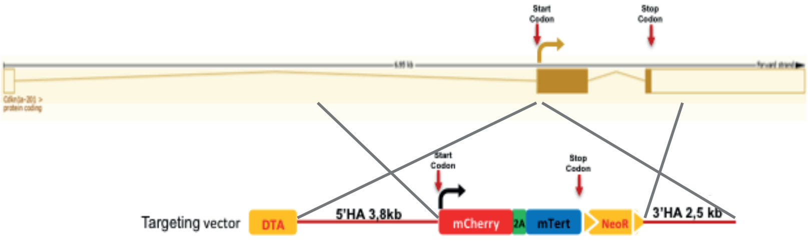

C

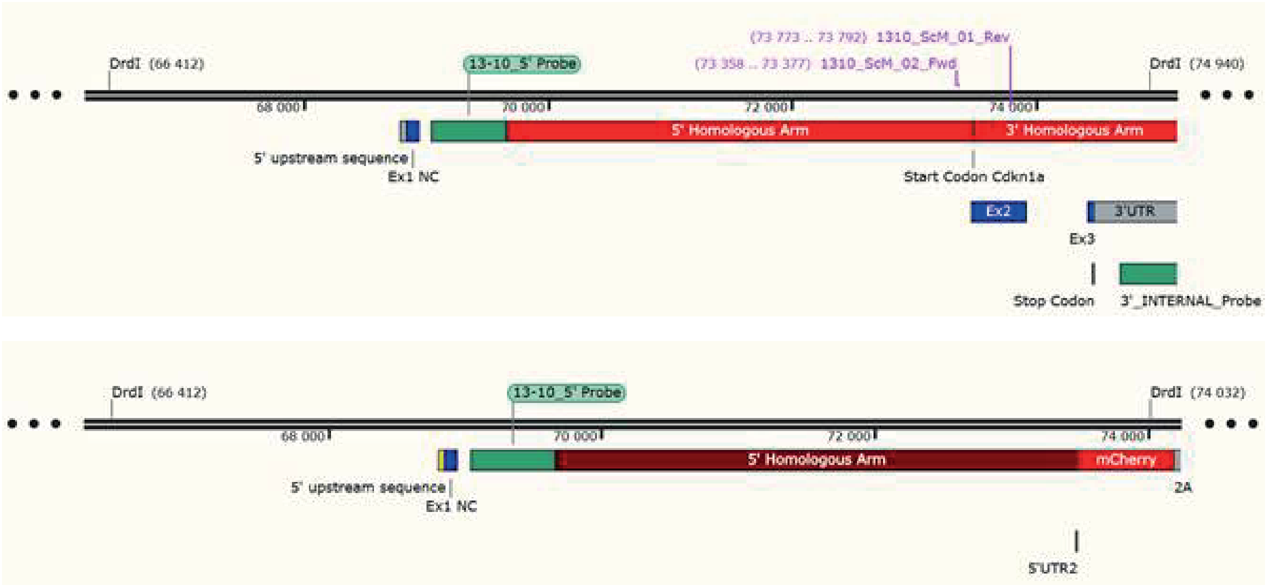

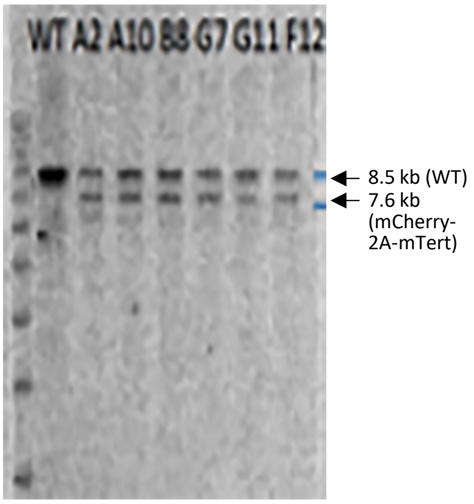

D

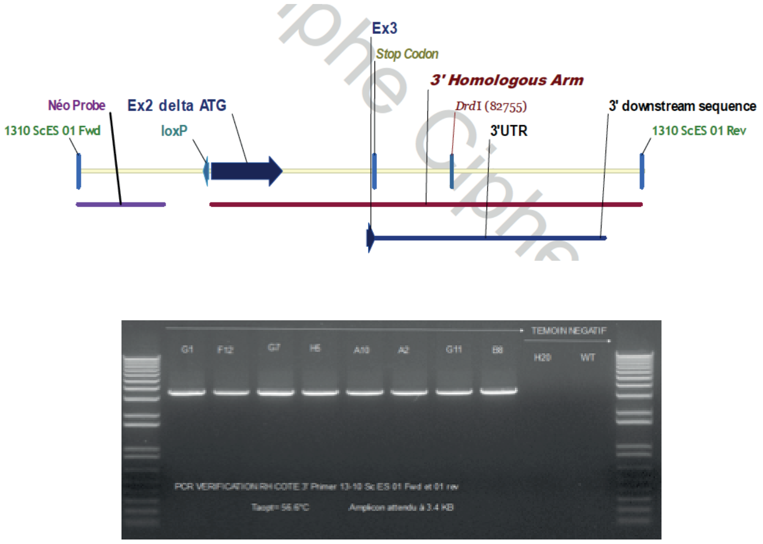

E

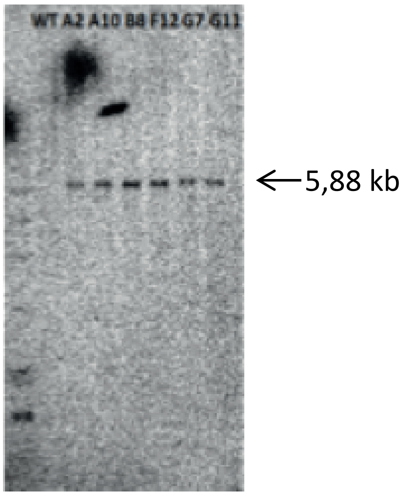

F

mCherry-2A-Tert

MVSKGEEDNMAIIKEFMRFKVHMEGSVNGHEFEIEGEGEGRPYEGTQTAKLKVTKGGPLPFAWDILSPQFMYGSK
AYVKHPADIPDYLKLSFPEGFKWERVMNFEDGGVVTVTQDSSLQDGEFIYKVKLRGTNFPSDGPVMQKKTMGWEA
SSERMYPEDGALKGEIKQRLKLKDGGHYDAEVKTTYKAKKPVQLPGAYNVNIKLDITSHNEDYTIVEQYERAEGR
HSTGGMDELYKGATNFSLLKQAGDVEENPGPLIKMTRAPRCPAVRSLLRSRYREVWPLATFVRRLGPEGRRLVQP
GDPKIYRTLVAQCLVCMHWGSQPPPADLSFHQVSSLKELVARVVQRLCERNERNVLAFGFELLNEARGGPPMAFT
SSVRSYLPNTVIETLRVSGAWMLLLSRVGDDLLVYLLAHCALYLLVPPSCAYQVCGSPLYQICATTDIWPSVSAS
YRPTRPVGRNFTNLRFLQQIKSSSRQEAPKPLALPSRGTKRHLSLTSTSVPSAKKARCYPVPRVEEGPHRQVLPT
PSGKSWVPSPARSPEVPTAEKDLSSKGKVSDLSLSGSVCCKHKPSSTSLLSPPRQNAFQLRPFIETRHFLYSRGD
GQERLNPSFLLSNLQPNLTGARRLVEIIFLGSRPRTSGPLCRTHRLSRRYWQMRPLFQQLLVNHAECQYVRLLRS
HCRFRTANQQVTDALNTSPPHLMDLLRLHSSPWQVYGFLRACLCKVVSASLWGTRHNERRFFKNLKKFISLGKYG
KLSLQELMWKMKVEDCHWLRSSPGKDRVPAAEHRLRERILATFLFWLMDTYVVQLLRSFFYITESTFQKNRLFFY
RKSVWSKLQSIGVRQHLERVRLRELSQEEVRHHQDTWLAMPICRLRFIPKPNGLRPIVNMSYSMGTRALGRRKQA
QHFTQRLKTLFSMLNYERTKHPHLMGSSVLGMNDIYRTWRAFVLRVRALDQTPRMYFVKADVTGAYDAIPQGKLV
EVVANMIRHSESTYCIRQYAVVRRDSQGQVHKSFRRQVTTLSDLQPYMGQFLKHLQDSDASALRNSVVIEQSISM
NESSSSLFDFFLHFLRHSVVKIGDRCYTQCQGIPQGSSLSTLLCSLCFGDMENKLFAEVQRDGLLLRFVDDFLLV
TPHLDQAKTFLSTLVHGVPEYGCMINLQKTVVNFPVEPGTLGGAAPYQLPAHCLFPWCGLLLDTQTLEVFCDYSG
YAQTSIKTSLTFQSVFKAGKTMRNKLLSVLRLKCHGLFLDLQVNSLQTVCINIYKIFLLQAYRFHACVIQLPFDQ
RVRKNLTFFLGIISSQASCCYAILKVKNPGMTLKASGSFPPEAAHWLCYQAFLLKLAAHSVIYKCLLGPLRTAQK
LLCRKLPEATMTILKAAADPALSTDFQTILD

Construction of the p21$^{+/Tert}$ knockin mouse

(A) Upper panel, map of the targeting vector used the introduction of the *mCherry-2A-Tert* cassette (see Methods). The targeting vector comprises the 5' homologous arm, the mCherry-2A-Tert cassette, the self-deleter neoR cassette, the 3' homologous arm and the DT-A expression cassette.

Appendix Figure S1 (continued)

(B) Scheme of the introduction of the mCherry-2A-Tert-NeoR cassette in place of the ATG codon of Cdkn1a. Yellow triangles surrounding the NeoR are the loxP sites. The diphtheria-toxin A expression cassette (DTA) is used a negative selection marker (see Methods).

(C) Checking the 5'integration of the mCherry-2A-Tert-NeoR cassette in Cdkn1a. Upper panel, schematic representation of Cdkn1a allele before (top) and after the integration of the mCherry-2A-Tert-NeoR cassette (bottom). The two first Cdkn1a exons (Ex1, Ex2) are shown in blue. Ex2 is the first coding Exon. The 5' homologous arm is shown in red (top) or in brown (bottom). The probe used to check the 5' integration cassette is shown in green. The DrdI restriction sites used to check the integration are indicated. Lower panel, selected ES cell genomic DNAs were digested with DrdI and analysed by Southern blot with the Cdkn1a probe indicated in the upper panel. The WT Cdkn1a allele is expected to give a single band of 8528 bp while the insertion of the cassette produces an additional band of 7620 bp. The names of the clones are indicated.

(D) Check of the 3'integration of the mCherry-2A-Tert-NeoR cassette in Cdkn1a. Left panel, schematic representation of Cdkn1a allele after integration of the mCherry-2A-Tert-NeoR cassette. The blue arrow represents the Cdkn1a Exon 2 without its ATG. The 3' homologous arm is represented in brown. The positions of the primers used to check the correct insertion of the cassette are indicated in green.
1310_ScES_01 Fwd: 5' CTGAATGAACTGCAGGACGA;
1310_ScES_01_Rev: 5' cttgcctatattgctcaagg.
Right panel, Long Range PCR was performed with the indicated Fwd and Rev primers. The reverse primer was outside the 3' homologous arm. The WT allele produces no amplification while the mutant allele gives a band of 3240 bp. The clones are the same as in (C).

(E) Check of the single integration of the mCherry-2A-Tert-NeoR cassette. Genomic DNA of the indicated clones were digested with EcoRV and analysed by Southern blot with the mCherry probe. DNA from the WT does not hybridize with the probe while the cassette produces a band of 5,88 kb recognized by the mCherry probe.

(F) Proteins produced by the polycistronic mCherry-2A-Tert mRNA. Ribosomal “skipping“ (Liu et al., Sci Rep 2017) produces indicated proteins.
eum iriure dolor

Appendix Figure S2

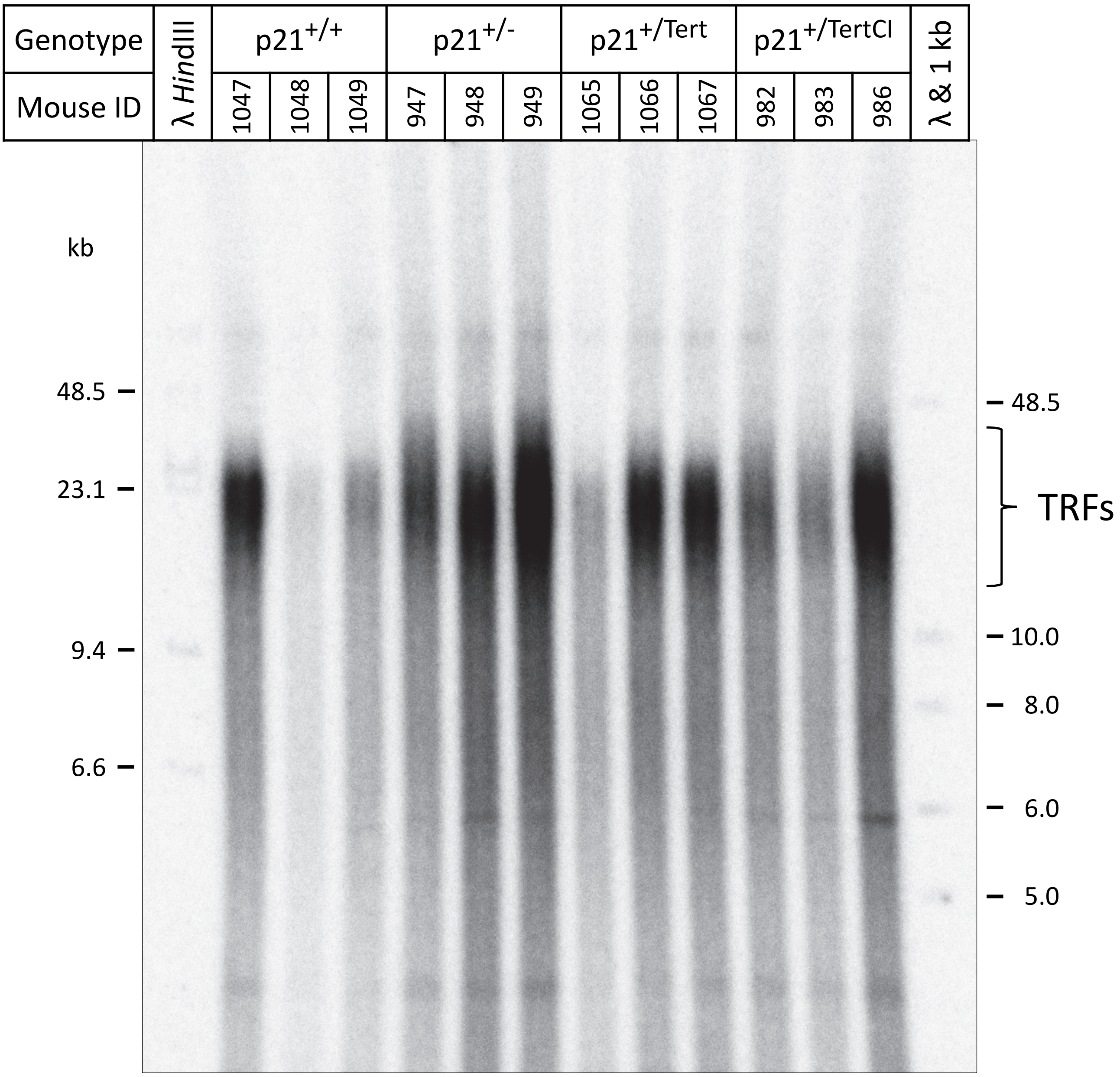

Terminal Restriction Fragments (TRFs) analysis by Southern blot

Genomic DNA (5 µg) was digested with *Hin*fI and *Rsa*I, separated in 1% agarose-0.5xTBE at 6V/cm for 20h with switch times ramped from 1 to 24 sec using CHEF-DR III System (Bio-Rad). The DNA then was transferred to Hybond N+ in denaturing conditions, and hybridized with the ^{32}P-$(CCCTAA)_{3\text{-}4}$ probe.

Appendix Figure S3

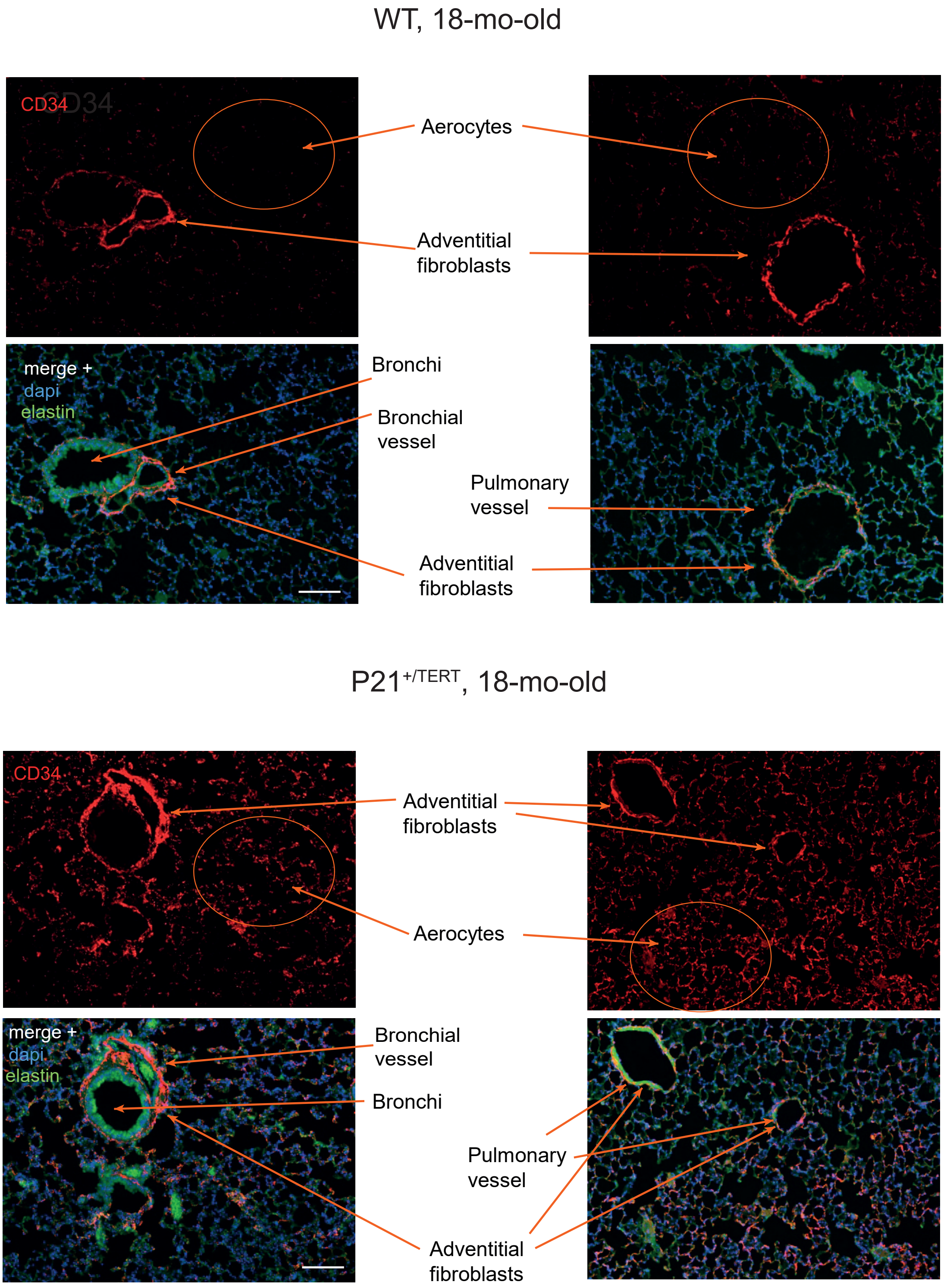

Differential localization in lung of adventitial fibroblasts and endothelial putative stem cells. Representative micrographs showing immunofluorescence of CD34 (red). Adventitial fibroblastes (CD34$^+$) are clearly identified in perivascular space of bronchial and pulmonary vessels in both young and old mice. Capillary endothelial stem cells (CD34$^+$) and aerocytes (CD34$^+$) can be observed in the parenchyma of p21$^{+/Tert\ mice}$, however in others analyzed old mice the number of CD34$^+$ cells in lung parenchyma was dramatically reduced. Blue DAPI nuclear staining, Green – elastin auto fluorescence. Bar - 50 µ.
