# Supplementary figures and images for: mTert induction in p21-positive cells counteracts capillary rarefaction and pulmonary emphysema (part 1 of 3)
Source: EMBO Rep. 2024 Feb 29;25(3):36. doi: 10.1038/s44319-023-00041-1 (PMC10933469; doi:10.1038/s44319-023-00041-1)

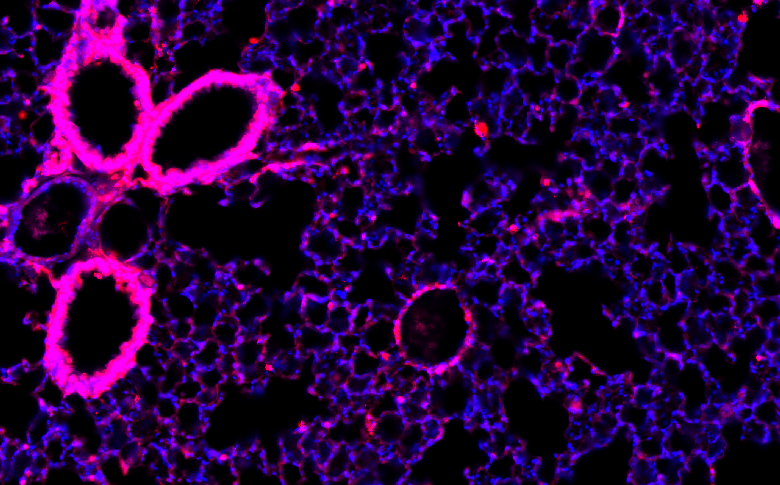

Supplement: Supplementary file 4 — Source Data Fig. 2 [file 44319_2023_41_MOESM4_ESM.zip › Source data Figure 2 /2C_image data, Micr.image/p21TERT CI old8 OxodG.tif]

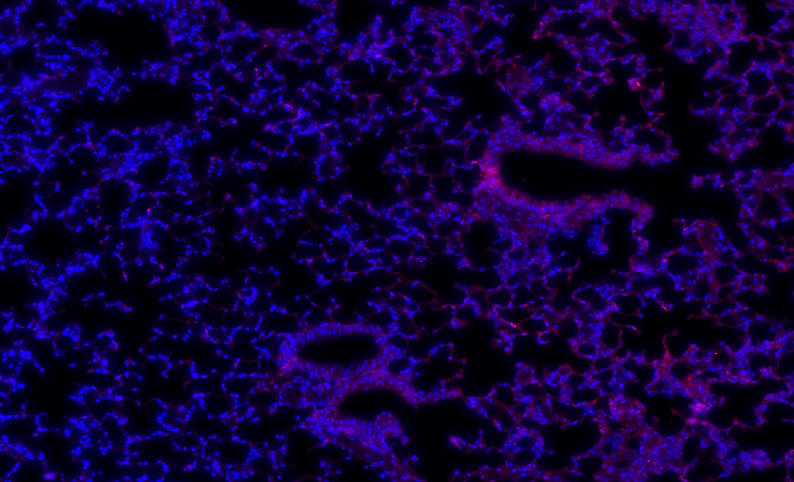

Supplement: Supplementary file 4 — Source Data Fig. 2 [file 44319_2023_41_MOESM4_ESM.zip › Source data Figure 2 /2C_image data, Micr.image/p21TERT CI young8OxodG.tif]

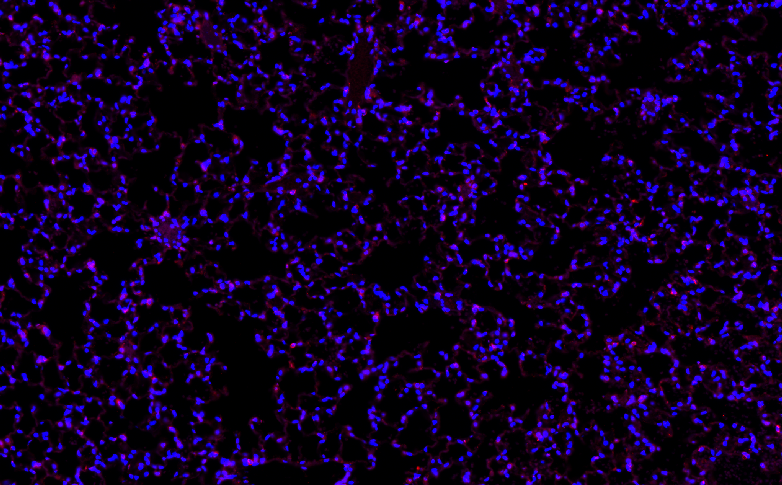

Supplement: Supplementary file 4 — Source Data Fig. 2 [file 44319_2023_41_MOESM4_ESM.zip › Source data Figure 2 /2C_image data, Micr.image/p21TERT young8OxodG.tif]

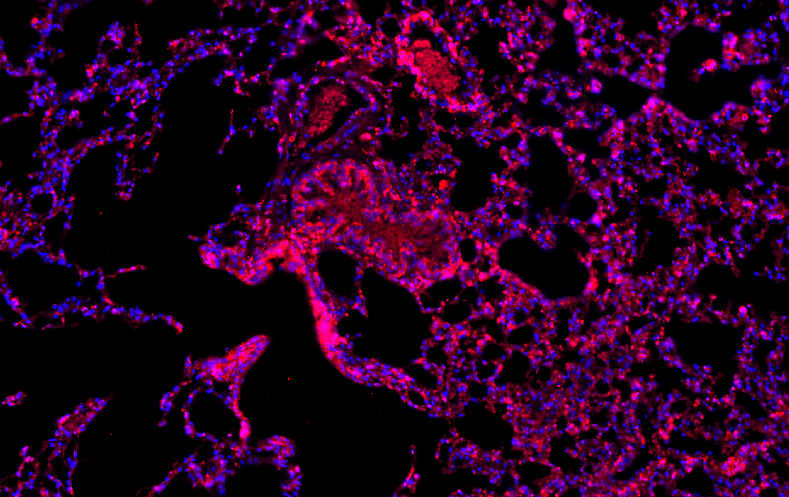

Supplement: Supplementary file 4 — Source Data Fig. 2 [file 44319_2023_41_MOESM4_ESM.zip › Source data Figure 2 /2C_image data, Micr.image/p21+- old.8OxodG.tif]

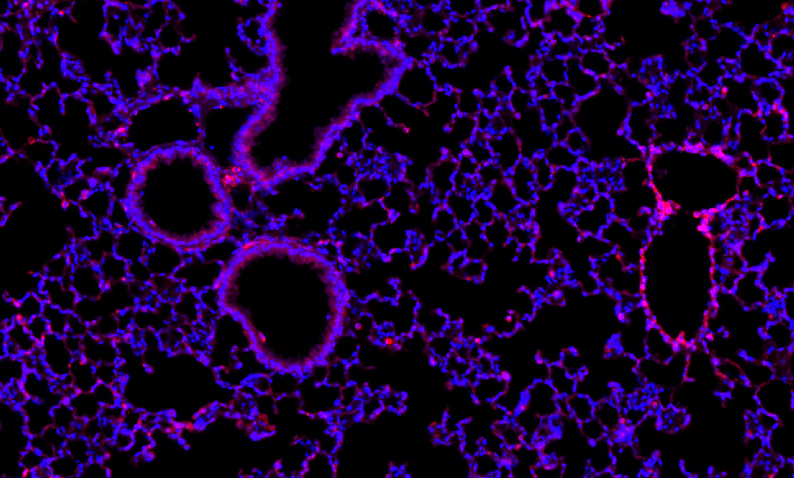

Supplement: Supplementary file 4 — Source Data Fig. 2 [file 44319_2023_41_MOESM4_ESM.zip › Source data Figure 2 /2C_image data, Micr.image/p21TERT old.8oxodG.tif]

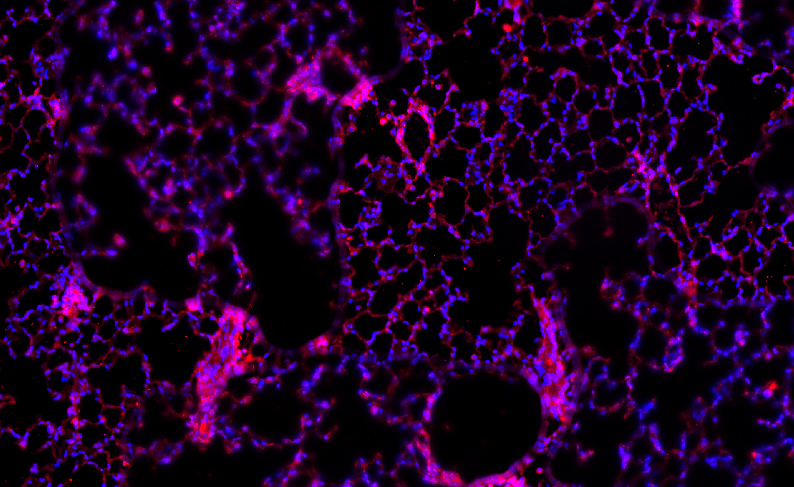

Supplement: Supplementary file 4 — Source Data Fig. 2 [file 44319_2023_41_MOESM4_ESM.zip › Source data Figure 2 /2C_image data, Micr.image/WT old.8OxodG.tif]

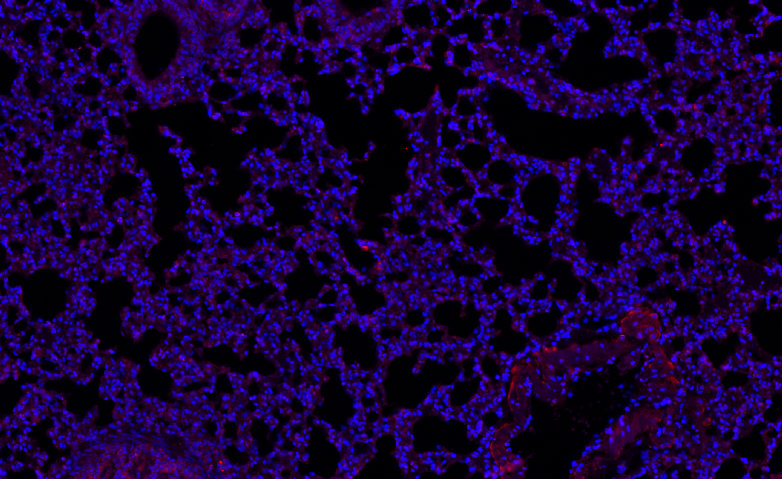

Supplement: Supplementary file 4 — Source Data Fig. 2 [file 44319_2023_41_MOESM4_ESM.zip › Source data Figure 2 /2C_image data, Micr.image/p21+-young.8OxodG.tif]

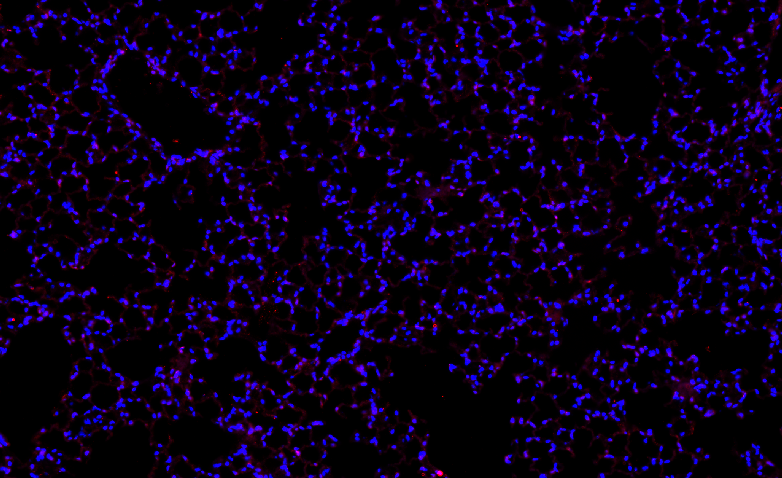

Supplement: Supplementary file 4 — Source Data Fig. 2 [file 44319_2023_41_MOESM4_ESM.zip › Source data Figure 2 /2C_image data, Micr.image/WT young.8oxodG.tif]

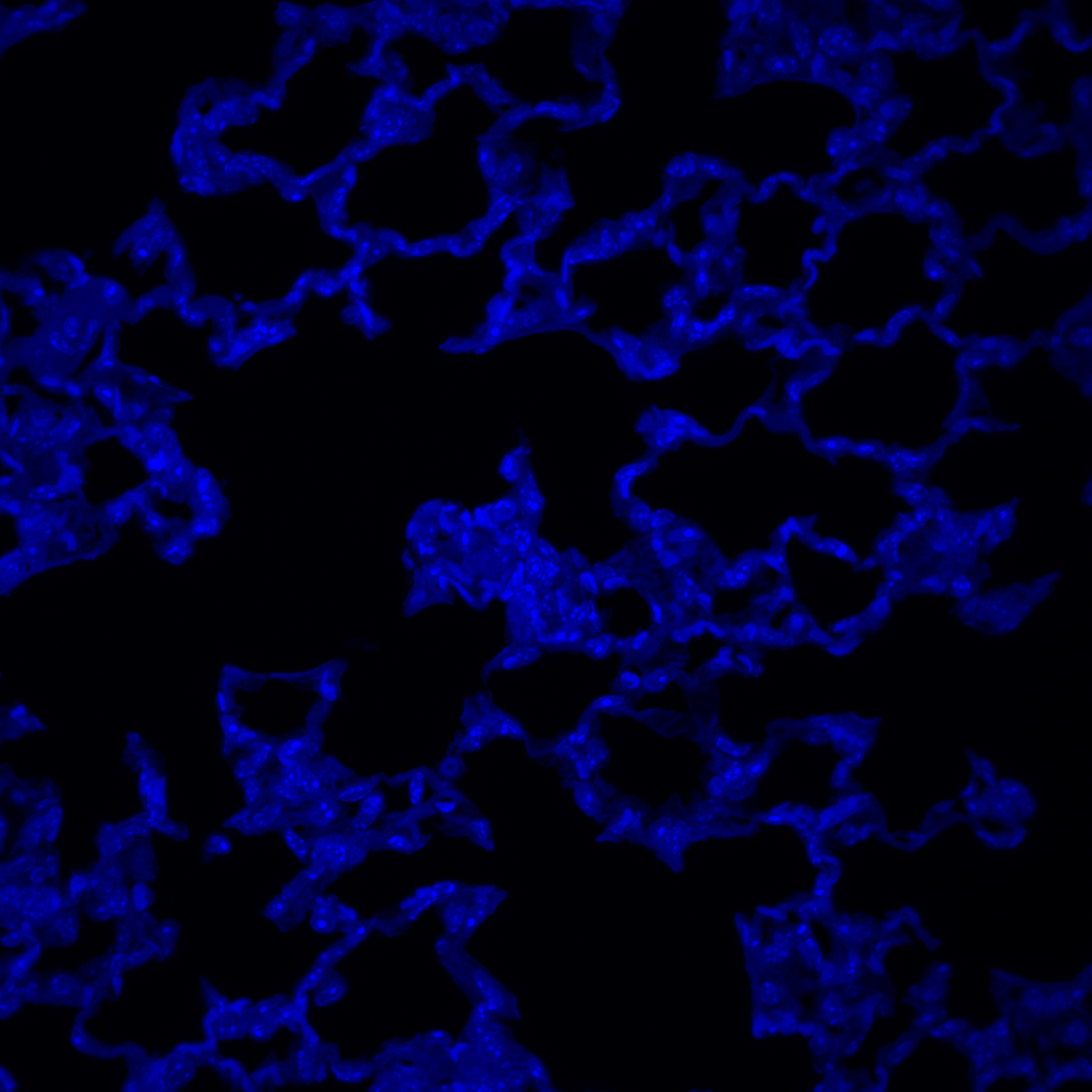

Supplement: Supplementary file 4 — Source Data Fig. 2 [file 44319_2023_41_MOESM4_ESM.zip › Source data Figure 2 /2B_Image data, Micr.image/DAPI_p21+TERTCI.lame981.nd2.tiff]

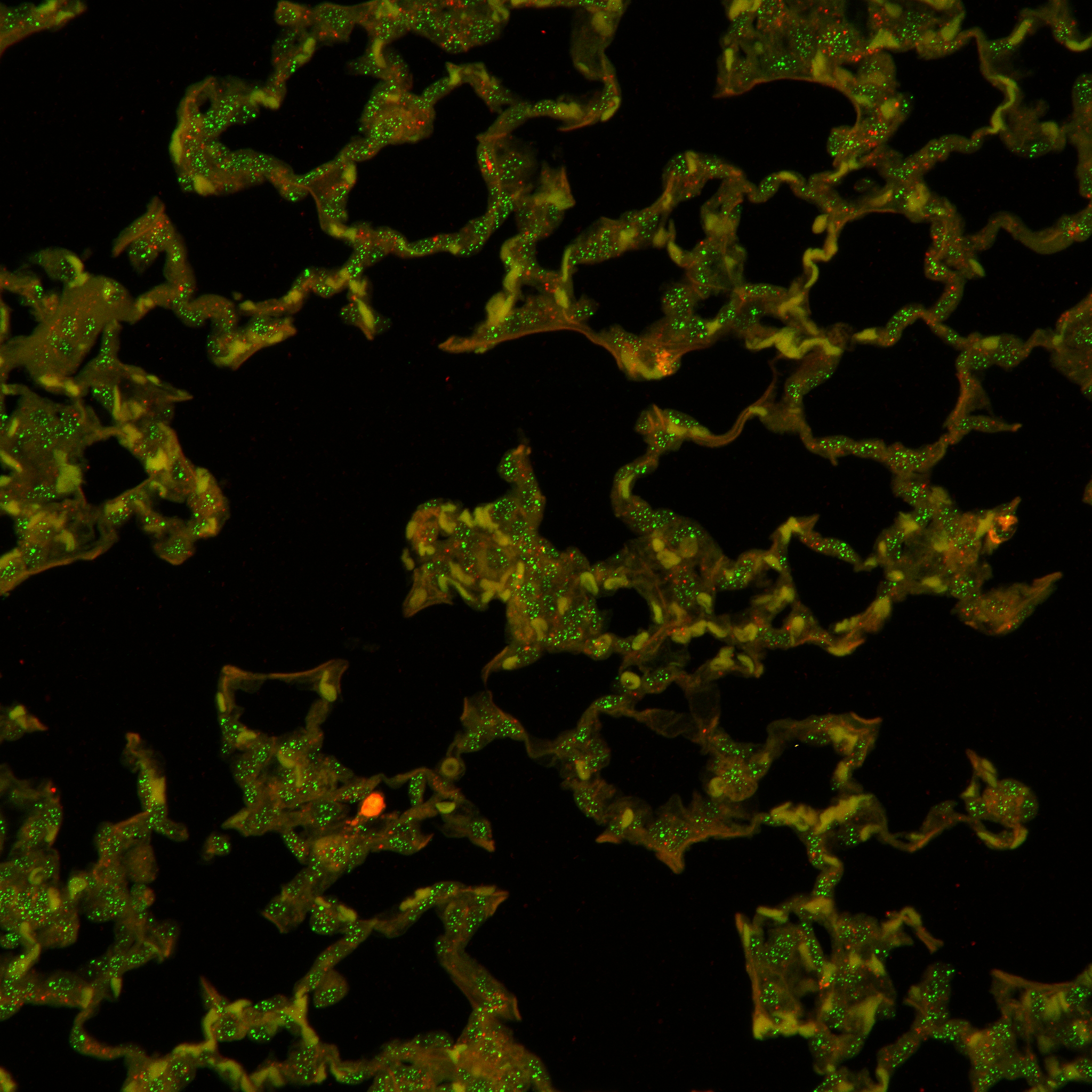

Supplement: Supplementary file 4 — Source Data Fig. 2 [file 44319_2023_41_MOESM4_ESM.zip › Source data Figure 2 /2B_Image data, Micr.image/TIFs_p21+TERTCI.lame981.nd2.tiff]

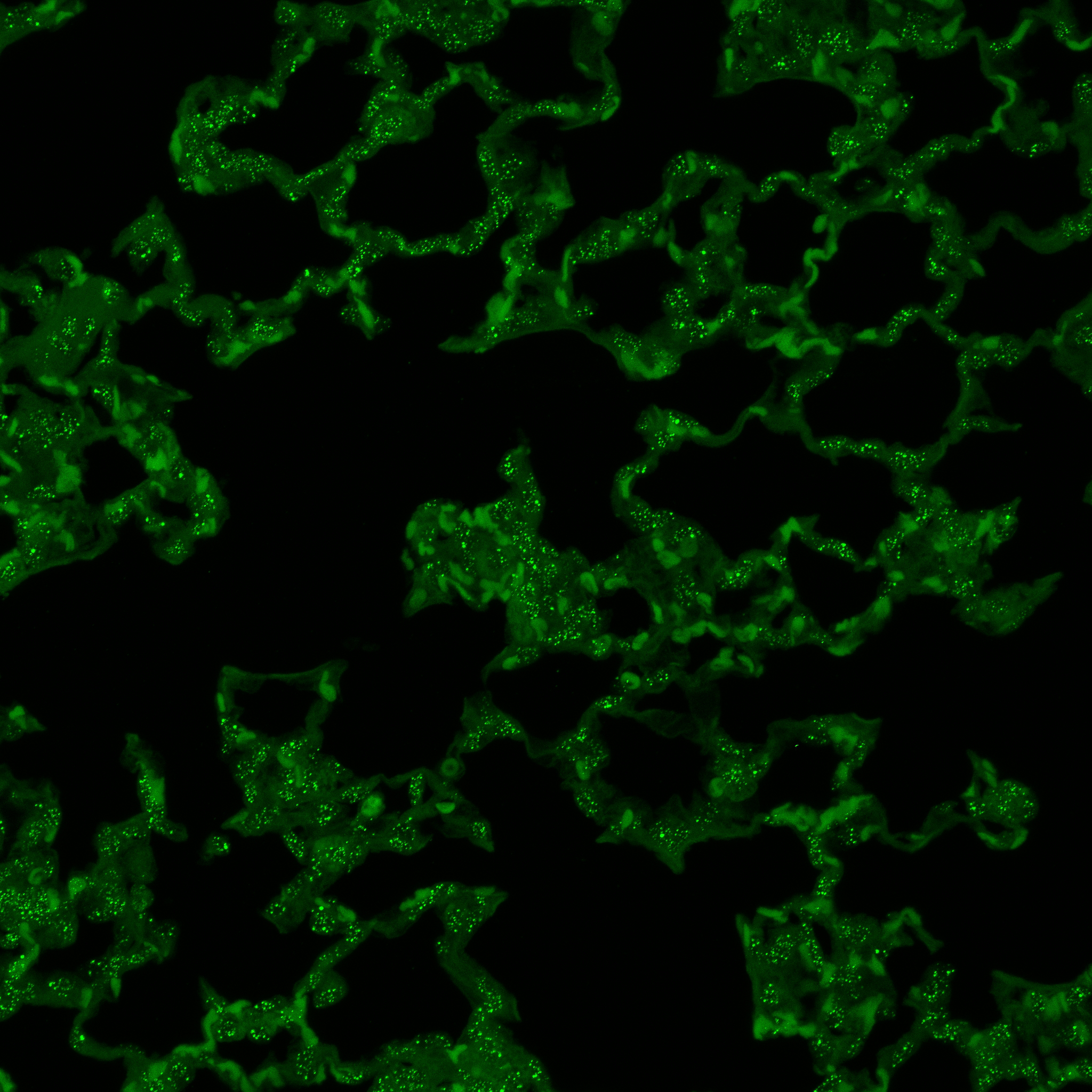

Supplement: Supplementary file 4 — Source Data Fig. 2 [file 44319_2023_41_MOESM4_ESM.zip › Source data Figure 2 /2B_Image data, Micr.image/Tel_p21+TERTCI.lame981.nd2.tiff]

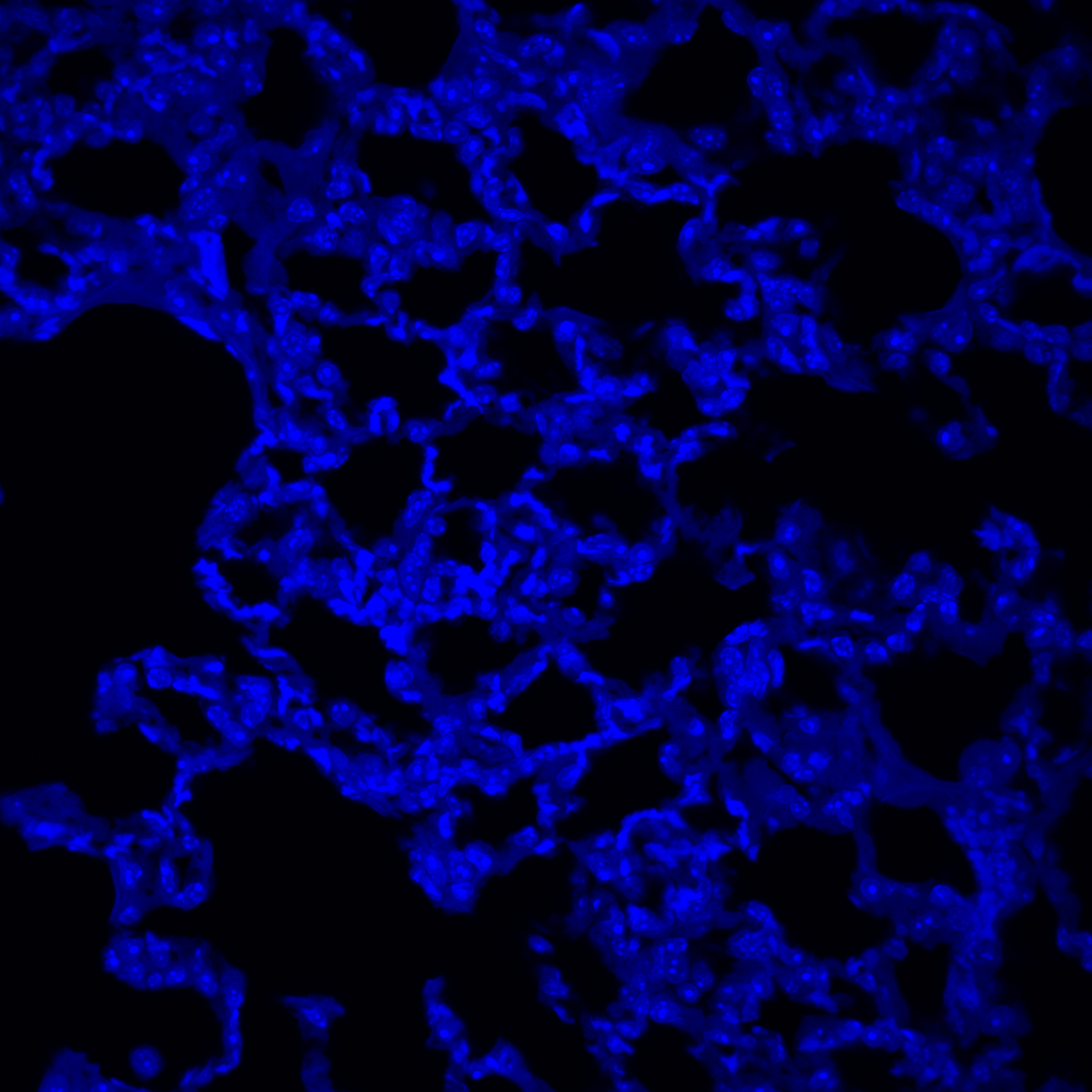

Supplement: Supplementary file 4 — Source Data Fig. 2 [file 44319_2023_41_MOESM4_ESM.zip › Source data Figure 2 /2B_Image data, Micr.image/DAPI_p21+TERT.lame961-2.nd2.tiff]

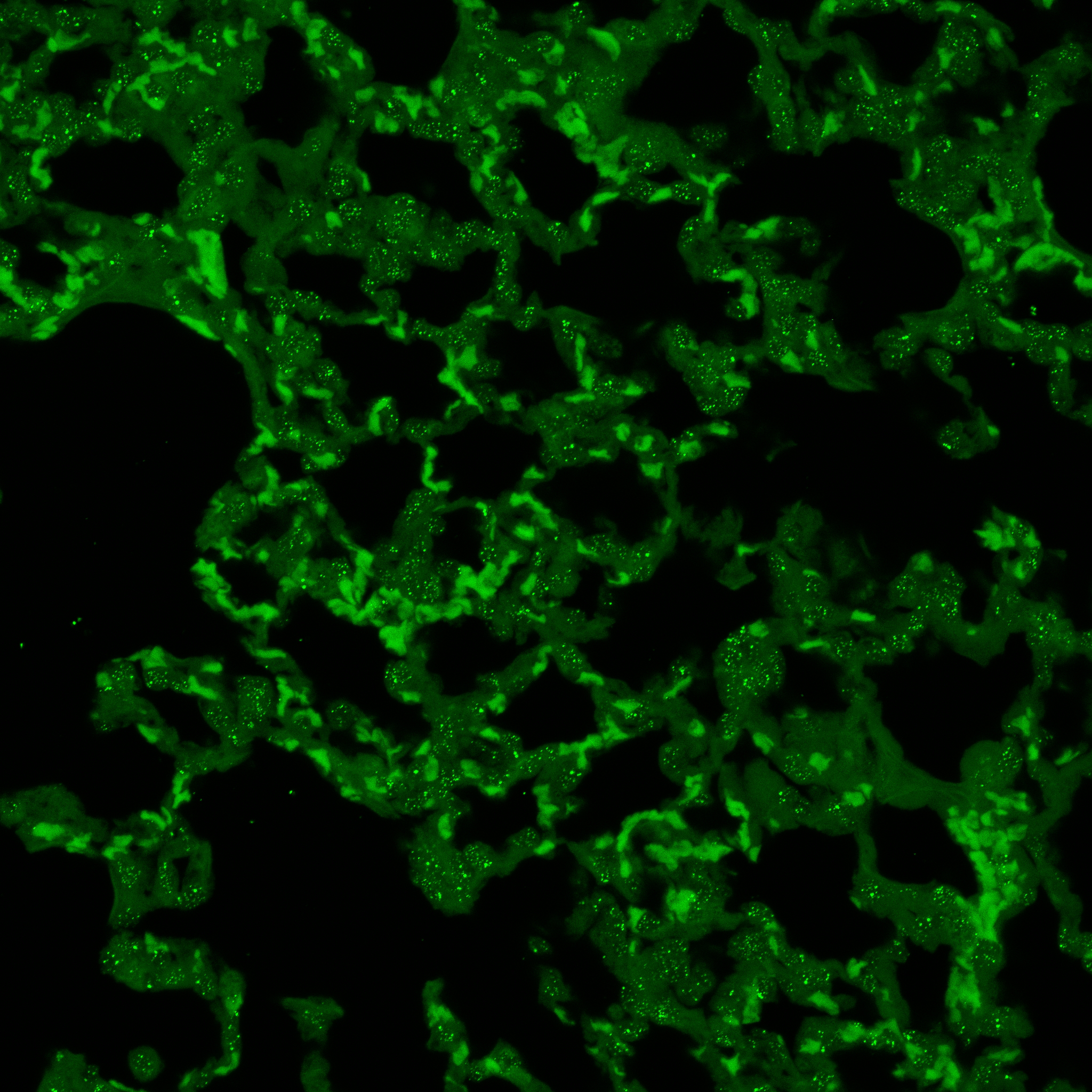

Supplement: Supplementary file 4 — Source Data Fig. 2 [file 44319_2023_41_MOESM4_ESM.zip › Source data Figure 2 /2B_Image data, Micr.image/Tel_p21+TERT.lame961-2.nd2.tiff]

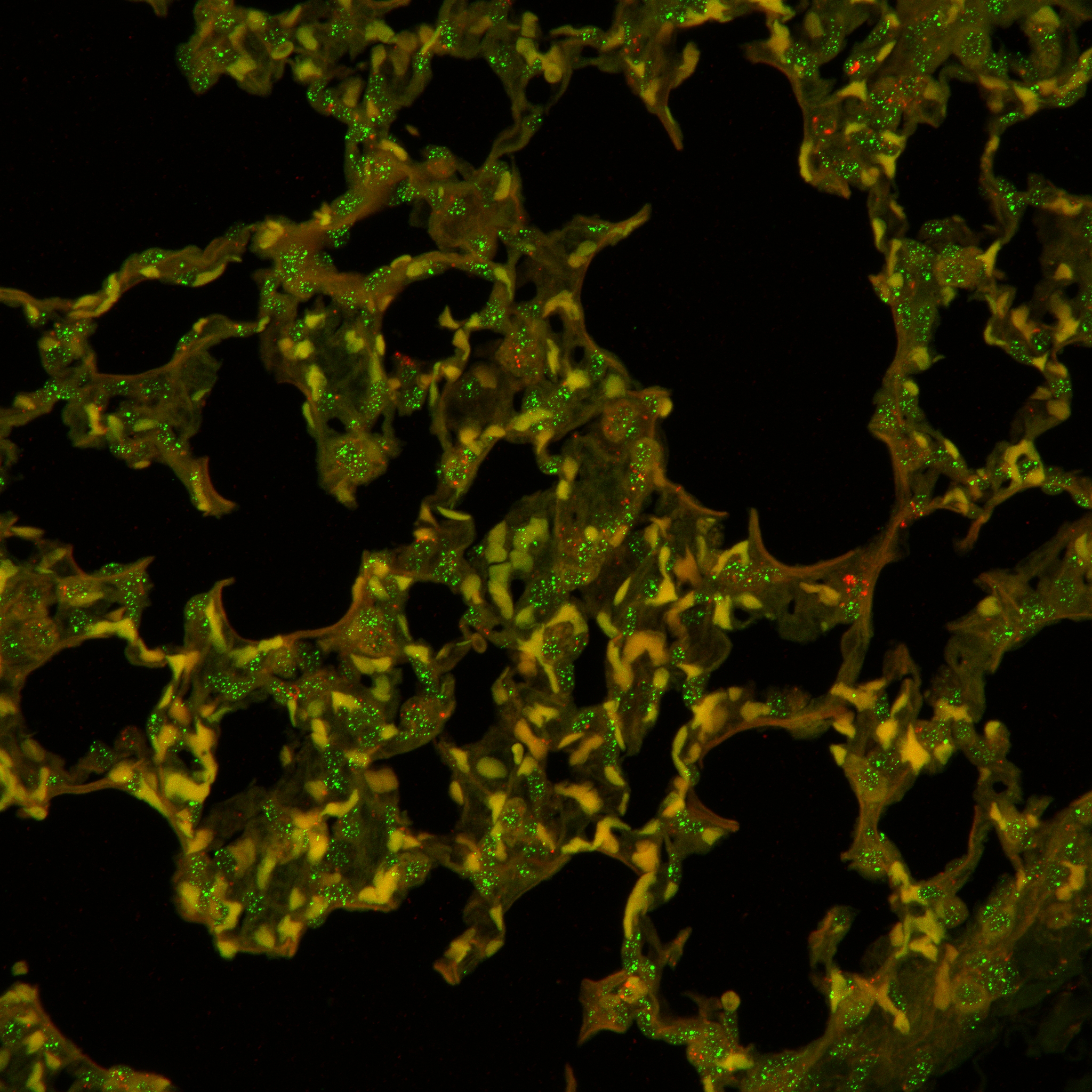

Supplement: Supplementary file 4 — Source Data Fig. 2 [file 44319_2023_41_MOESM4_ESM.zip › Source data Figure 2 /2B_Image data, Micr.image/TIFs_p21+- lame965.nd2.tiff]

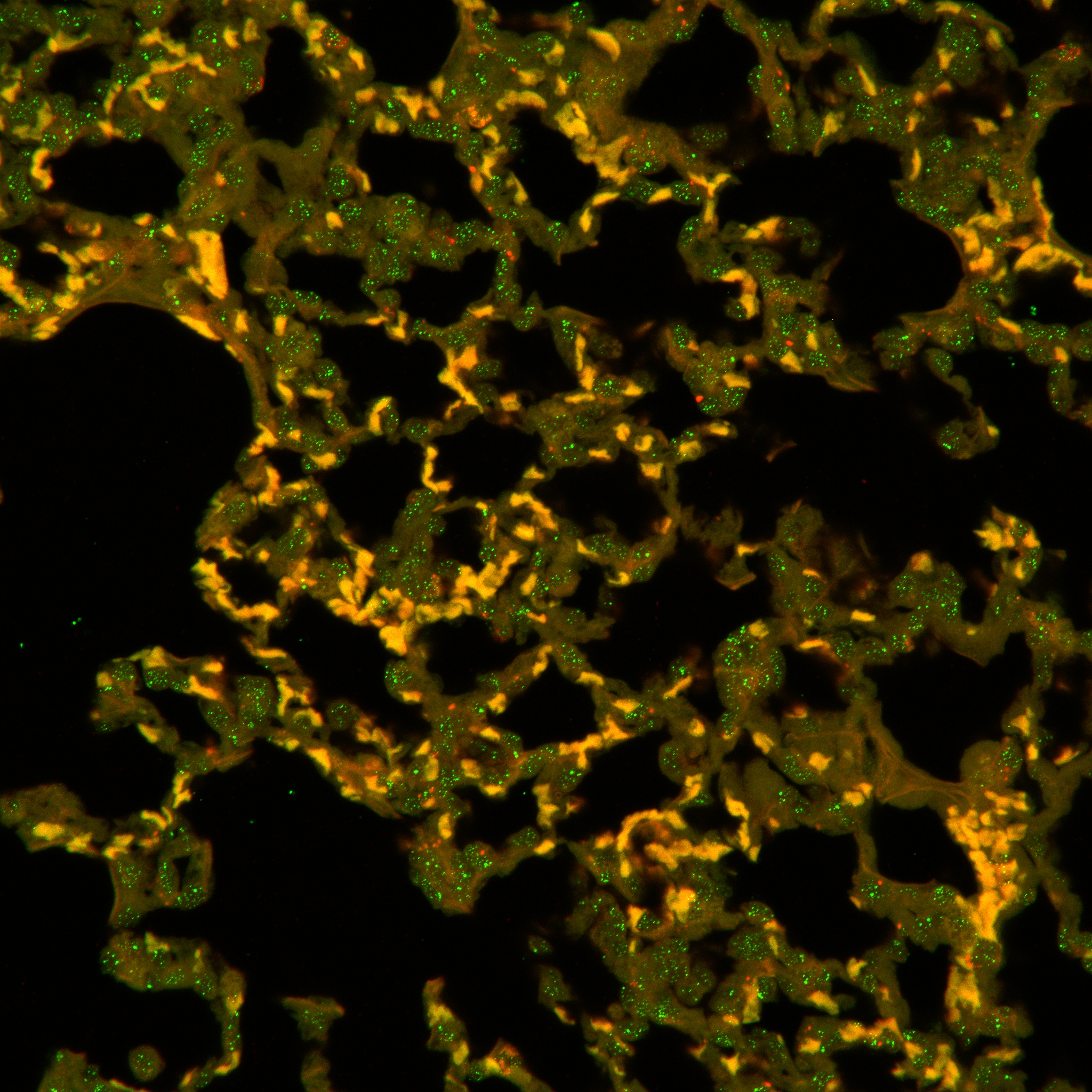

Supplement: Supplementary file 4 — Source Data Fig. 2 [file 44319_2023_41_MOESM4_ESM.zip › Source data Figure 2 /2B_Image data, Micr.image/TIFs_p21+TERT.lame961-2.nd2.tiff]

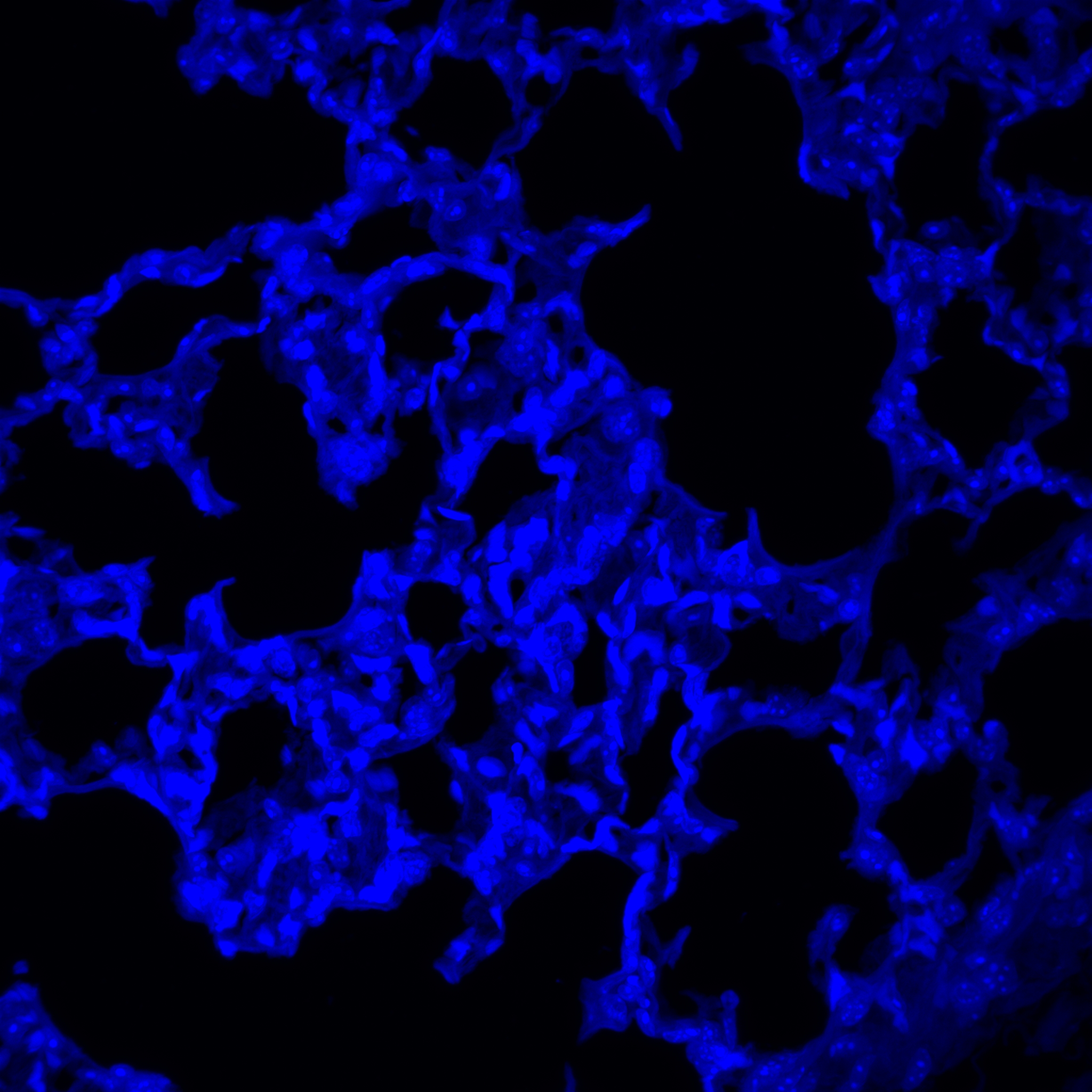

Supplement: Supplementary file 4 — Source Data Fig. 2 [file 44319_2023_41_MOESM4_ESM.zip › Source data Figure 2 /2B_Image data, Micr.image/DAPI_p21+-lame965.nd2.tiff]

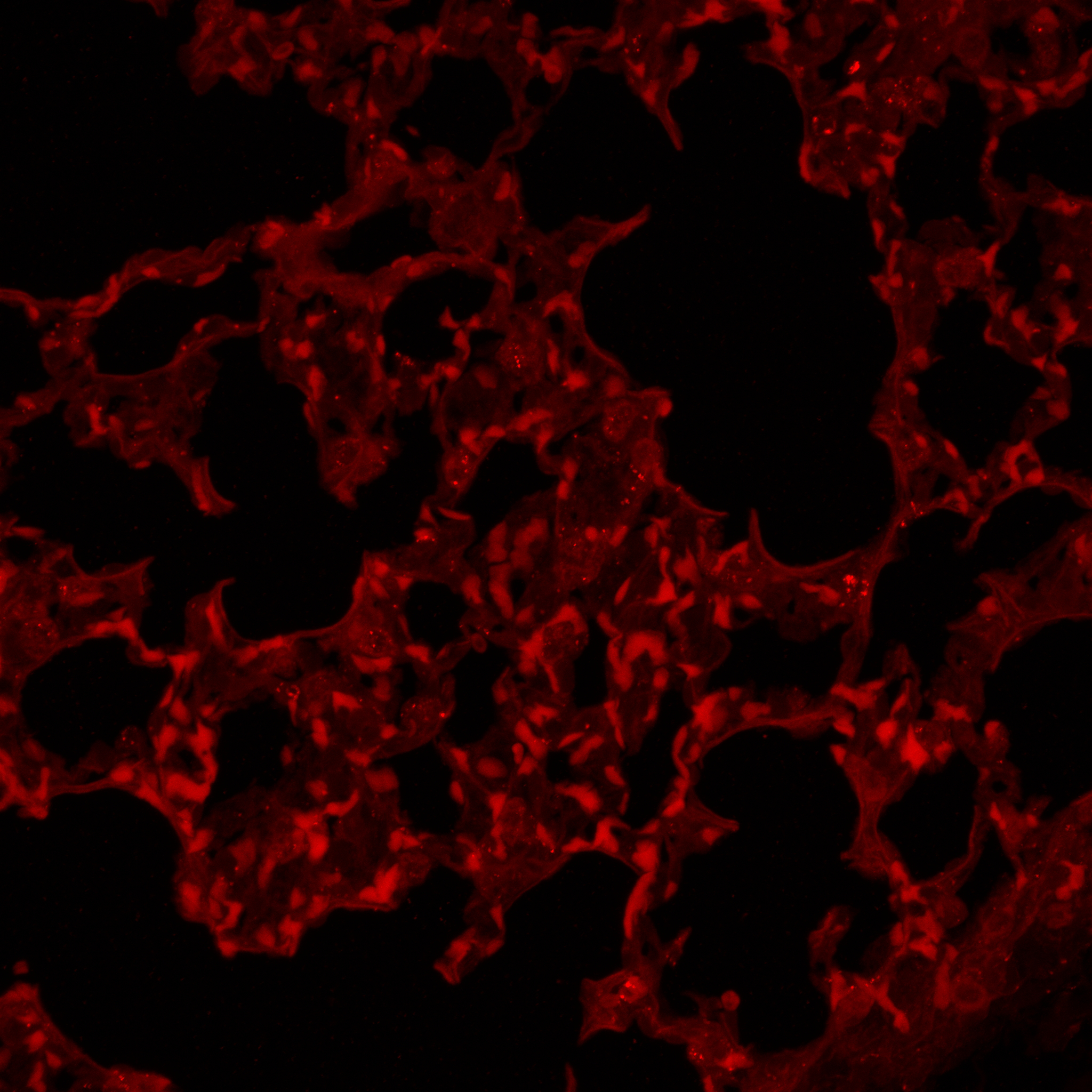

Supplement: Supplementary file 4 — Source Data Fig. 2 [file 44319_2023_41_MOESM4_ESM.zip › Source data Figure 2 /2B_Image data, Micr.image/53BP1_p21+-lame965.nd2.tiff]

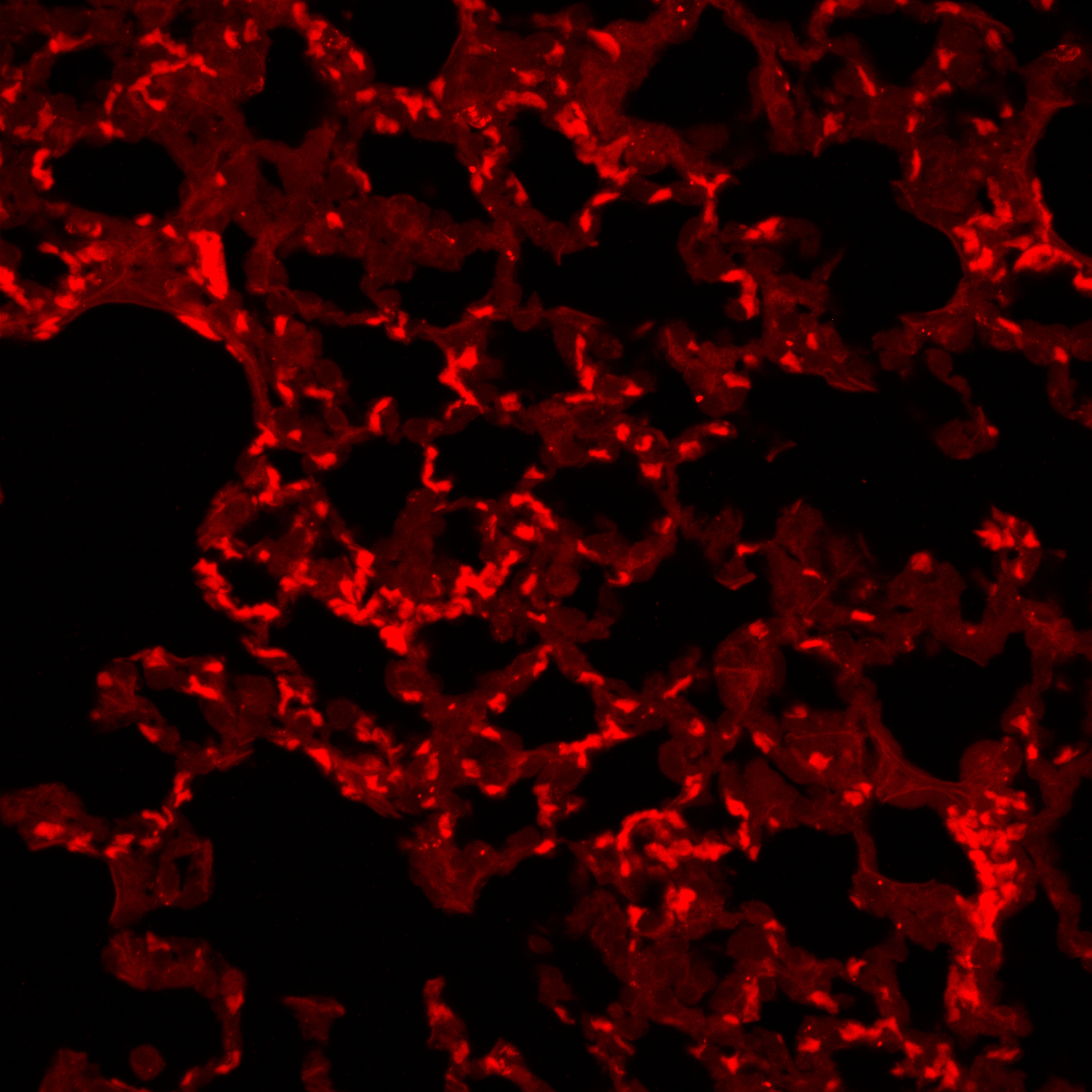

Supplement: Supplementary file 4 — Source Data Fig. 2 [file 44319_2023_41_MOESM4_ESM.zip › Source data Figure 2 /2B_Image data, Micr.image/53BP1_p21+TERT.lame961-2.nd2.tiff]

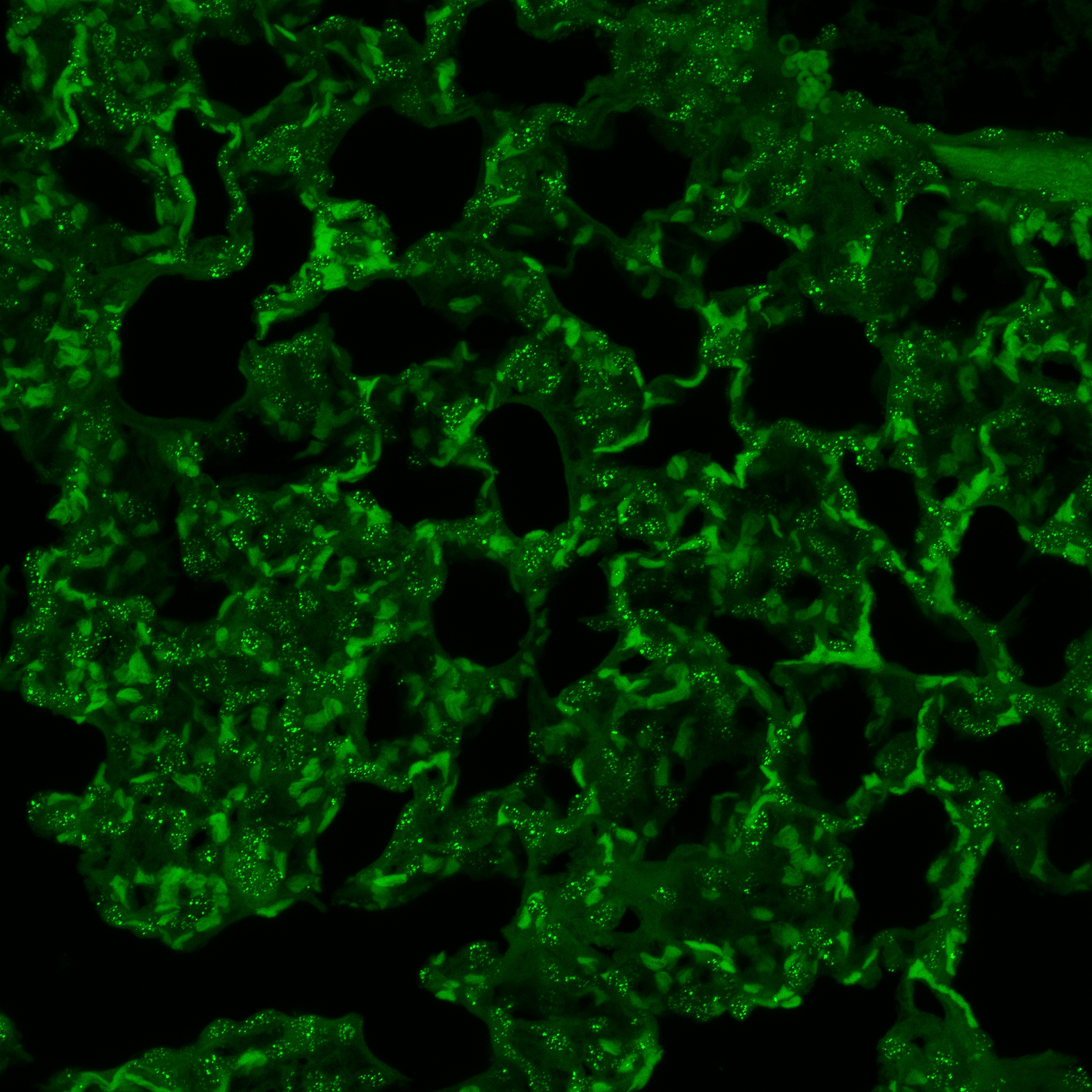

Supplement: Supplementary file 4 — Source Data Fig. 2 [file 44319_2023_41_MOESM4_ESM.zip › Source data Figure 2 /2B_Image data, Micr.image/Tel_p21++lame945.nd2.tiff]

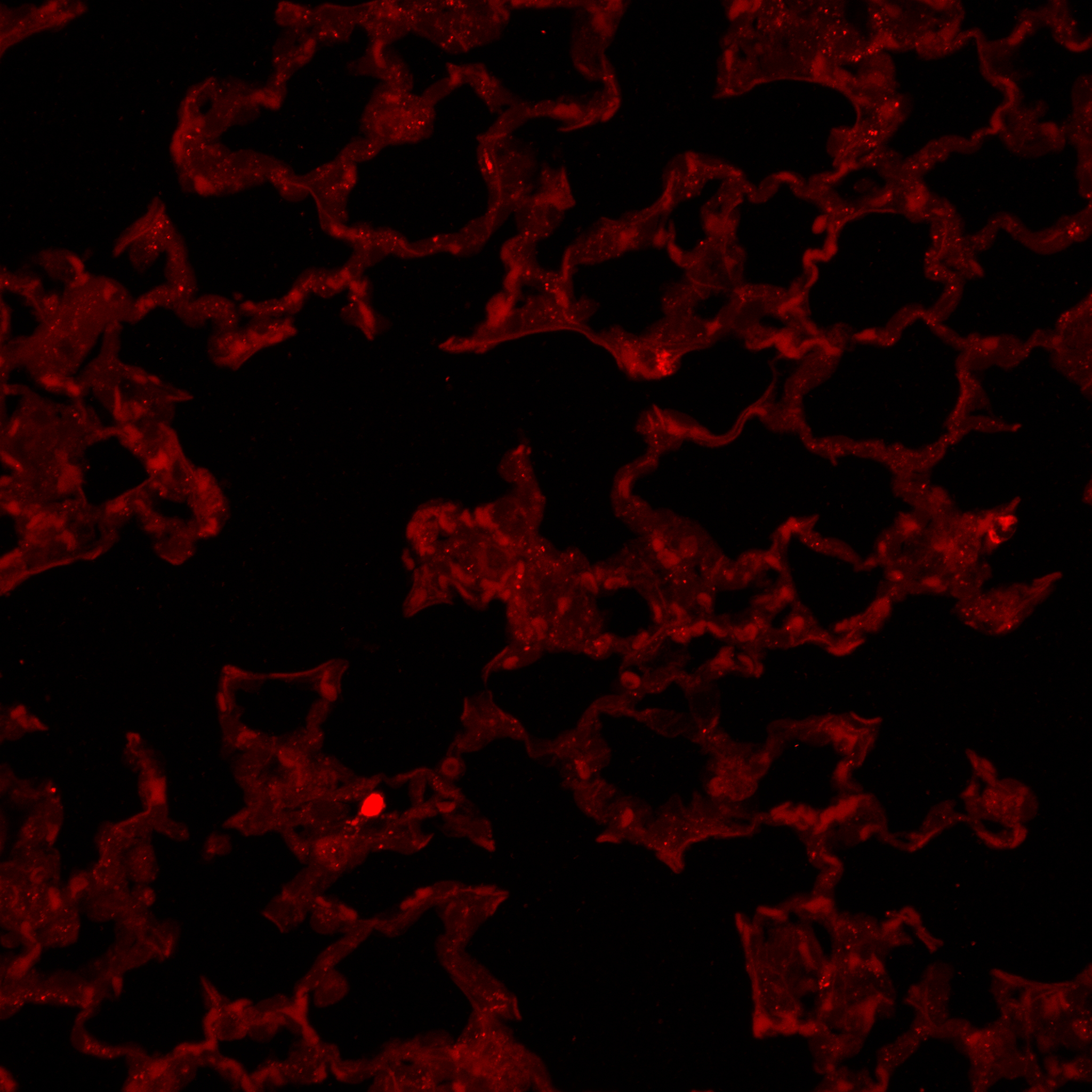

Supplement: Supplementary file 4 — Source Data Fig. 2 [file 44319_2023_41_MOESM4_ESM.zip › Source data Figure 2 /2B_Image data, Micr.image/53BP1_p21+TERTCI.lame981.nd2.tiff]

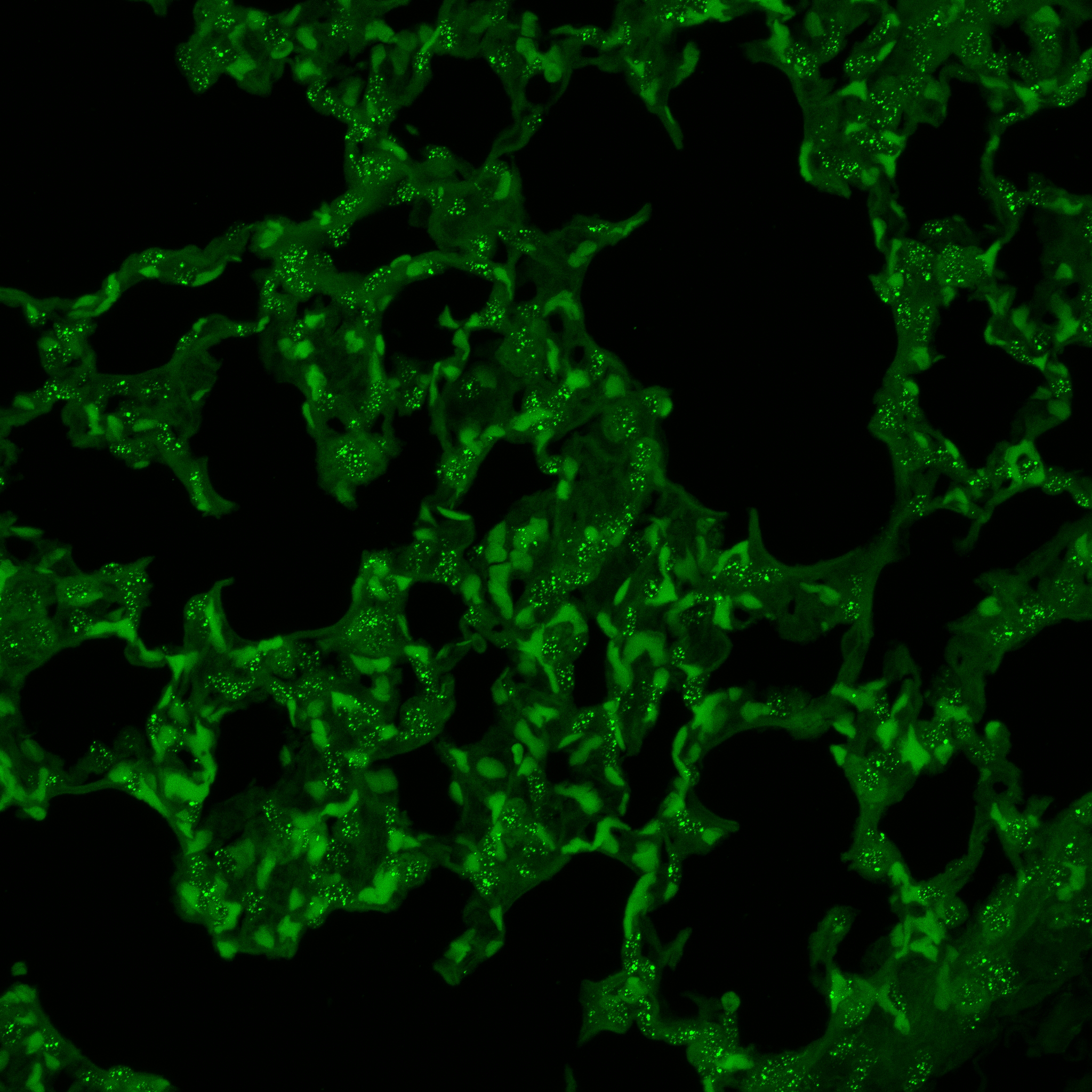

Supplement: Supplementary file 4 — Source Data Fig. 2 [file 44319_2023_41_MOESM4_ESM.zip › Source data Figure 2 /2B_Image data, Micr.image/Tel_p21+-lame965.nd2.tiff]

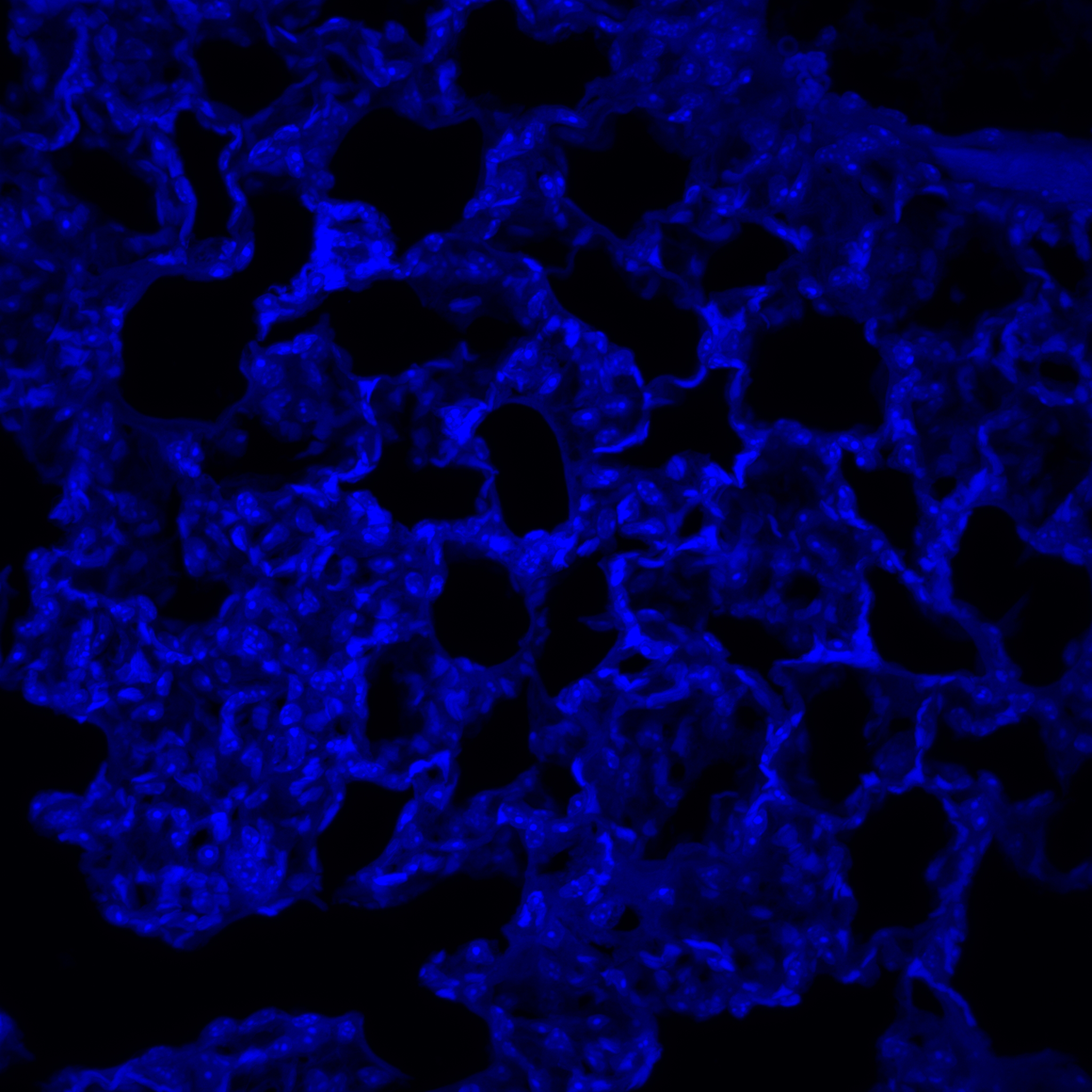

Supplement: Supplementary file 4 — Source Data Fig. 2 [file 44319_2023_41_MOESM4_ESM.zip › Source data Figure 2 /2B_Image data, Micr.image/DAPI_p21++lame945.nd2.tiff]

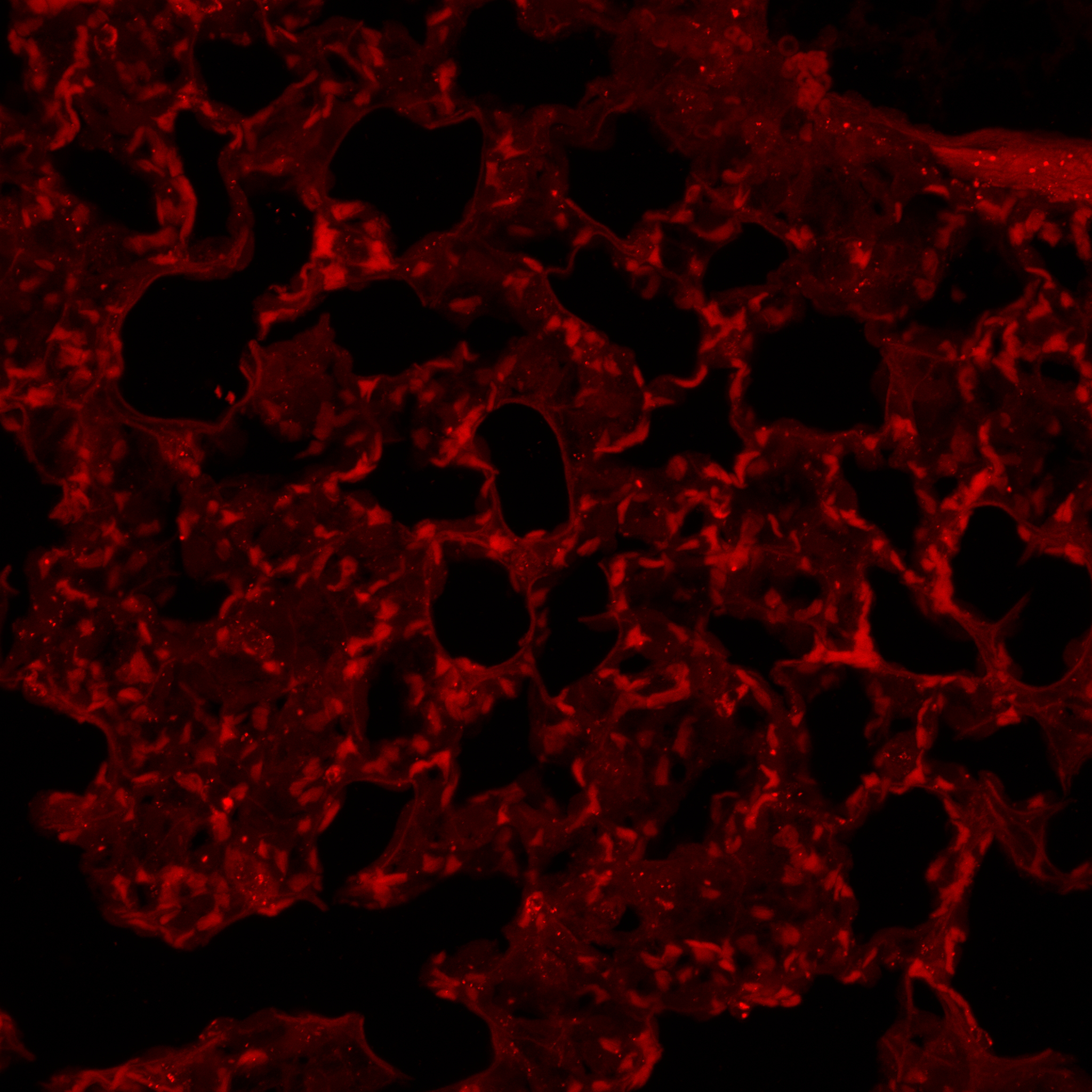

Supplement: Supplementary file 4 — Source Data Fig. 2 [file 44319_2023_41_MOESM4_ESM.zip › Source data Figure 2 /2B_Image data, Micr.image/53BP1_p21++lame945.nd2.tiff]

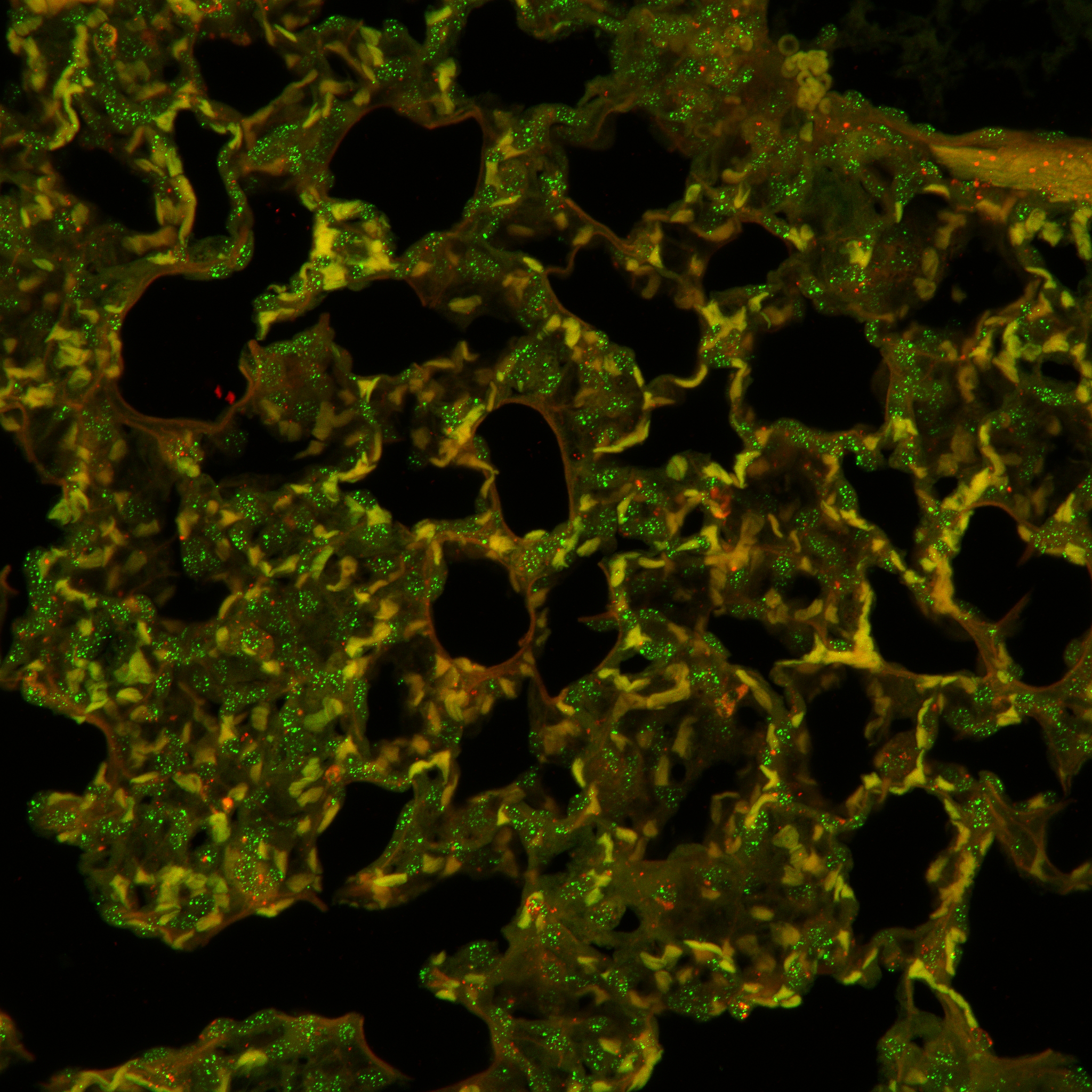

Supplement: Supplementary file 4 — Source Data Fig. 2 [file 44319_2023_41_MOESM4_ESM.zip › Source data Figure 2 /2B_Image data, Micr.image/TIFs_p21++lame945.nd2.tiff]

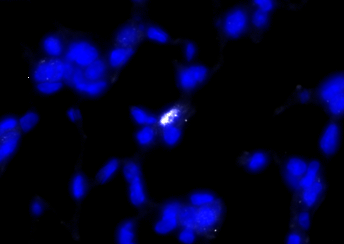

Supplement: Supplementary file 4 — Source Data Fig. 2 [file 44319_2023_41_MOESM4_ESM.zip › Source data Figure 2 /2A, Image data, Micr.image/Illustration images en TIFF/Young/p21+TERT CI zoom.tif]

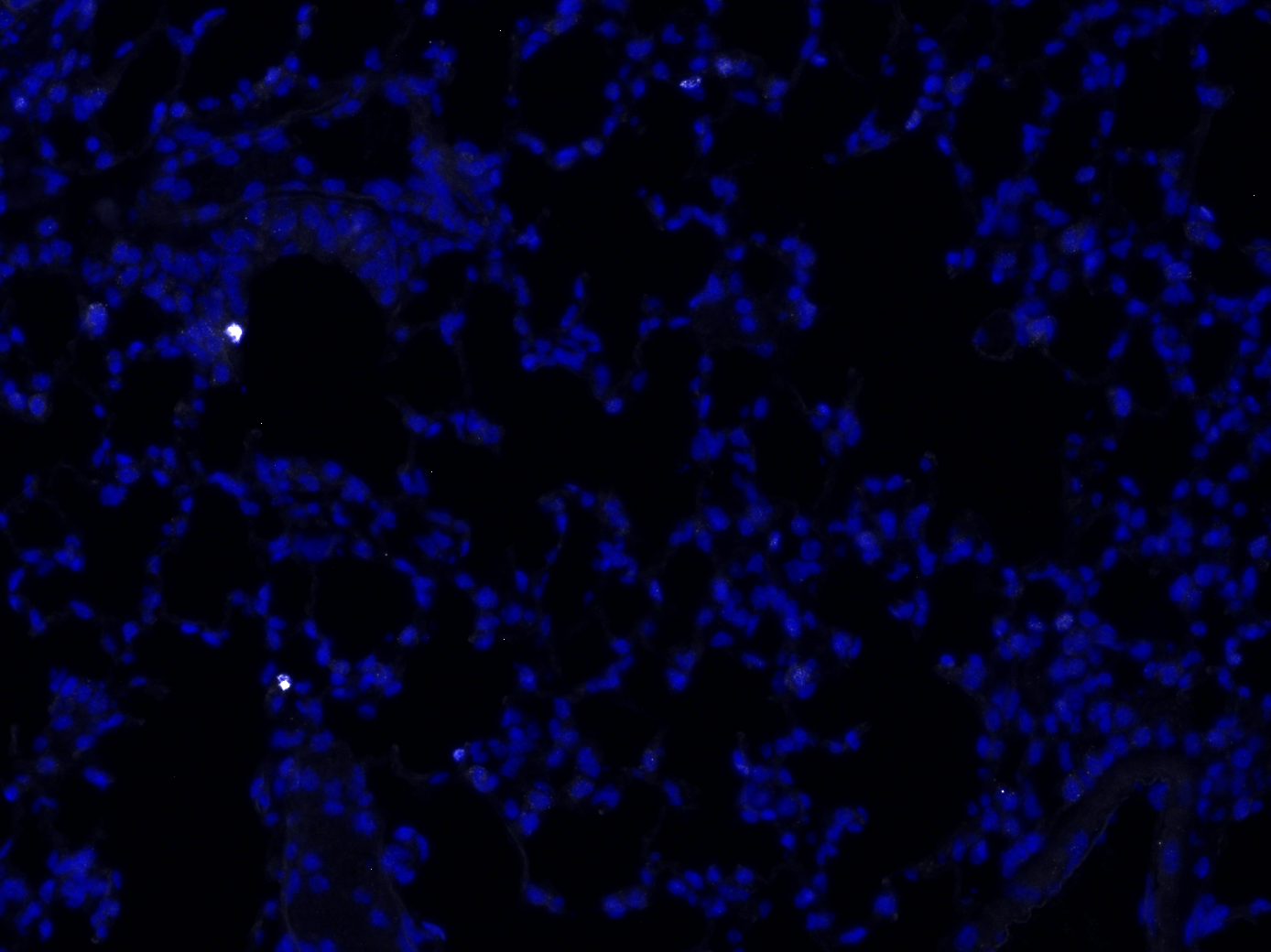

Supplement: Supplementary file 4 — Source Data Fig. 2 [file 44319_2023_41_MOESM4_ESM.zip › Source data Figure 2 /2A, Image data, Micr.image/Illustration images en TIFF/Young/p21+-.tif]

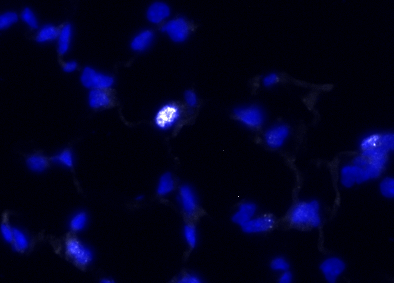

Supplement: Supplementary file 4 — Source Data Fig. 2 [file 44319_2023_41_MOESM4_ESM.zip › Source data Figure 2 /2A, Image data, Micr.image/Illustration images en TIFF/Young/p21+TERT zoom.tif]

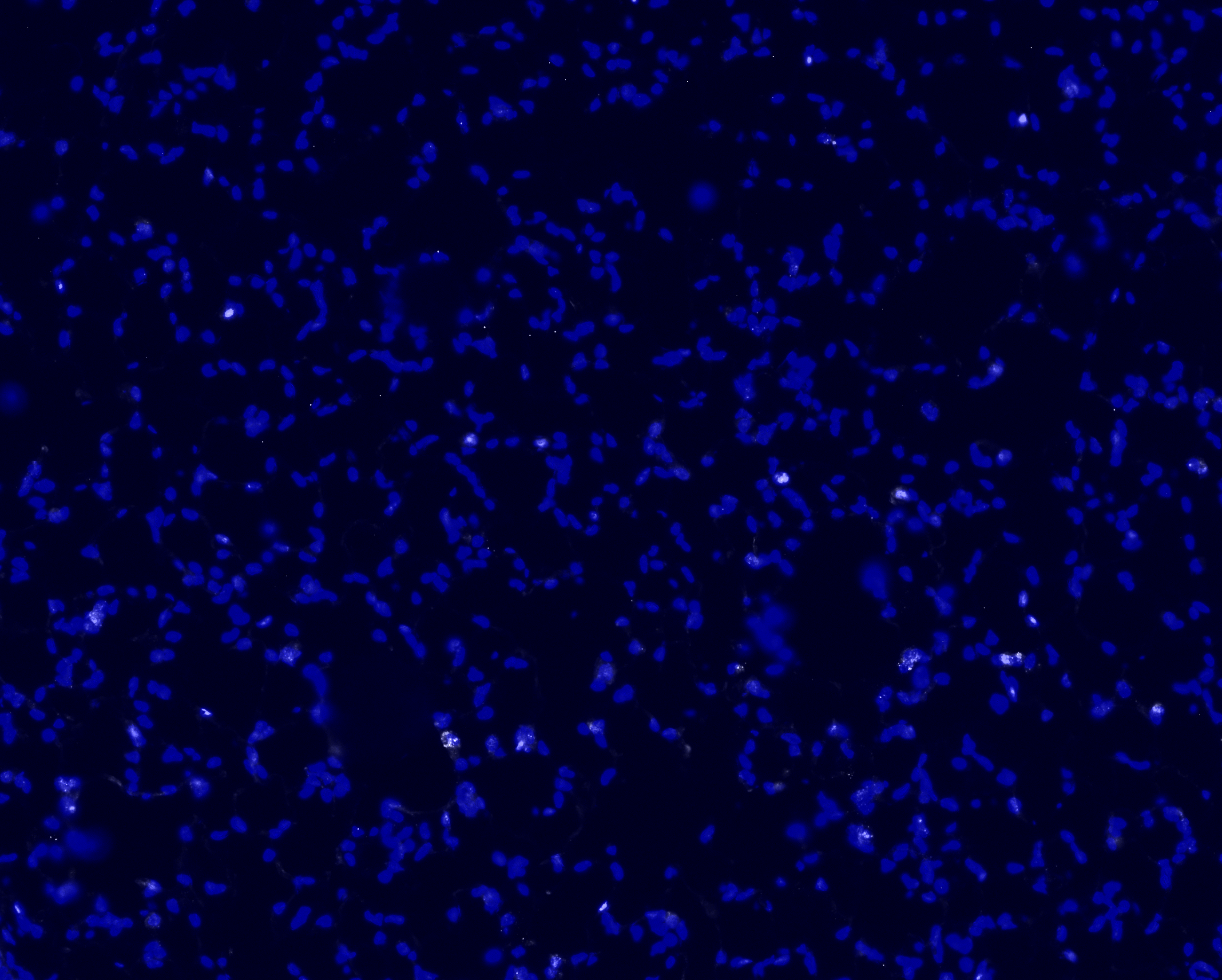

Supplement: Supplementary file 4 — Source Data Fig. 2 [file 44319_2023_41_MOESM4_ESM.zip › Source data Figure 2 /2A, Image data, Micr.image/Illustration images en TIFF/Young/p21++.tif]

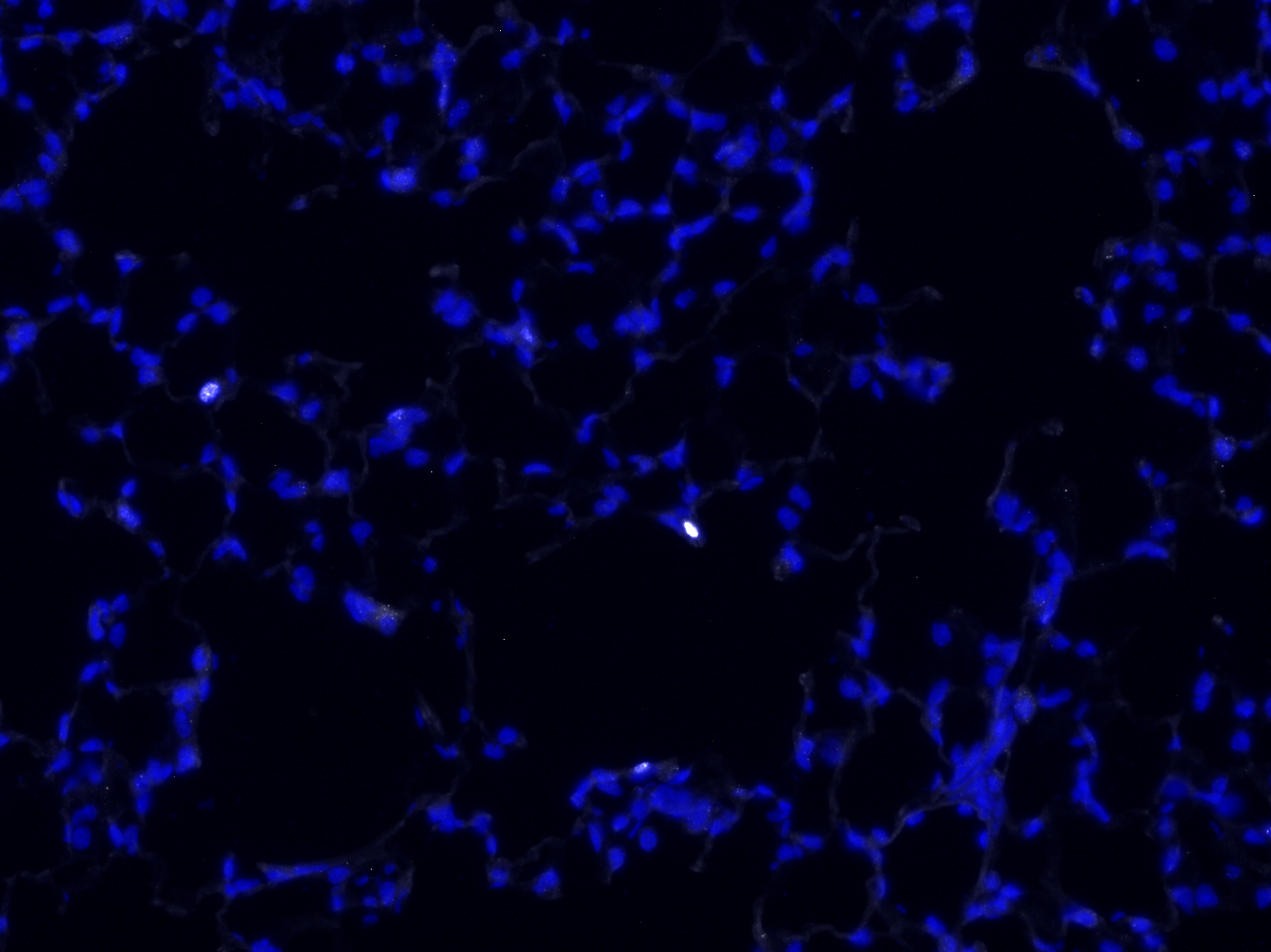

Supplement: Supplementary file 4 — Source Data Fig. 2 [file 44319_2023_41_MOESM4_ESM.zip › Source data Figure 2 /2A, Image data, Micr.image/Illustration images en TIFF/Young/p21+TERT.tif]

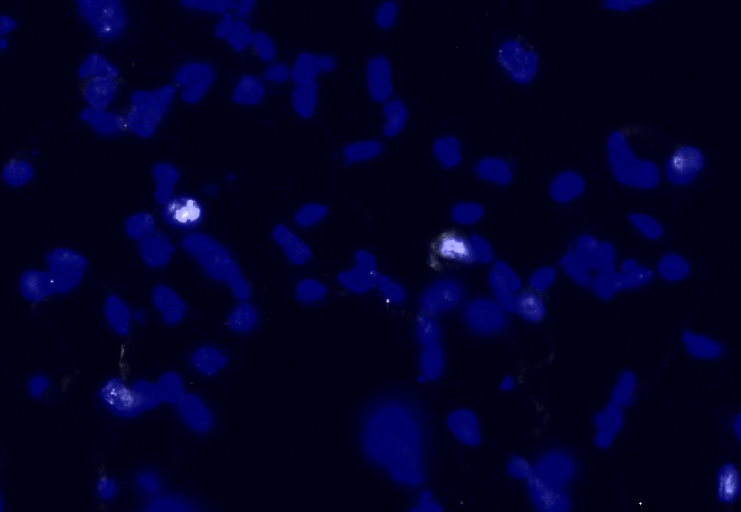

Supplement: Supplementary file 4 — Source Data Fig. 2 [file 44319_2023_41_MOESM4_ESM.zip › Source data Figure 2 /2A, Image data, Micr.image/Illustration images en TIFF/Young/p21++ zoom.tif]

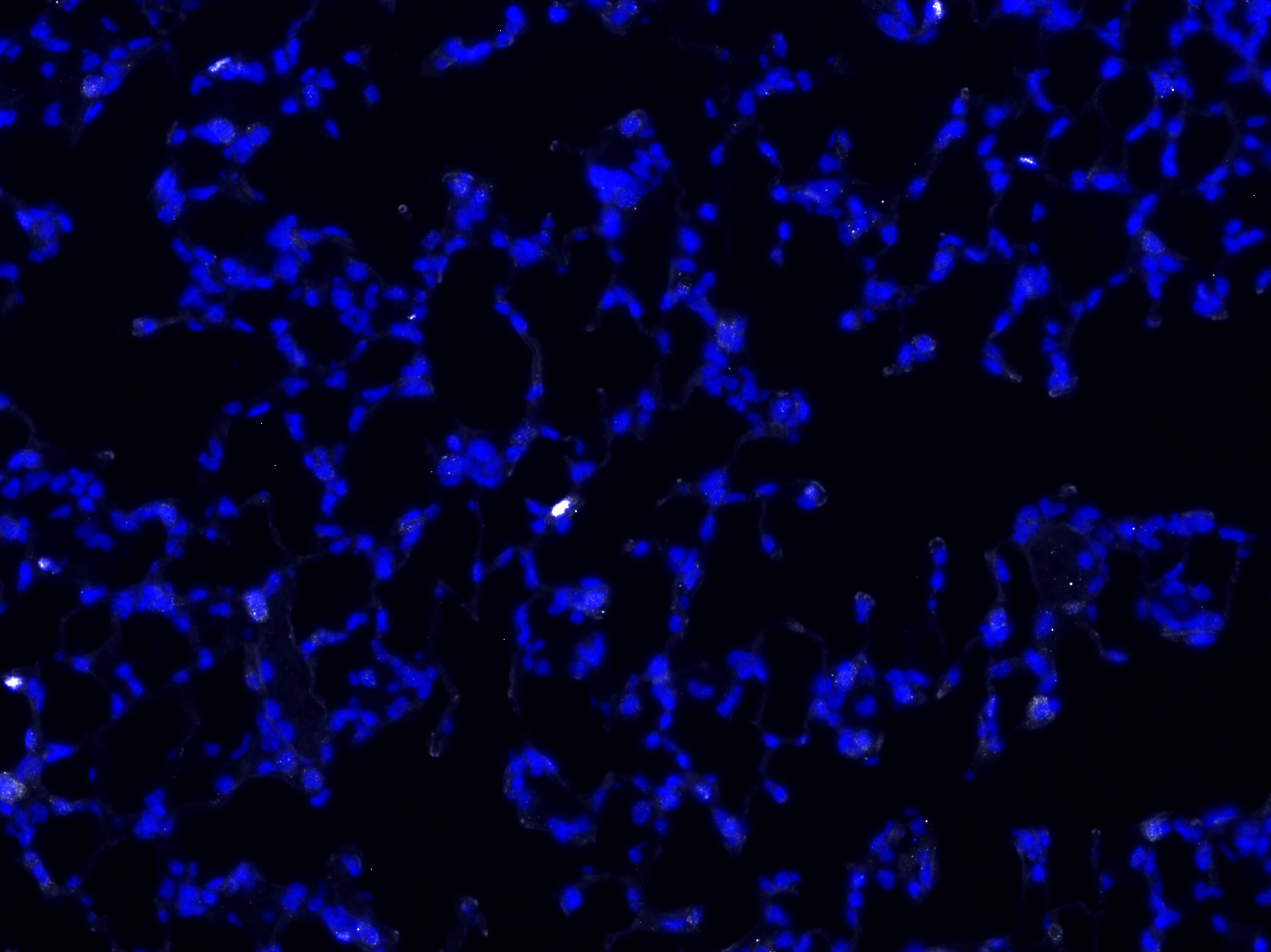

Supplement: Supplementary file 4 — Source Data Fig. 2 [file 44319_2023_41_MOESM4_ESM.zip › Source data Figure 2 /2A, Image data, Micr.image/Illustration images en TIFF/Young/p21+ TERT CI.tif]

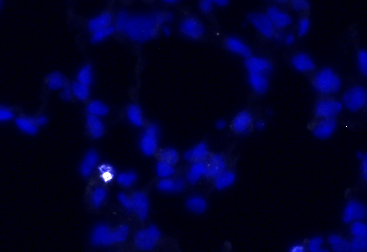

Supplement: Supplementary file 4 — Source Data Fig. 2 [file 44319_2023_41_MOESM4_ESM.zip › Source data Figure 2 /2A, Image data, Micr.image/Illustration images en TIFF/Young/p21+- zoom.tif]

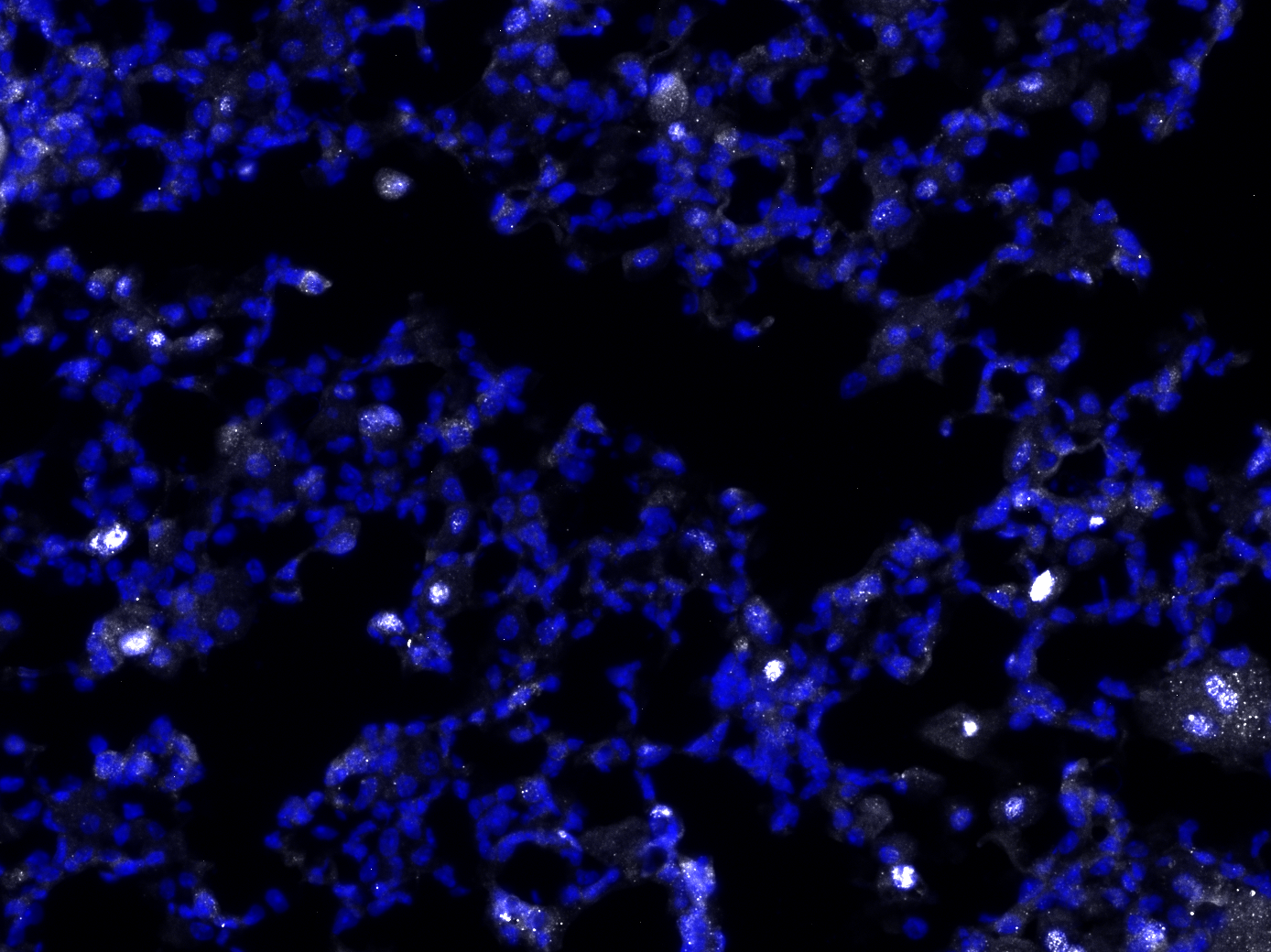

Supplement: Supplementary file 4 — Source Data Fig. 2 [file 44319_2023_41_MOESM4_ESM.zip › Source data Figure 2 /2A, Image data, Micr.image/Illustration images en TIFF/Aged/p21+-.tif]

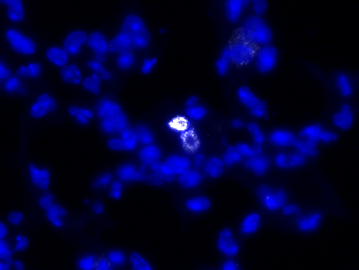

Supplement: Supplementary file 4 — Source Data Fig. 2 [file 44319_2023_41_MOESM4_ESM.zip › Source data Figure 2 /2A, Image data, Micr.image/Illustration images en TIFF/Aged/p21+TERT zoom.tif]

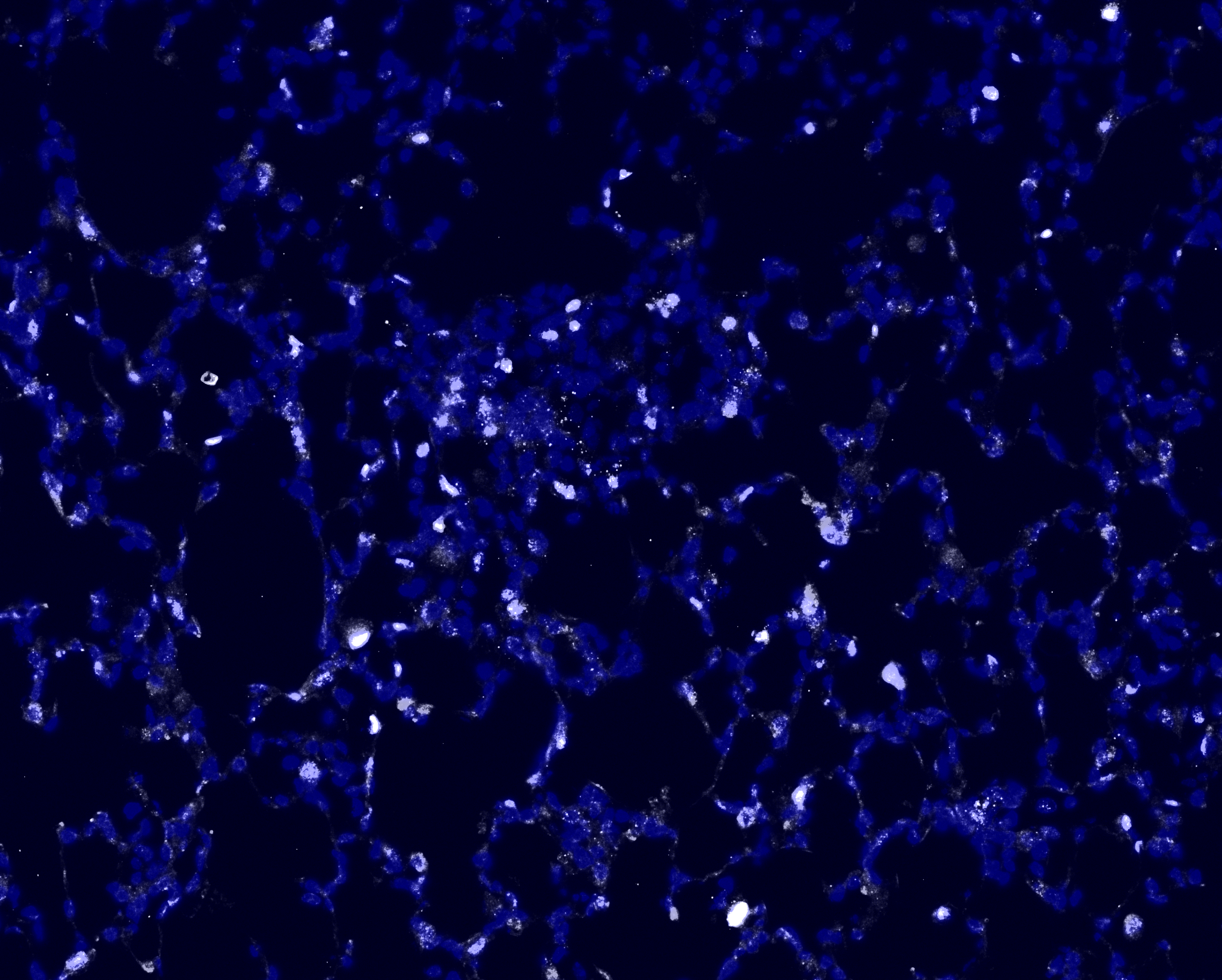

Supplement: Supplementary file 4 — Source Data Fig. 2 [file 44319_2023_41_MOESM4_ESM.zip › Source data Figure 2 /2A, Image data, Micr.image/Illustration images en TIFF/Aged/p21++.tif]

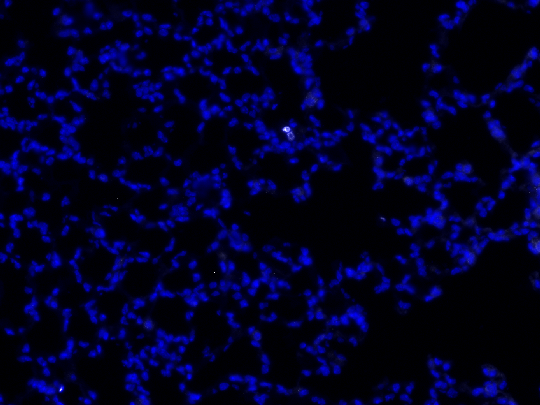

Supplement: Supplementary file 4 — Source Data Fig. 2 [file 44319_2023_41_MOESM4_ESM.zip › Source data Figure 2 /2A, Image data, Micr.image/Illustration images en TIFF/Aged/p21+TERT.tif]

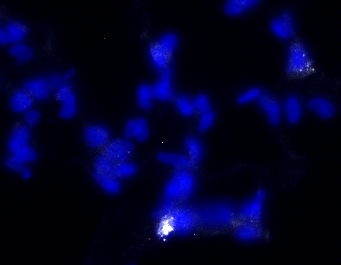

Supplement: Supplementary file 4 — Source Data Fig. 2 [file 44319_2023_41_MOESM4_ESM.zip › Source data Figure 2 /2A, Image data, Micr.image/Illustration images en TIFF/Aged/p21+ TERT CI zoom.tif]

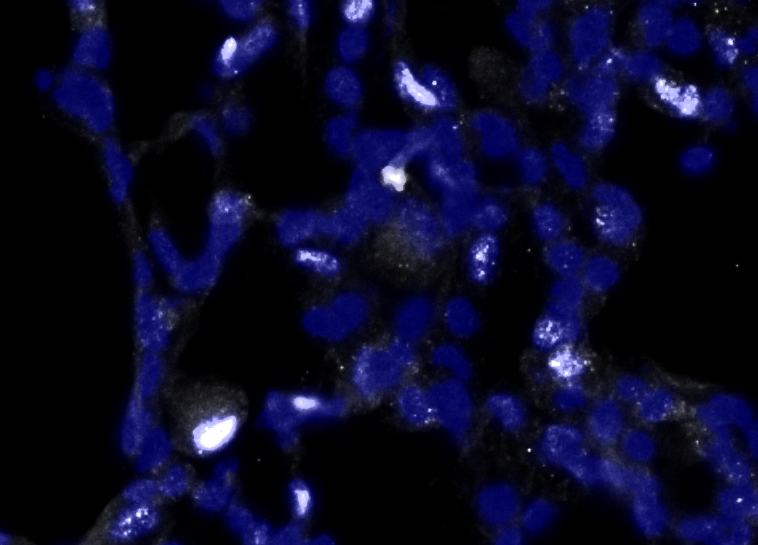

Supplement: Supplementary file 4 — Source Data Fig. 2 [file 44319_2023_41_MOESM4_ESM.zip › Source data Figure 2 /2A, Image data, Micr.image/Illustration images en TIFF/Aged/p21++ zoom.tif]

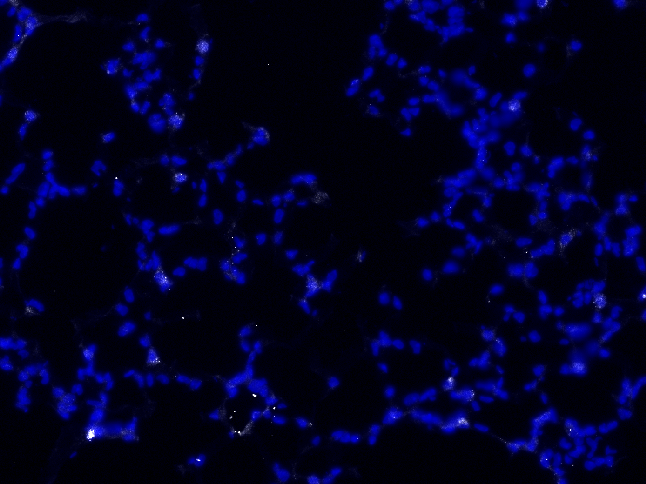

Supplement: Supplementary file 4 — Source Data Fig. 2 [file 44319_2023_41_MOESM4_ESM.zip › Source data Figure 2 /2A, Image data, Micr.image/Illustration images en TIFF/Aged/p21+ TERT CI.tif]

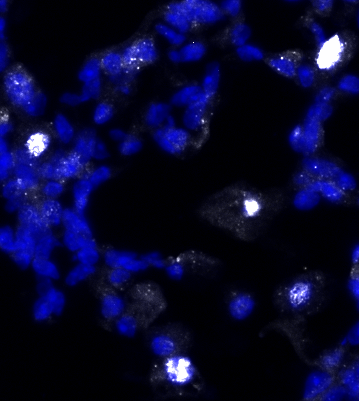

Supplement: Supplementary file 4 — Source Data Fig. 2 [file 44319_2023_41_MOESM4_ESM.zip › Source data Figure 2 /2A, Image data, Micr.image/Illustration images en TIFF/Aged/p21+- zoom.tif]

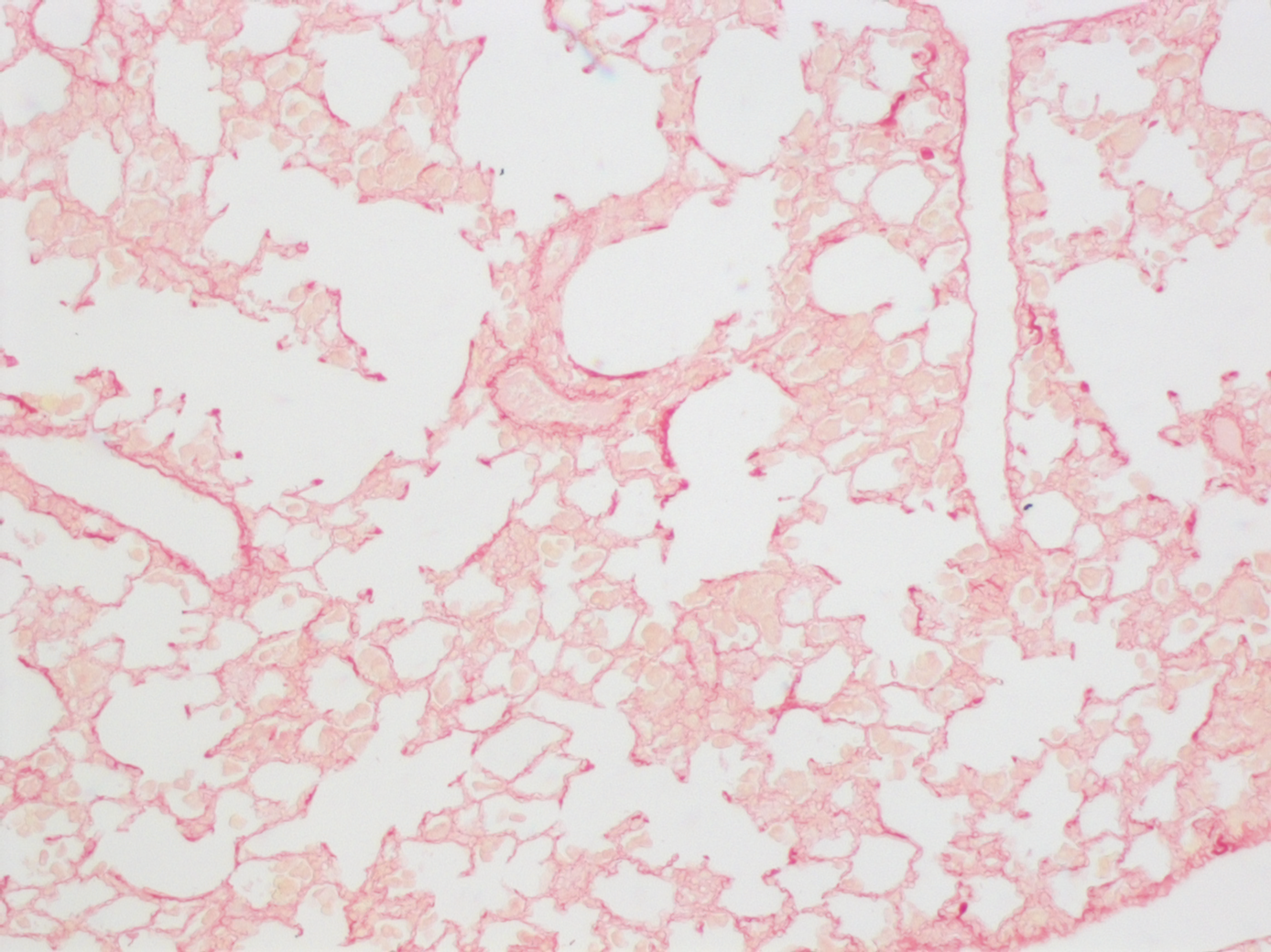

Supplement: Supplementary file 5 — Source Data Fig. 3 [file 44319_2023_41_MOESM5_ESM.zip › Source data Figure 3 /3C Image data, micr. image/SR/p21+-.tif]

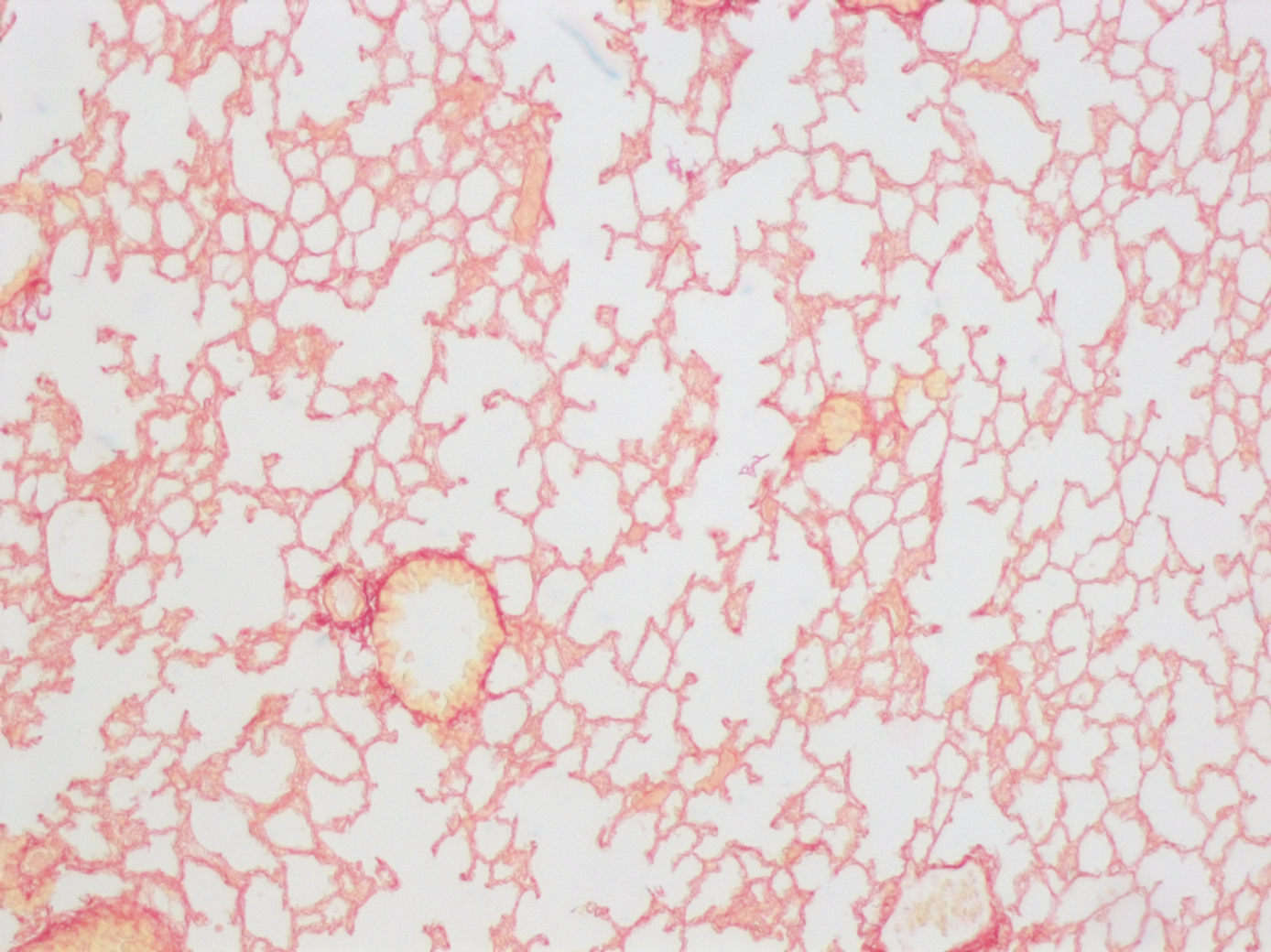

Supplement: Supplementary file 5 — Source Data Fig. 3 [file 44319_2023_41_MOESM5_ESM.zip › Source data Figure 3 /3C Image data, micr. image/SR/p21++.tif]

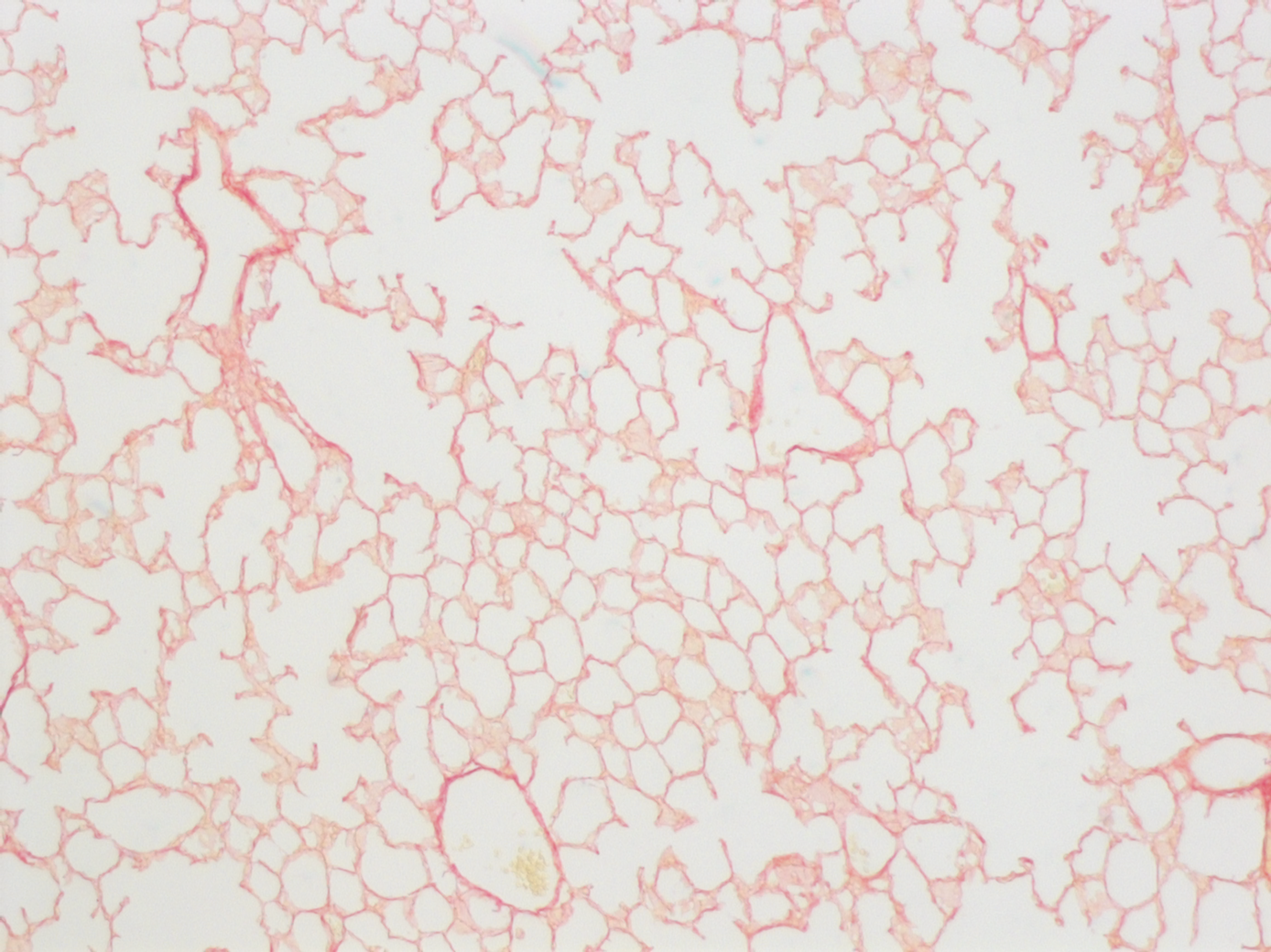

Supplement: Supplementary file 5 — Source Data Fig. 3 [file 44319_2023_41_MOESM5_ESM.zip › Source data Figure 3 /3C Image data, micr. image/SR/p21+TERT.tif]

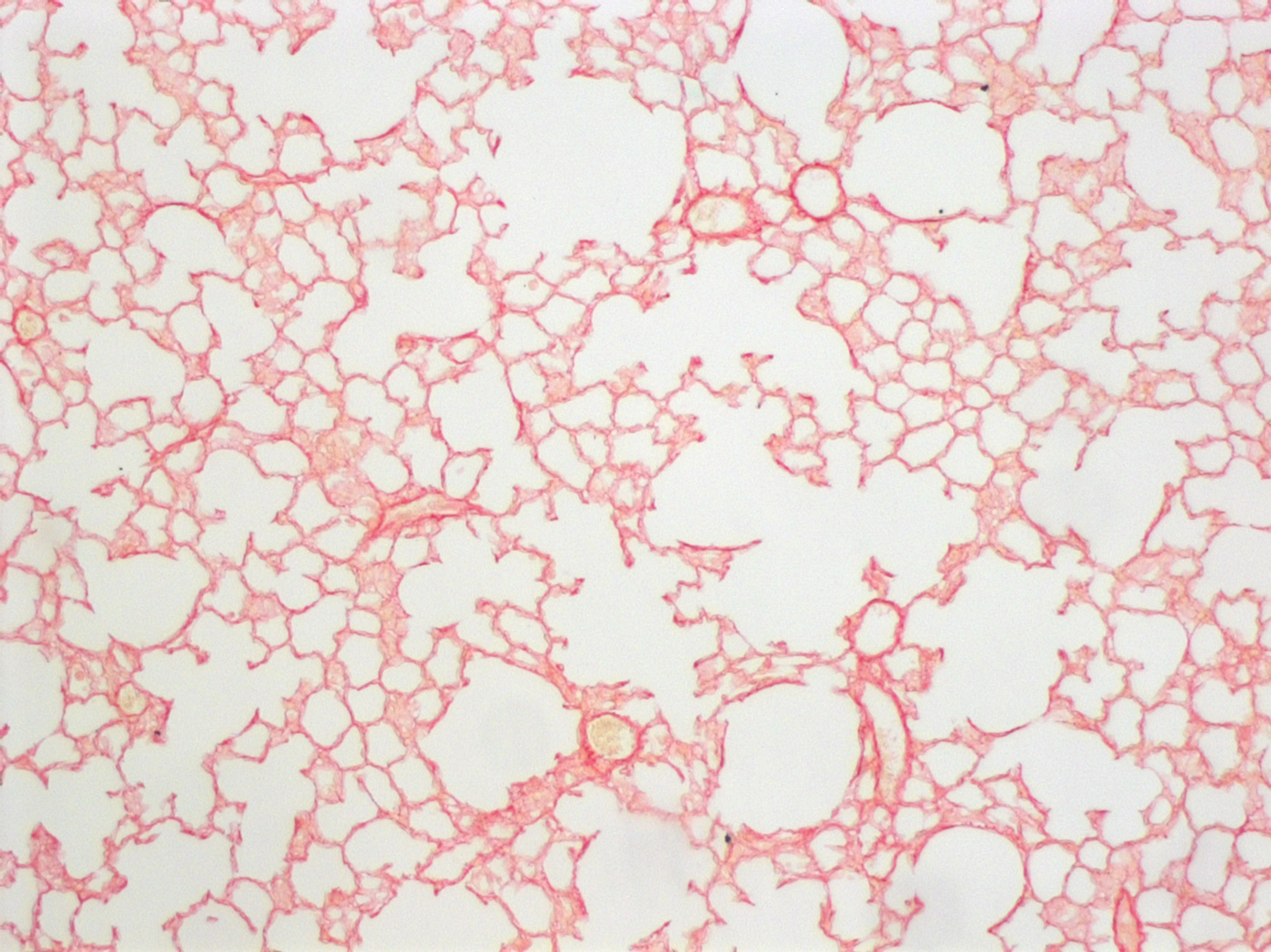

Supplement: Supplementary file 5 — Source Data Fig. 3 [file 44319_2023_41_MOESM5_ESM.zip › Source data Figure 3 /3C Image data, micr. image/SR/p21+TERT CI.tif]

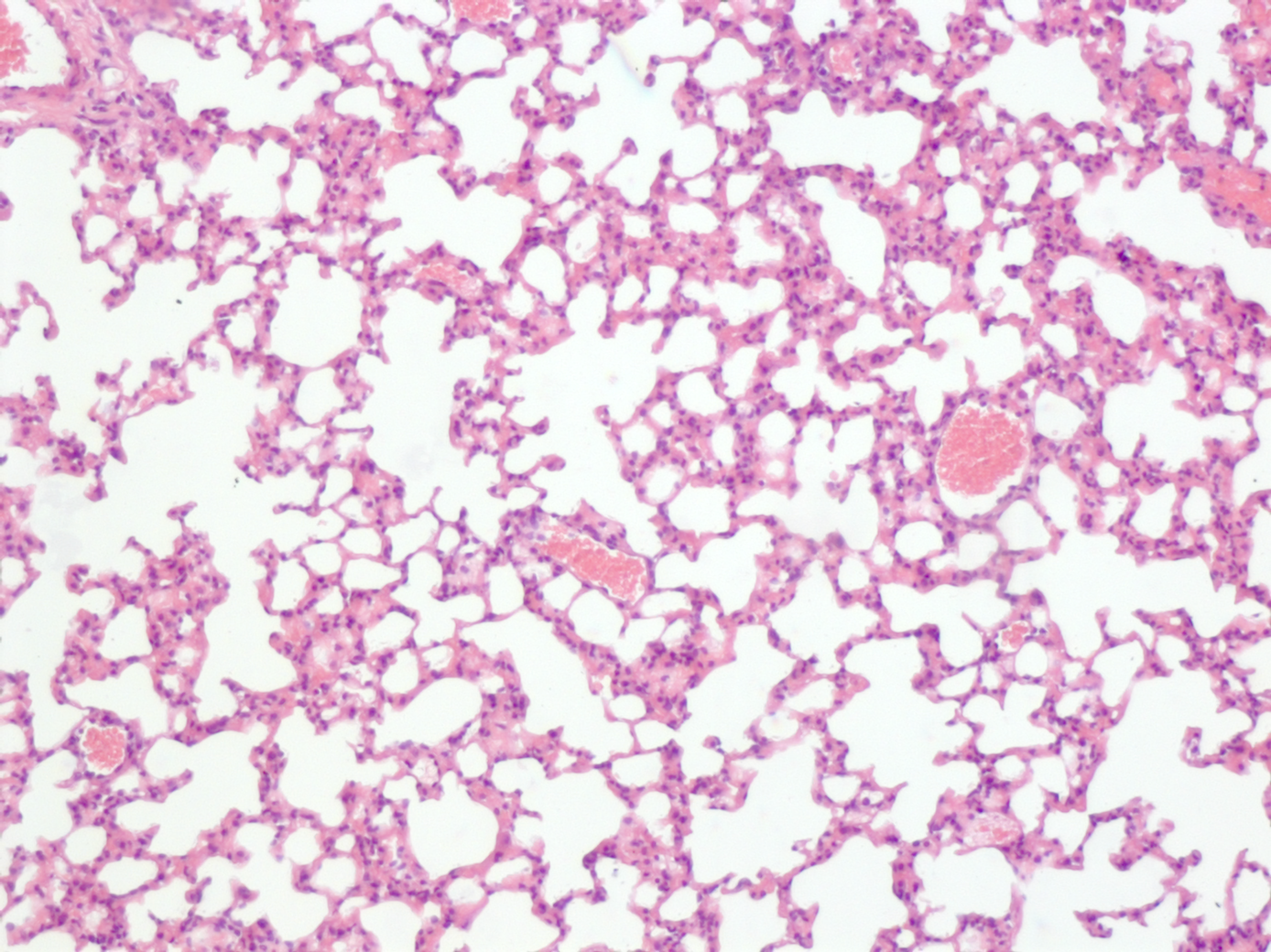

Supplement: Supplementary file 5 — Source Data Fig. 3 [file 44319_2023_41_MOESM5_ESM.zip › Source data Figure 3 /3A Image data, micr. image/young/p21+-.tif]

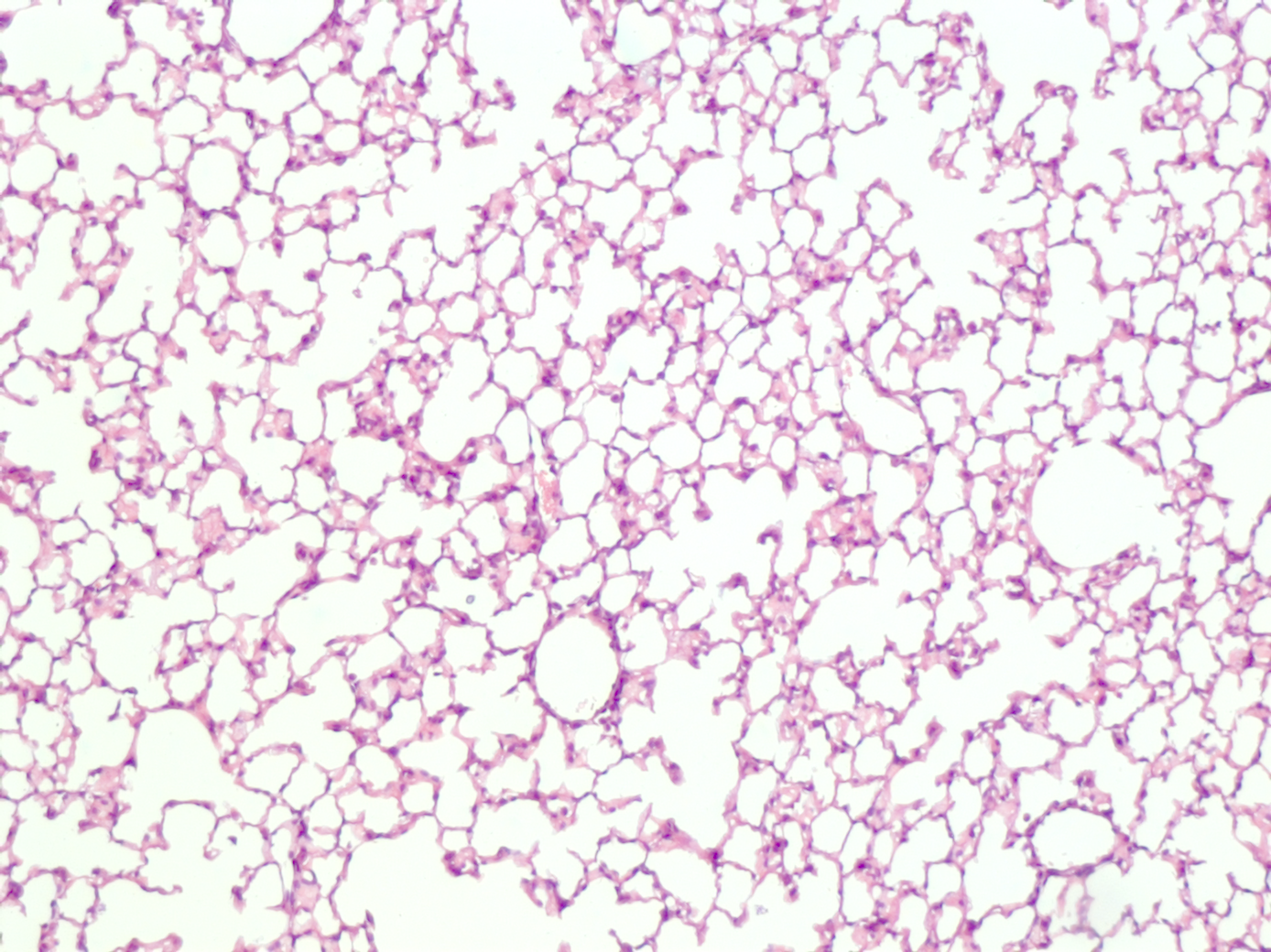

Supplement: Supplementary file 5 — Source Data Fig. 3 [file 44319_2023_41_MOESM5_ESM.zip › Source data Figure 3 /3A Image data, micr. image/young/p21++.tif]

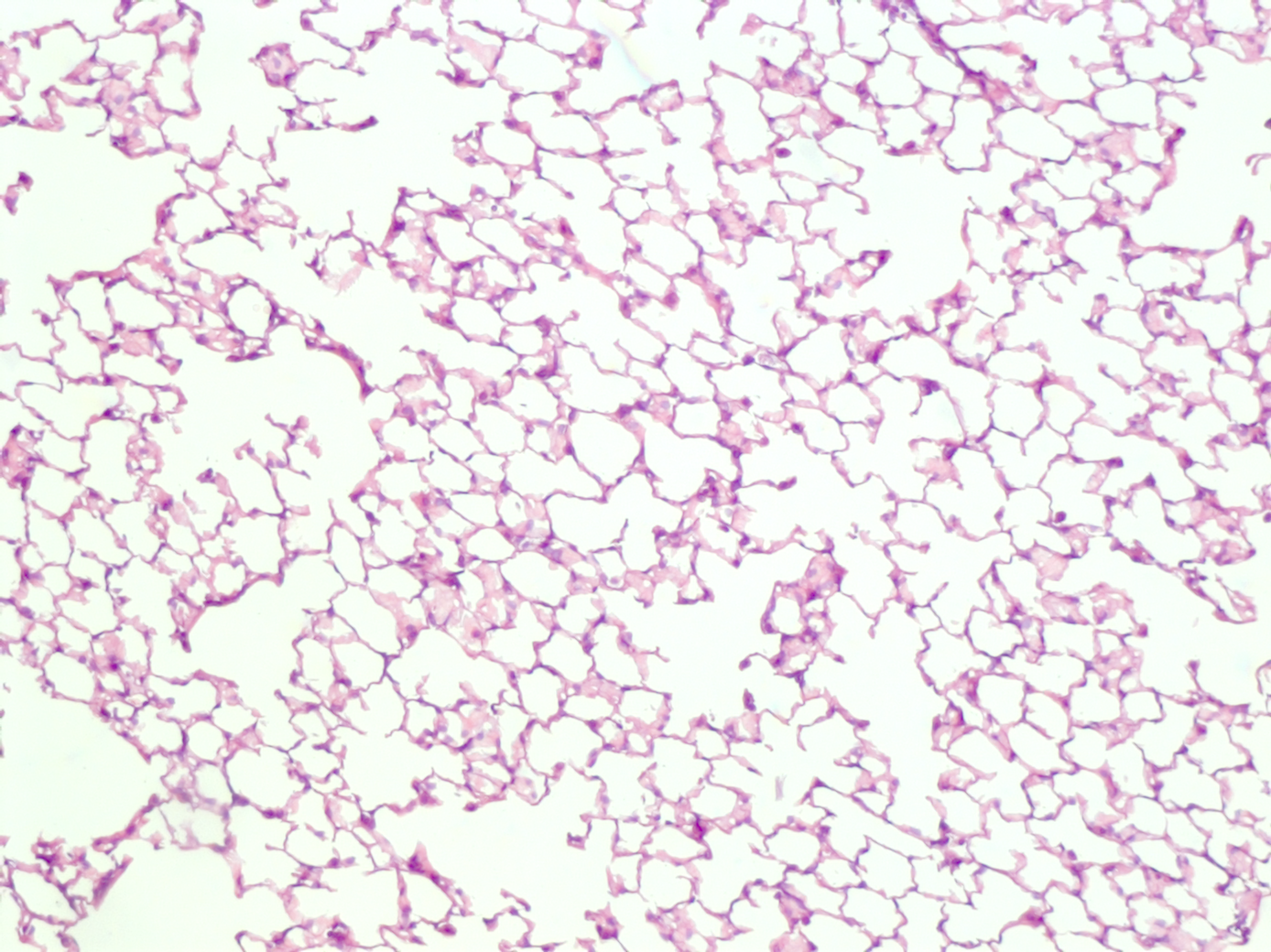

Supplement: Supplementary file 5 — Source Data Fig. 3 [file 44319_2023_41_MOESM5_ESM.zip › Source data Figure 3 /3A Image data, micr. image/young/p21+TERT.tif]

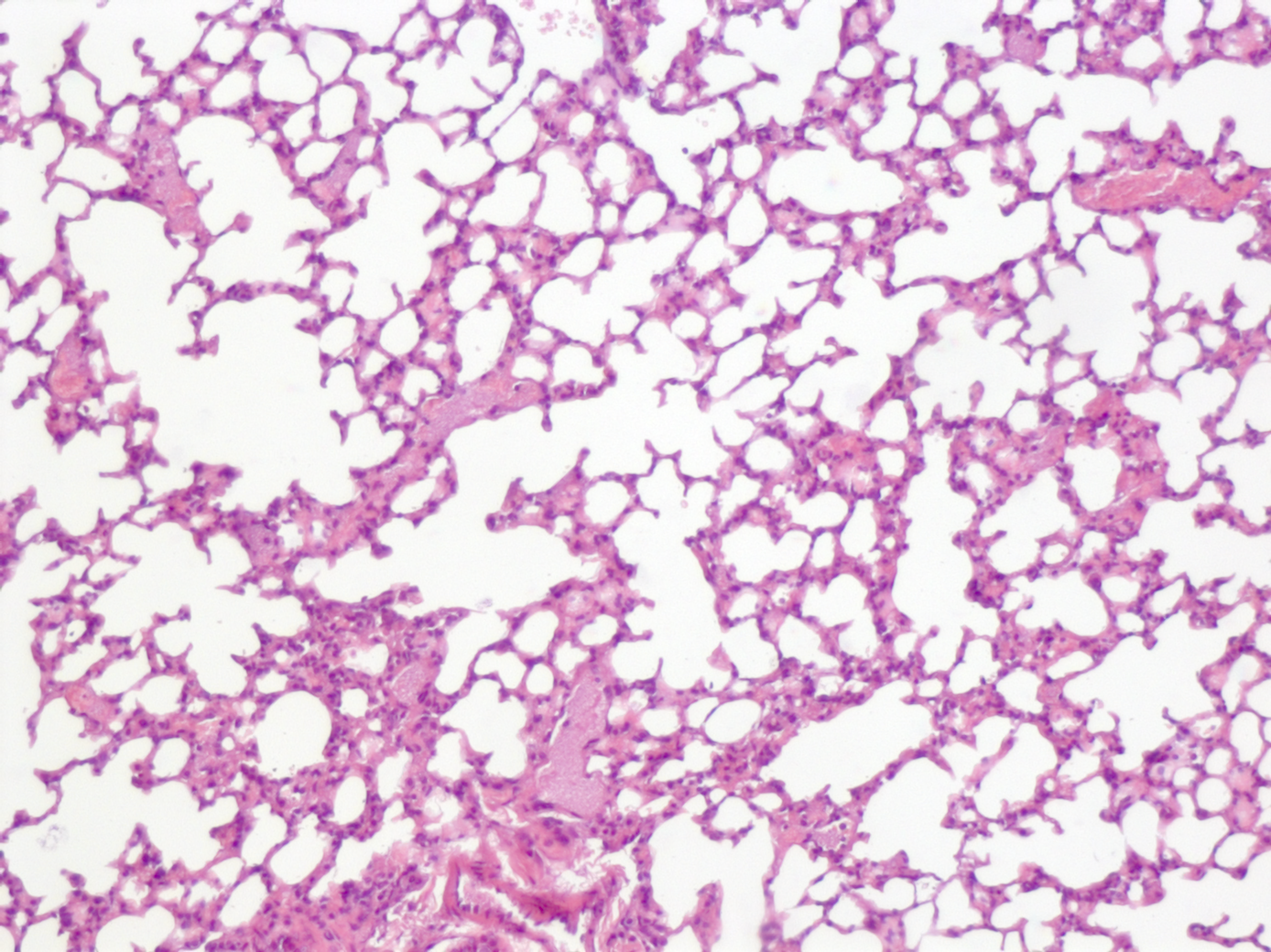

Supplement: Supplementary file 5 — Source Data Fig. 3 [file 44319_2023_41_MOESM5_ESM.zip › Source data Figure 3 /3A Image data, micr. image/young/p21+TERT CI.tif]

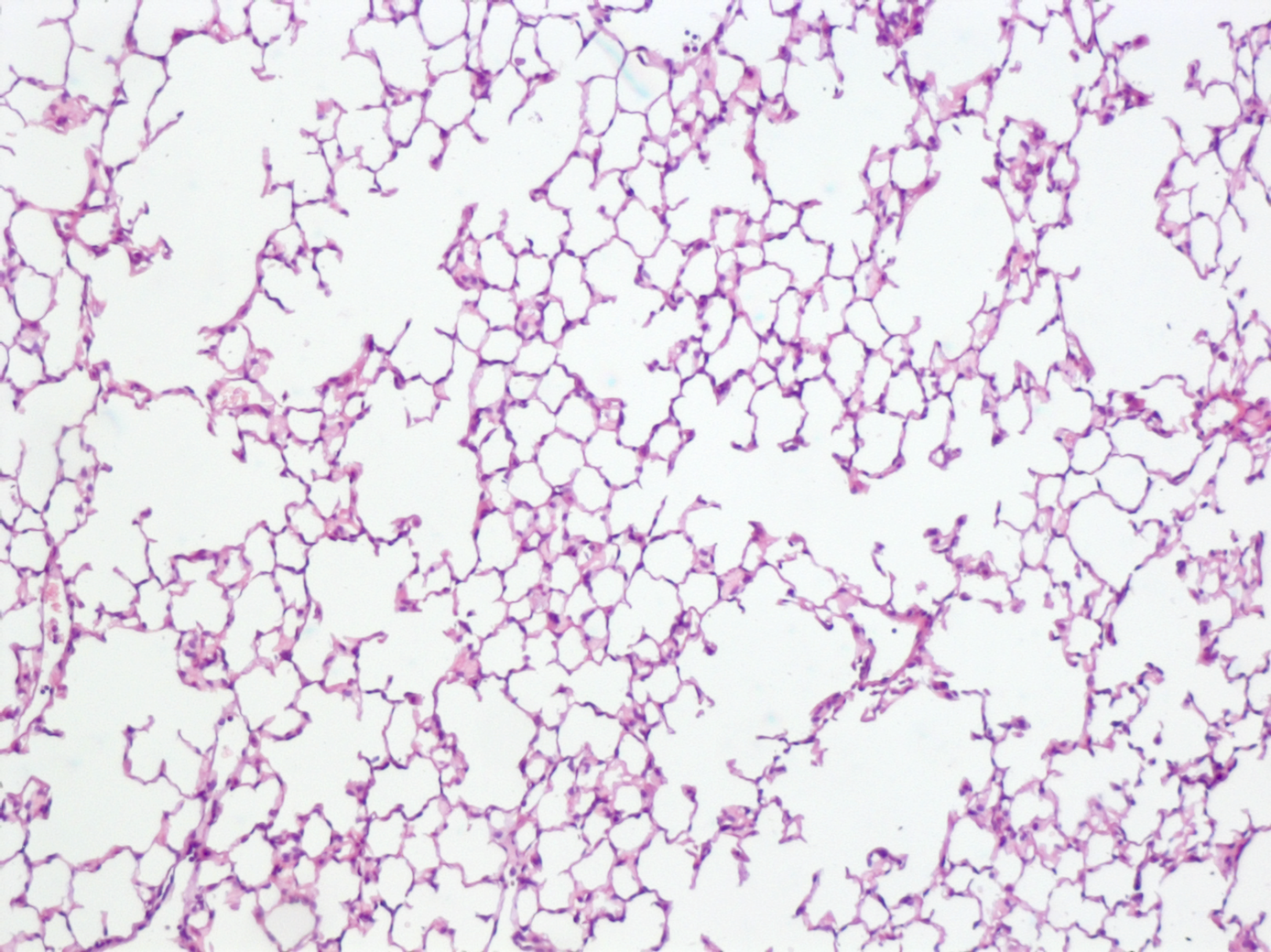

Supplement: Supplementary file 5 — Source Data Fig. 3 [file 44319_2023_41_MOESM5_ESM.zip › Source data Figure 3 /3A Image data, micr. image/aged/p21++.tif]

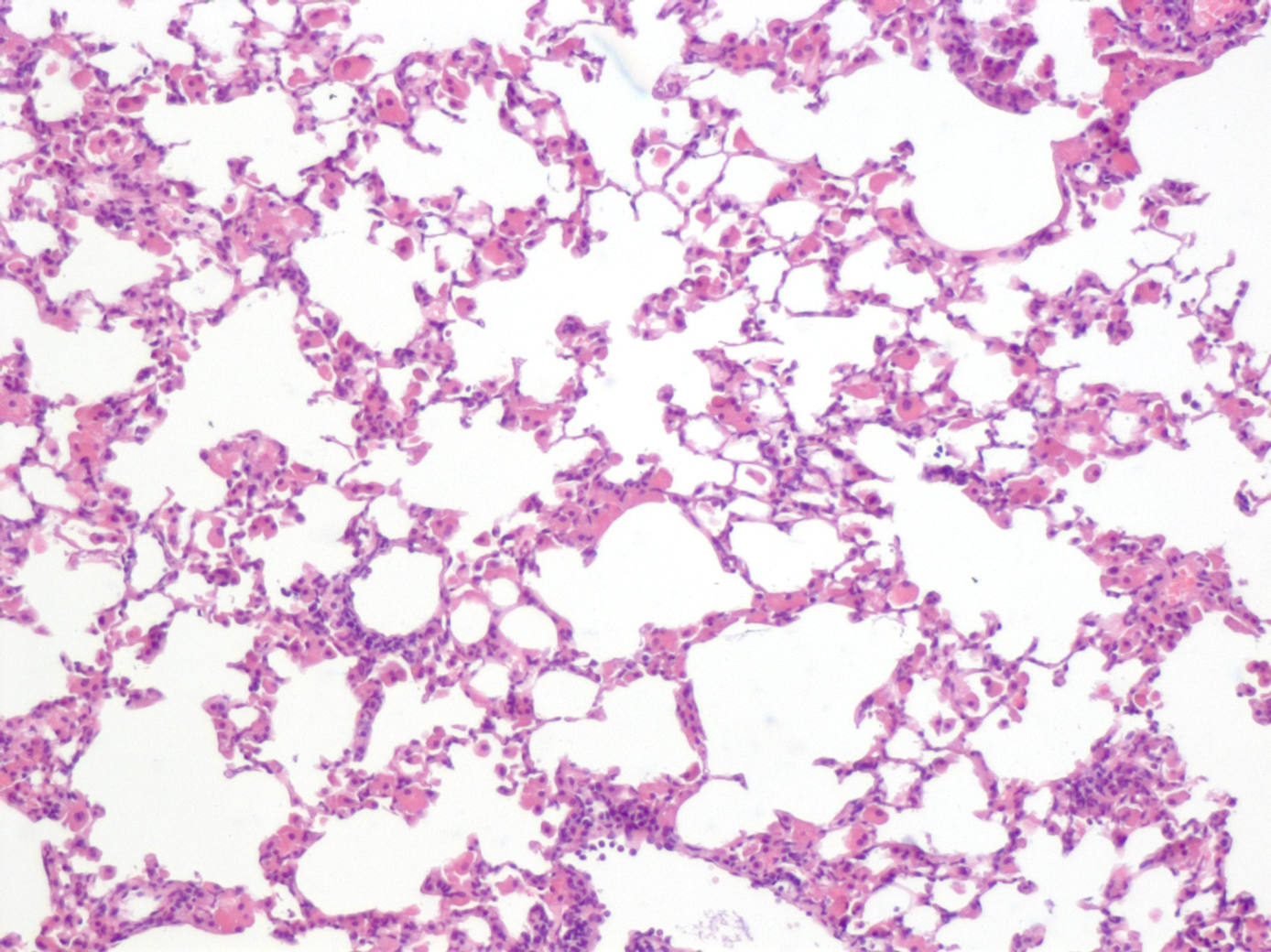

Supplement: Supplementary file 5 — Source Data Fig. 3 [file 44319_2023_41_MOESM5_ESM.zip › Source data Figure 3 /3A Image data, micr. image/aged/p21 ┬».tif]

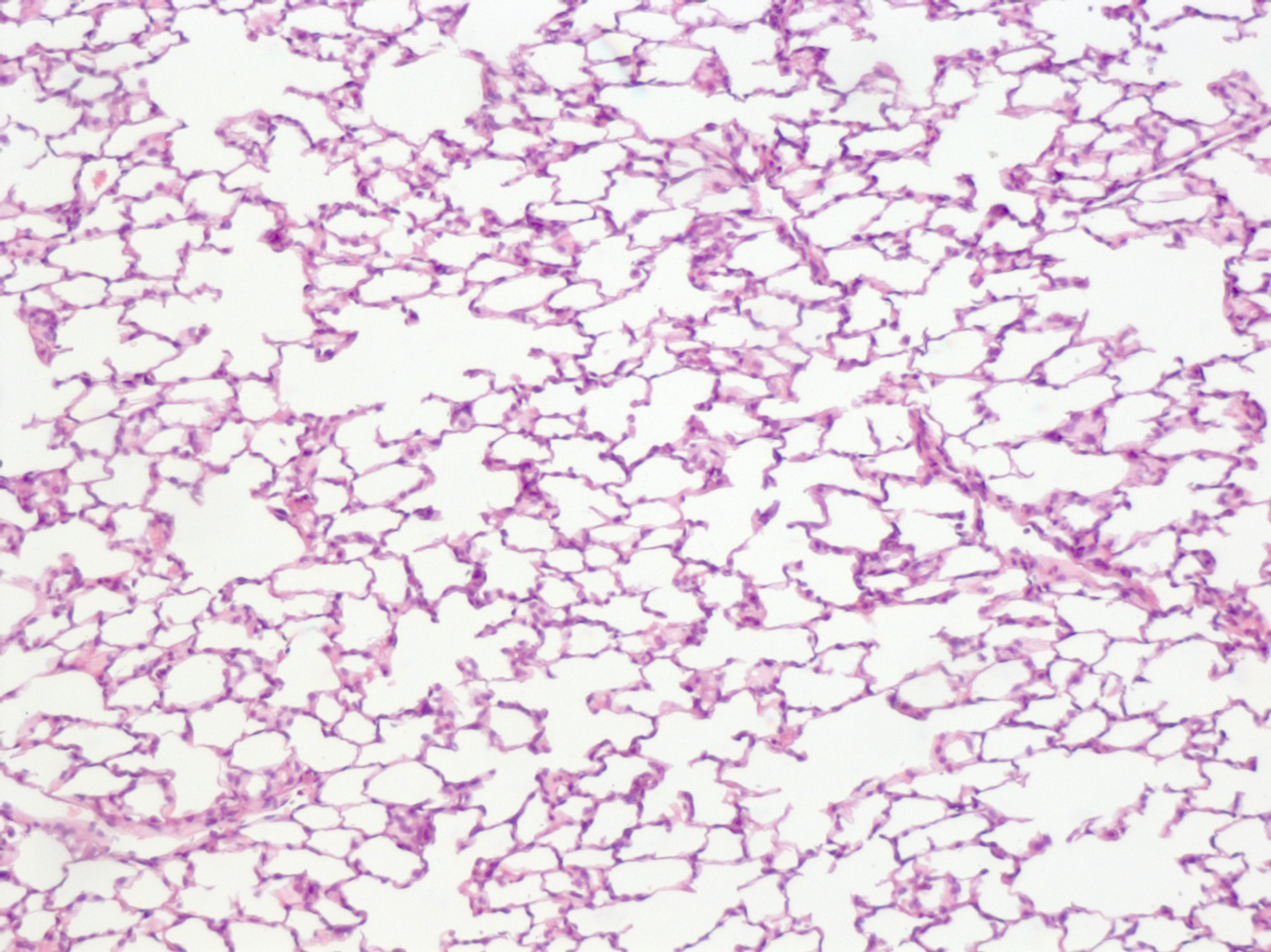

Supplement: Supplementary file 5 — Source Data Fig. 3 [file 44319_2023_41_MOESM5_ESM.zip › Source data Figure 3 /3A Image data, micr. image/aged/p21+-TERT.tif]

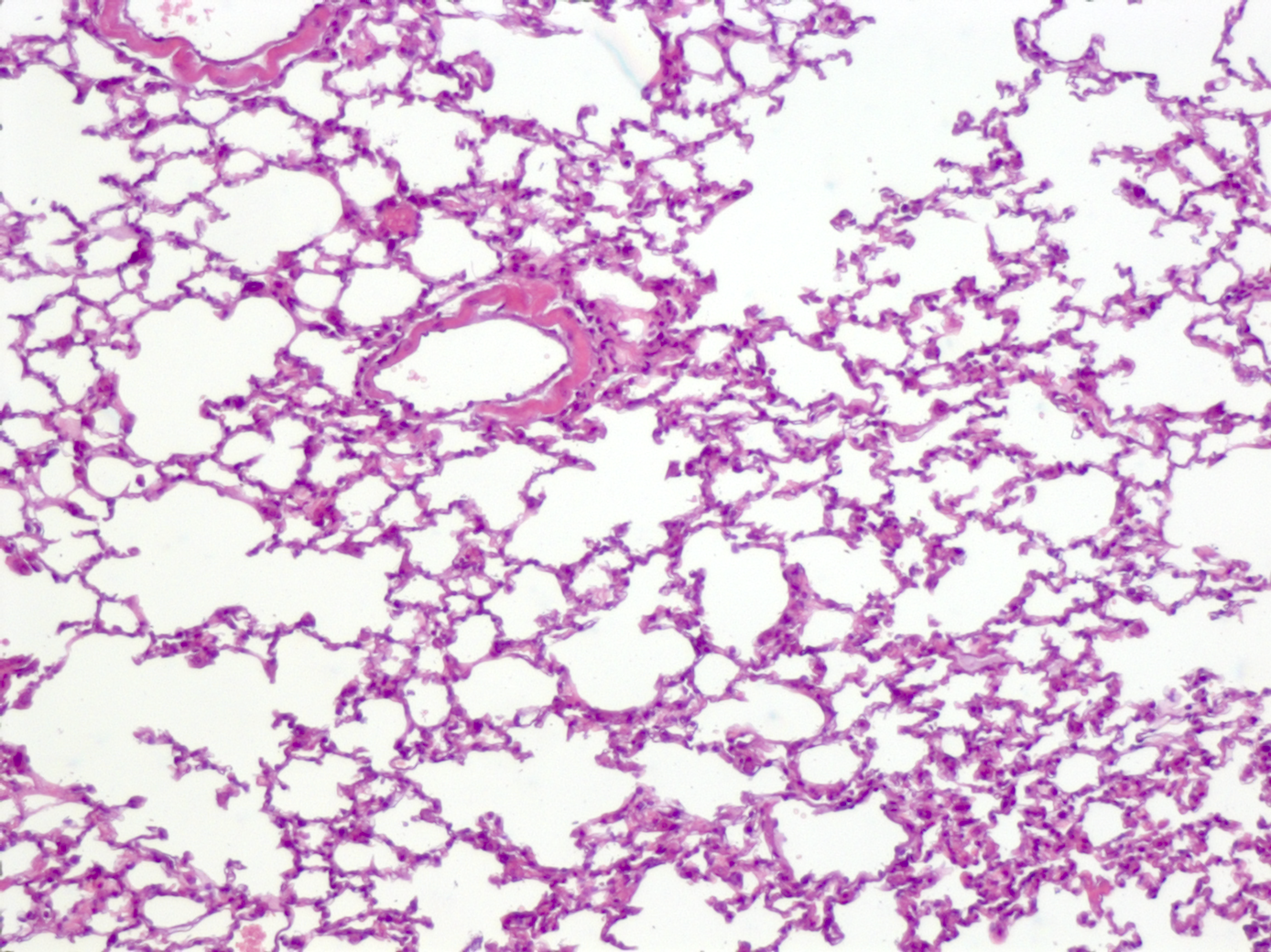

Supplement: Supplementary file 5 — Source Data Fig. 3 [file 44319_2023_41_MOESM5_ESM.zip › Source data Figure 3 /3A Image data, micr. image/aged/p21+TERT CI.tif]

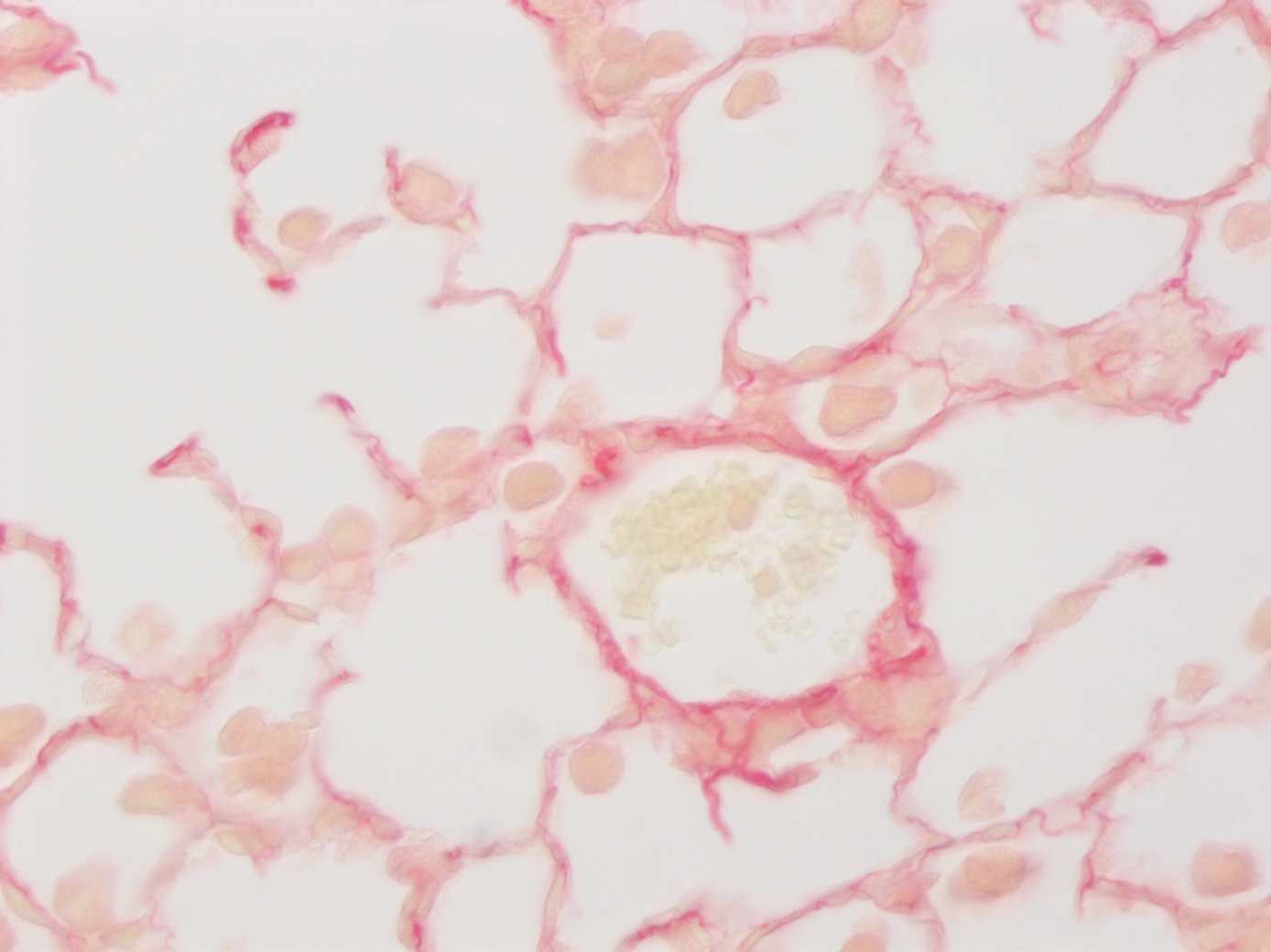

Supplement: Supplementary file 5 — Source Data Fig. 3 [file 44319_2023_41_MOESM5_ESM.zip › Source data Figure 3 /3E Image data, micr. image/Vessels, SR/p21+-.tif]

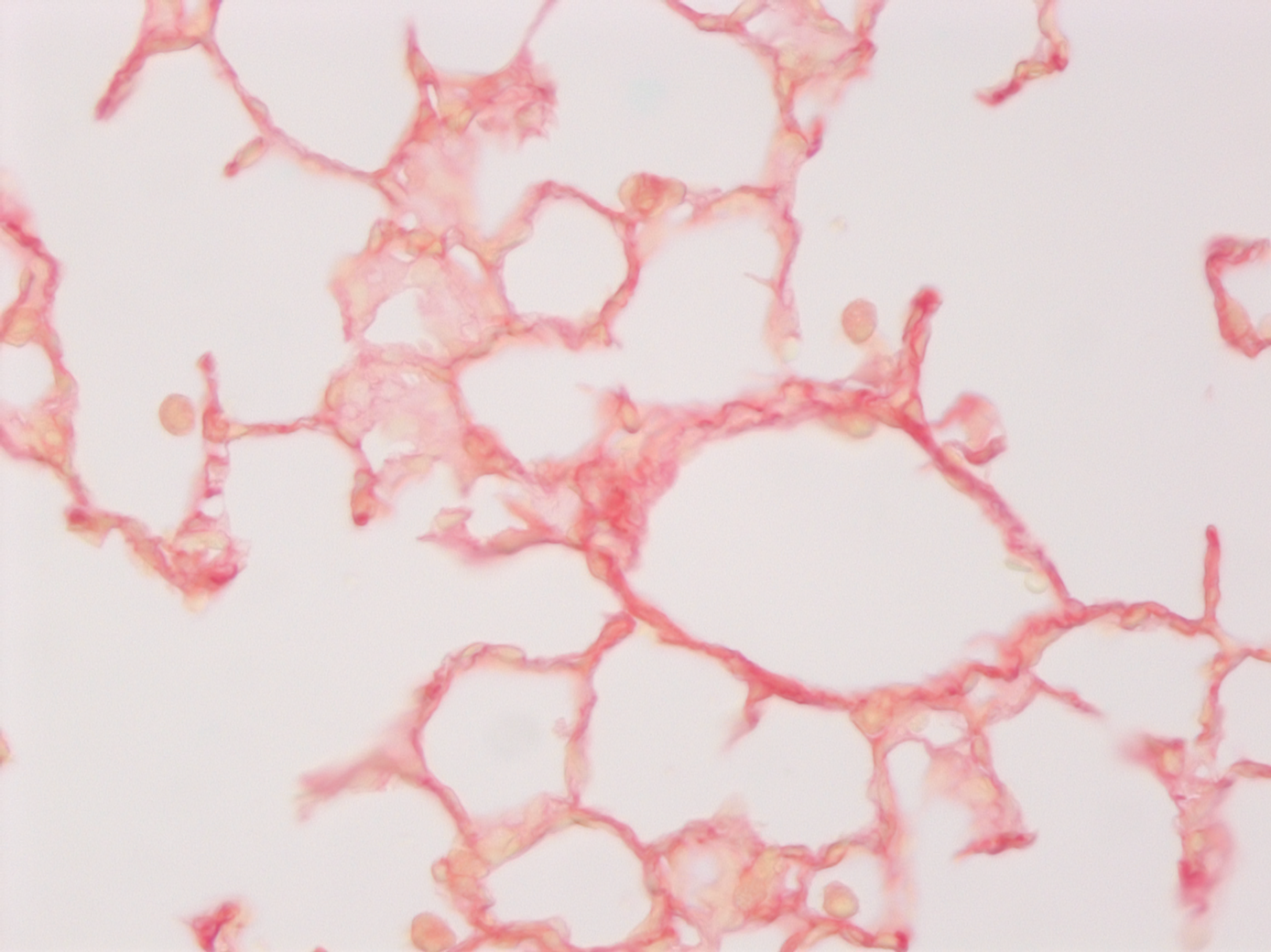

Supplement: Supplementary file 5 — Source Data Fig. 3 [file 44319_2023_41_MOESM5_ESM.zip › Source data Figure 3 /3E Image data, micr. image/Vessels, SR/p21++.tif]

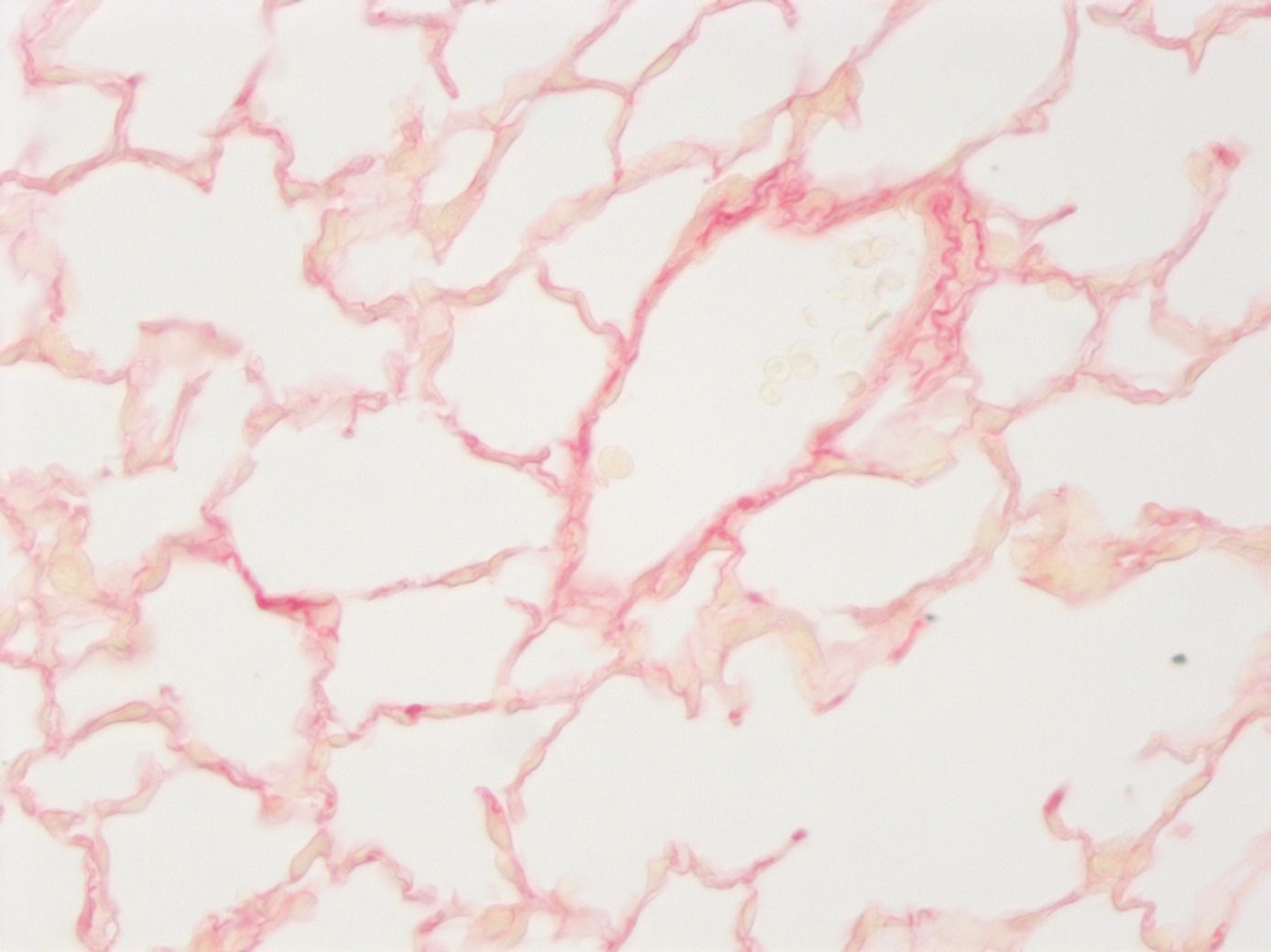

Supplement: Supplementary file 5 — Source Data Fig. 3 [file 44319_2023_41_MOESM5_ESM.zip › Source data Figure 3 /3E Image data, micr. image/Vessels, SR/p21+TERT.tif]

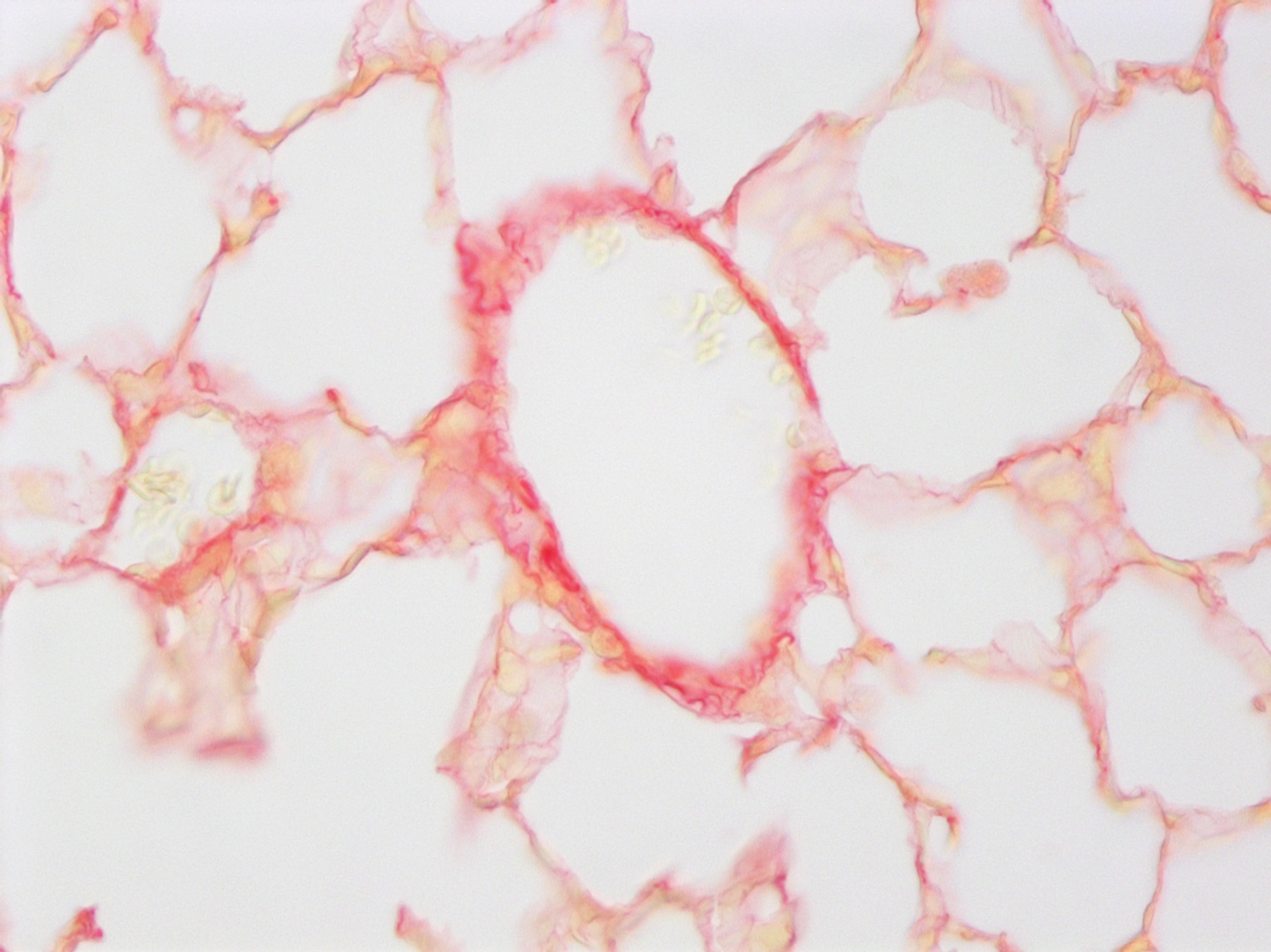

Supplement: Supplementary file 5 — Source Data Fig. 3 [file 44319_2023_41_MOESM5_ESM.zip › Source data Figure 3 /3E Image data, micr. image/Vessels, SR/p21+TERT CI.tif]

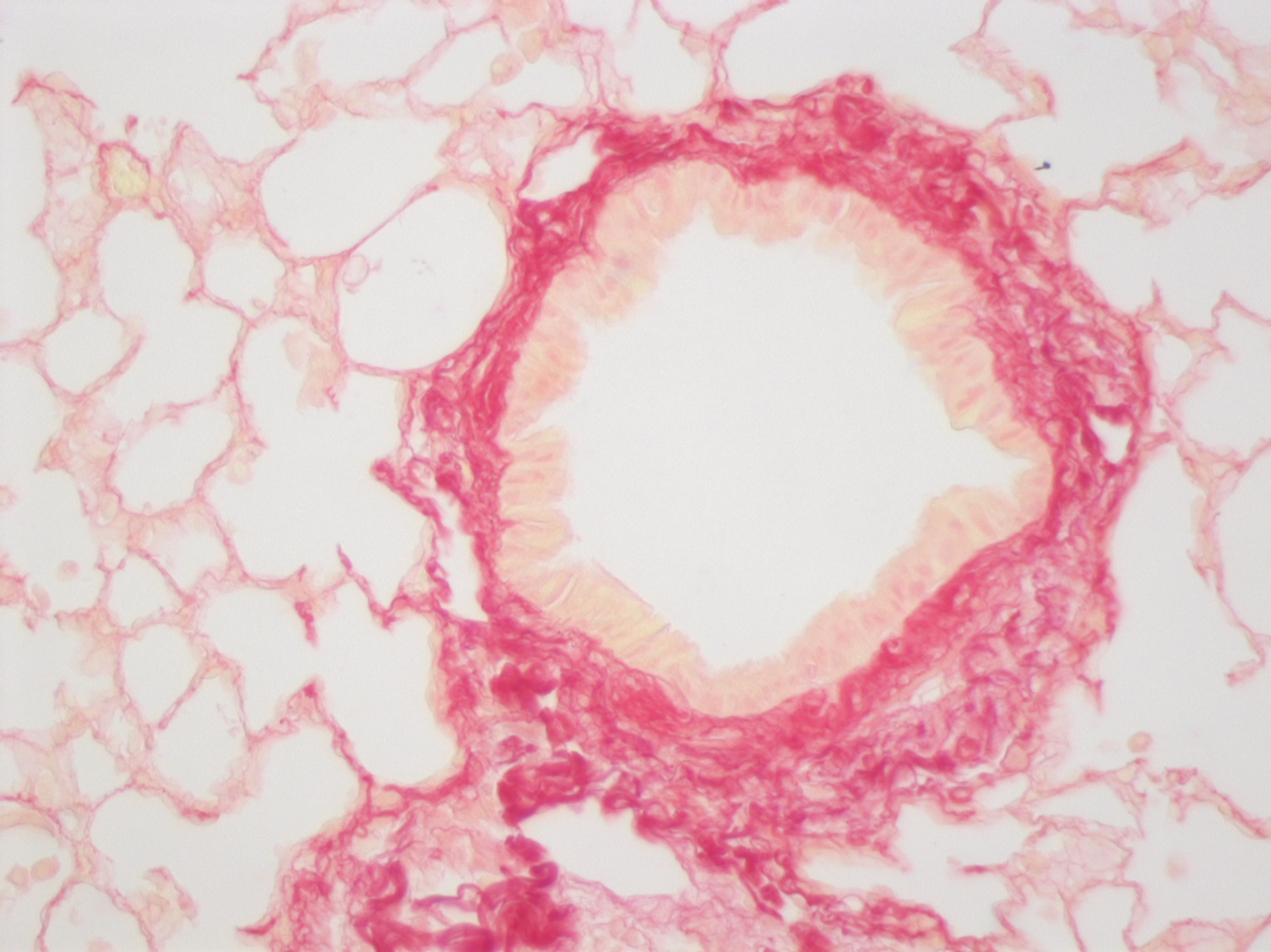

Supplement: Supplementary file 5 — Source Data Fig. 3 [file 44319_2023_41_MOESM5_ESM.zip › Source data Figure 3 /3E Image data, micr. image/Bronchi, SR/p21+-.tif]

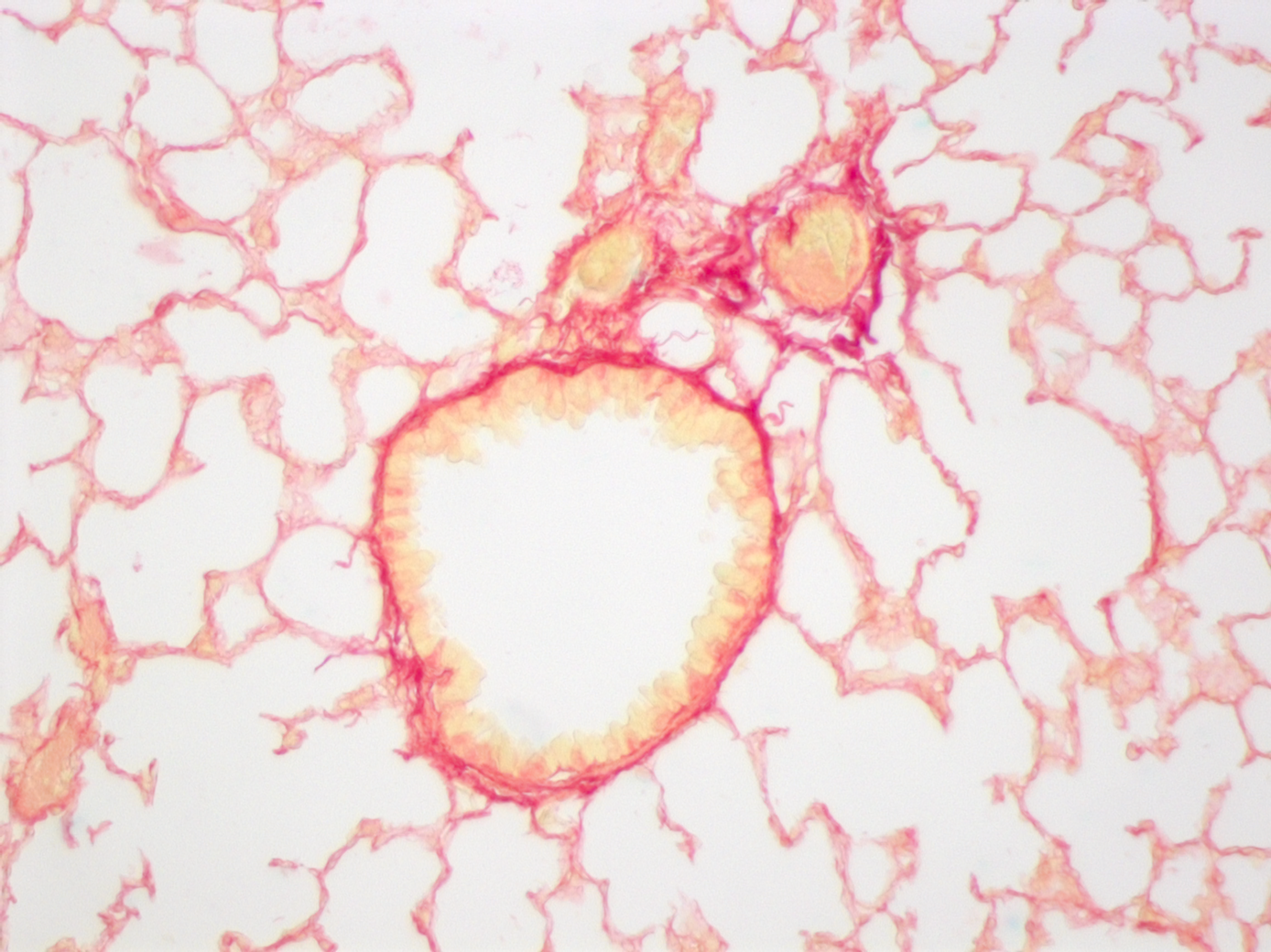

Supplement: Supplementary file 5 — Source Data Fig. 3 [file 44319_2023_41_MOESM5_ESM.zip › Source data Figure 3 /3E Image data, micr. image/Bronchi, SR/p21++.tif]

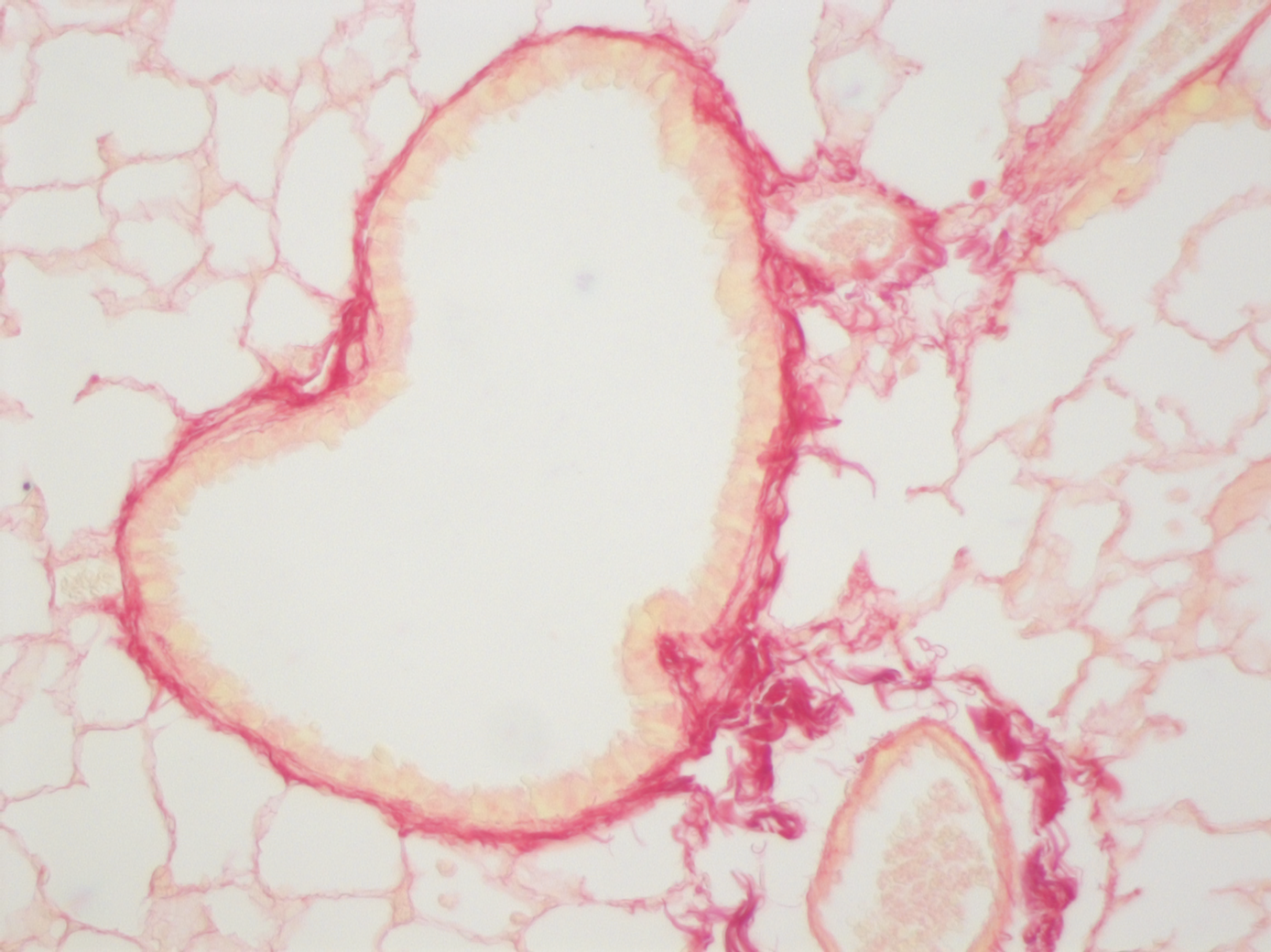

Supplement: Supplementary file 5 — Source Data Fig. 3 [file 44319_2023_41_MOESM5_ESM.zip › Source data Figure 3 /3E Image data, micr. image/Bronchi, SR/p21+TERT.tif]

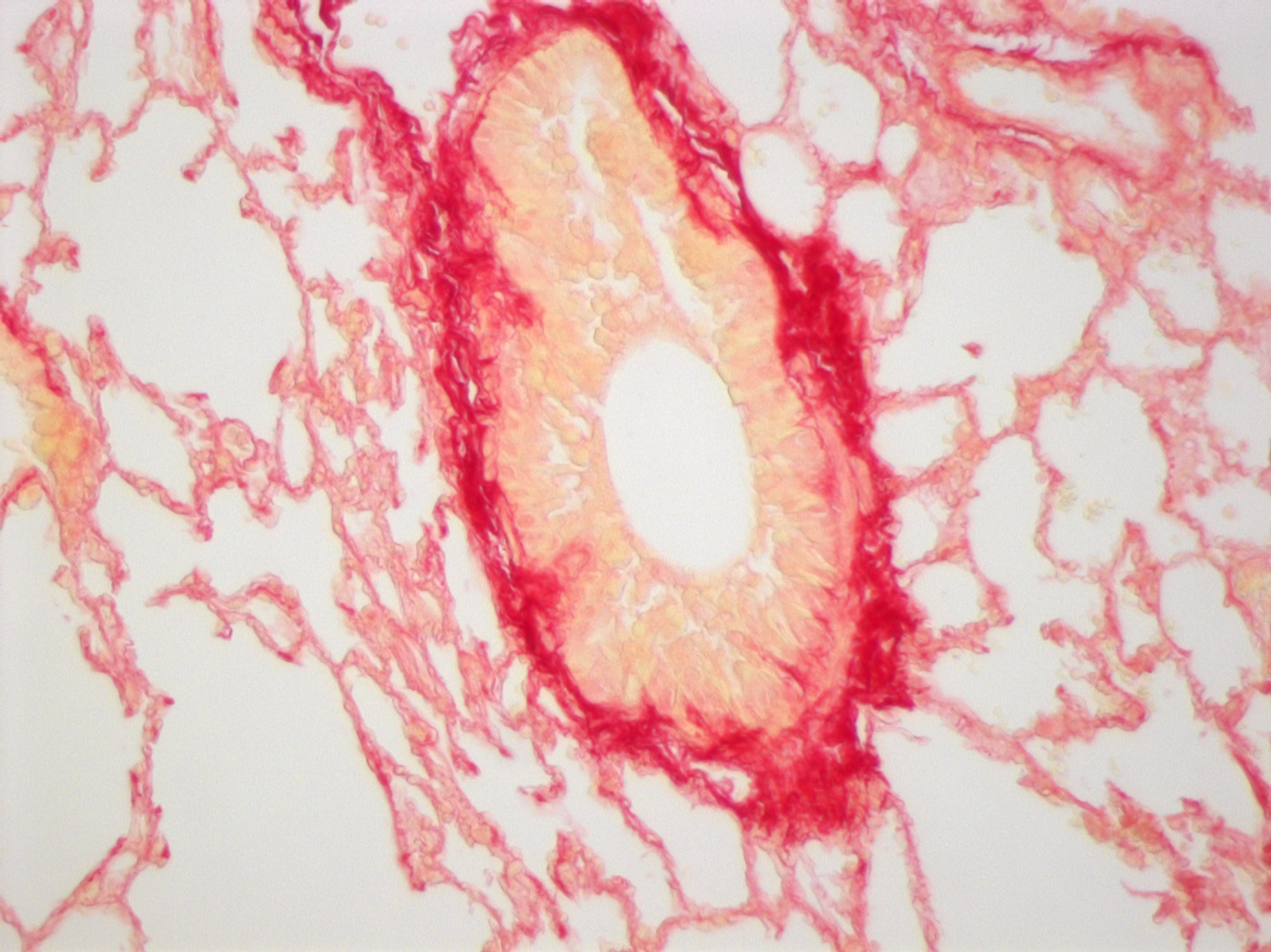

Supplement: Supplementary file 5 — Source Data Fig. 3 [file 44319_2023_41_MOESM5_ESM.zip › Source data Figure 3 /3E Image data, micr. image/Bronchi, SR/p21+TERTCI.tif]

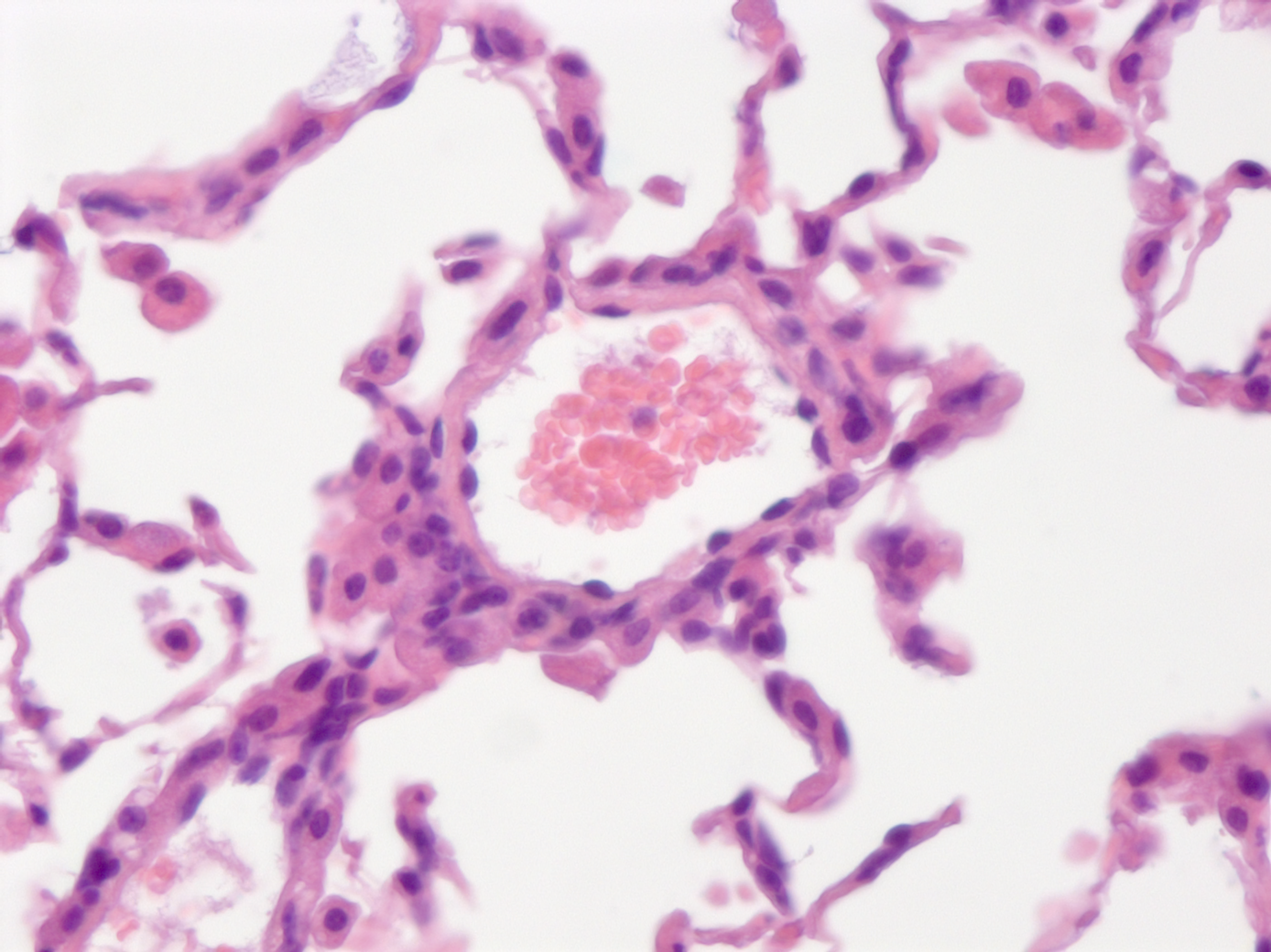

Supplement: Supplementary file 5 — Source Data Fig. 3 [file 44319_2023_41_MOESM5_ESM.zip › Source data Figure 3 /3E Image data, micr. image/Vessels, HE/p21+-.tif]

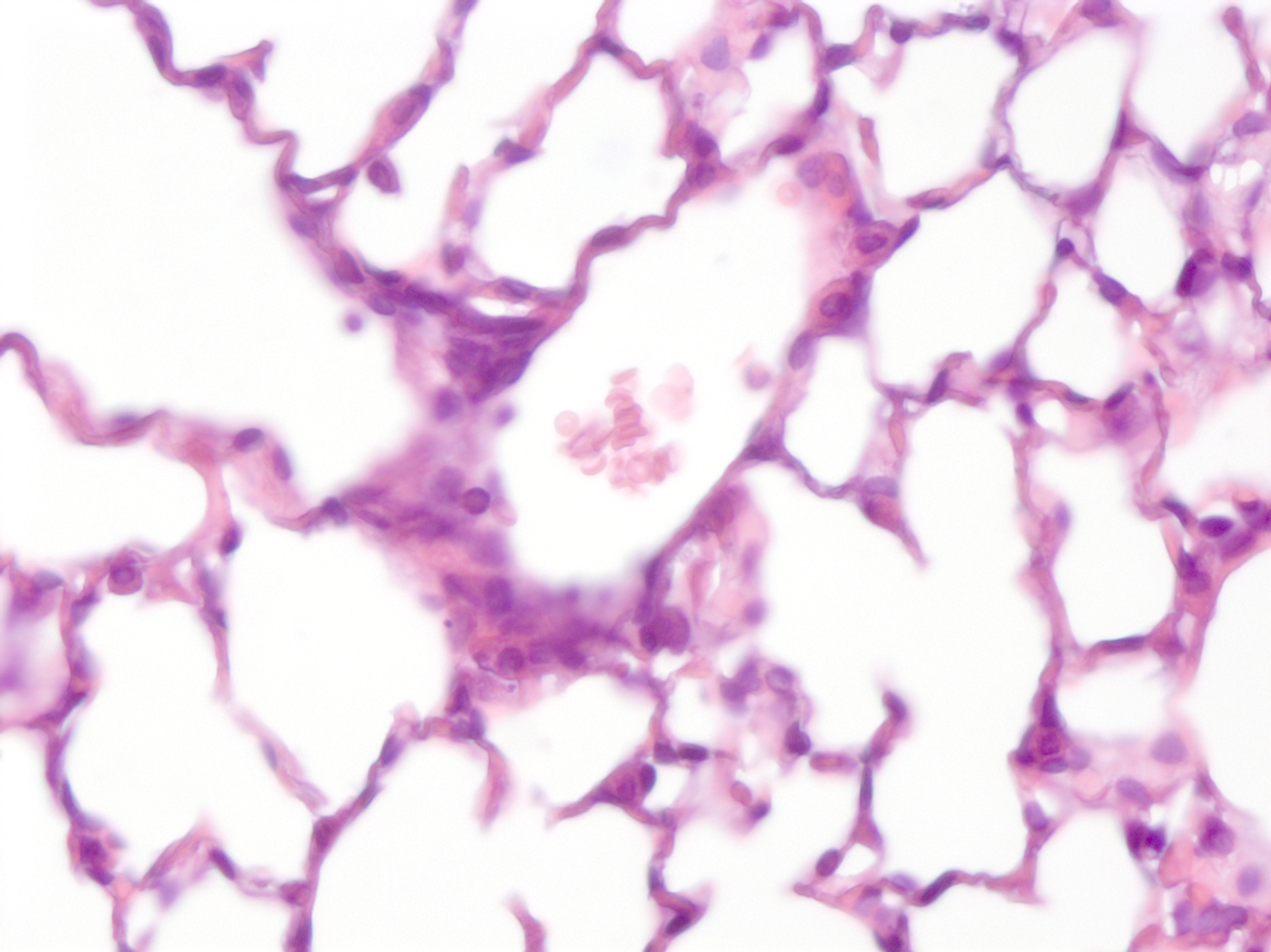

Supplement: Supplementary file 5 — Source Data Fig. 3 [file 44319_2023_41_MOESM5_ESM.zip › Source data Figure 3 /3E Image data, micr. image/Vessels, HE/p21++.tif]

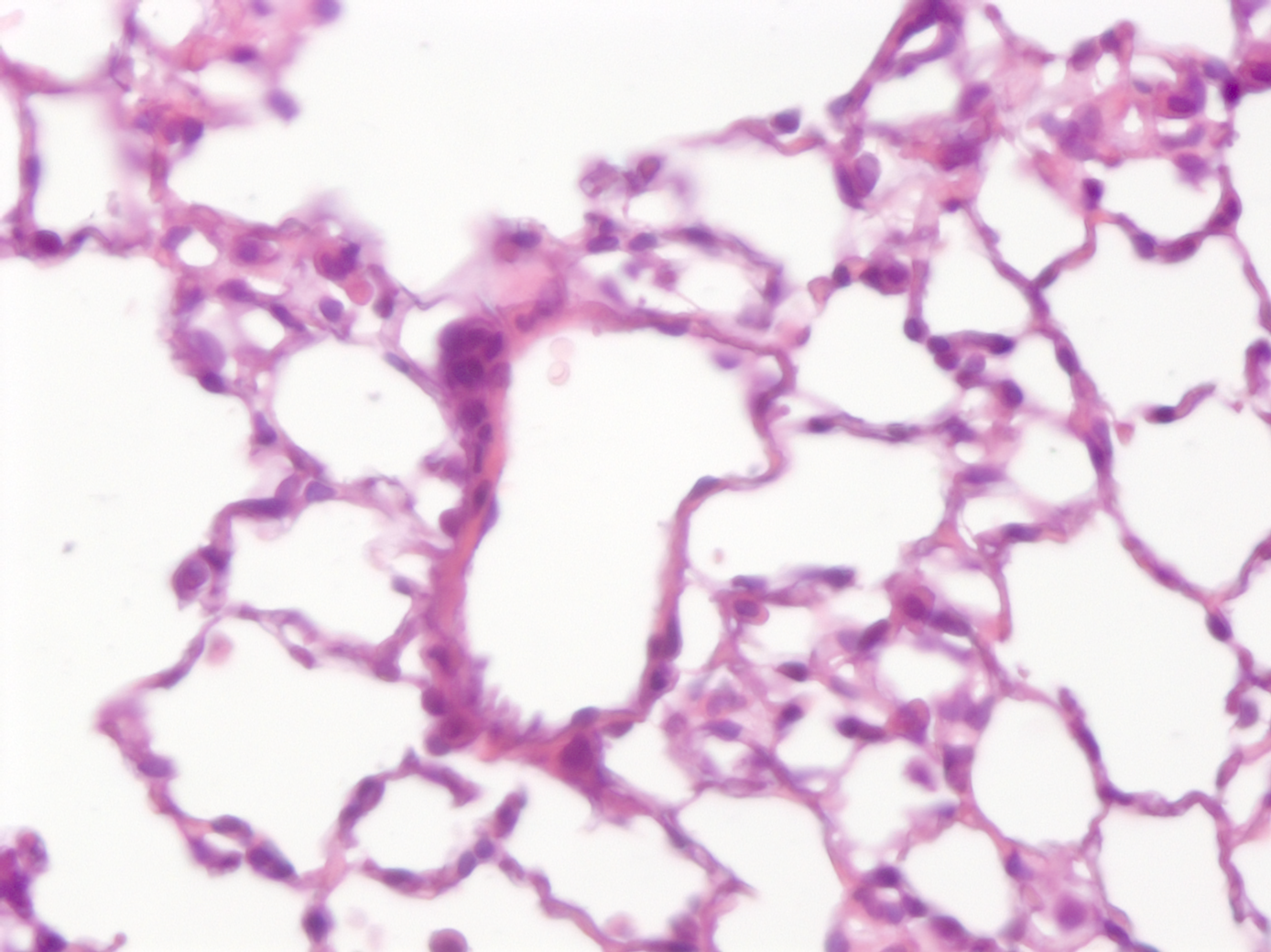

Supplement: Supplementary file 5 — Source Data Fig. 3 [file 44319_2023_41_MOESM5_ESM.zip › Source data Figure 3 /3E Image data, micr. image/Vessels, HE/p21+TERT.tif]

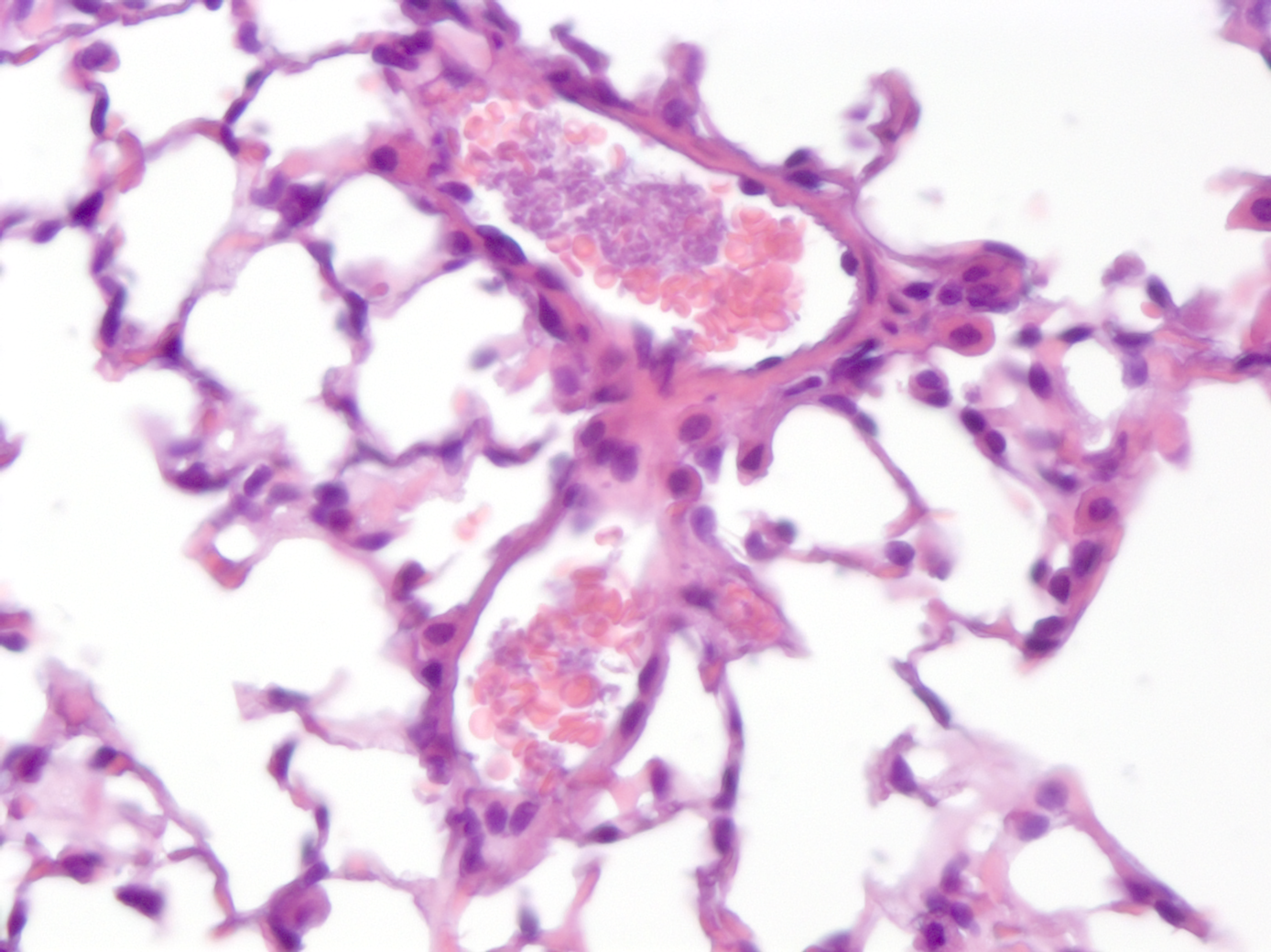

Supplement: Supplementary file 5 — Source Data Fig. 3 [file 44319_2023_41_MOESM5_ESM.zip › Source data Figure 3 /3E Image data, micr. image/Vessels, HE/p21+TERT CI.tif]

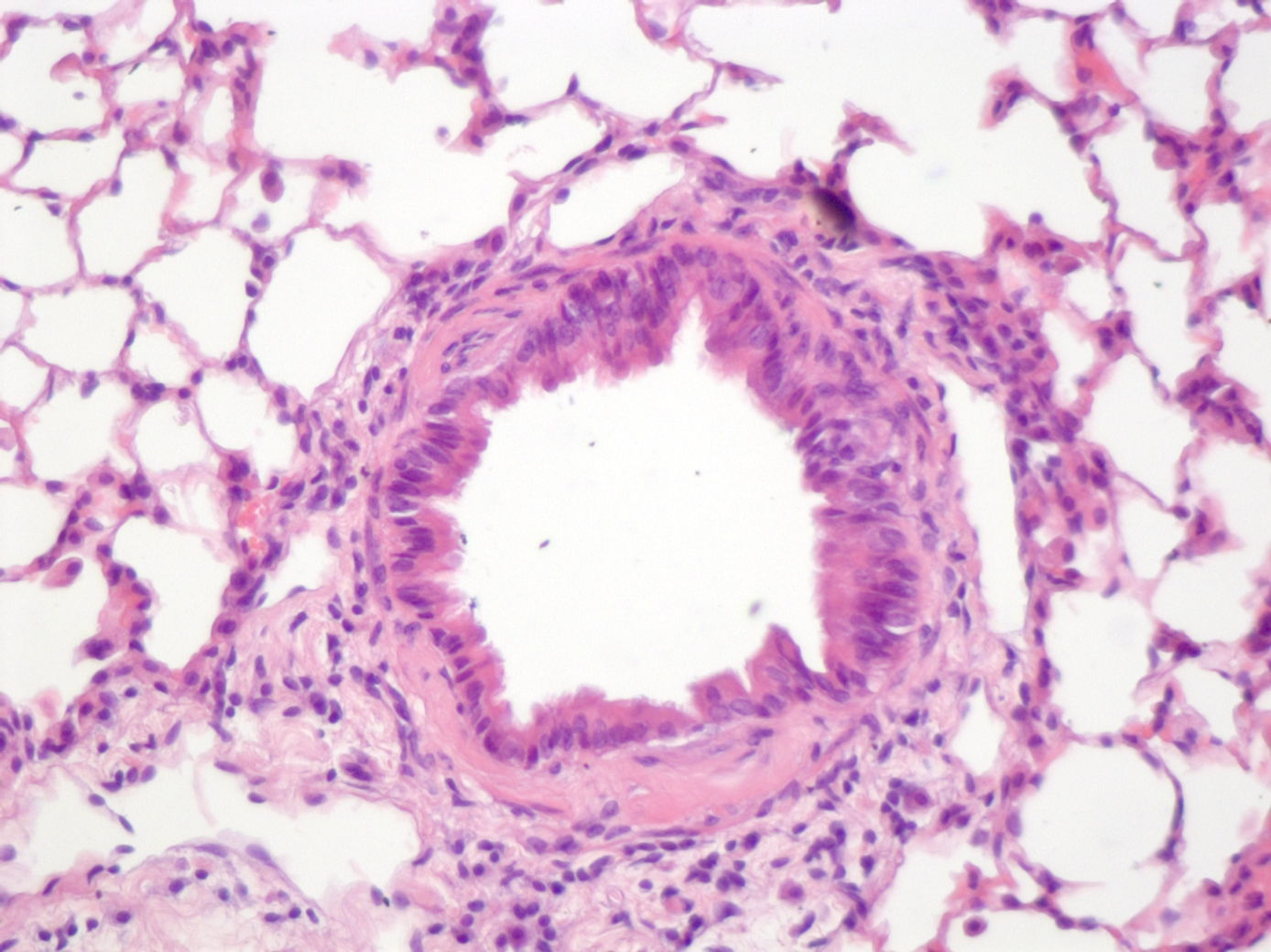

Supplement: Supplementary file 5 — Source Data Fig. 3 [file 44319_2023_41_MOESM5_ESM.zip › Source data Figure 3 /3E Image data, micr. image/Bronchi, HE/p21+-.tif]

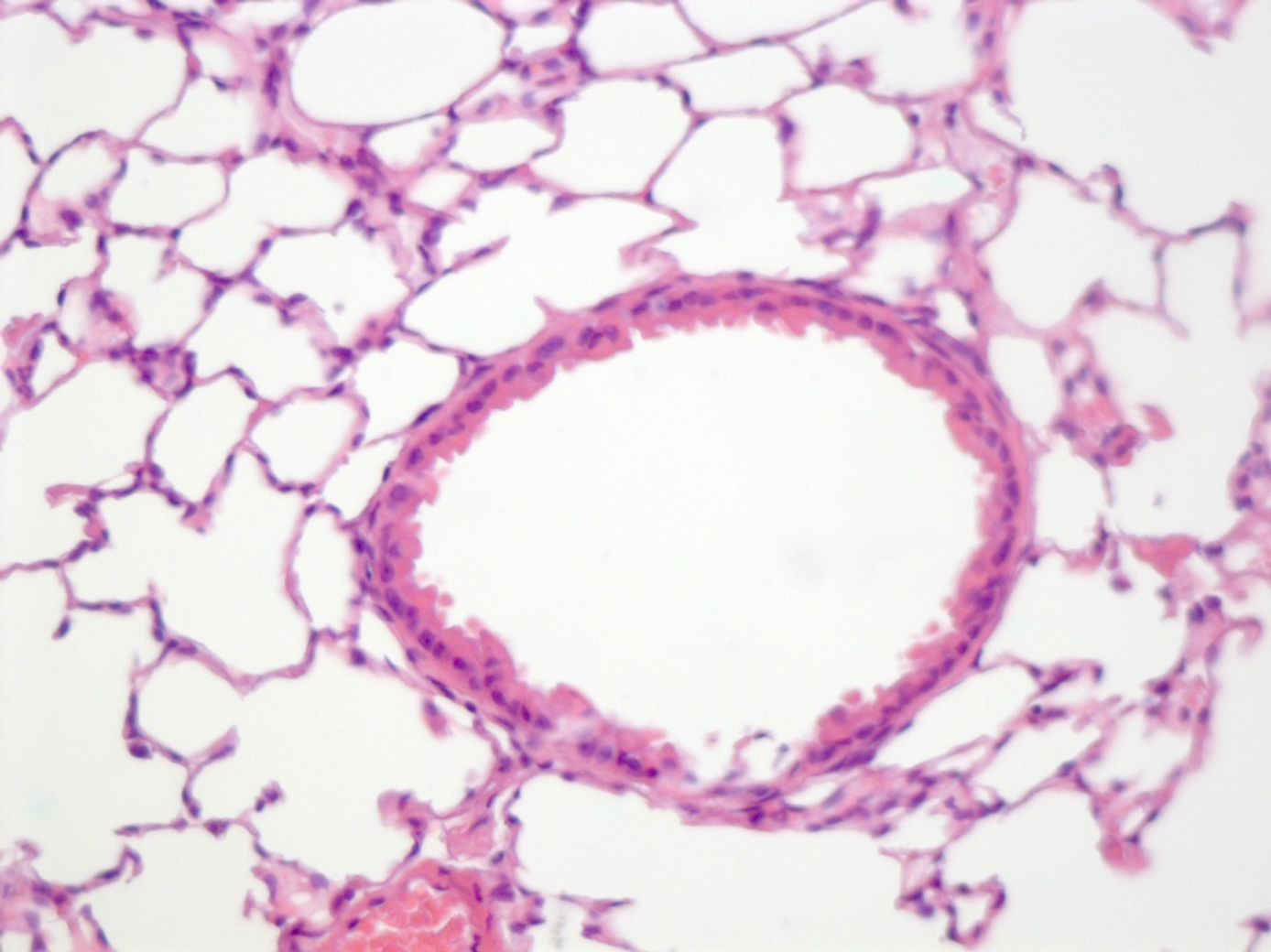

Supplement: Supplementary file 5 — Source Data Fig. 3 [file 44319_2023_41_MOESM5_ESM.zip › Source data Figure 3 /3E Image data, micr. image/Bronchi, HE/p21++.tif]

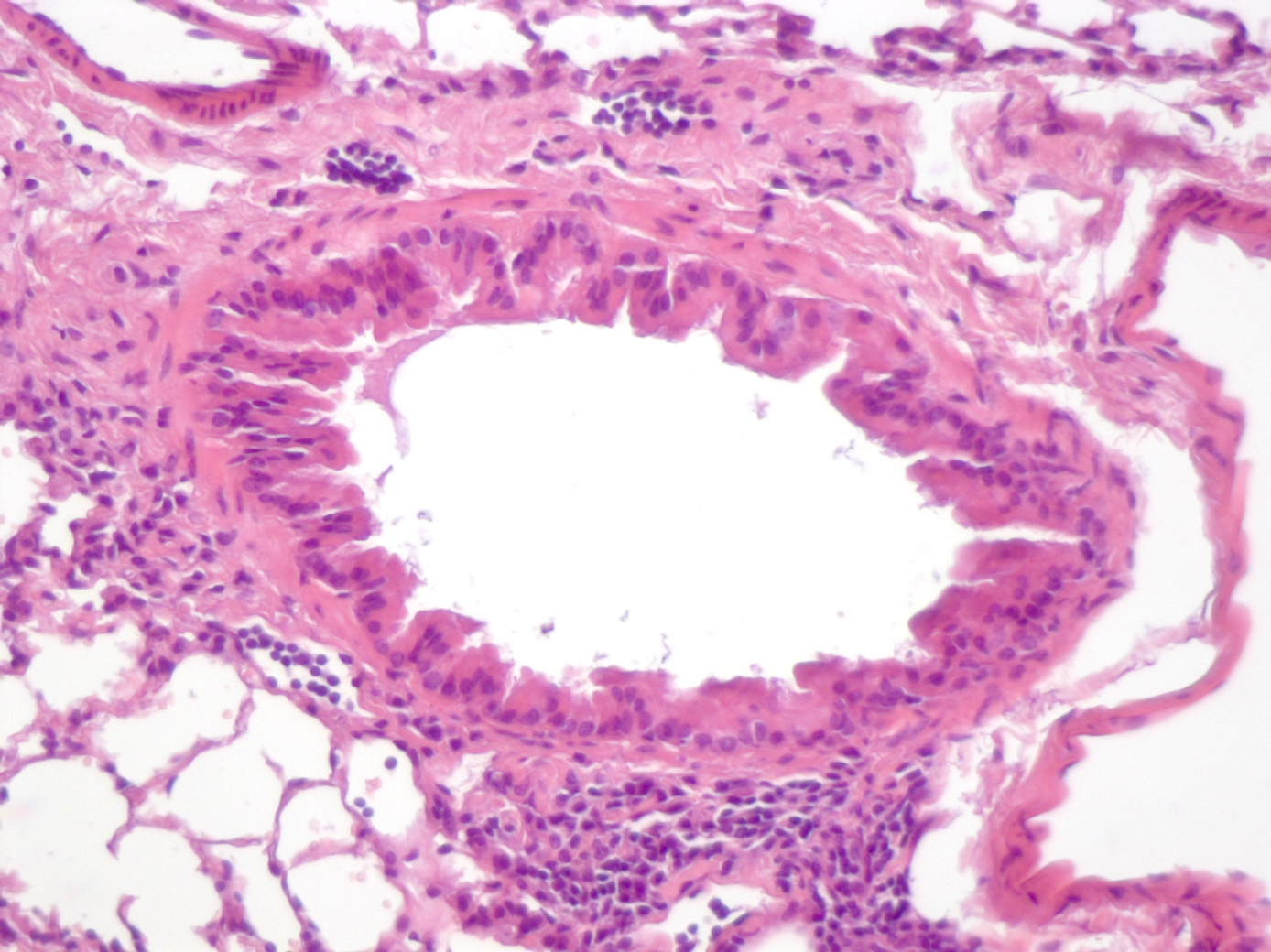

Supplement: Supplementary file 5 — Source Data Fig. 3 [file 44319_2023_41_MOESM5_ESM.zip › Source data Figure 3 /3E Image data, micr. image/Bronchi, HE/p21TERT CI.tif]

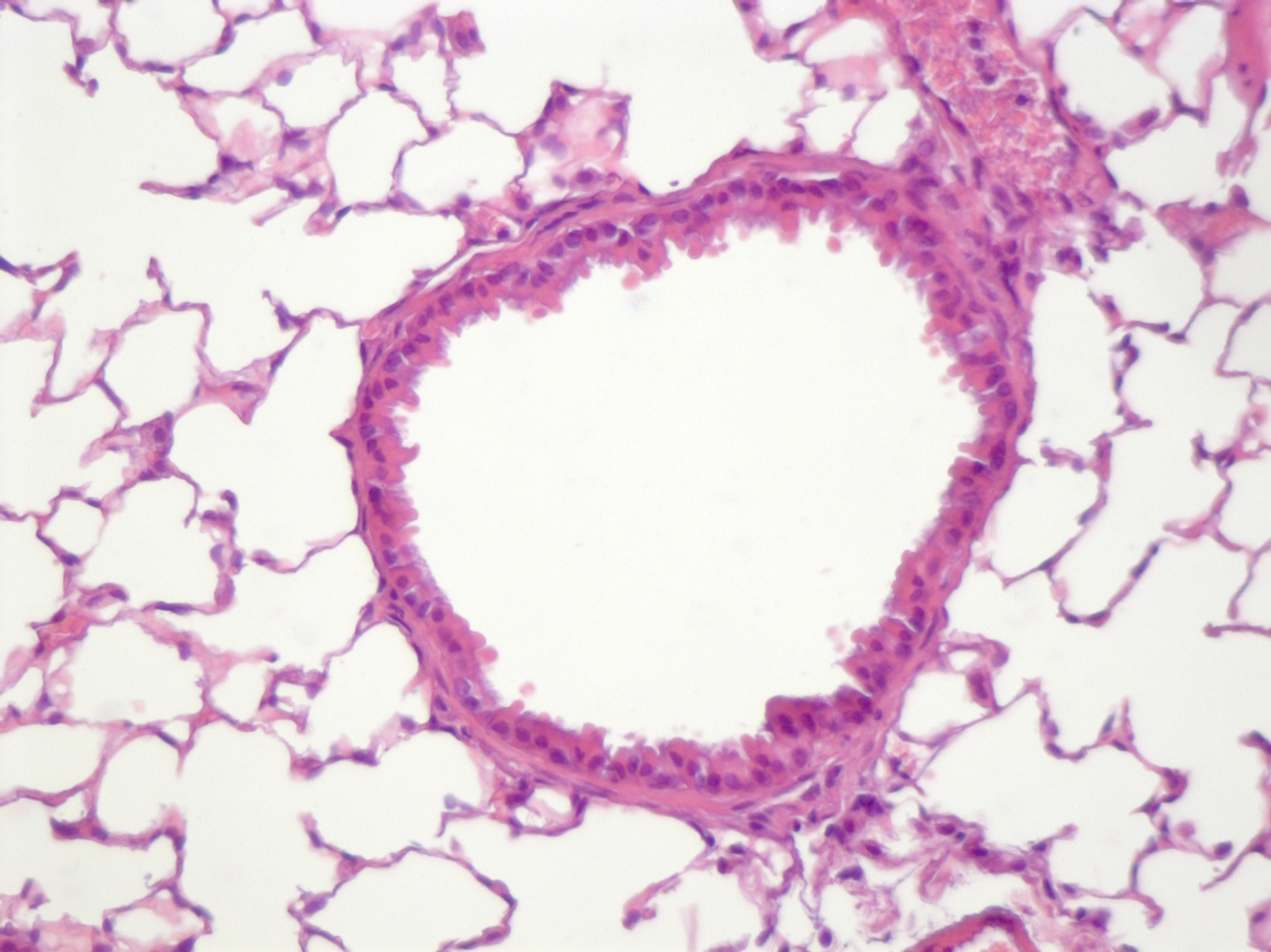

Supplement: Supplementary file 5 — Source Data Fig. 3 [file 44319_2023_41_MOESM5_ESM.zip › Source data Figure 3 /3E Image data, micr. image/Bronchi, HE/p21+TERT.tif]

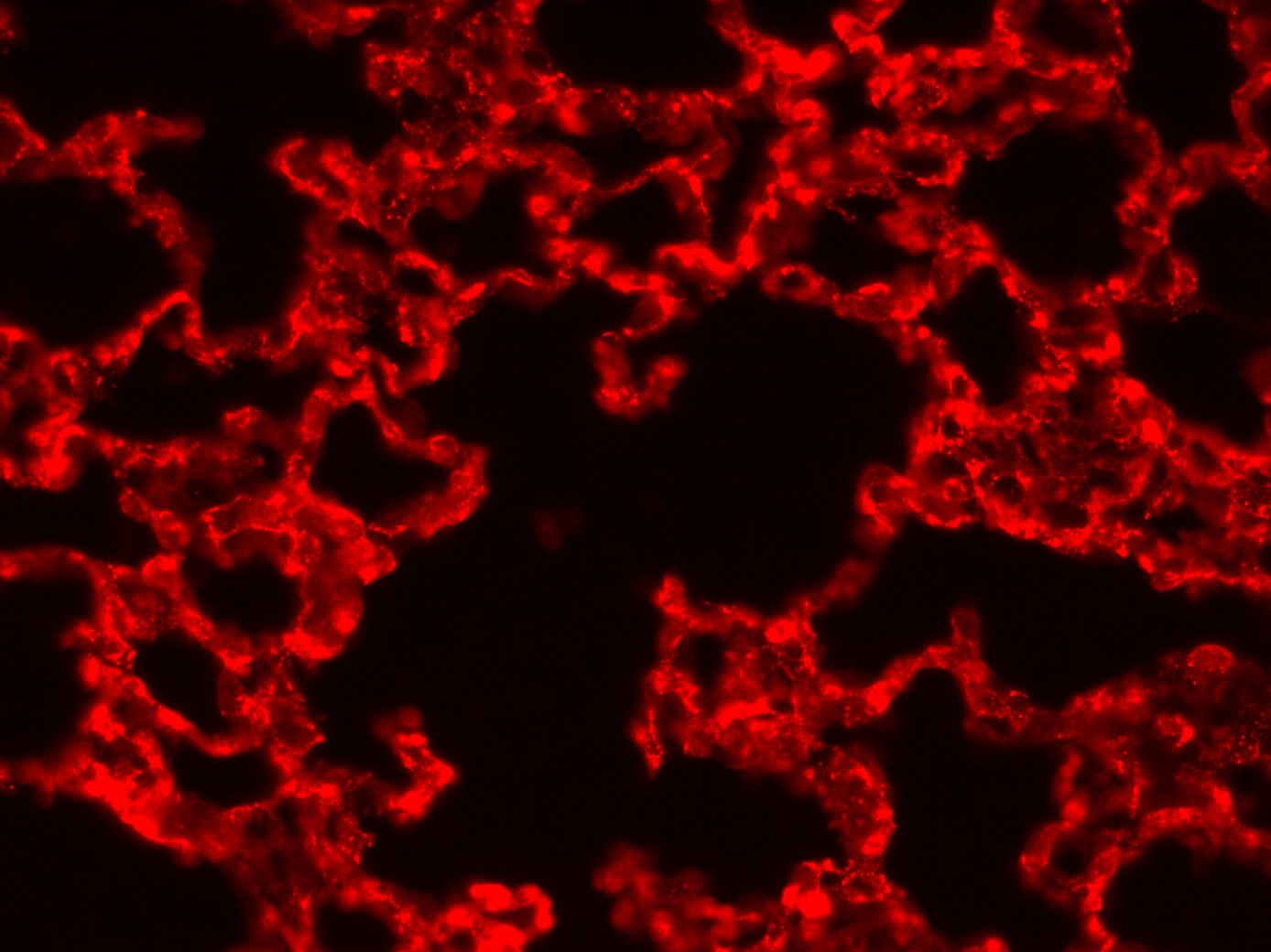

Supplement: Supplementary file 7 — Source Data Fig. 5 [file 44319_2023_41_MOESM7_ESM.zip › Source data Figure 5/5C Image data Micr image/p21++ CD31.tif]

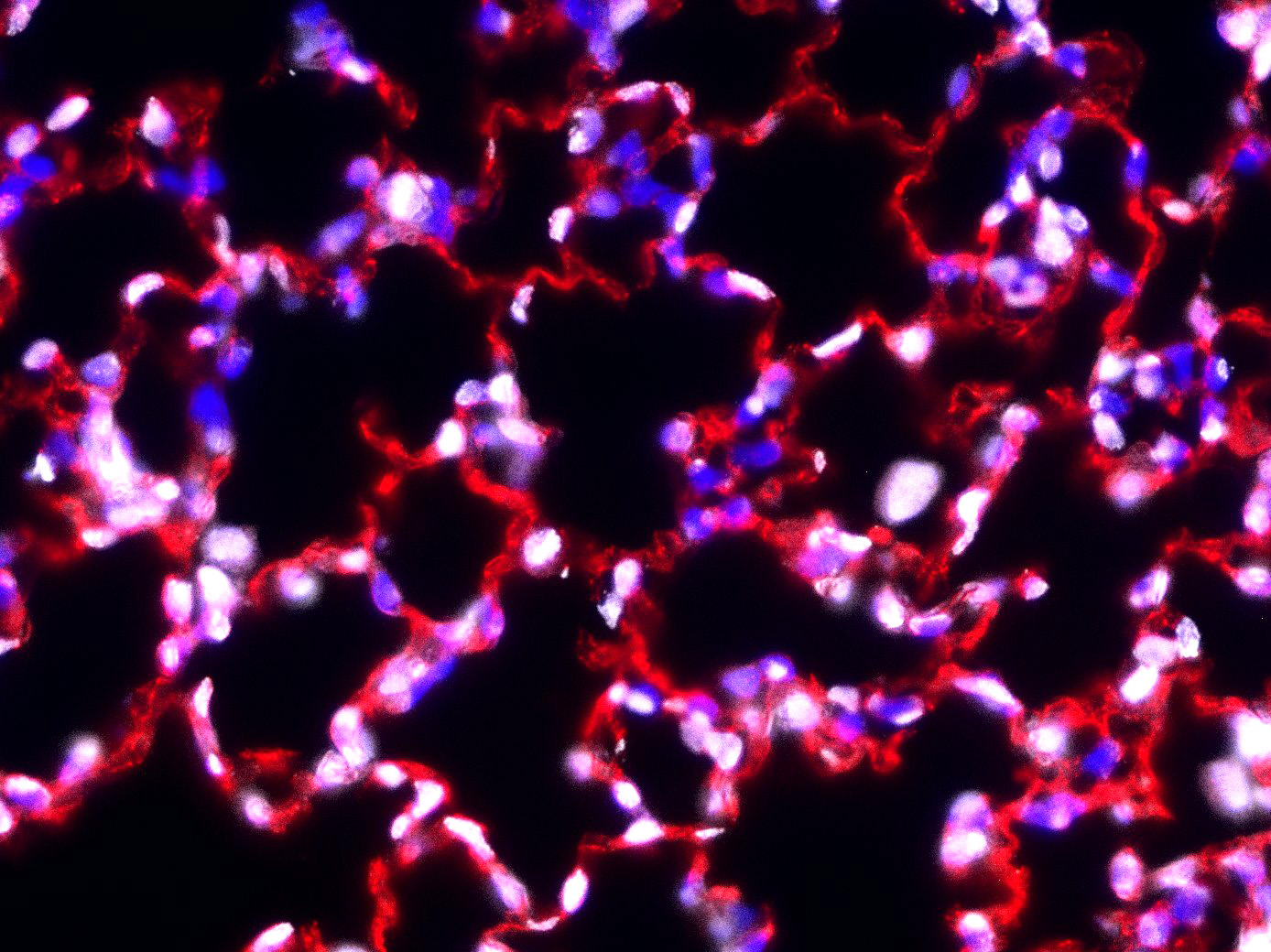

Supplement: Supplementary file 7 — Source Data Fig. 5 [file 44319_2023_41_MOESM7_ESM.zip › Source data Figure 5/5C Image data Micr image/p21+TERT CI MERGE.tif]

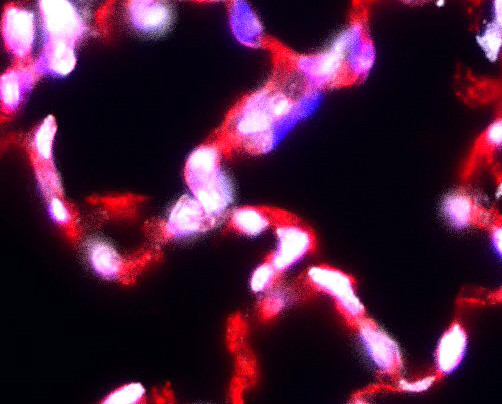

Supplement: Supplementary file 7 — Source Data Fig. 5 [file 44319_2023_41_MOESM7_ESM.zip › Source data Figure 5/5C Image data Micr image/p21+TERT CI ZOOM.tif]

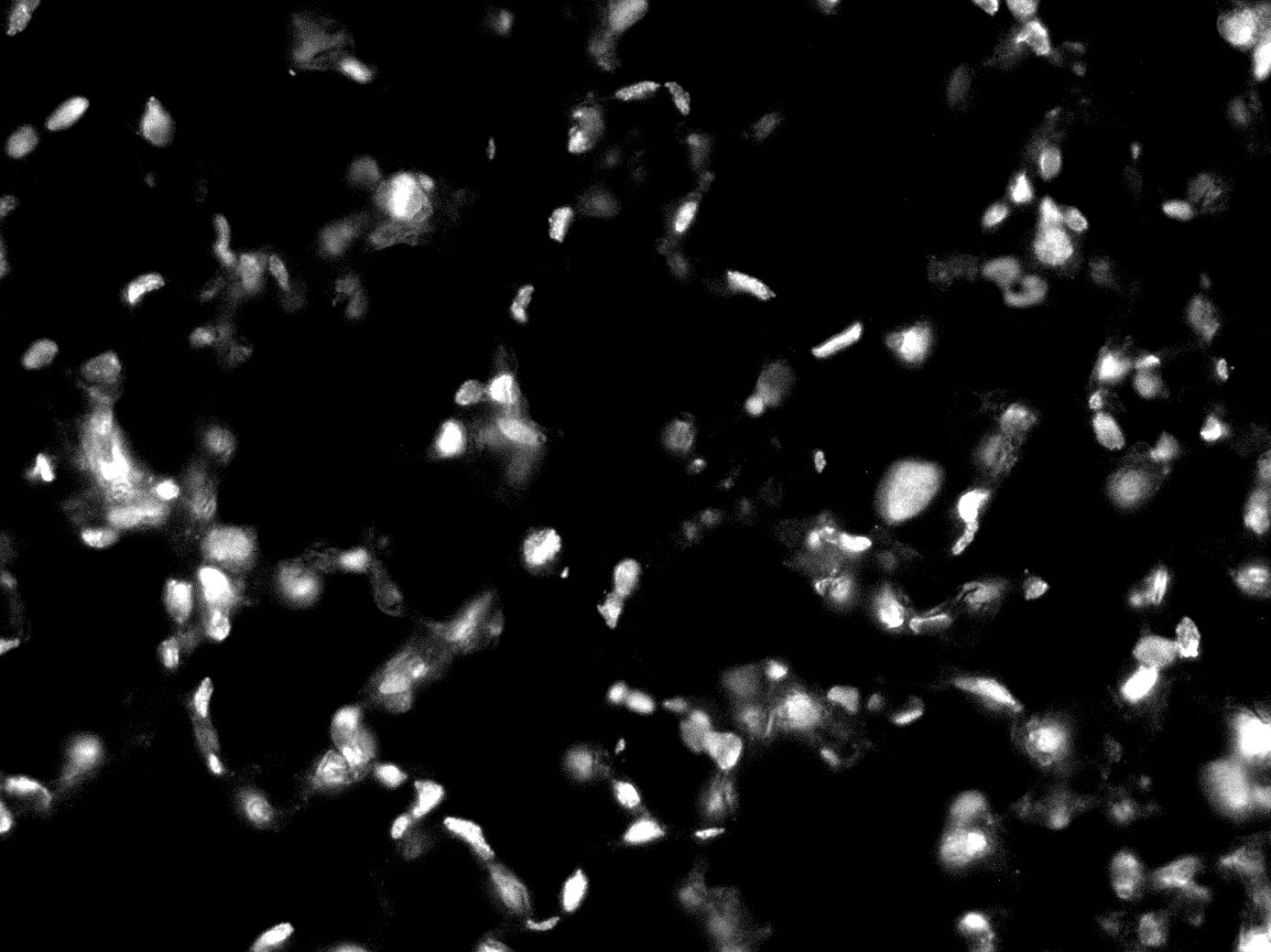

Supplement: Supplementary file 7 — Source Data Fig. 5 [file 44319_2023_41_MOESM7_ESM.zip › Source data Figure 5/5C Image data Micr image/p21+TERT CI p16.tif]

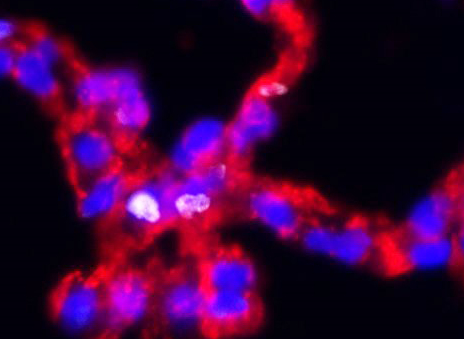

Supplement: Supplementary file 7 — Source Data Fig. 5 [file 44319_2023_41_MOESM7_ESM.zip › Source data Figure 5/5C Image data Micr image/p21+TERT ZOOM.tif]

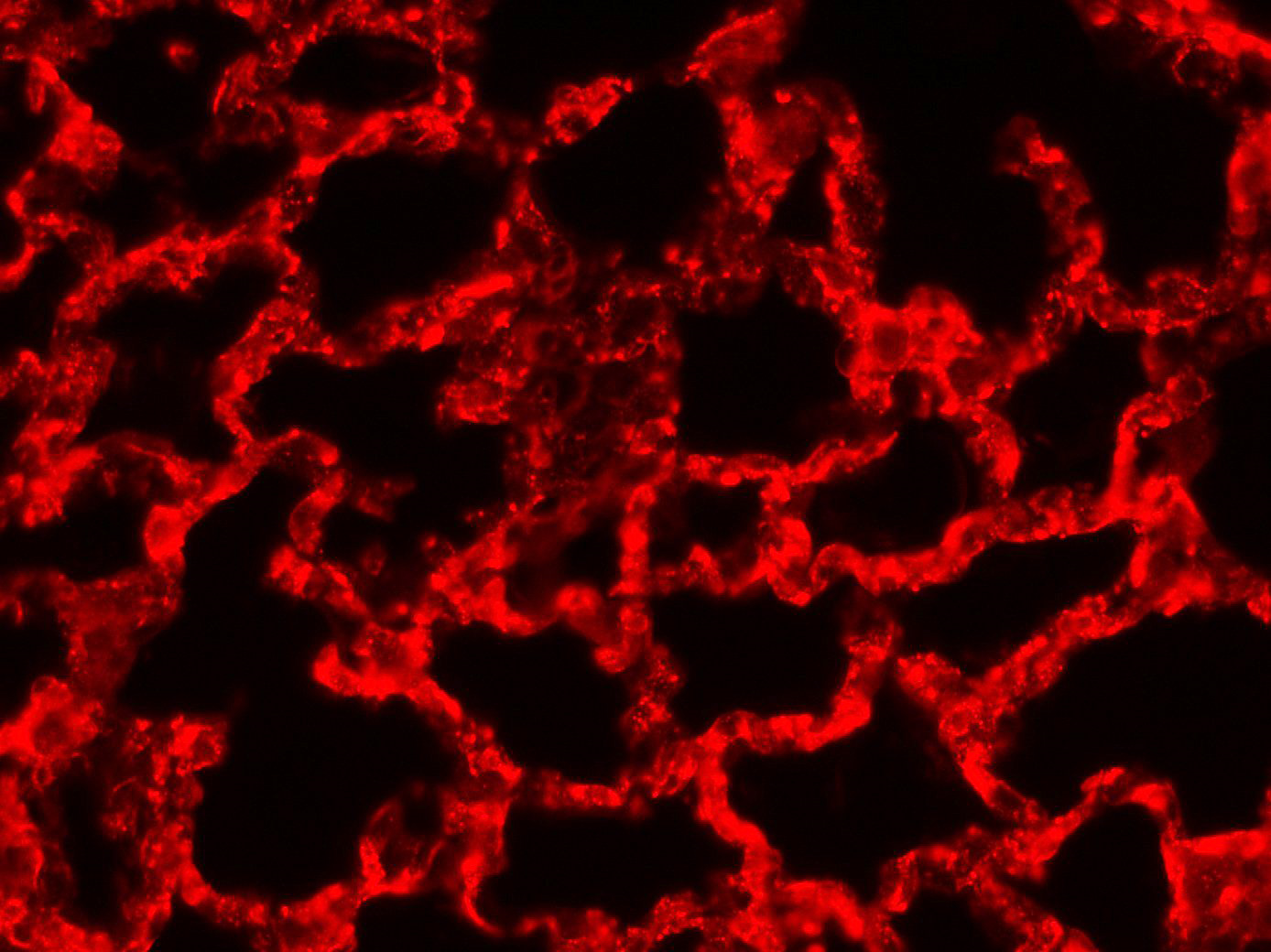

Supplement: Supplementary file 7 — Source Data Fig. 5 [file 44319_2023_41_MOESM7_ESM.zip › Source data Figure 5/5C Image data Micr image/p21+- CD31.tif]

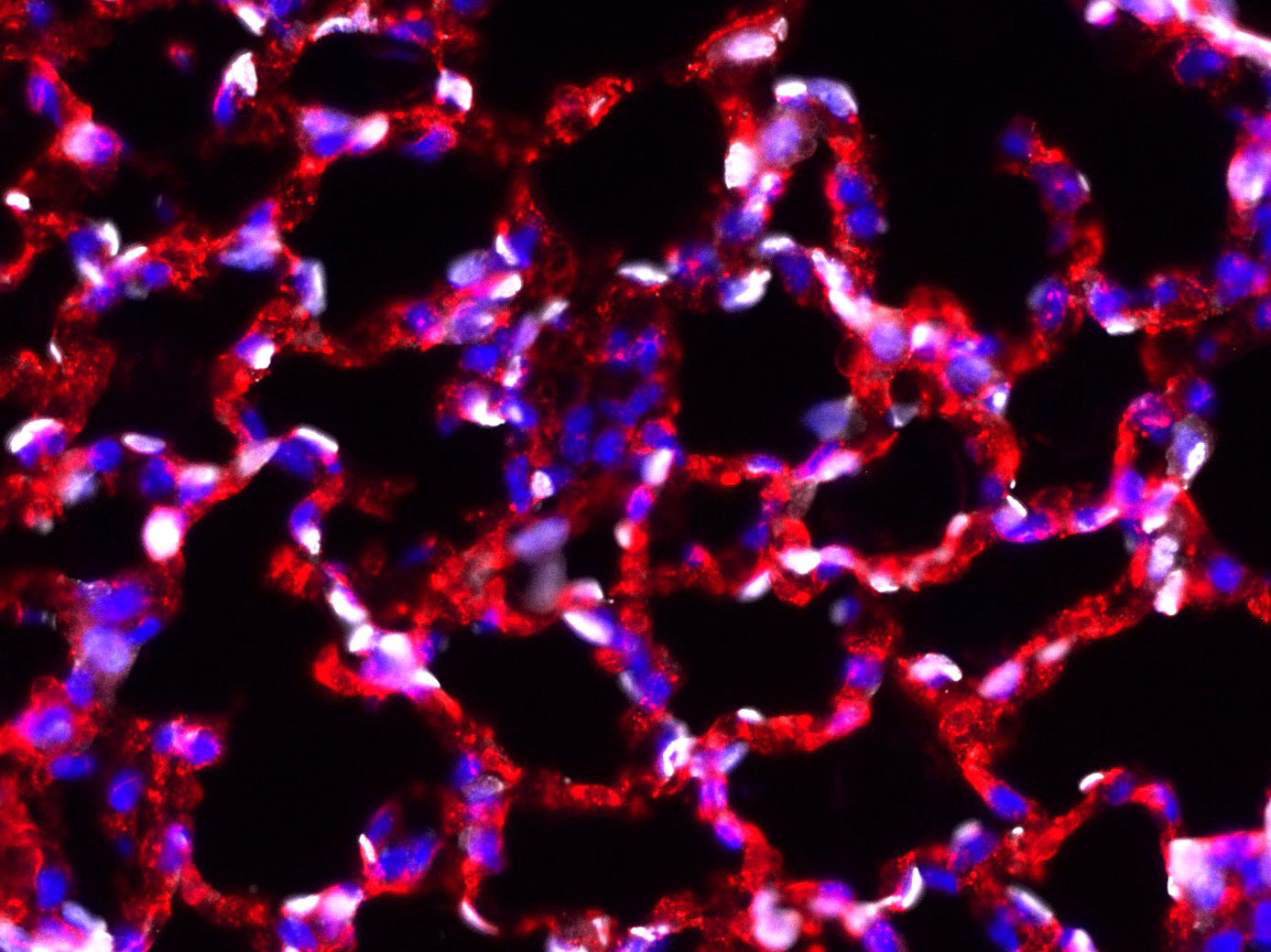

Supplement: Supplementary file 7 — Source Data Fig. 5 [file 44319_2023_41_MOESM7_ESM.zip › Source data Figure 5/5C Image data Micr image/p21-+ MERGE.tif]

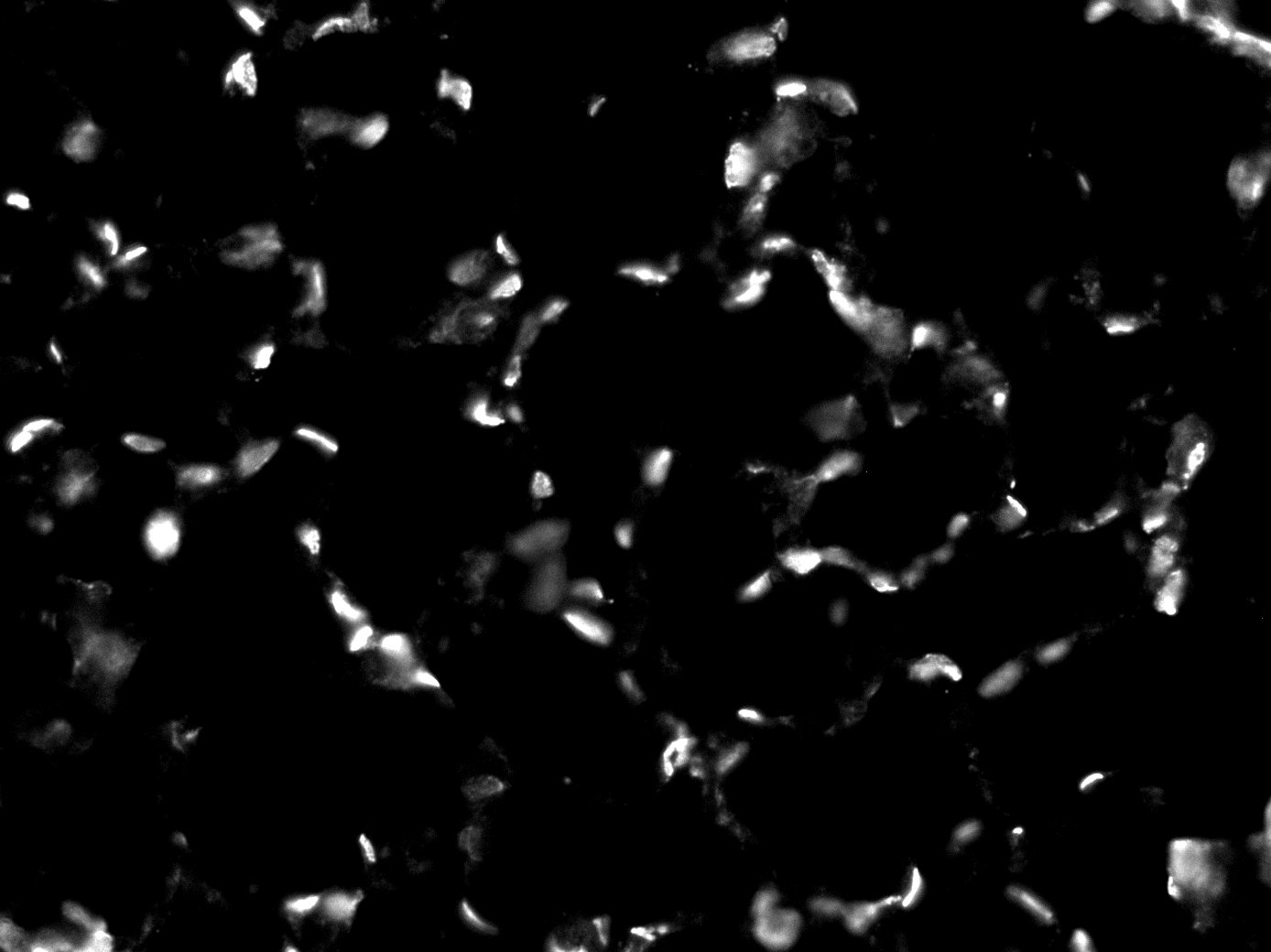

Supplement: Supplementary file 7 — Source Data Fig. 5 [file 44319_2023_41_MOESM7_ESM.zip › Source data Figure 5/5C Image data Micr image/p21+- p16.tif]

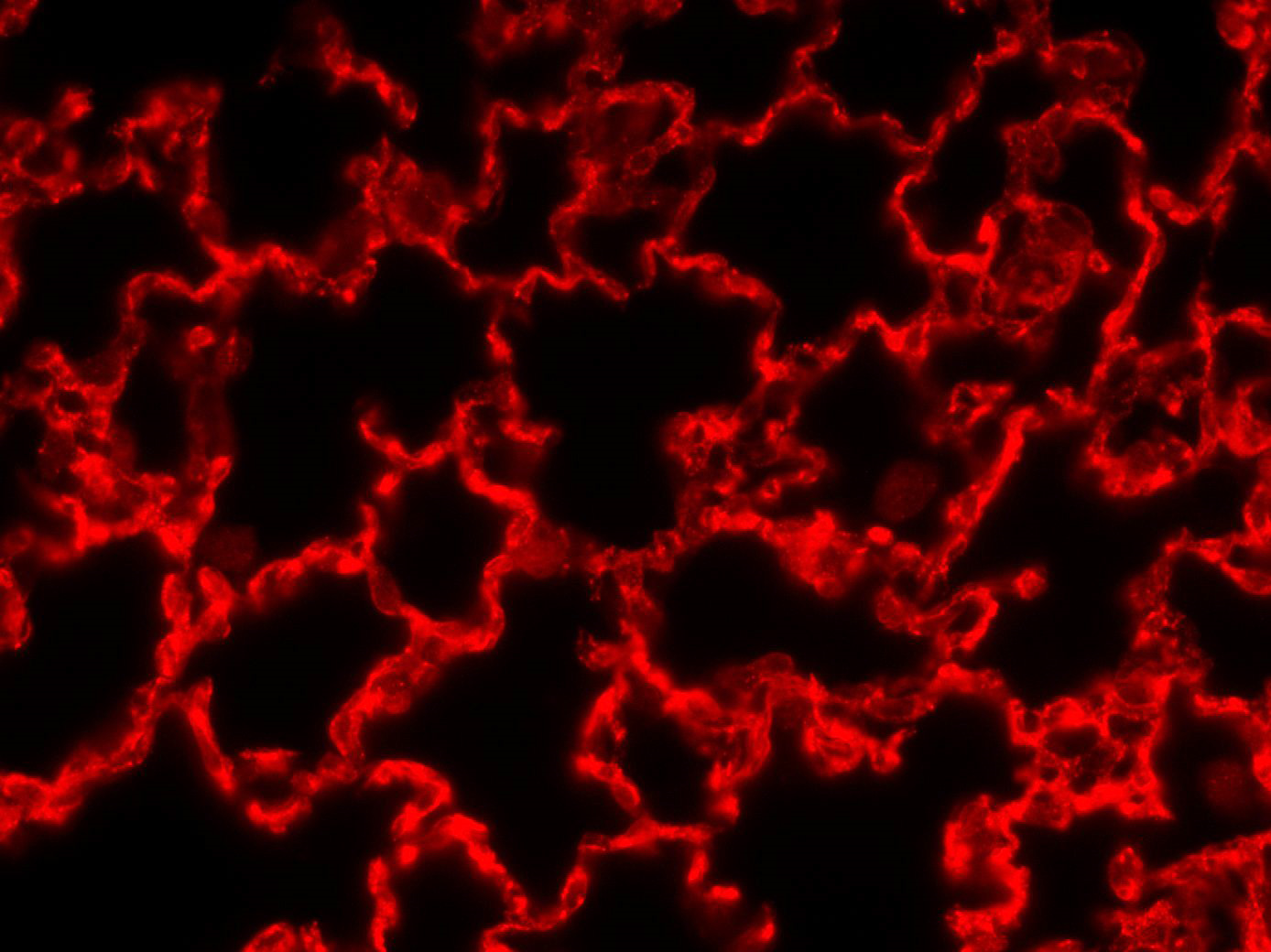

Supplement: Supplementary file 7 — Source Data Fig. 5 [file 44319_2023_41_MOESM7_ESM.zip › Source data Figure 5/5C Image data Micr image/p21+TERT CI CD31.tif]

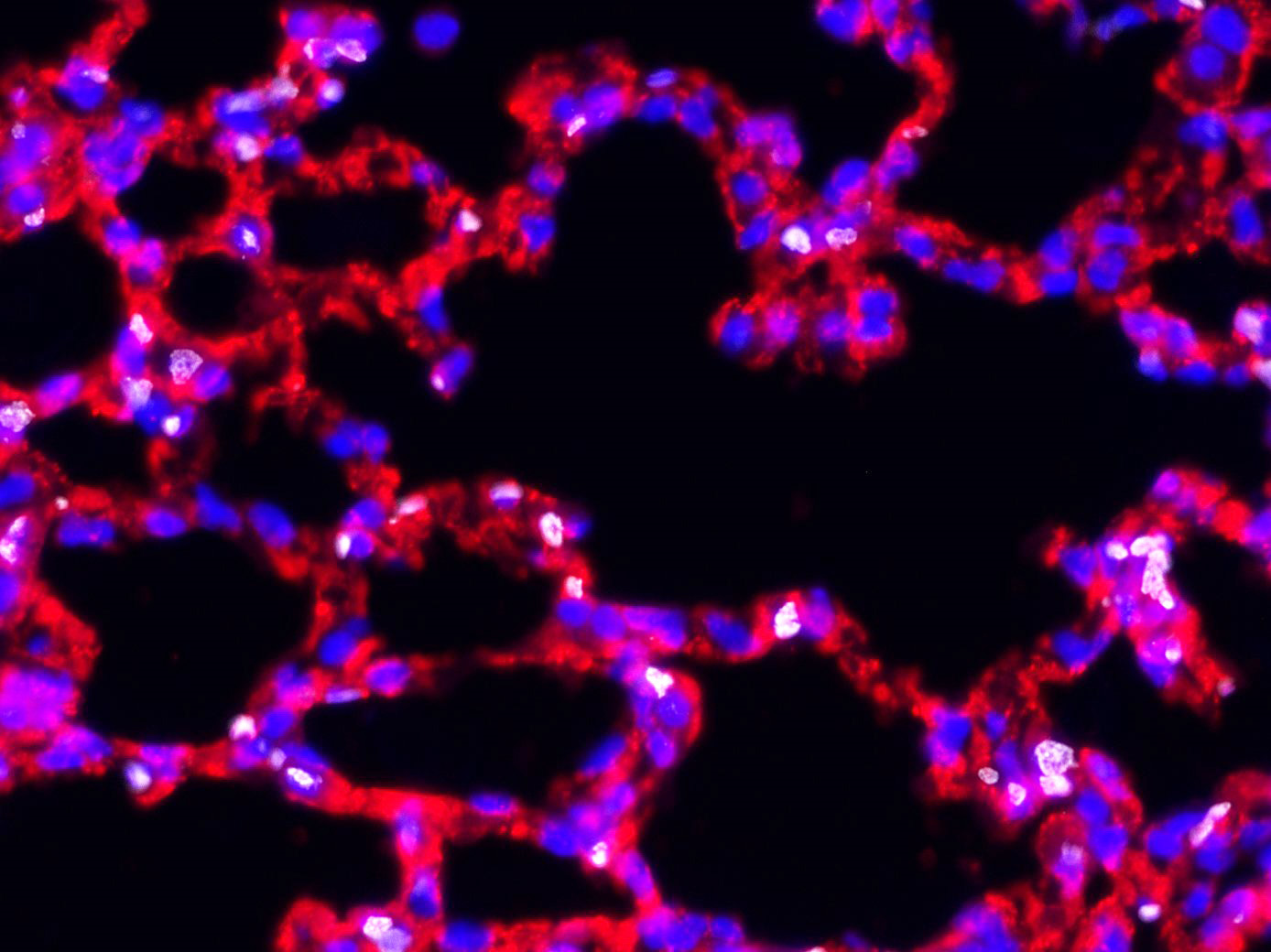

Supplement: Supplementary file 7 — Source Data Fig. 5 [file 44319_2023_41_MOESM7_ESM.zip › Source data Figure 5/5C Image data Micr image/p21+TERT MERGE.tif]

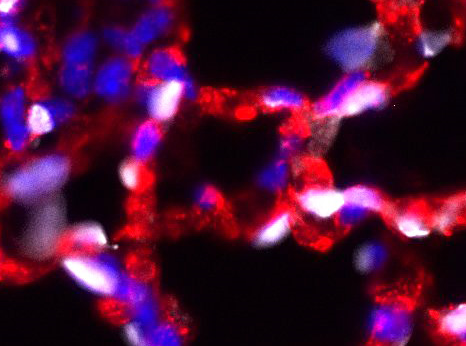

Supplement: Supplementary file 7 — Source Data Fig. 5 [file 44319_2023_41_MOESM7_ESM.zip › Source data Figure 5/5C Image data Micr image/p21-+ZOOM.tif]

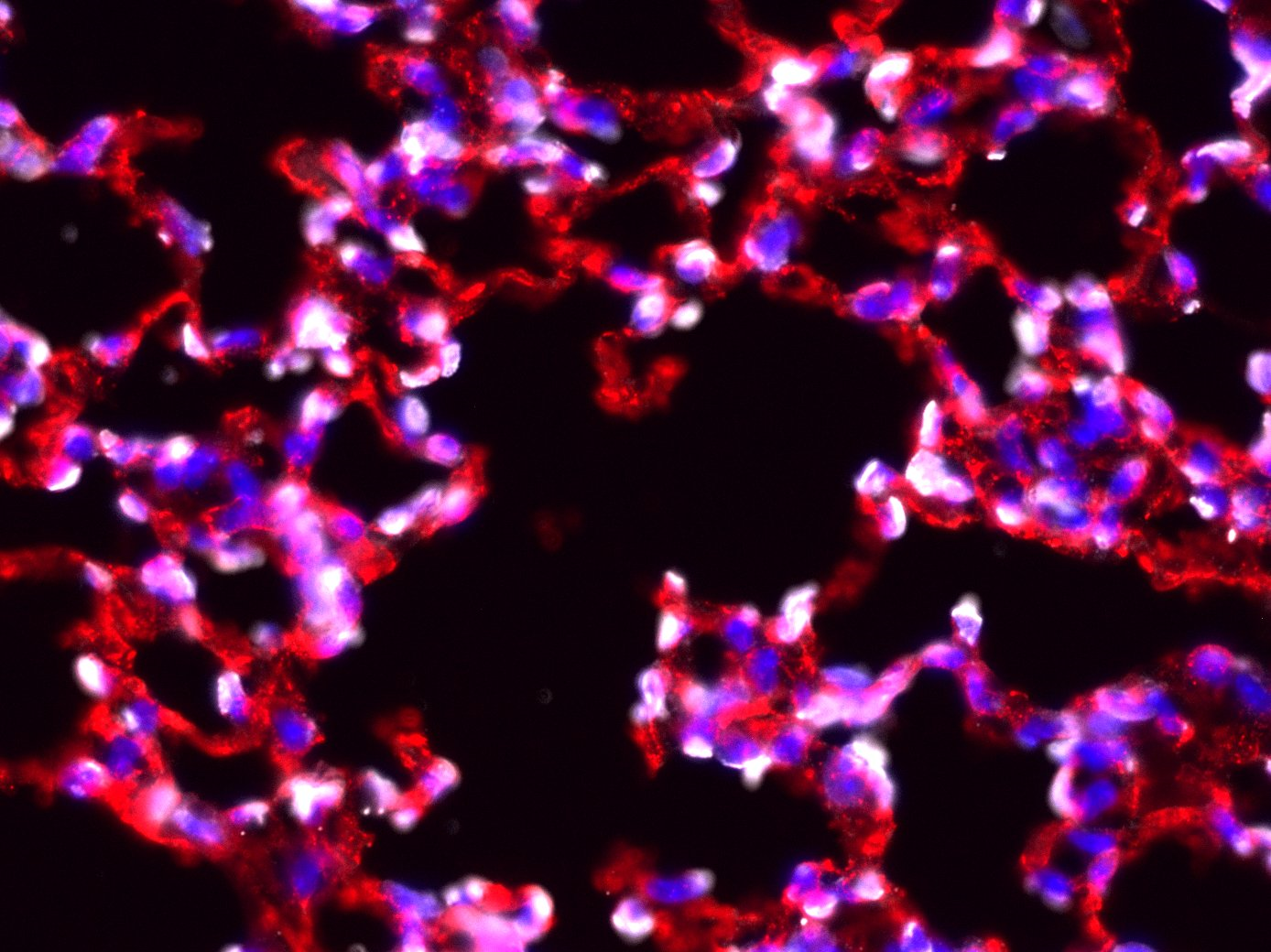

Supplement: Supplementary file 7 — Source Data Fig. 5 [file 44319_2023_41_MOESM7_ESM.zip › Source data Figure 5/5C Image data Micr image/p21++ MERGE.tif]

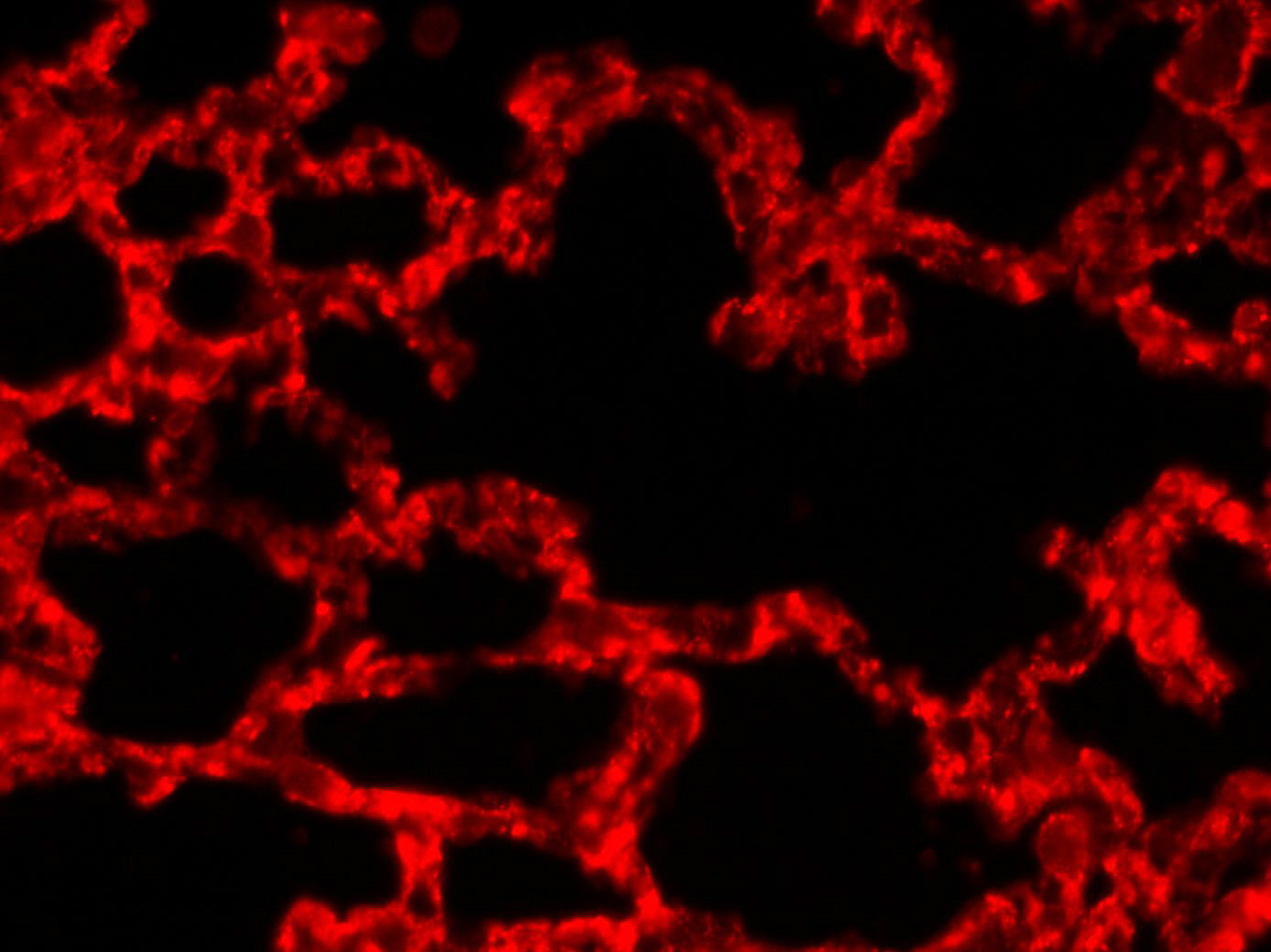

Supplement: Supplementary file 7 — Source Data Fig. 5 [file 44319_2023_41_MOESM7_ESM.zip › Source data Figure 5/5C Image data Micr image/p21+TERT CD31.tif]

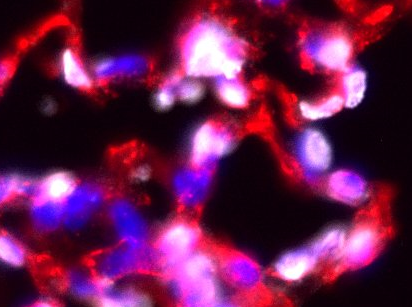

Supplement: Supplementary file 7 — Source Data Fig. 5 [file 44319_2023_41_MOESM7_ESM.zip › Source data Figure 5/5C Image data Micr image/p21++ZOOM.tif]

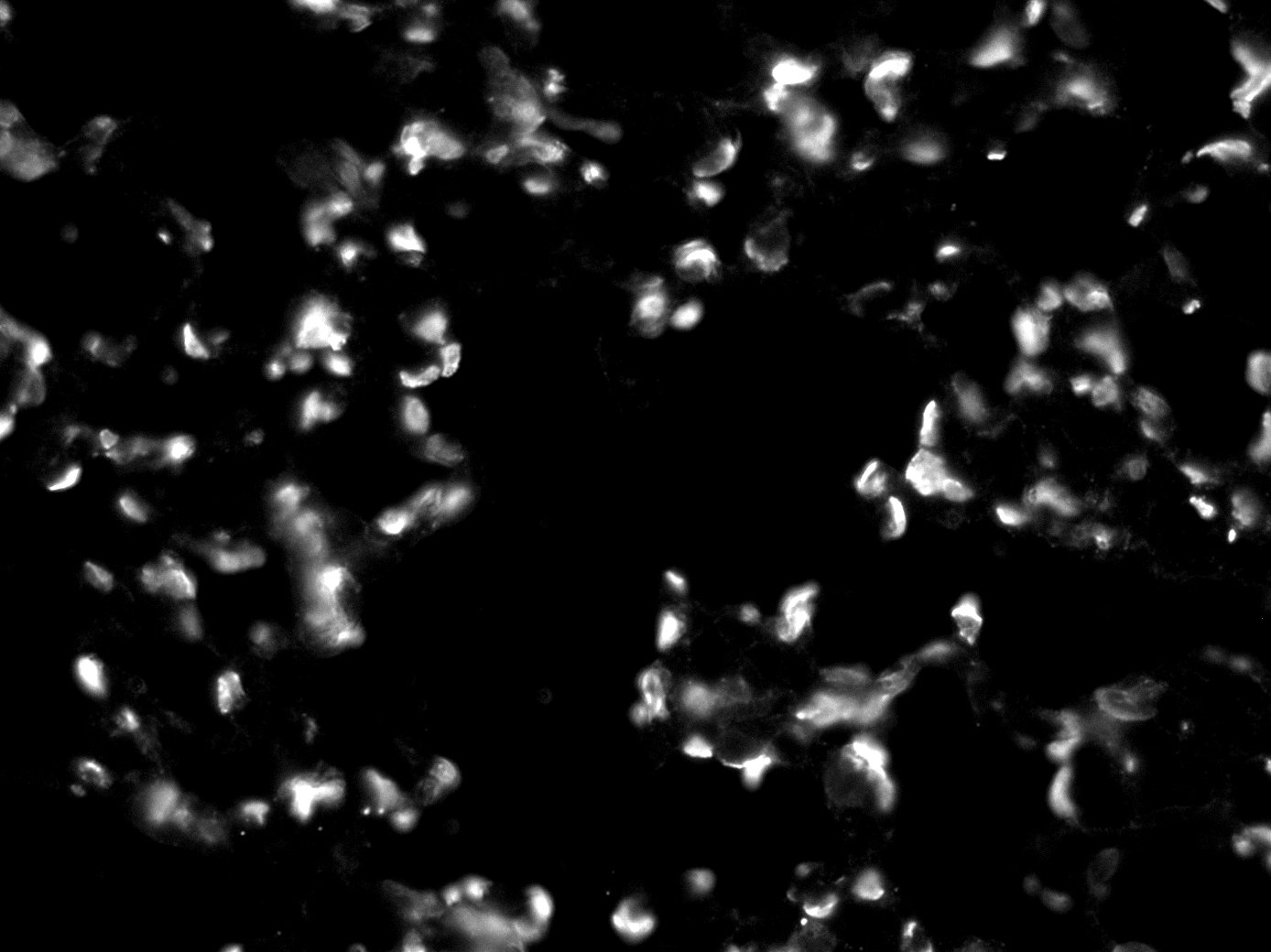

Supplement: Supplementary file 7 — Source Data Fig. 5 [file 44319_2023_41_MOESM7_ESM.zip › Source data Figure 5/5C Image data Micr image/p21++ p16.tif]

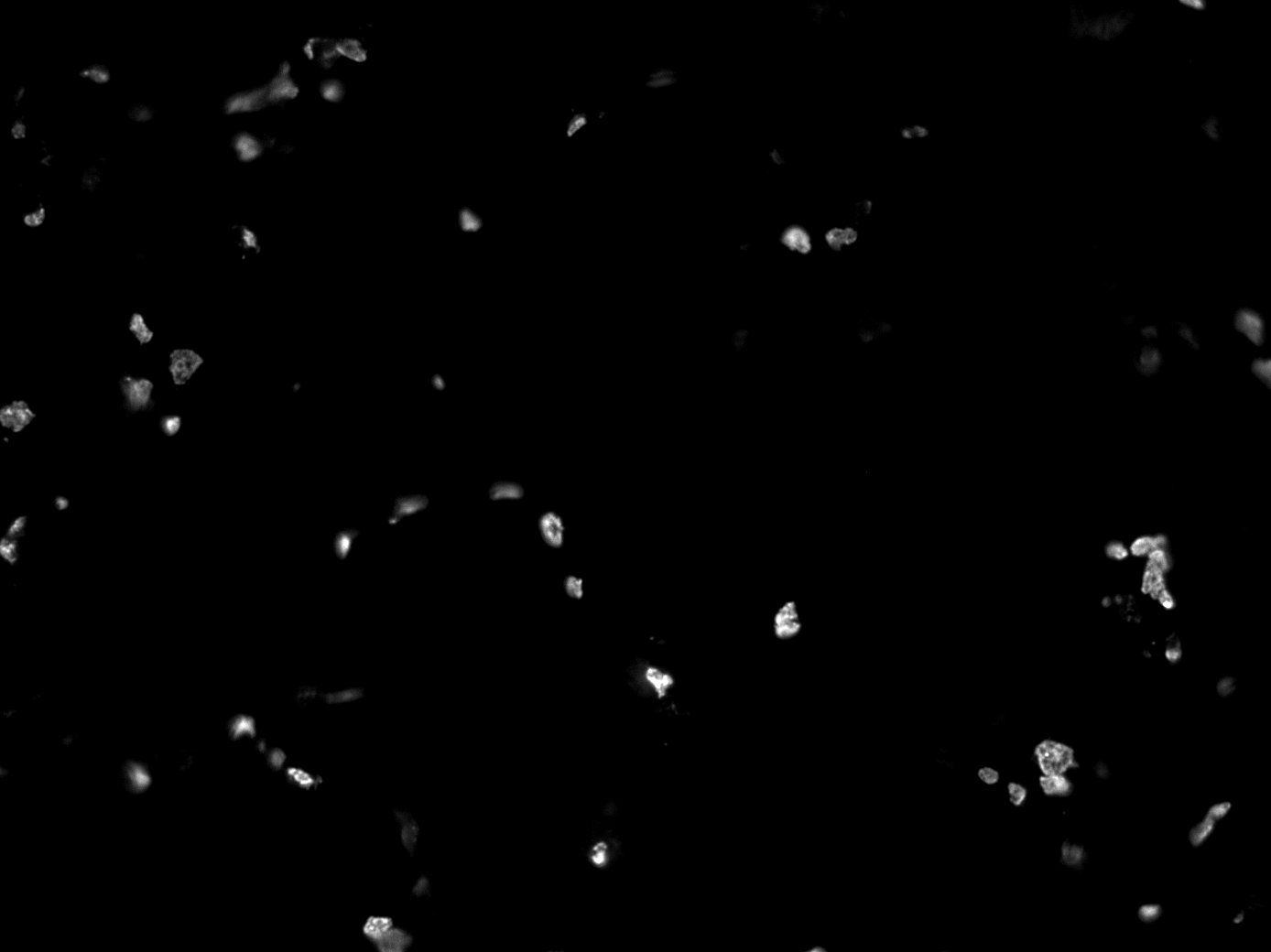

Supplement: Supplementary file 7 — Source Data Fig. 5 [file 44319_2023_41_MOESM7_ESM.zip › Source data Figure 5/5C Image data Micr image/p21+TERT p16.tif]

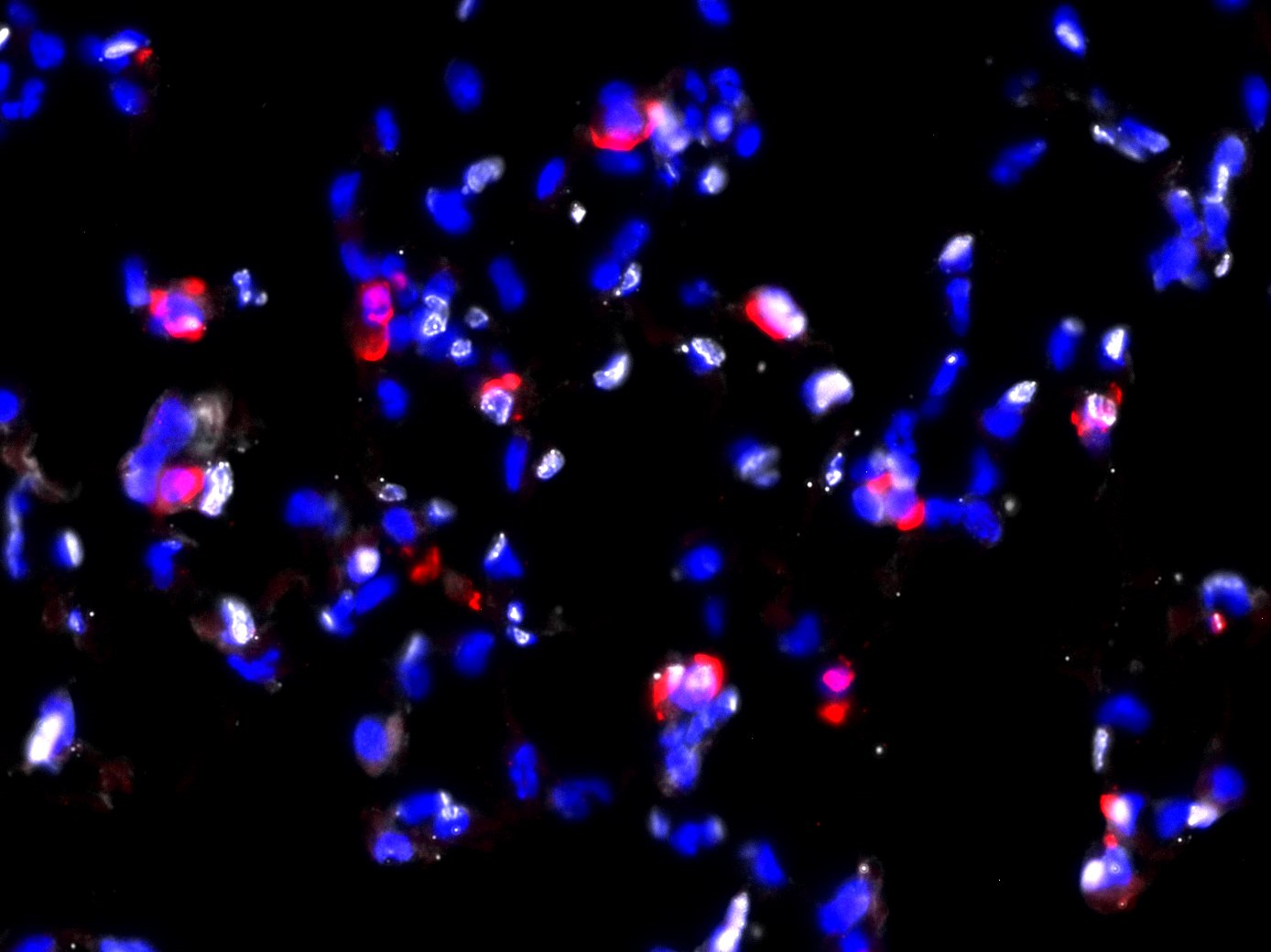

Supplement: Supplementary file 7 — Source Data Fig. 5 [file 44319_2023_41_MOESM7_ESM.zip › Source data Figure 5/5A Image data Micr image/p21+- MERGE .tif]

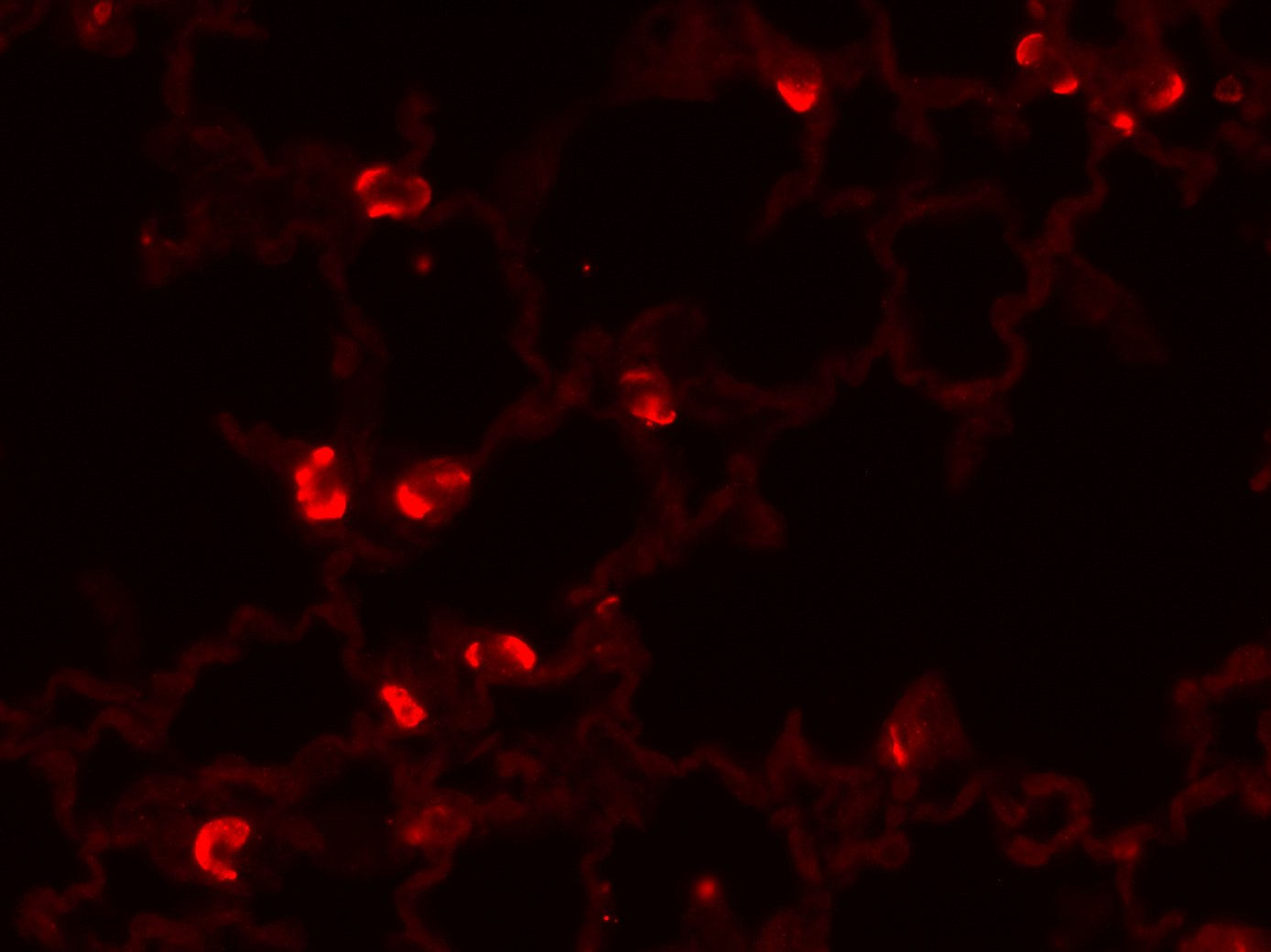

Supplement: Supplementary file 7 — Source Data Fig. 5 [file 44319_2023_41_MOESM7_ESM.zip › Source data Figure 5/5A Image data Micr image/p21+TERT CI MUC1.tif]

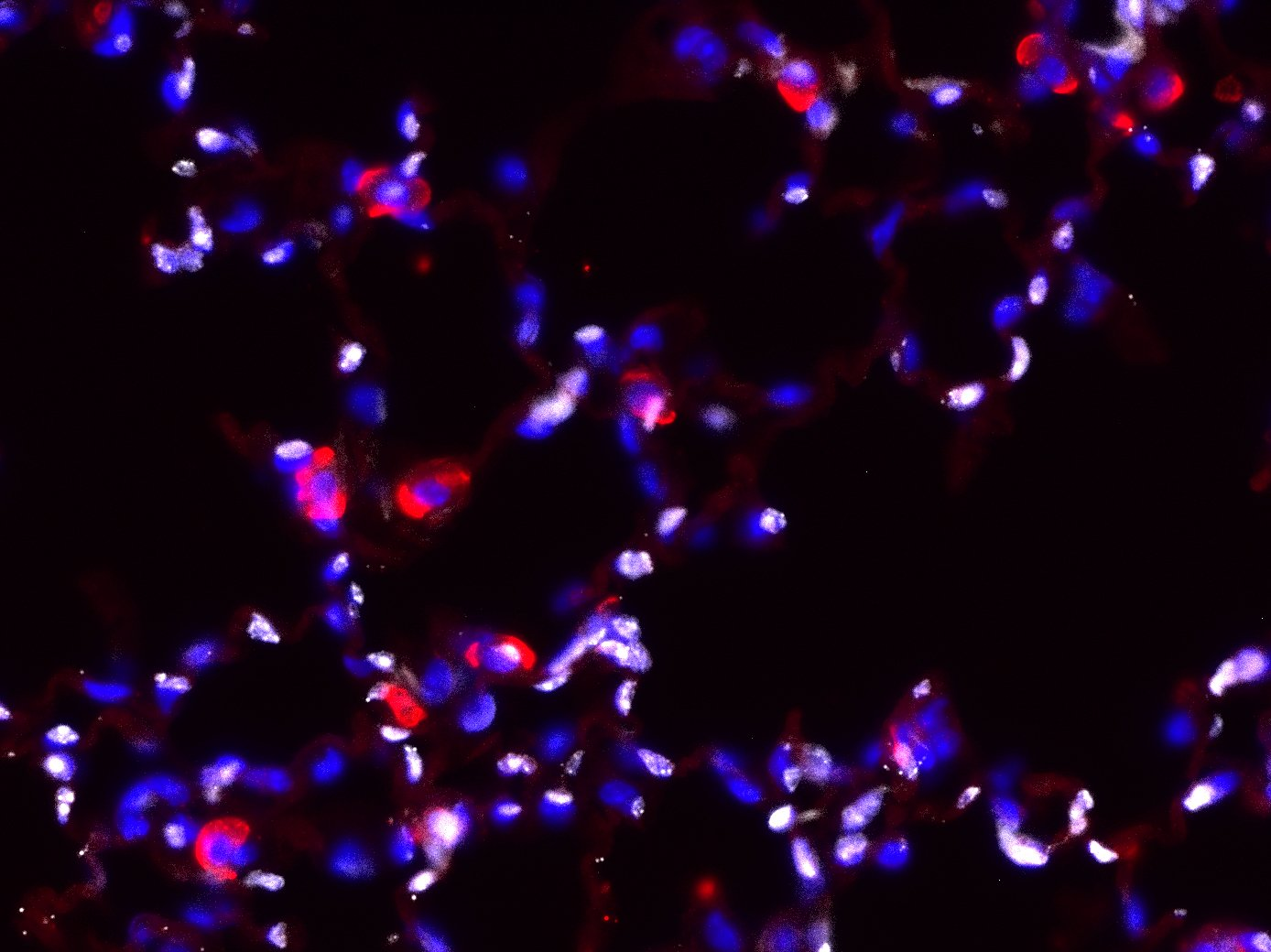

Supplement: Supplementary file 7 — Source Data Fig. 5 [file 44319_2023_41_MOESM7_ESM.zip › Source data Figure 5/5A Image data Micr image/p21+TERT CI MERGE.tif]

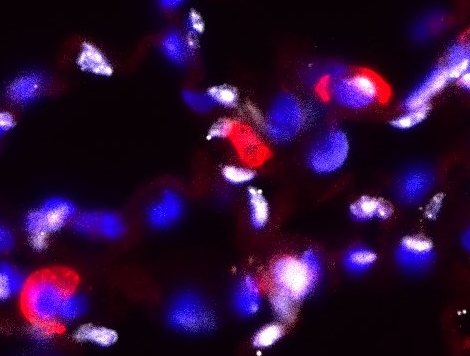

Supplement: Supplementary file 7 — Source Data Fig. 5 [file 44319_2023_41_MOESM7_ESM.zip › Source data Figure 5/5A Image data Micr image/p21+TERT CI zoom.tif]

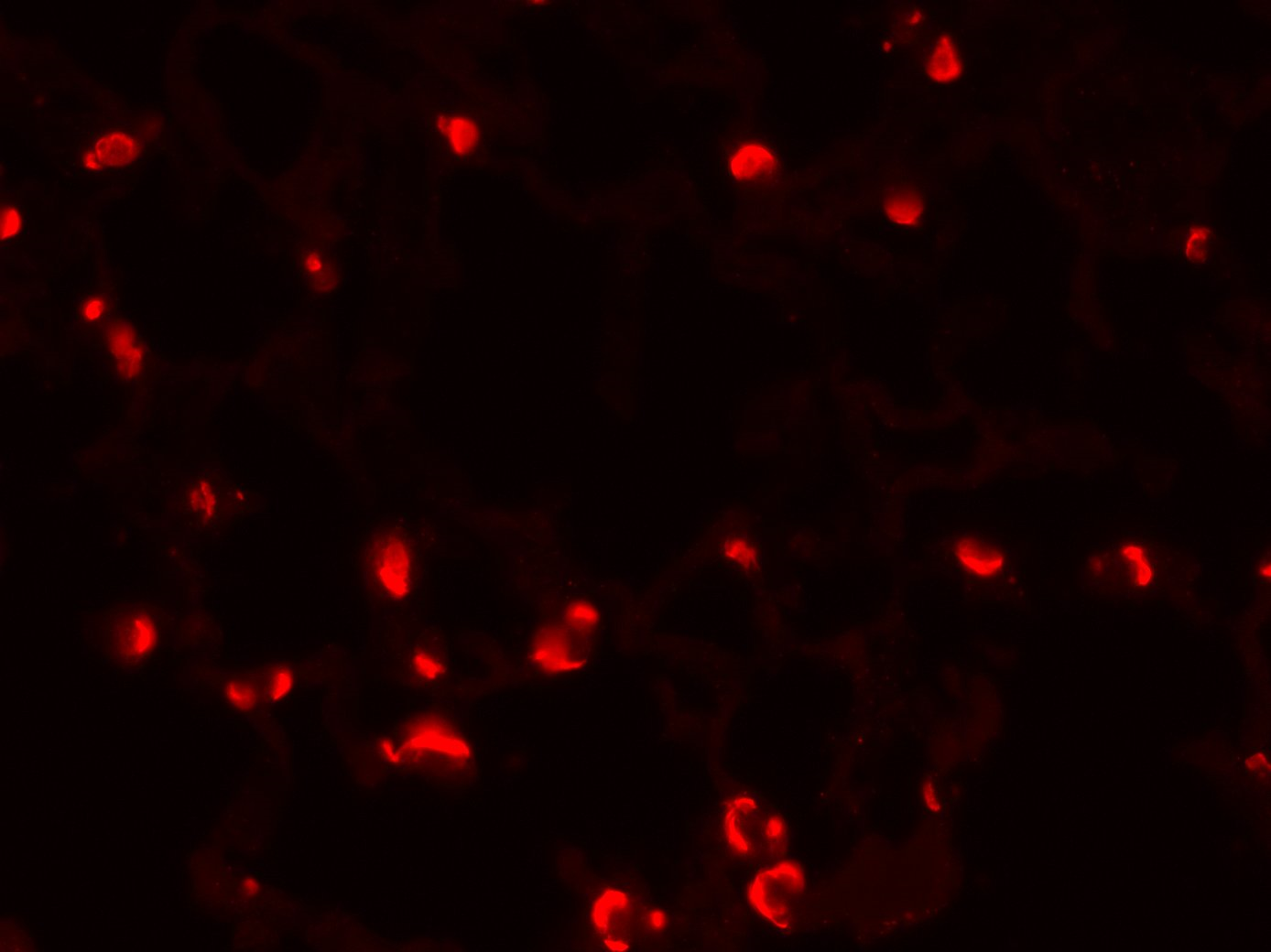

Supplement: Supplementary file 7 — Source Data Fig. 5 [file 44319_2023_41_MOESM7_ESM.zip › Source data Figure 5/5A Image data Micr image/p21+TERT MUC1.tif]

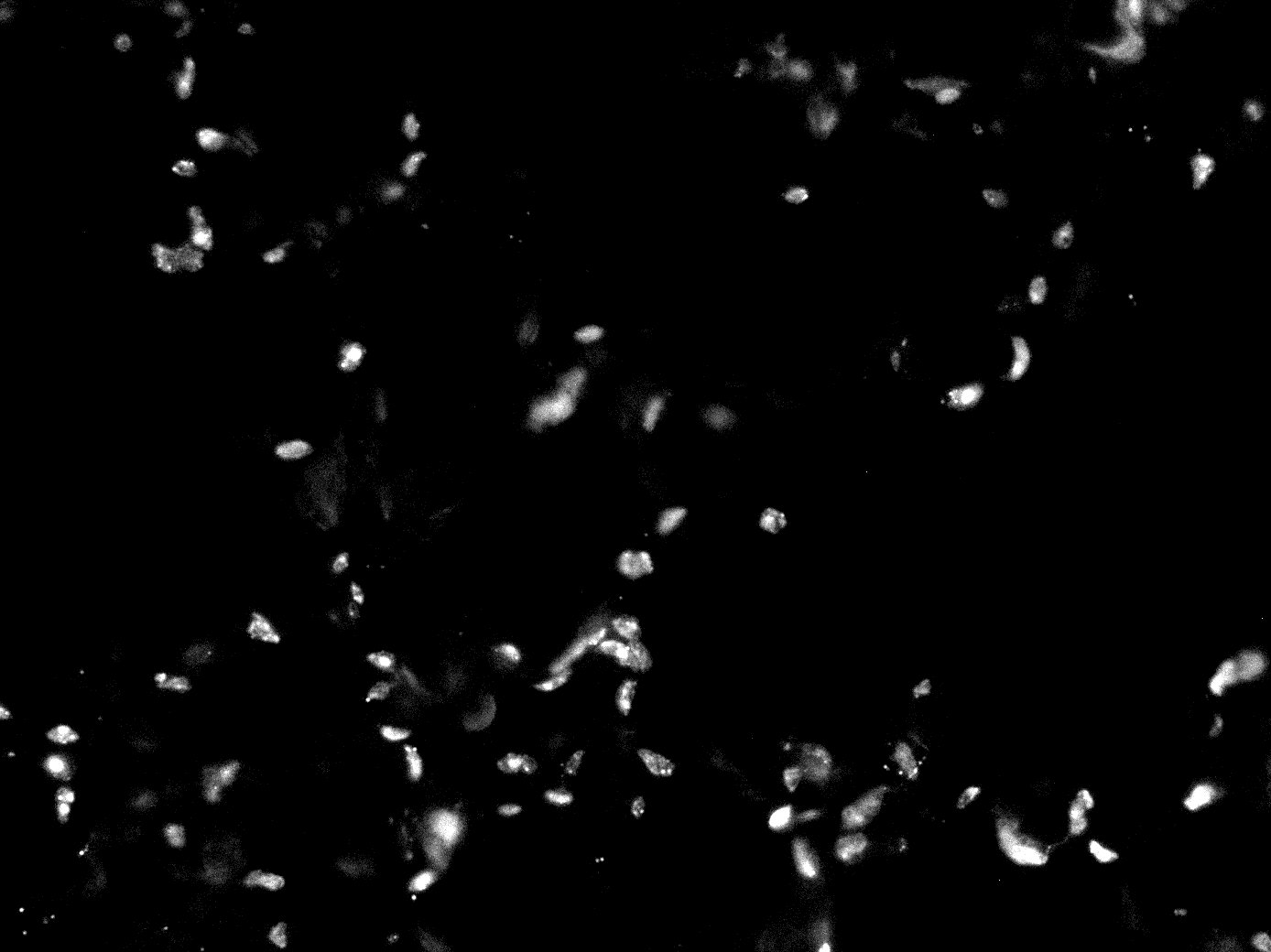

Supplement: Supplementary file 7 — Source Data Fig. 5 [file 44319_2023_41_MOESM7_ESM.zip › Source data Figure 5/5A Image data Micr image/p21+TERT CI p16.tif]

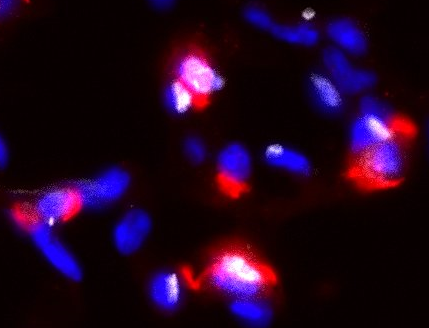

Supplement: Supplementary file 7 — Source Data Fig. 5 [file 44319_2023_41_MOESM7_ESM.zip › Source data Figure 5/5A Image data Micr image/p21+TERT zoom.tif]

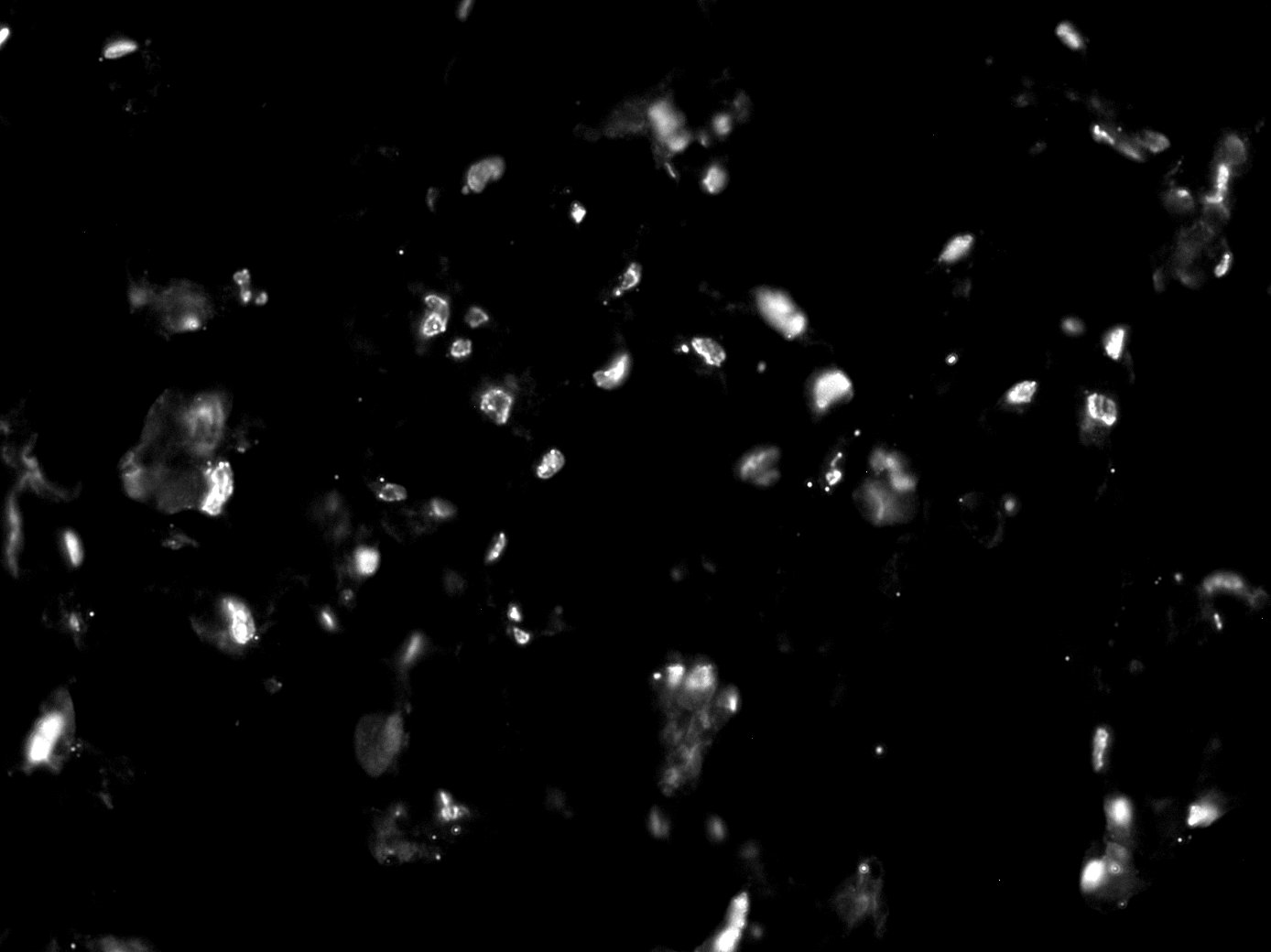

Supplement: Supplementary file 7 — Source Data Fig. 5 [file 44319_2023_41_MOESM7_ESM.zip › Source data Figure 5/5A Image data Micr image/p21+- p16.tif]

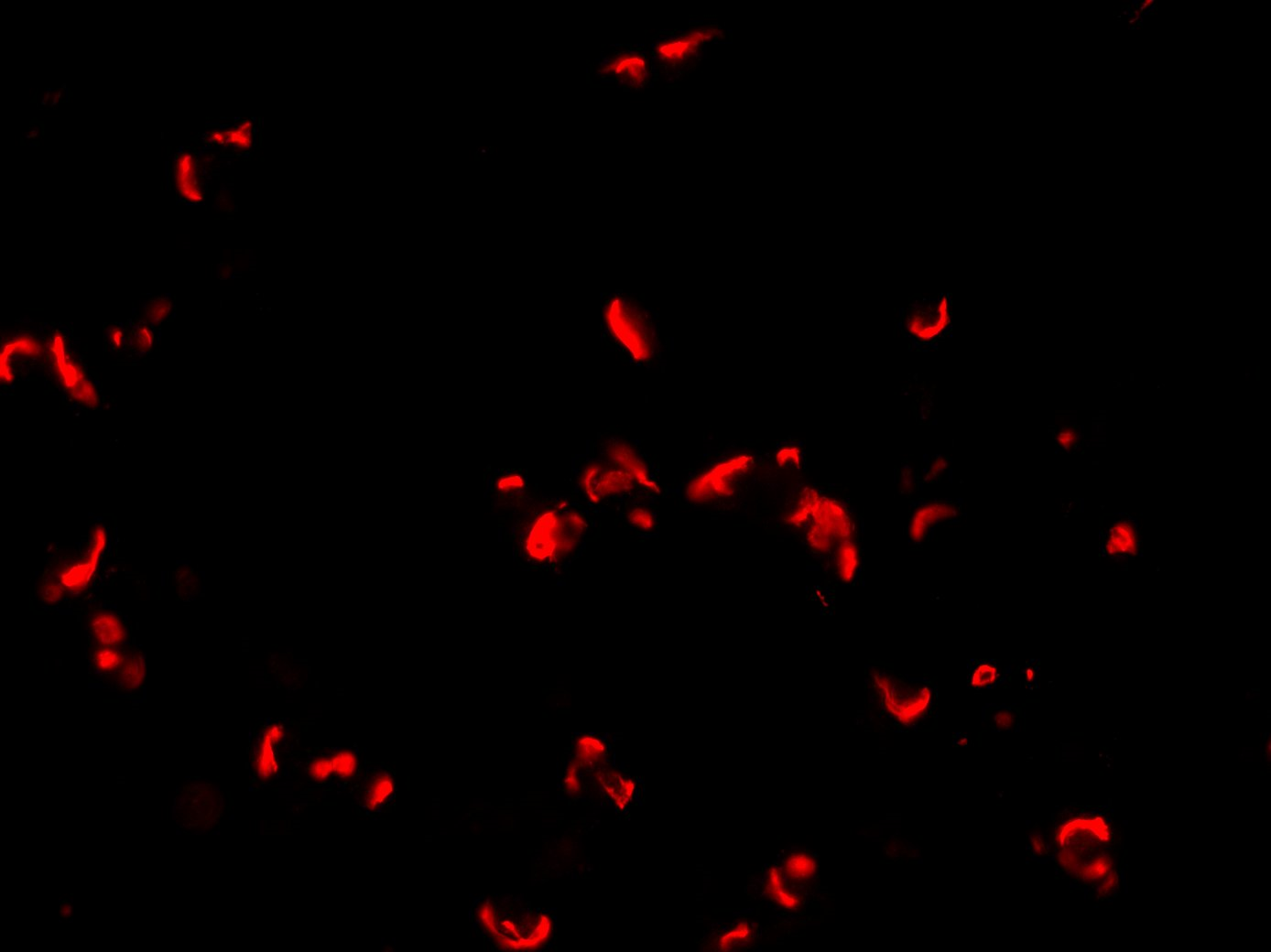

Supplement: Supplementary file 7 — Source Data Fig. 5 [file 44319_2023_41_MOESM7_ESM.zip › Source data Figure 5/5A Image data Micr image/p21++ MUC1.tif]

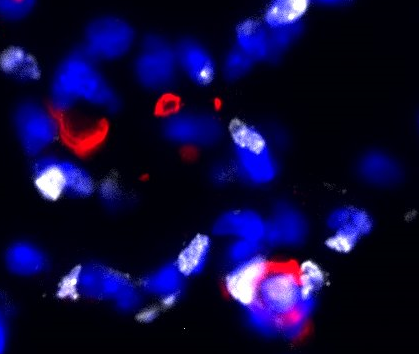

Supplement: Supplementary file 7 — Source Data Fig. 5 [file 44319_2023_41_MOESM7_ESM.zip › Source data Figure 5/5A Image data Micr image/p21++ ZOOM.tif]

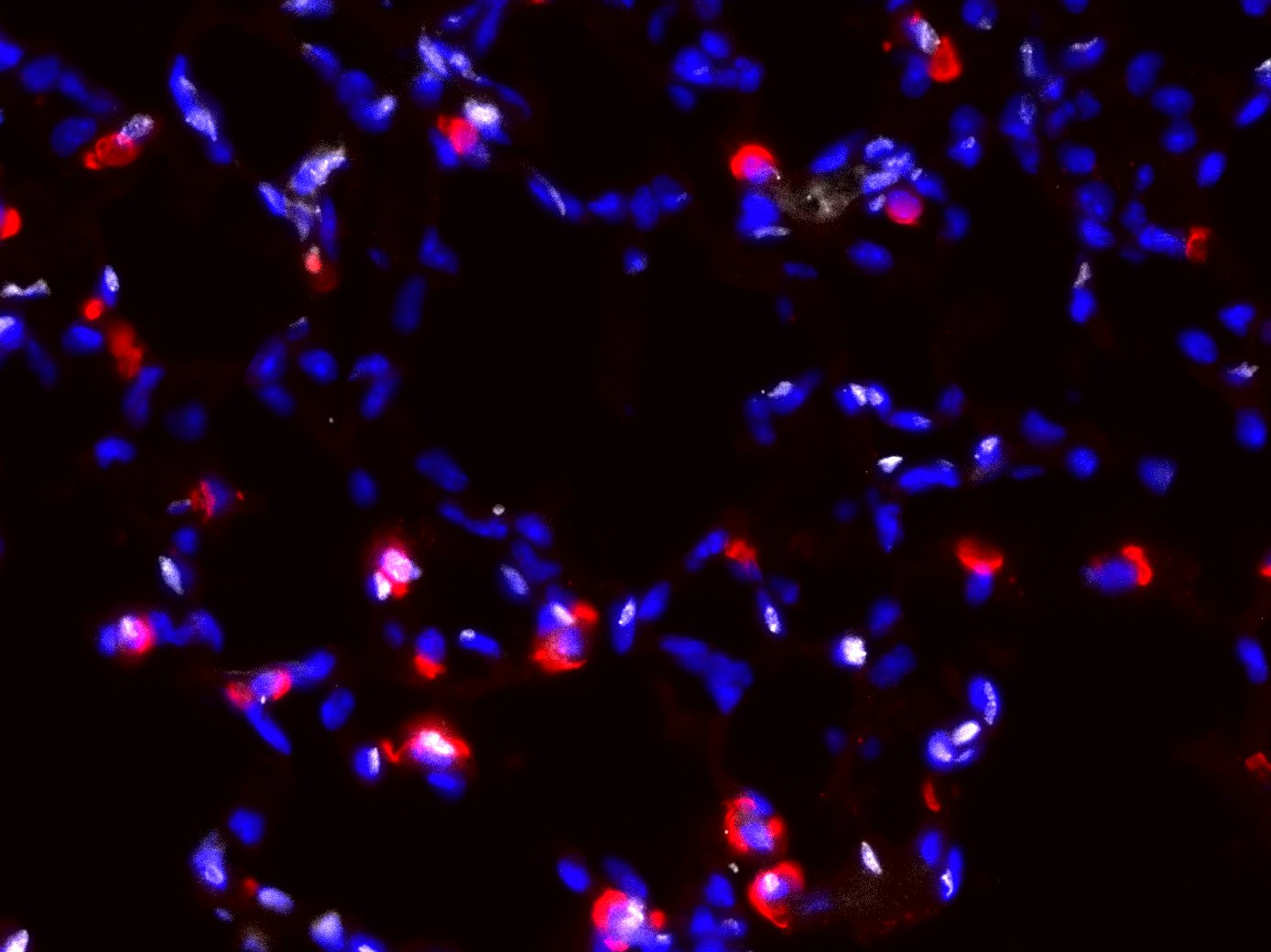

Supplement: Supplementary file 7 — Source Data Fig. 5 [file 44319_2023_41_MOESM7_ESM.zip › Source data Figure 5/5A Image data Micr image/p21+TERT MERGE.tif]

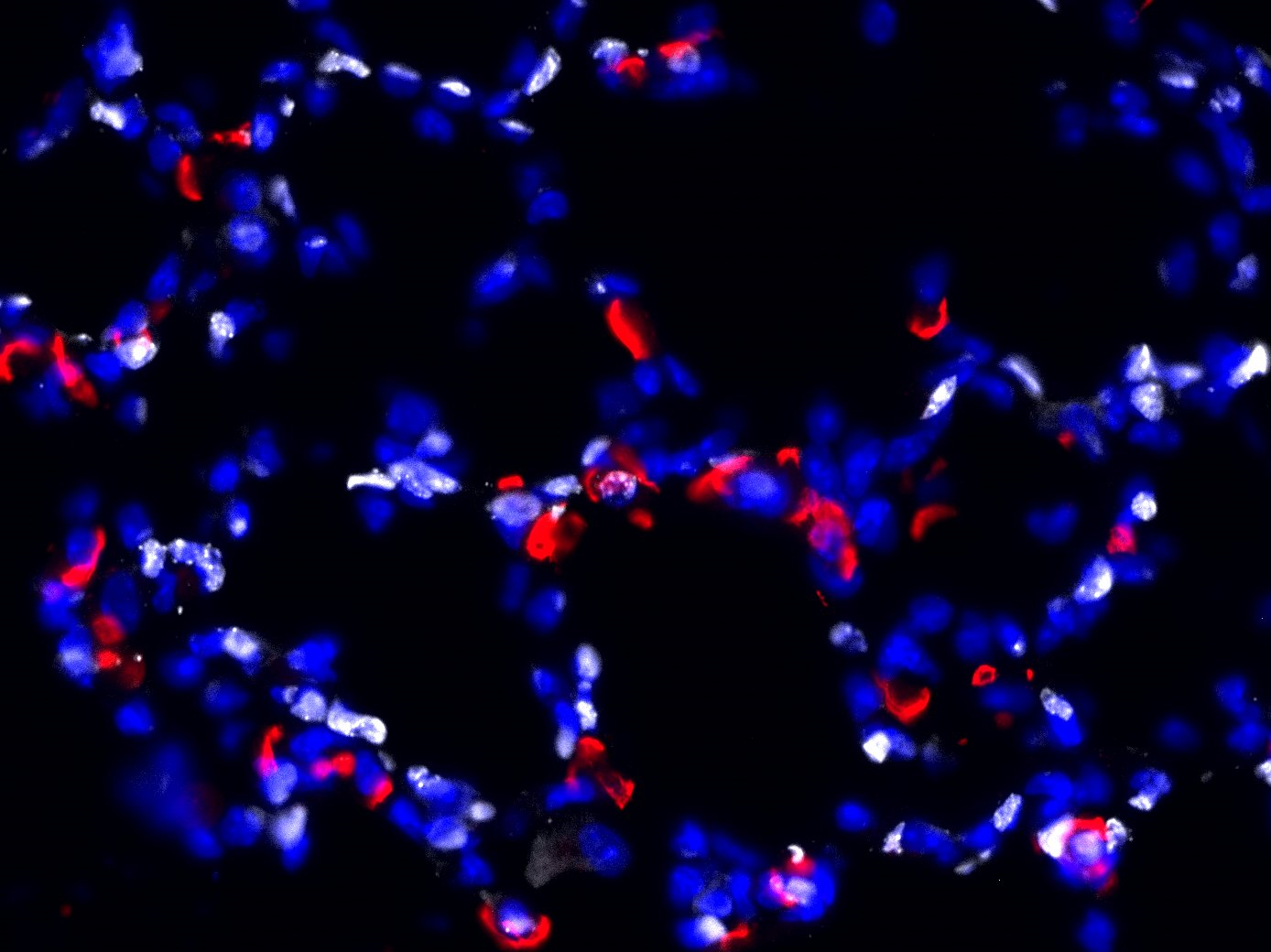

Supplement: Supplementary file 7 — Source Data Fig. 5 [file 44319_2023_41_MOESM7_ESM.zip › Source data Figure 5/5A Image data Micr image/p21++ MERGE.tif]

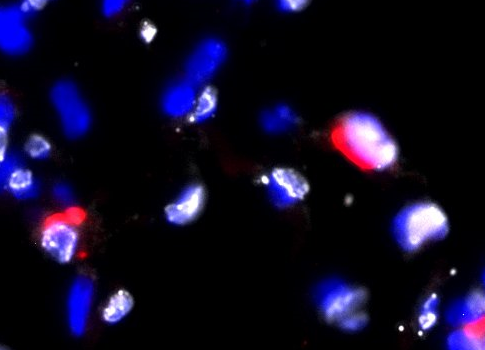

Supplement: Supplementary file 7 — Source Data Fig. 5 [file 44319_2023_41_MOESM7_ESM.zip › Source data Figure 5/5A Image data Micr image/p21+- ZOOM.tif]

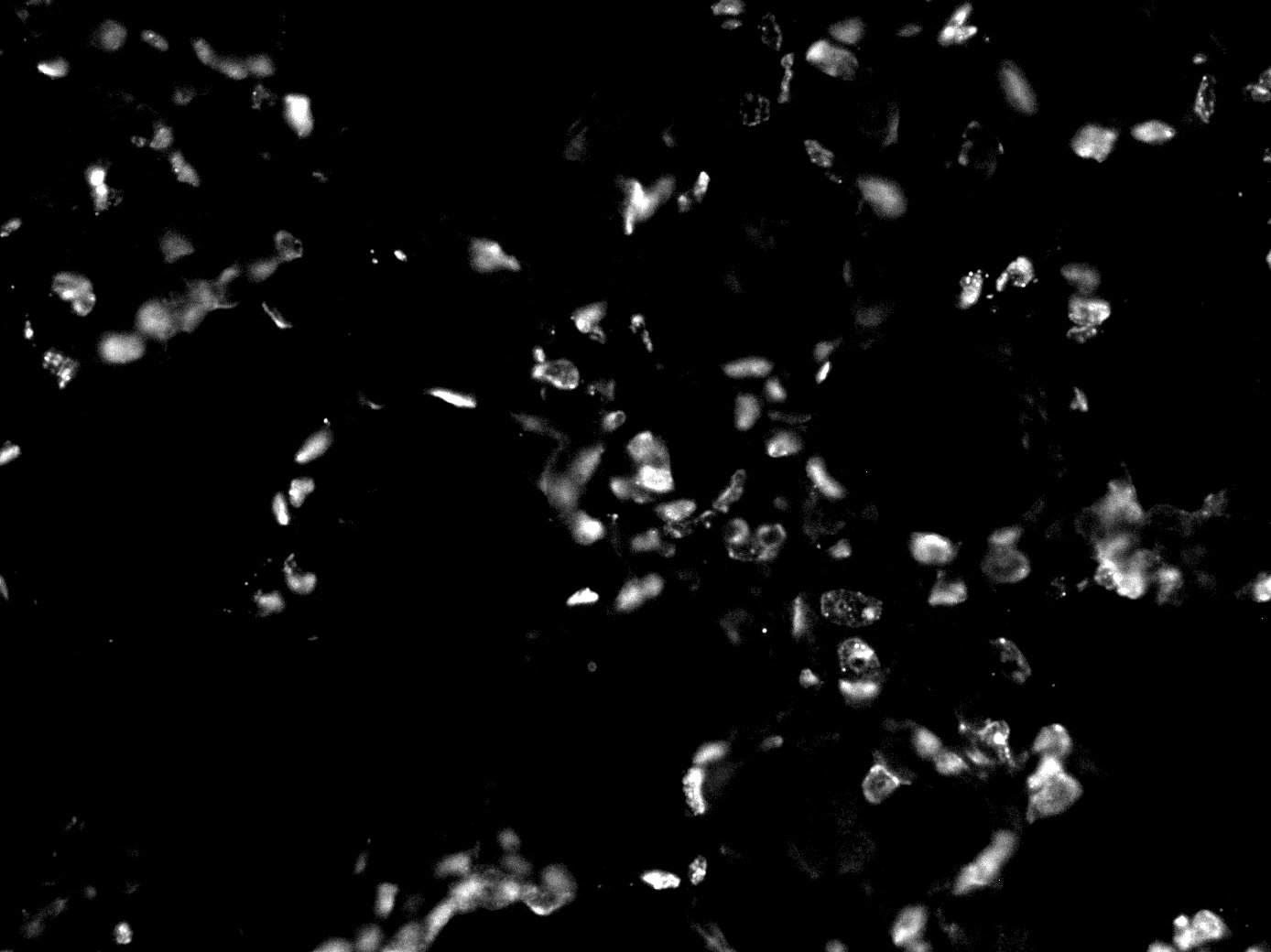

Supplement: Supplementary file 7 — Source Data Fig. 5 [file 44319_2023_41_MOESM7_ESM.zip › Source data Figure 5/5A Image data Micr image/p21++ p16.tif]

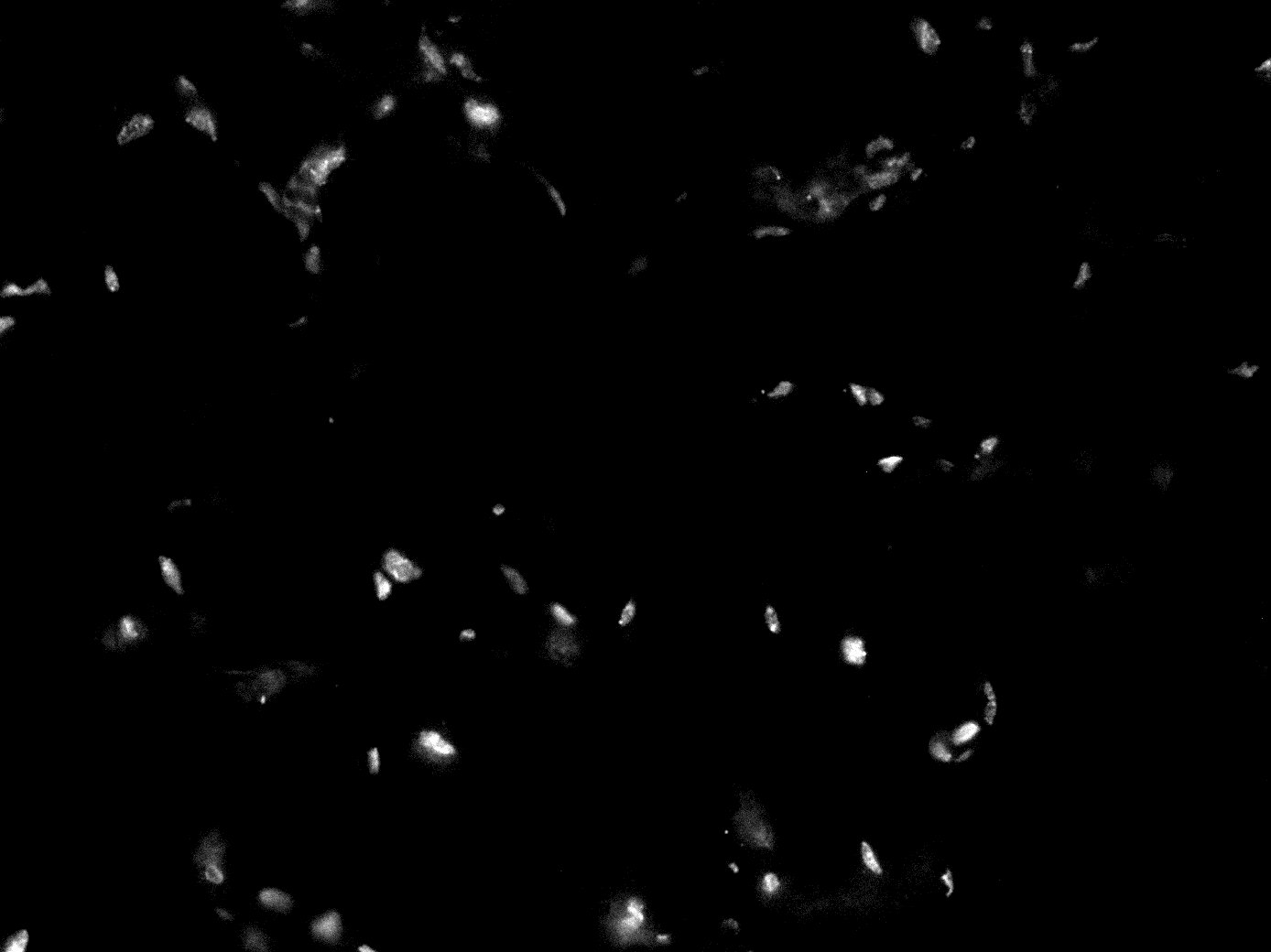

Supplement: Supplementary file 7 — Source Data Fig. 5 [file 44319_2023_41_MOESM7_ESM.zip › Source data Figure 5/5A Image data Micr image/p21+TERT p16.tif]

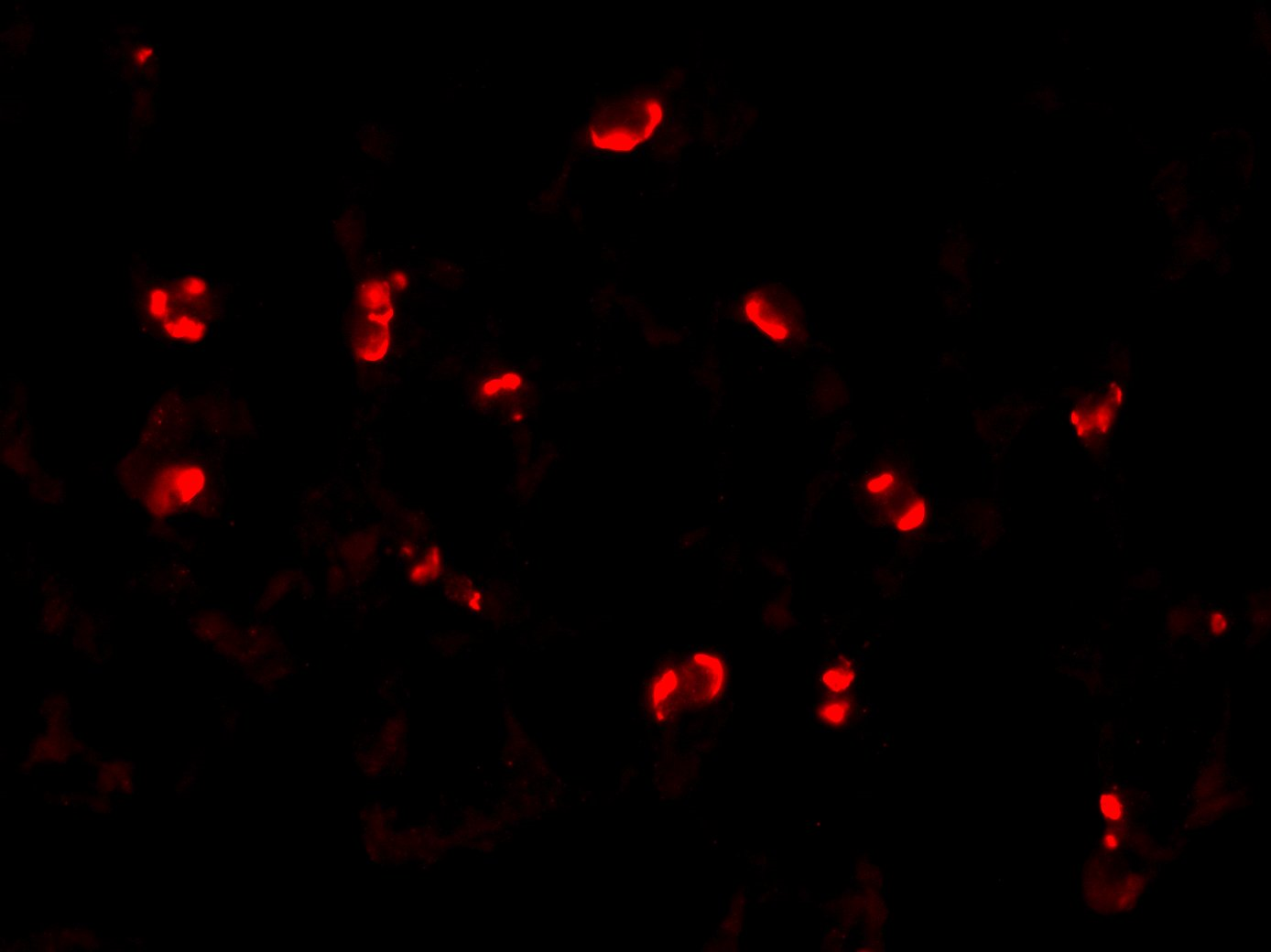

Supplement: Supplementary file 7 — Source Data Fig. 5 [file 44319_2023_41_MOESM7_ESM.zip › Source data Figure 5/5A Image data Micr image/p21+- MUC1.tif]
