# Supplementary figures and images for: mTert induction in p21-positive cells counteracts capillary rarefaction and pulmonary emphysema (part 2 of 3)
Source: EMBO Rep. 2024 Feb 29;25(3):36. doi: 10.1038/s44319-023-00041-1 (PMC10933469; doi:10.1038/s44319-023-00041-1)

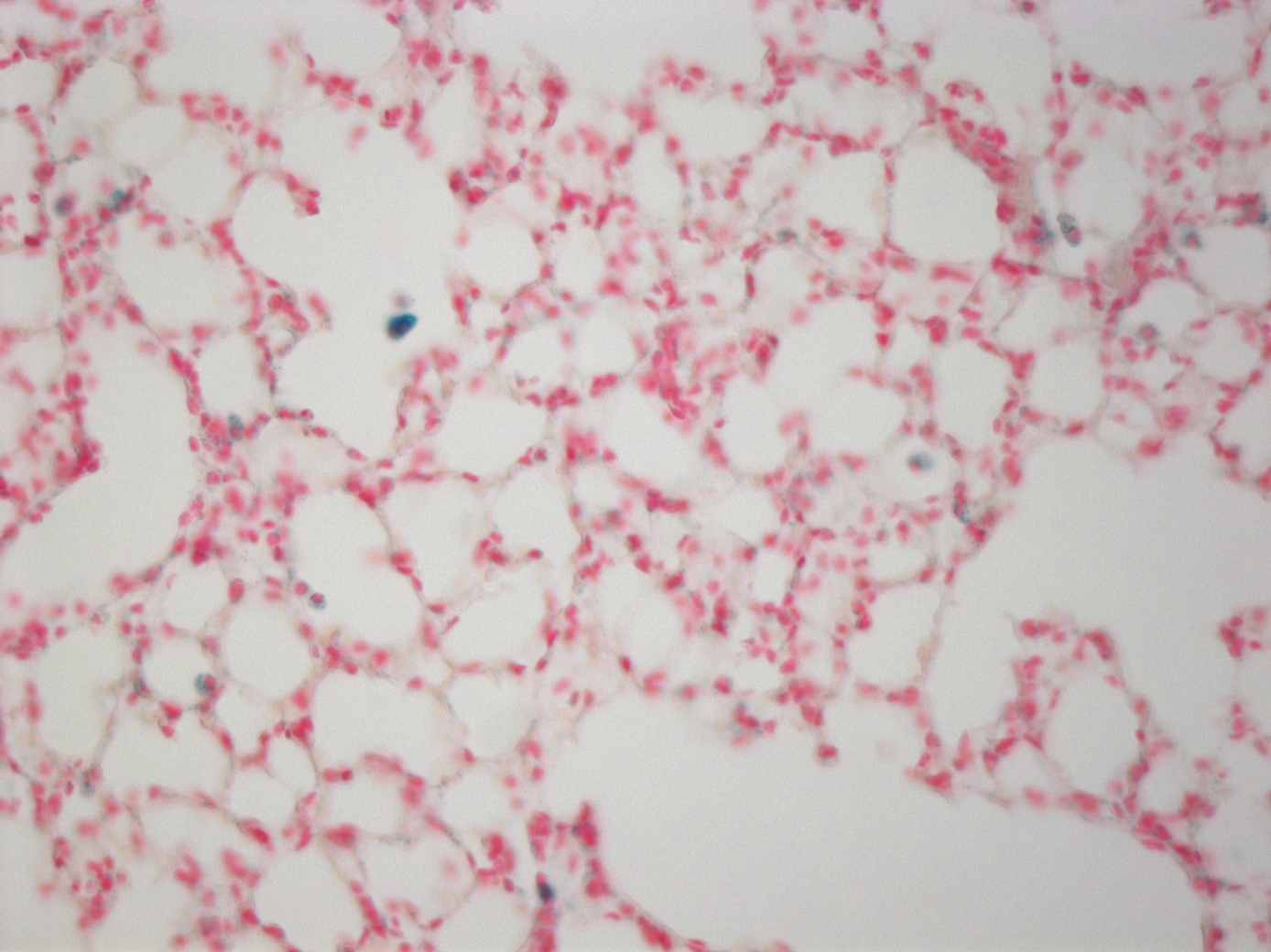

Supplement: Supplementary file 7 — Source Data Fig. 5 [file 44319_2023_41_MOESM7_ESM.zip › Source data Figure 5/5E image data Micr image/p21+- TERT.tif]

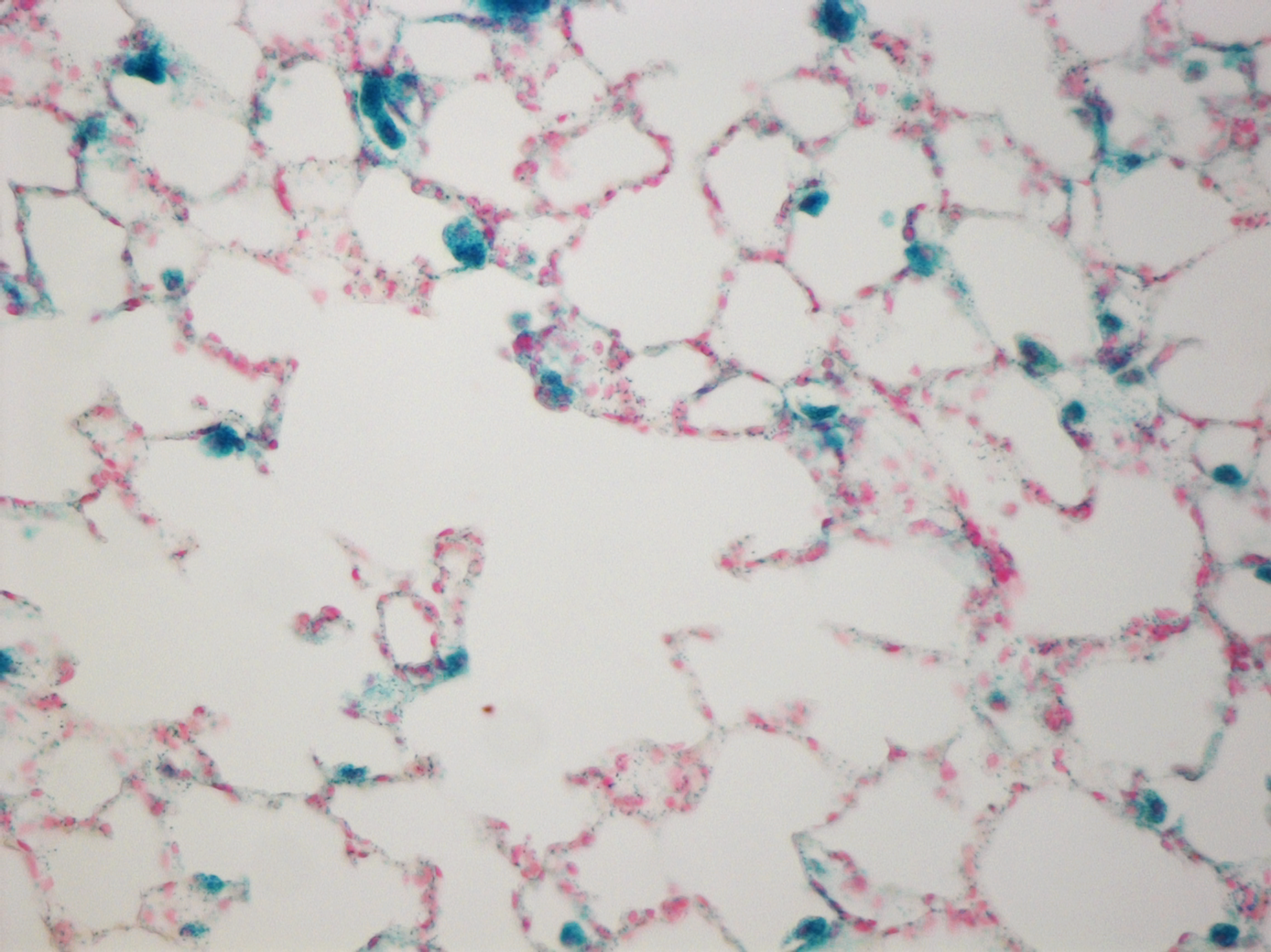

Supplement: Supplementary file 7 — Source Data Fig. 5 [file 44319_2023_41_MOESM7_ESM.zip › Source data Figure 5/5E image data Micr image/p21+-.tif]

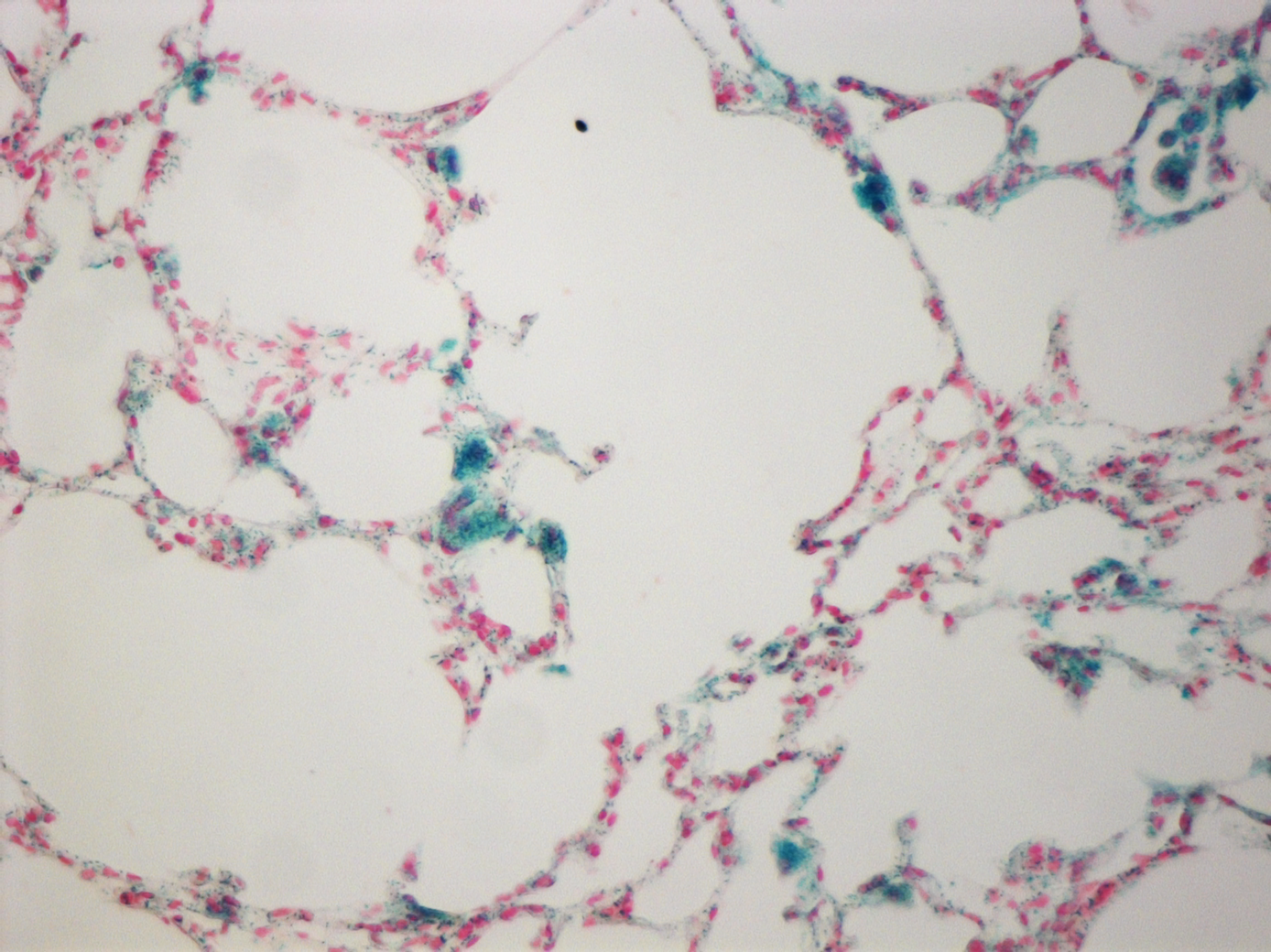

Supplement: Supplementary file 7 — Source Data Fig. 5 [file 44319_2023_41_MOESM7_ESM.zip › Source data Figure 5/5E image data Micr image/p21++.tif]

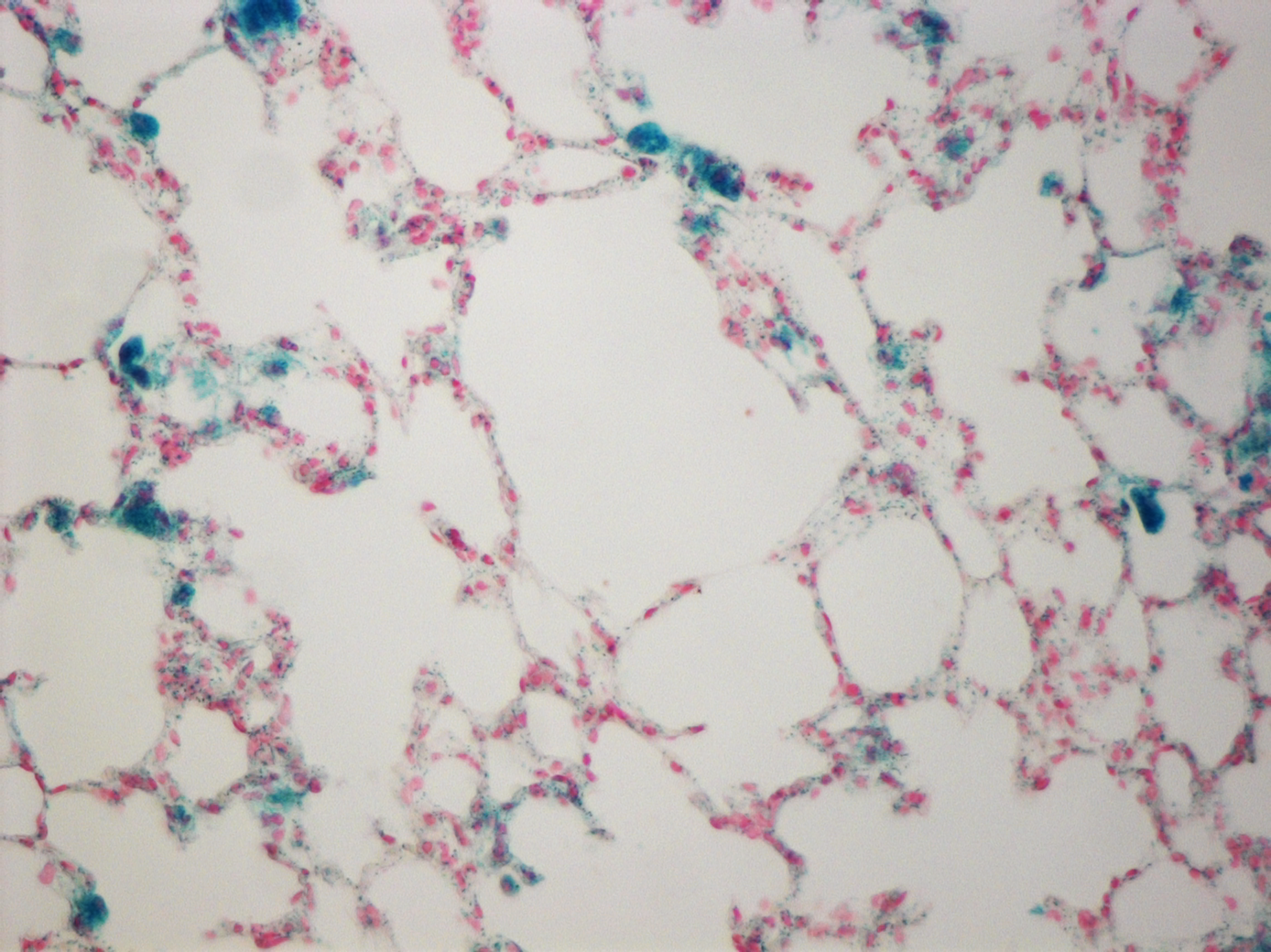

Supplement: Supplementary file 7 — Source Data Fig. 5 [file 44319_2023_41_MOESM7_ESM.zip › Source data Figure 5/5E image data Micr image/p21+TERT CI.tif]

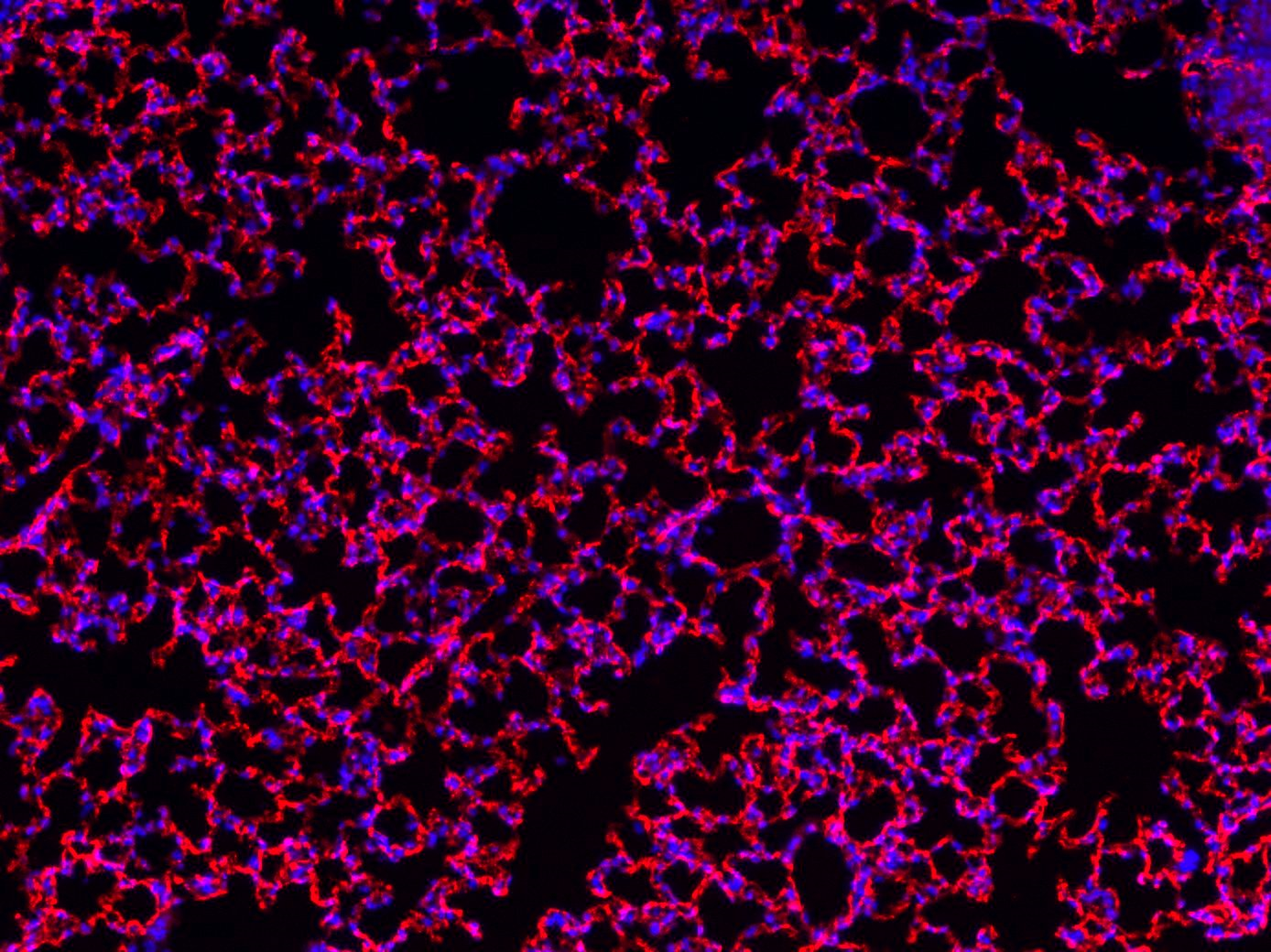

Supplement: Supplementary file 8 — Source Data Fig. 6 [file 44319_2023_41_MOESM8_ESM.zip › Source data Figure 6 /6A Image data Micr images/young p21+TERT merge.tif]

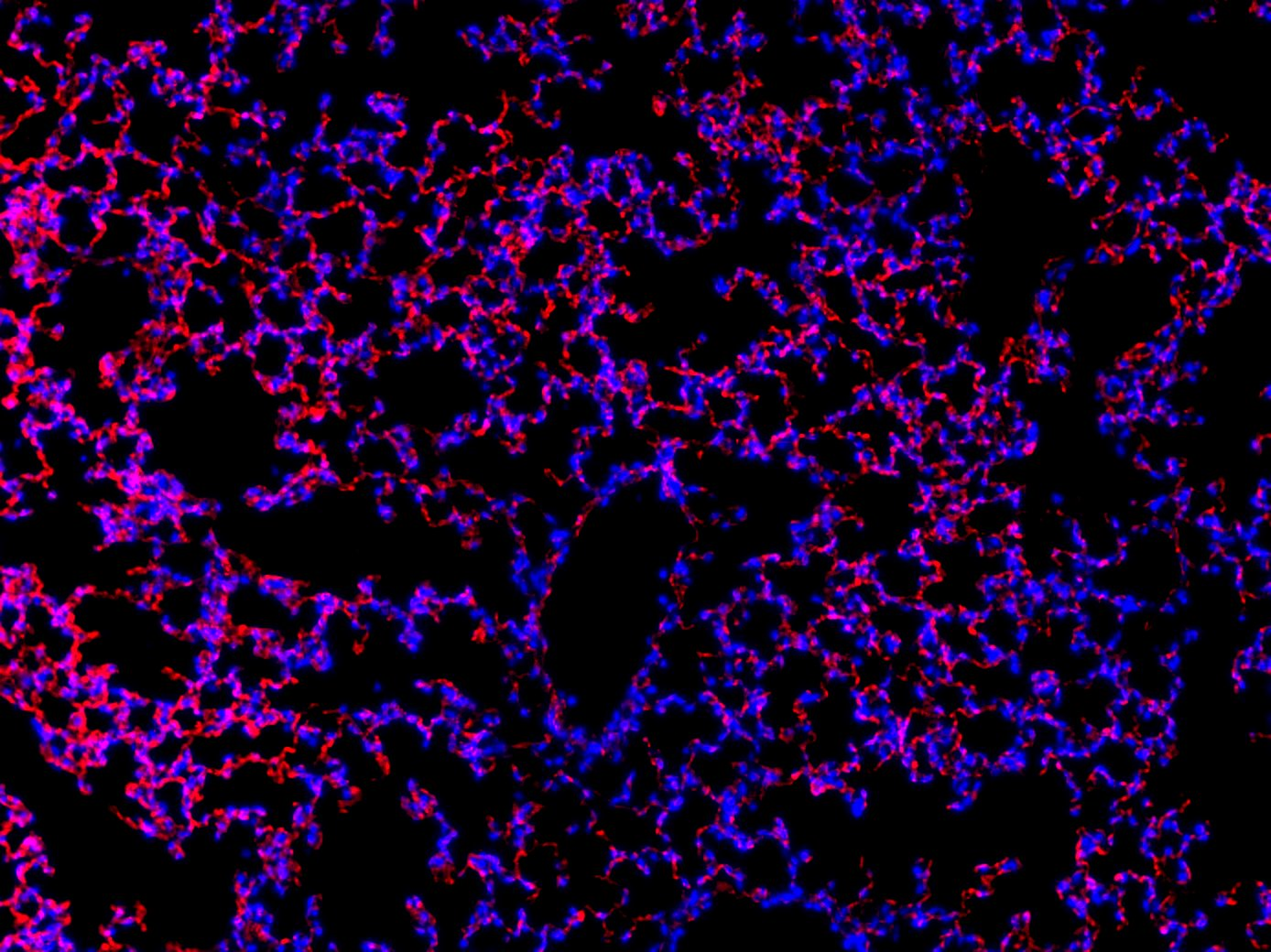

Supplement: Supplementary file 8 — Source Data Fig. 6 [file 44319_2023_41_MOESM8_ESM.zip › Source data Figure 6 /6A Image data Micr images/old p21++ merge.tif]

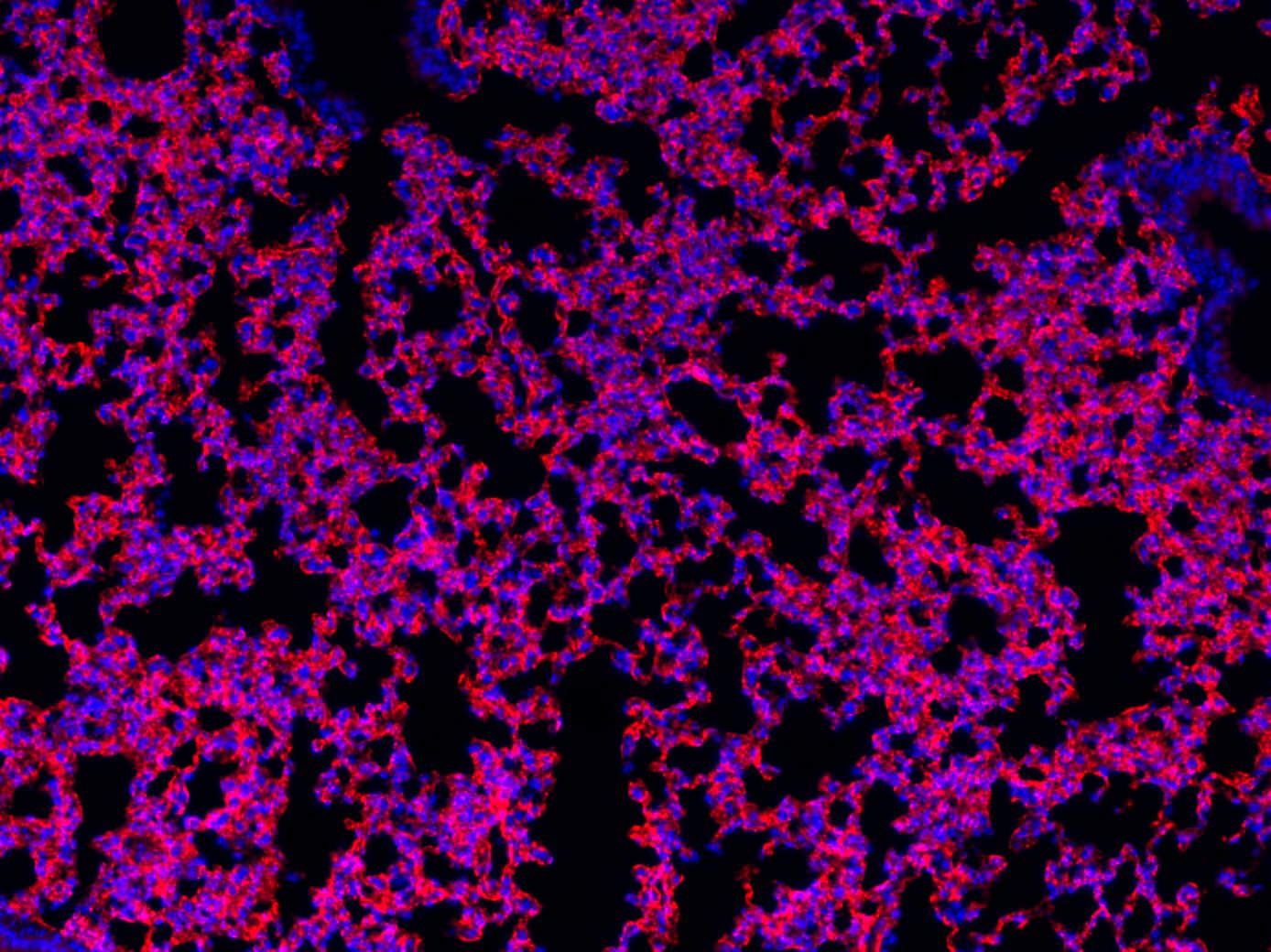

Supplement: Supplementary file 8 — Source Data Fig. 6 [file 44319_2023_41_MOESM8_ESM.zip › Source data Figure 6 /6A Image data Micr images/young p21+TERT CI merge.tif]

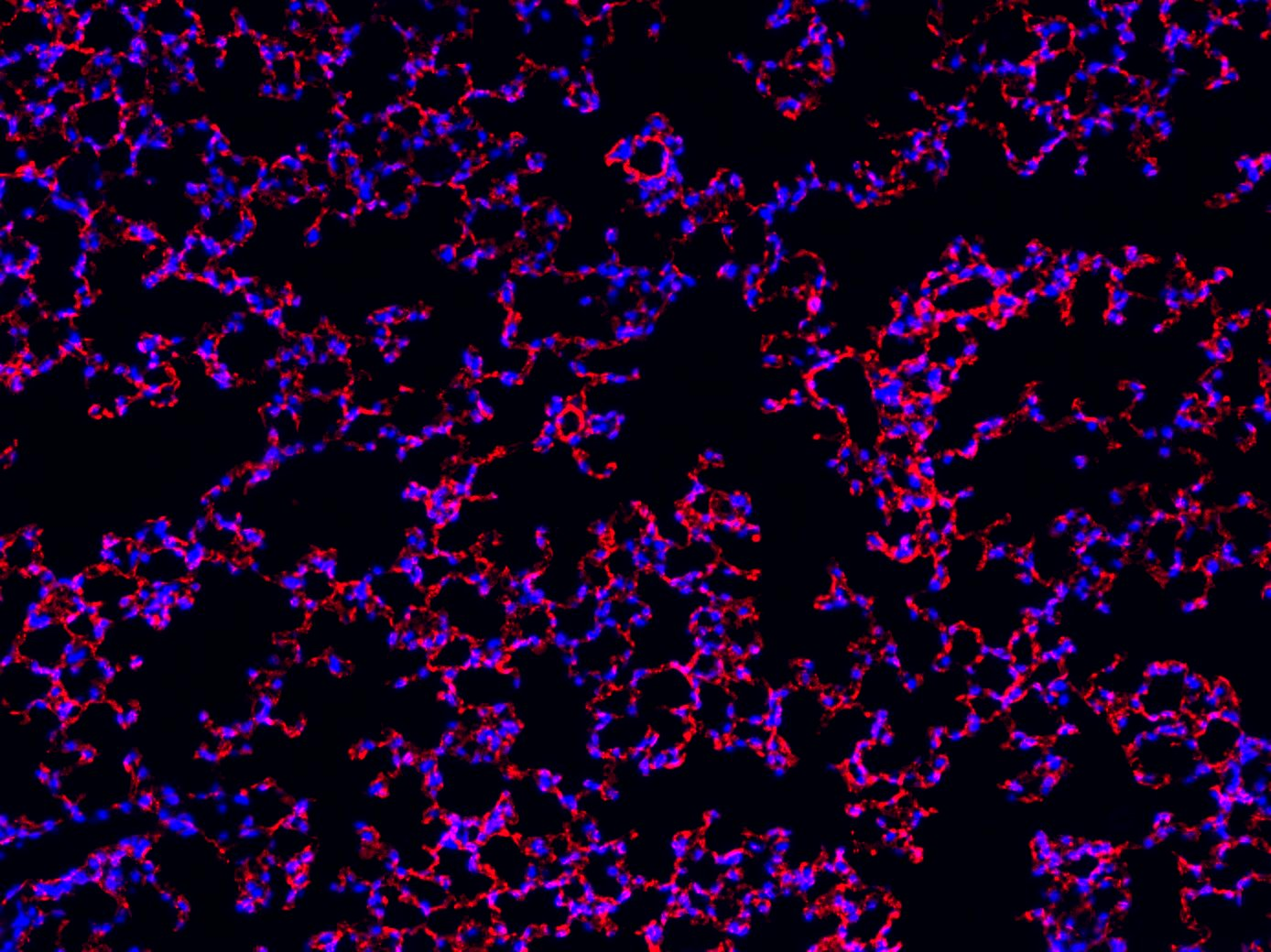

Supplement: Supplementary file 8 — Source Data Fig. 6 [file 44319_2023_41_MOESM8_ESM.zip › Source data Figure 6 /6A Image data Micr images/old p21+- merge.tif]

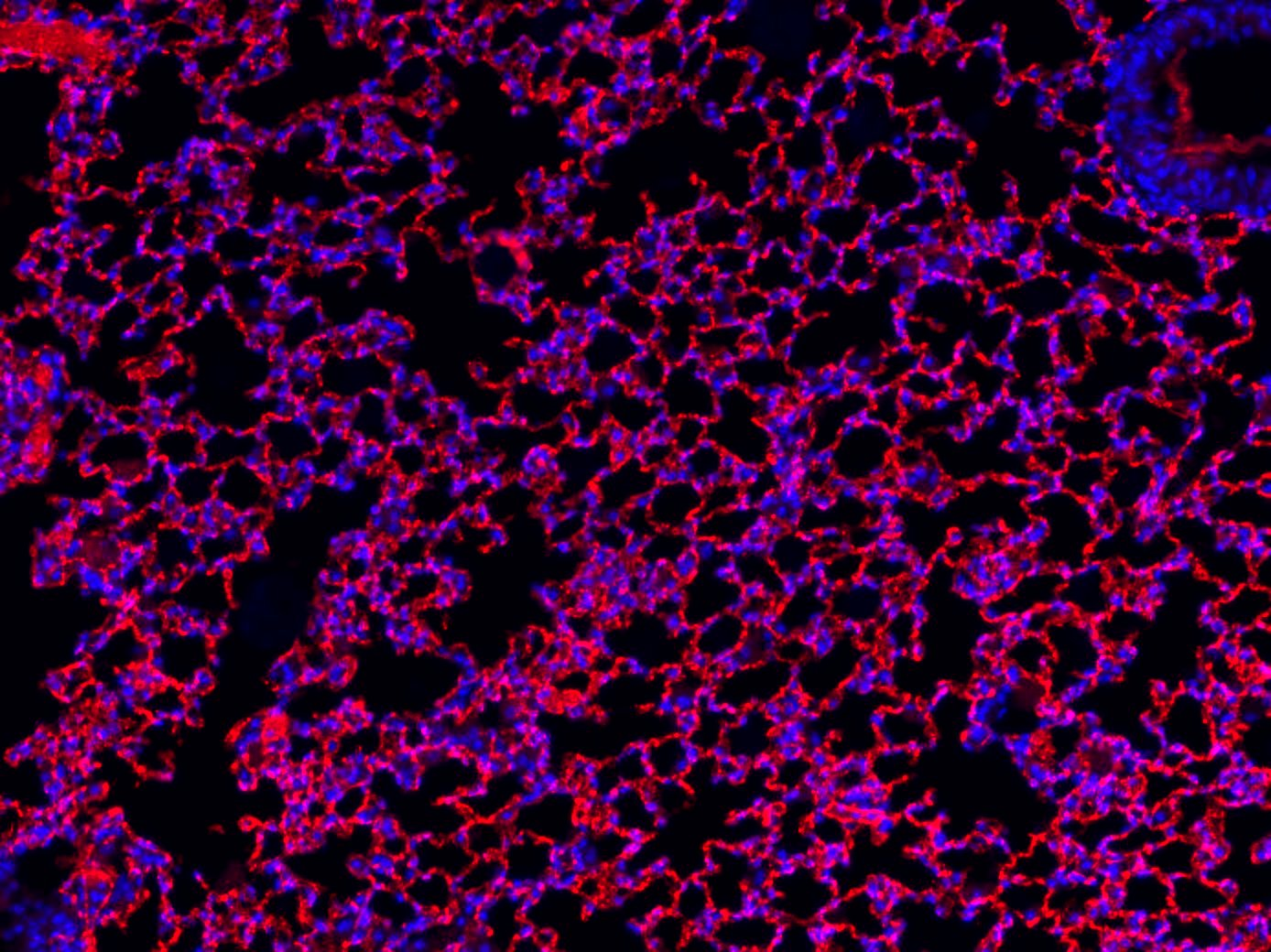

Supplement: Supplementary file 8 — Source Data Fig. 6 [file 44319_2023_41_MOESM8_ESM.zip › Source data Figure 6 /6A Image data Micr images/young p21+- merge.tif]

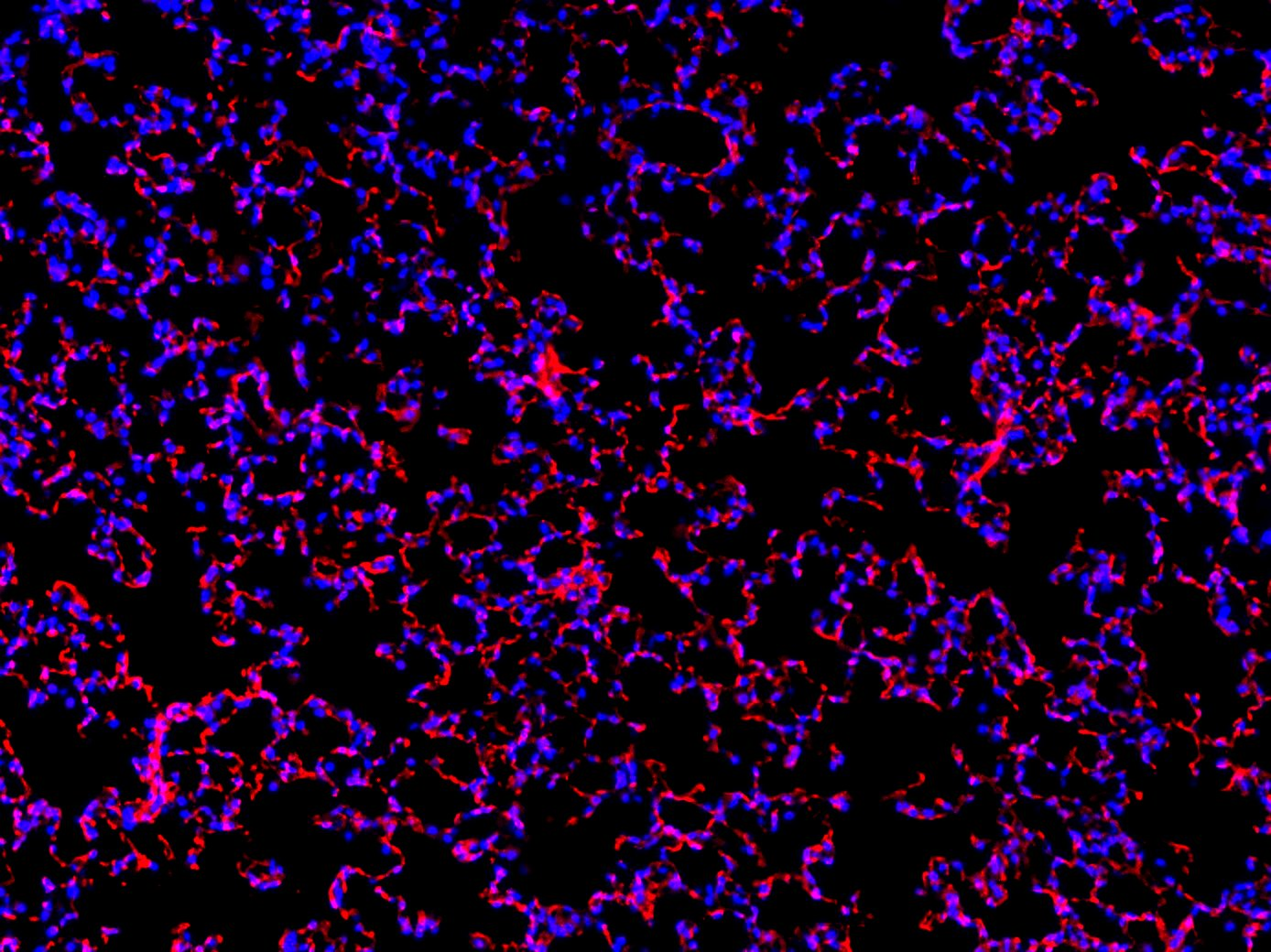

Supplement: Supplementary file 8 — Source Data Fig. 6 [file 44319_2023_41_MOESM8_ESM.zip › Source data Figure 6 /6A Image data Micr images/old p21+TERT CI merge.tif]

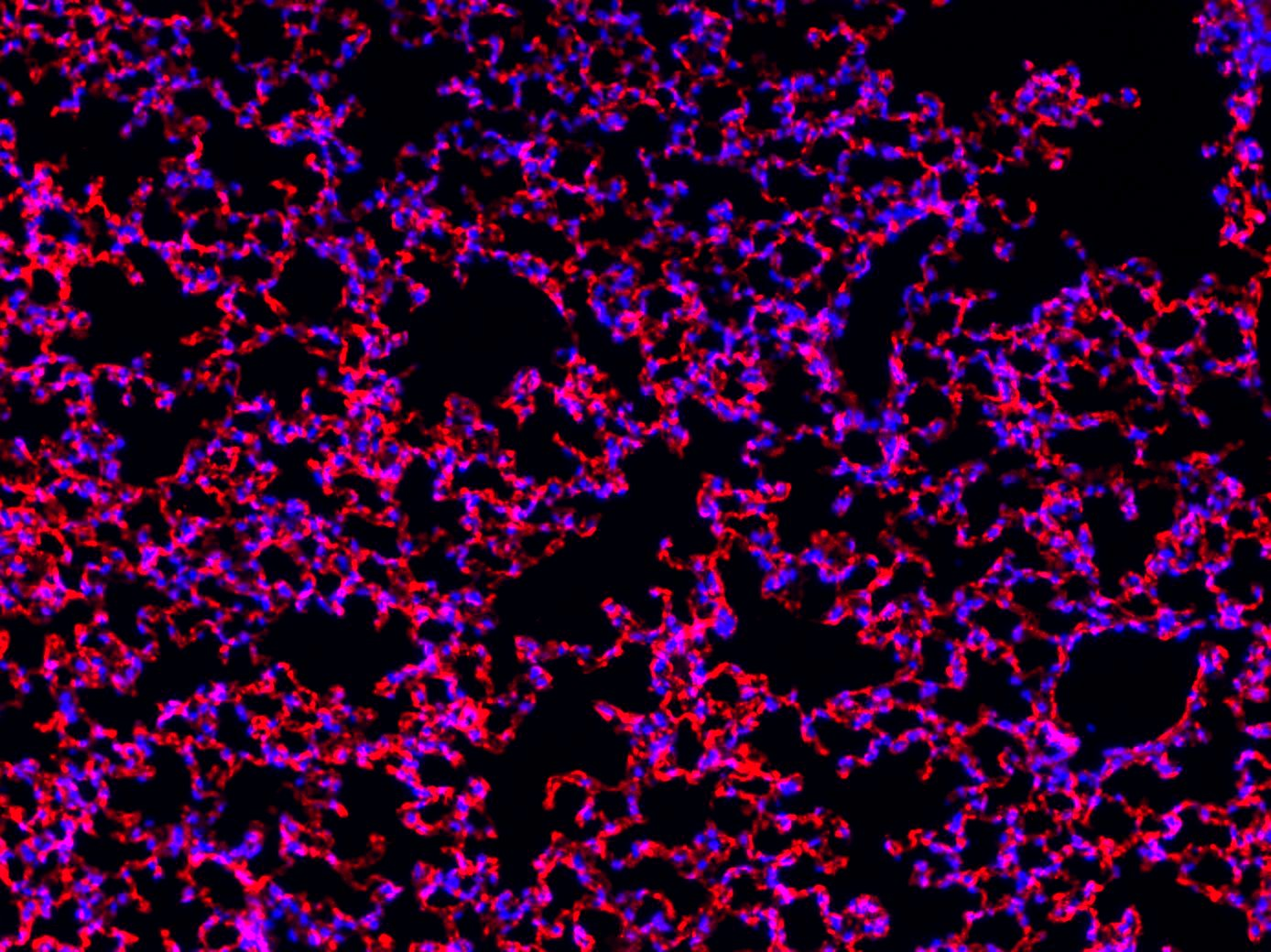

Supplement: Supplementary file 8 — Source Data Fig. 6 [file 44319_2023_41_MOESM8_ESM.zip › Source data Figure 6 /6A Image data Micr images/young p21++ merge.tif]

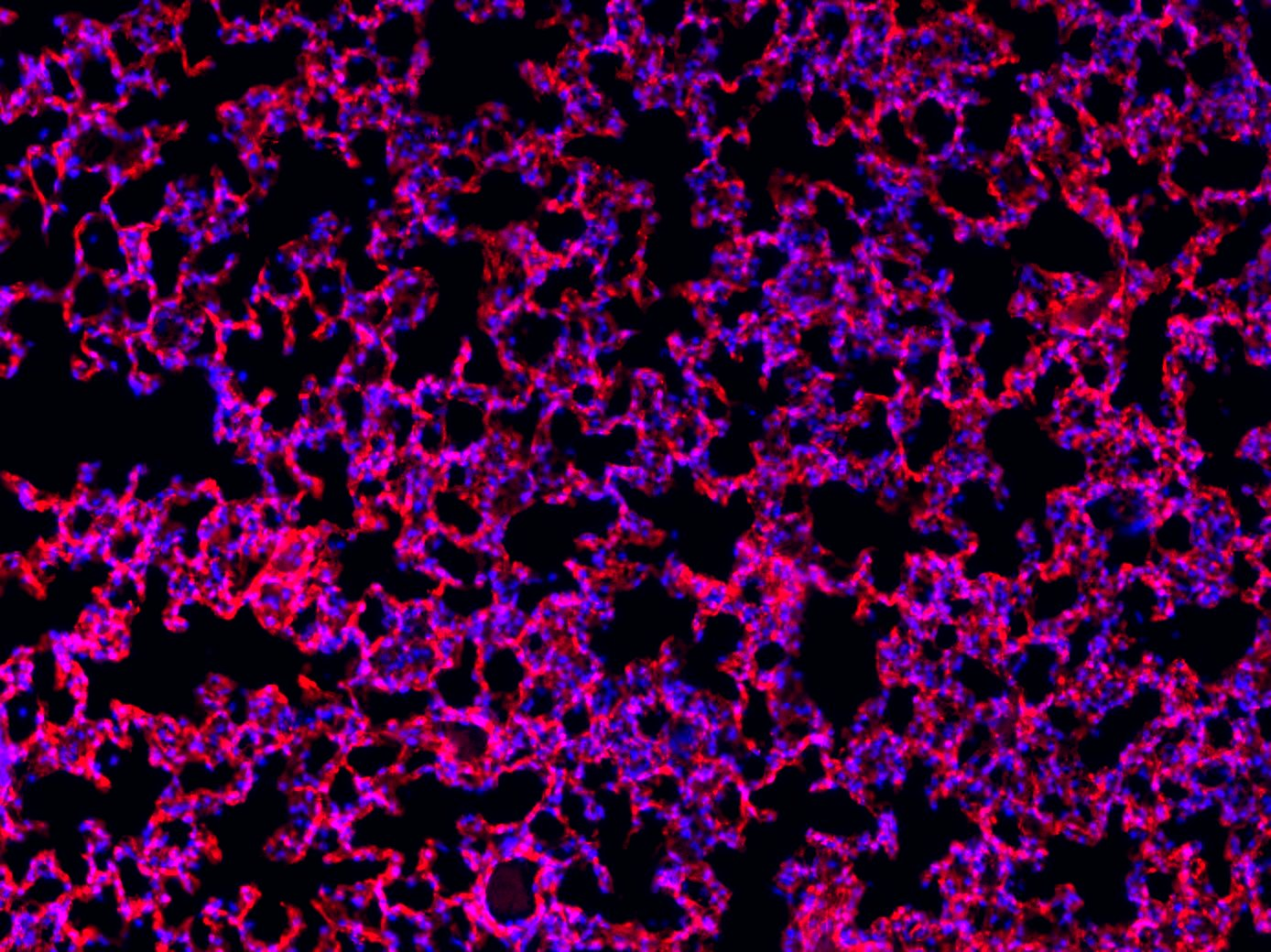

Supplement: Supplementary file 8 — Source Data Fig. 6 [file 44319_2023_41_MOESM8_ESM.zip › Source data Figure 6 /6A Image data Micr images/old p21+TERT merge.tif]

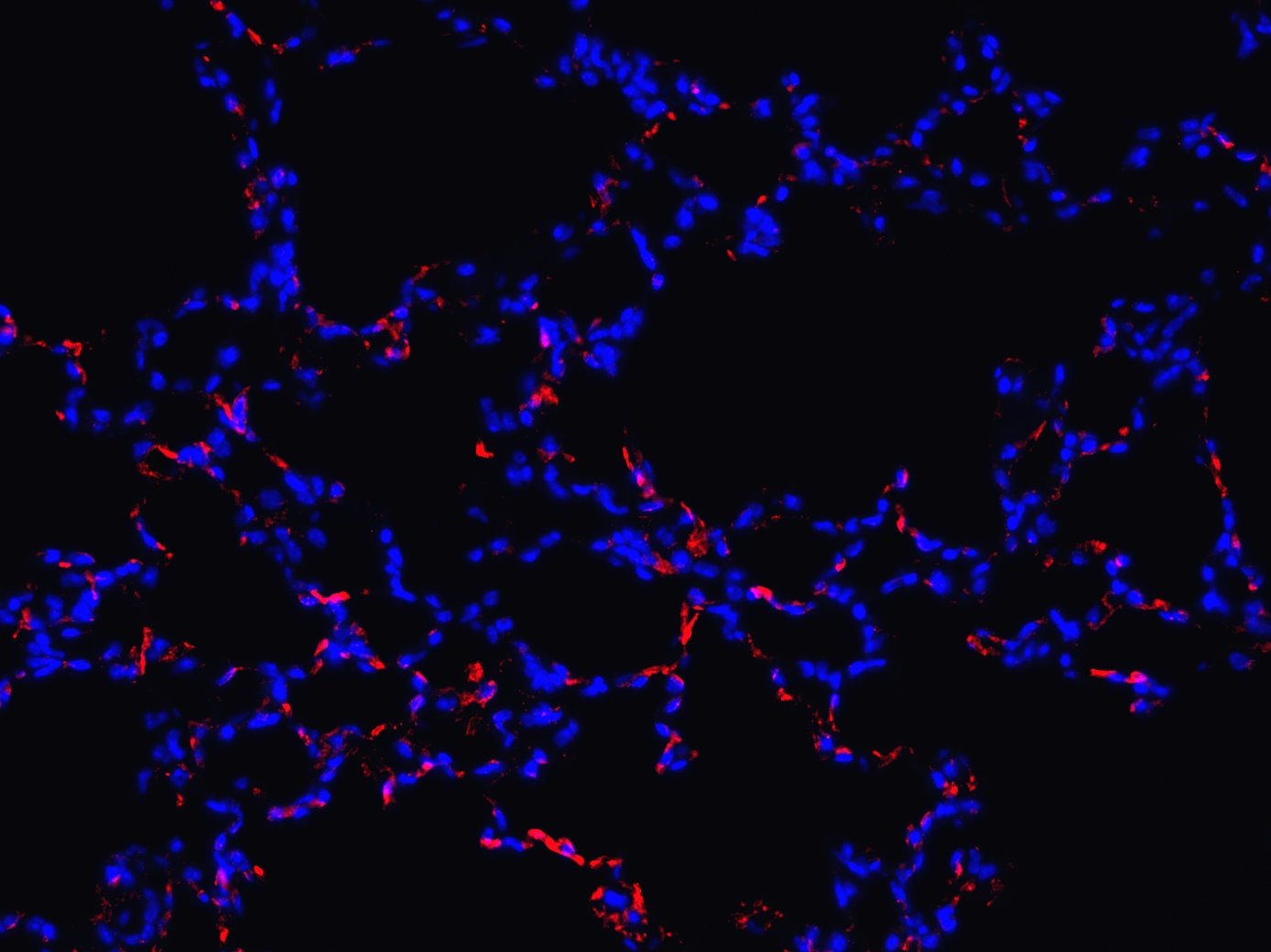

Supplement: Supplementary file 9 — Source Data Fig. 7 [file 44319_2023_41_MOESM9_ESM.zip › Source data Figure 7 /7A Image data Micr images/p21+TERT CI old.tif]

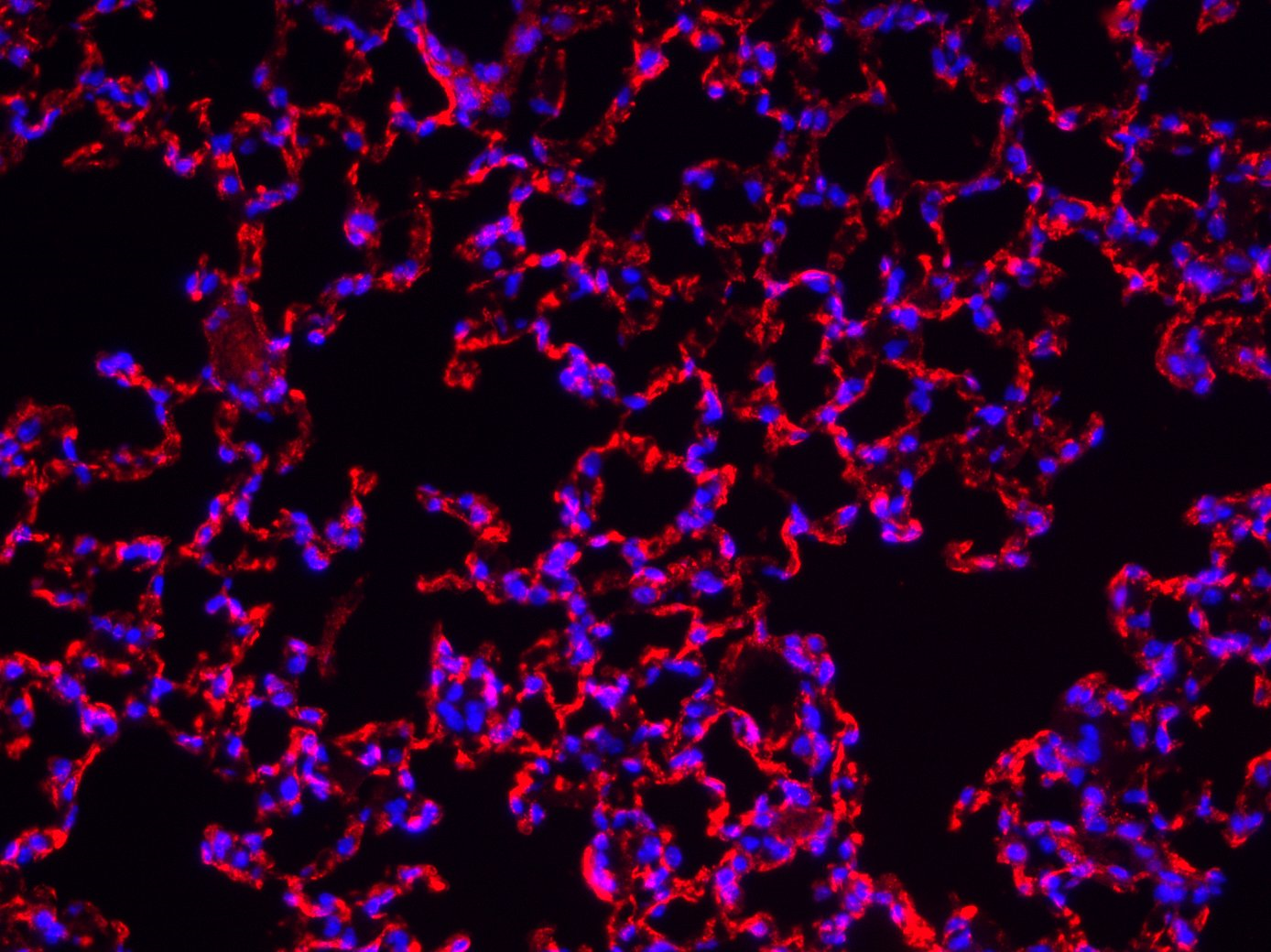

Supplement: Supplementary file 9 — Source Data Fig. 7 [file 44319_2023_41_MOESM9_ESM.zip › Source data Figure 7 /7A Image data Micr images/p21+TERT young.tif]

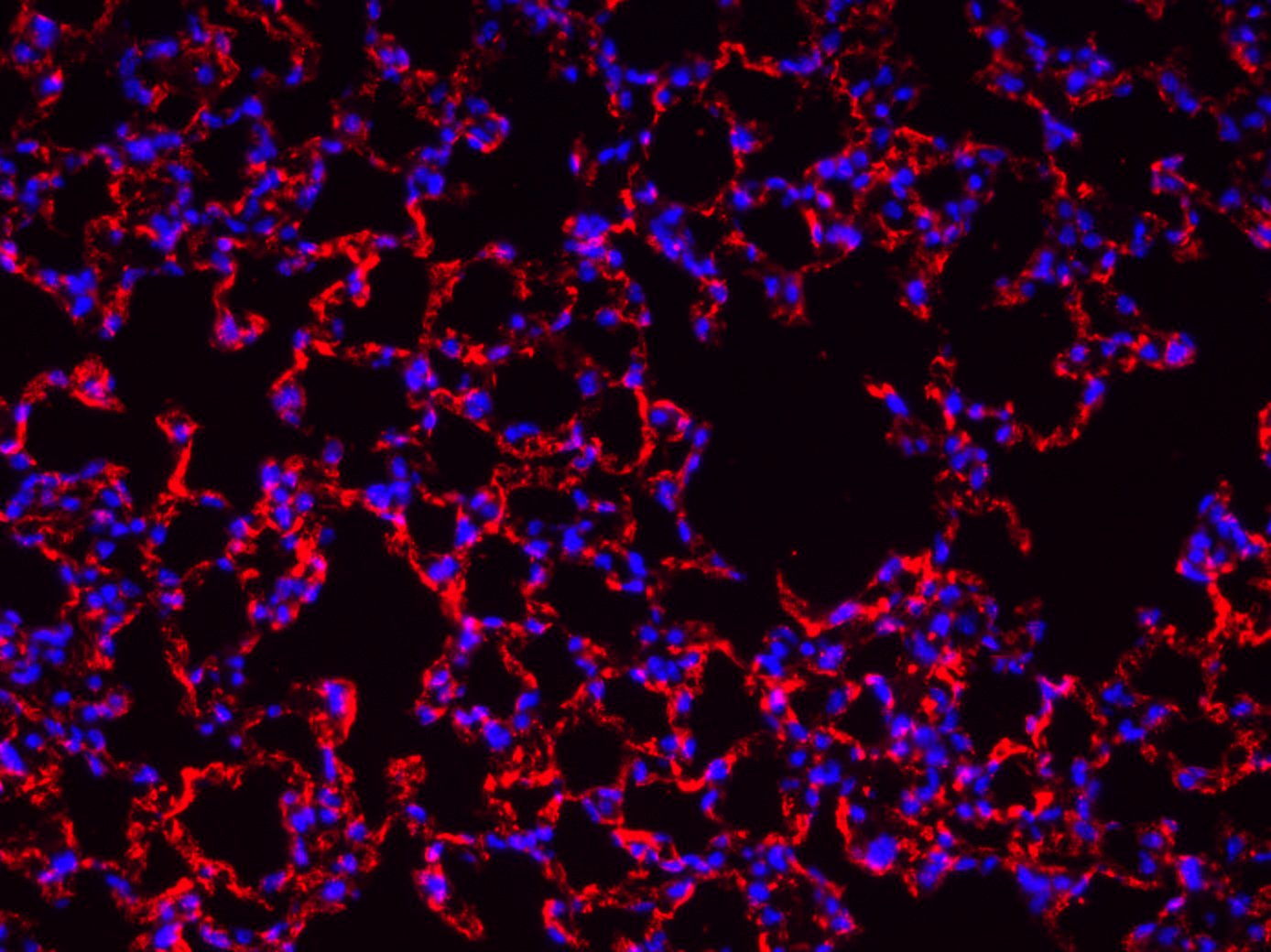

Supplement: Supplementary file 9 — Source Data Fig. 7 [file 44319_2023_41_MOESM9_ESM.zip › Source data Figure 7 /7A Image data Micr images/p21++ young .tif]

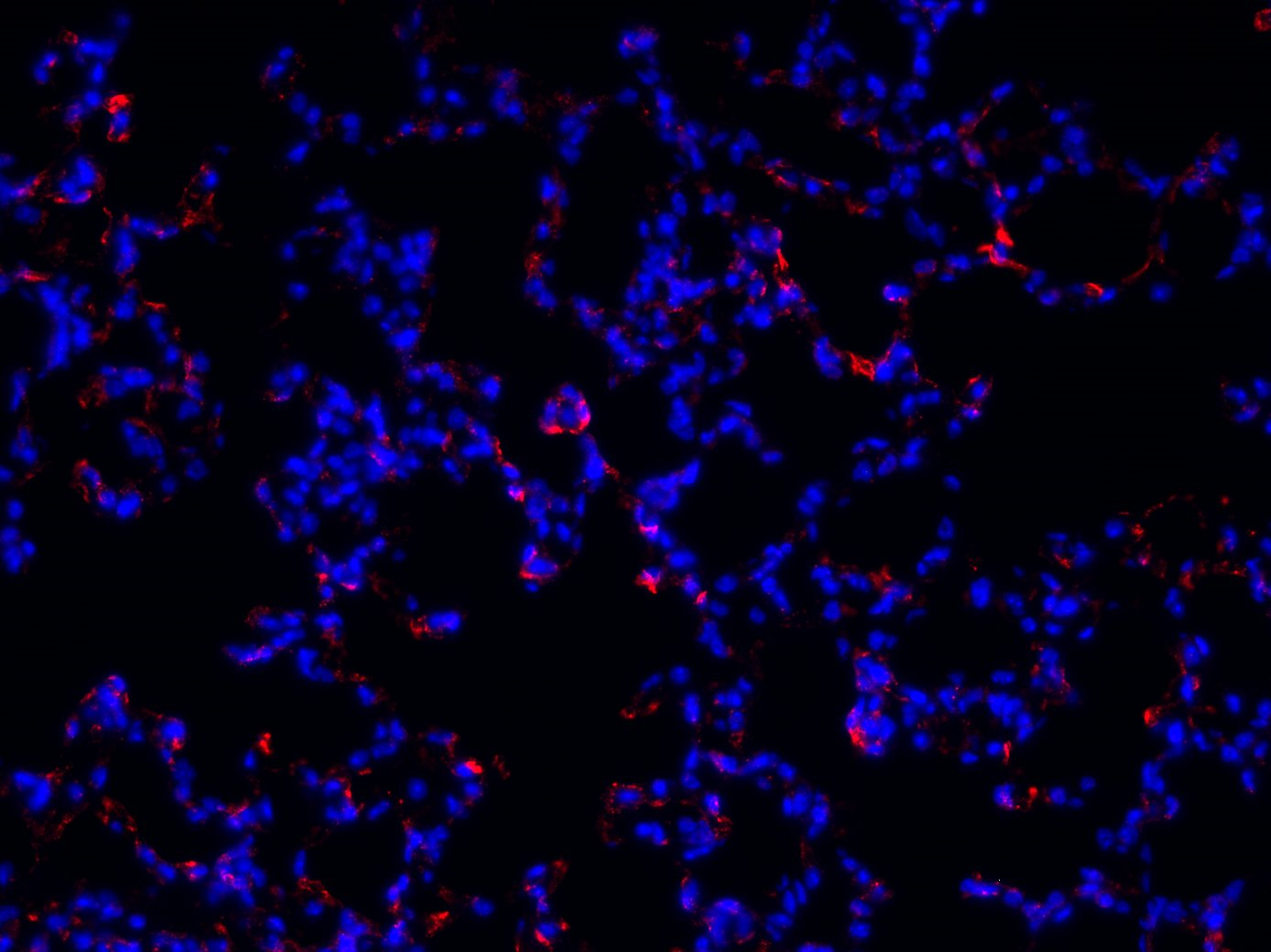

Supplement: Supplementary file 9 — Source Data Fig. 7 [file 44319_2023_41_MOESM9_ESM.zip › Source data Figure 7 /7A Image data Micr images/p21+- old.tif]

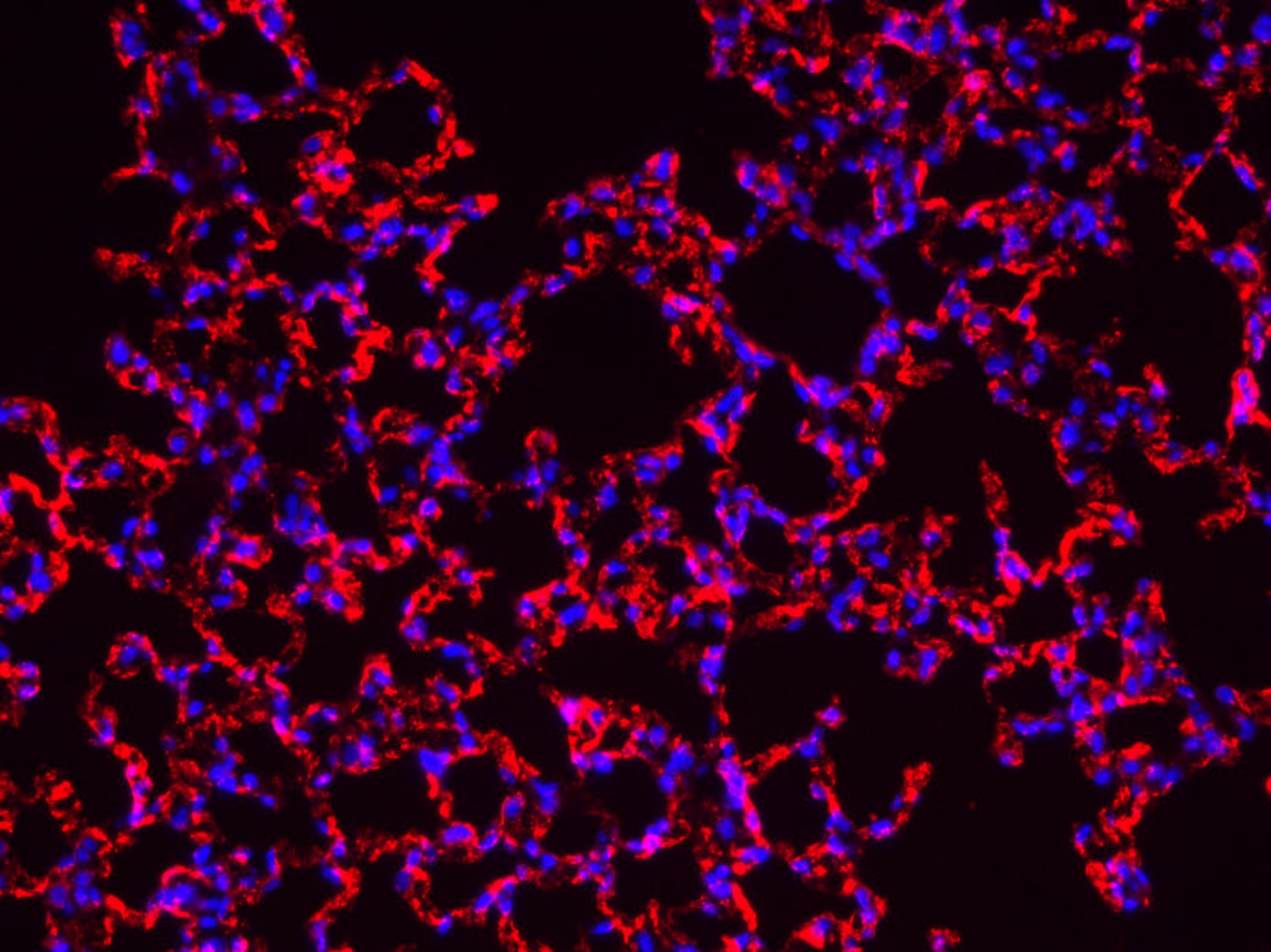

Supplement: Supplementary file 9 — Source Data Fig. 7 [file 44319_2023_41_MOESM9_ESM.zip › Source data Figure 7 /7A Image data Micr images/p21+- young.tif]

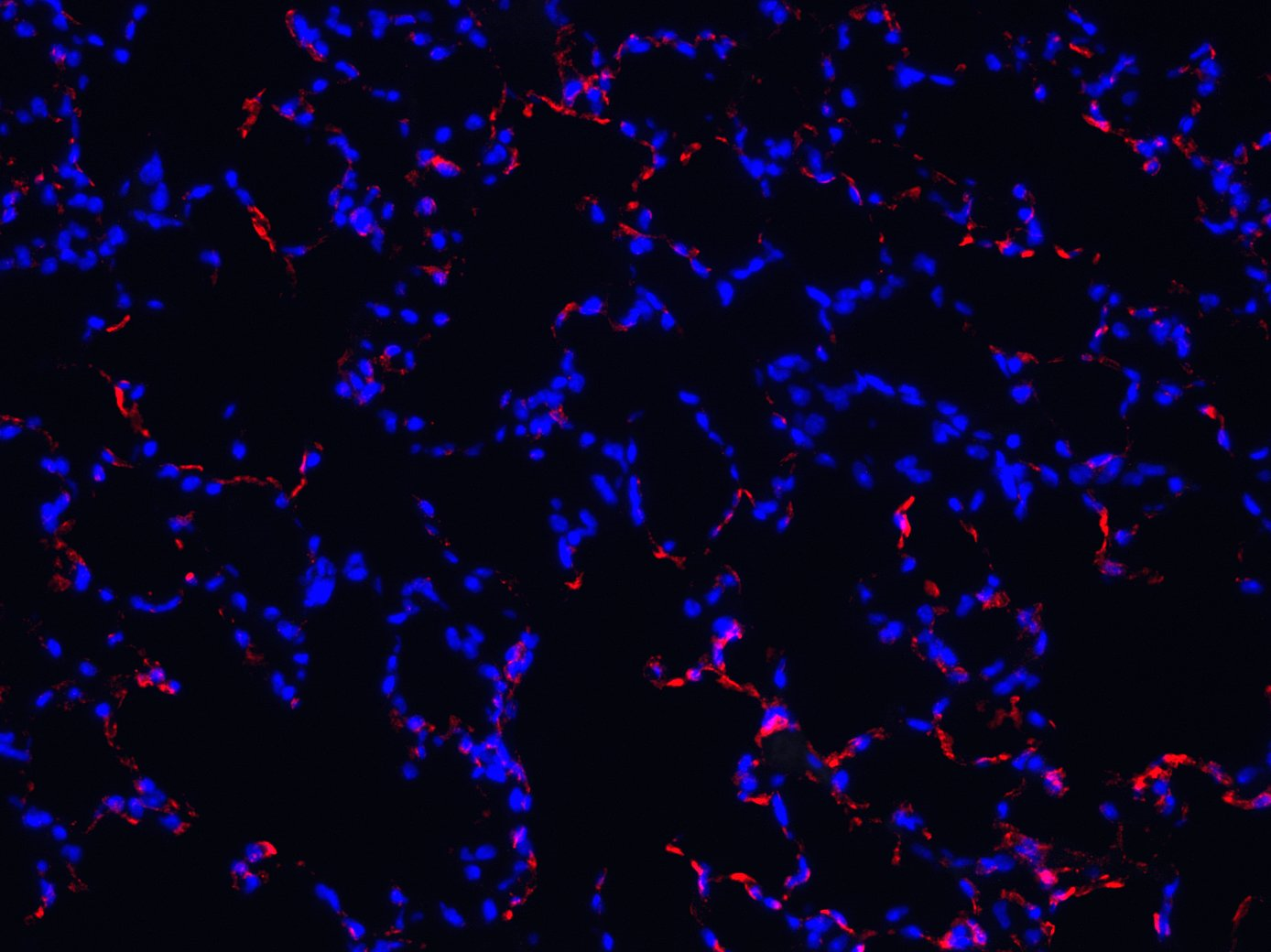

Supplement: Supplementary file 9 — Source Data Fig. 7 [file 44319_2023_41_MOESM9_ESM.zip › Source data Figure 7 /7A Image data Micr images/p21++ old.tif]

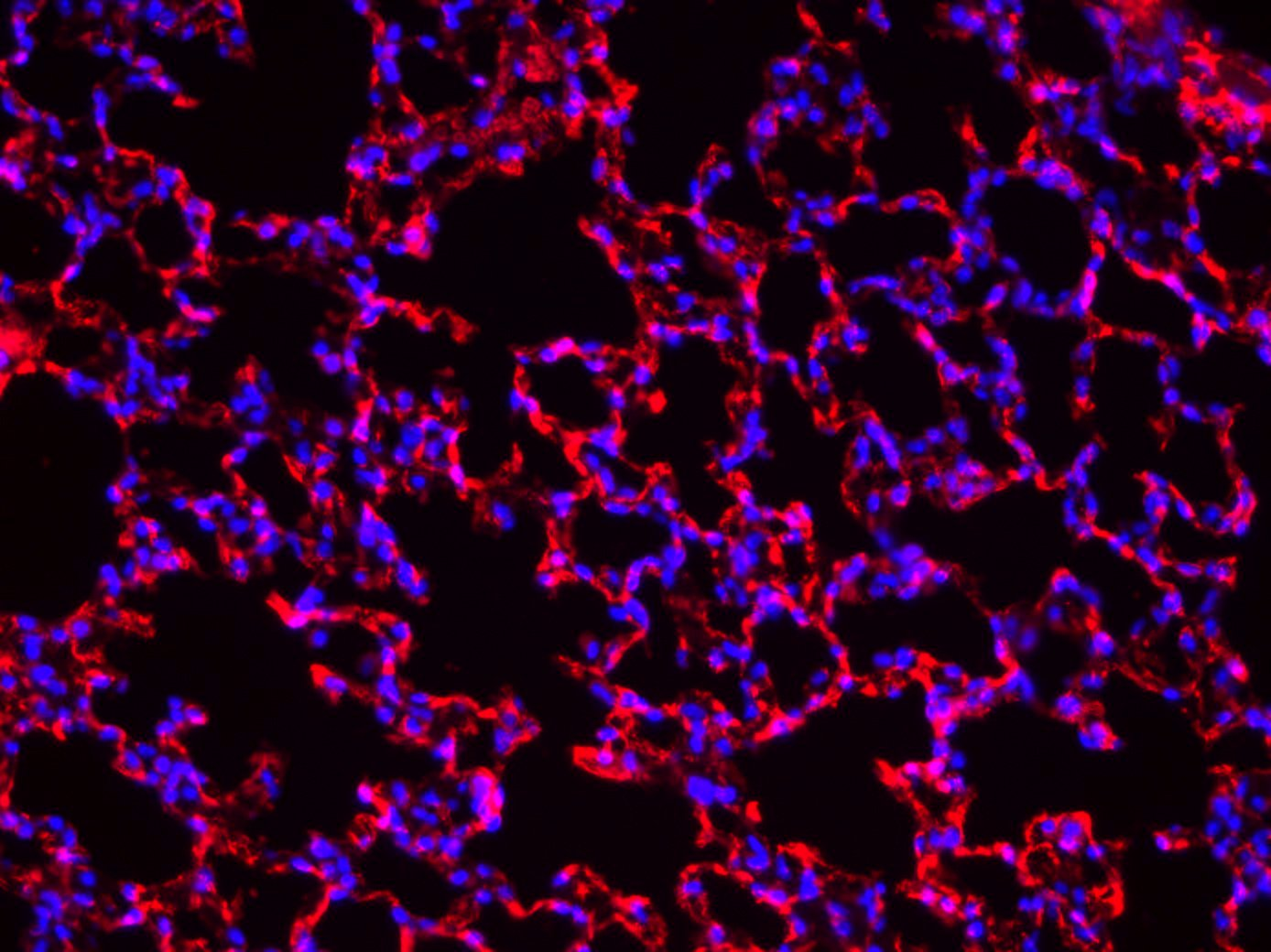

Supplement: Supplementary file 9 — Source Data Fig. 7 [file 44319_2023_41_MOESM9_ESM.zip › Source data Figure 7 /7A Image data Micr images/p21+TERT old.tif]

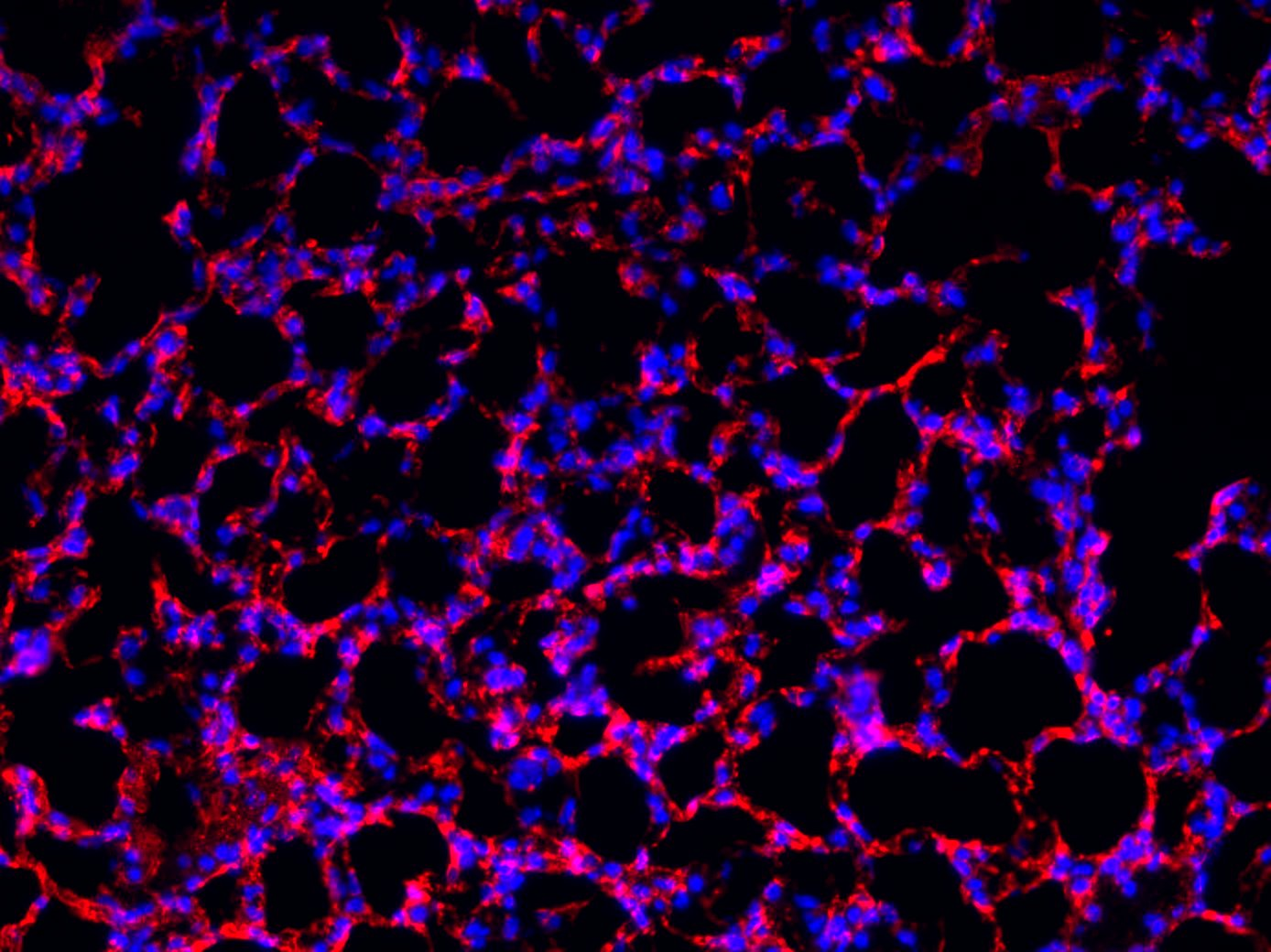

Supplement: Supplementary file 9 — Source Data Fig. 7 [file 44319_2023_41_MOESM9_ESM.zip › Source data Figure 7 /7A Image data Micr images/p21+TERT CI young.tif]

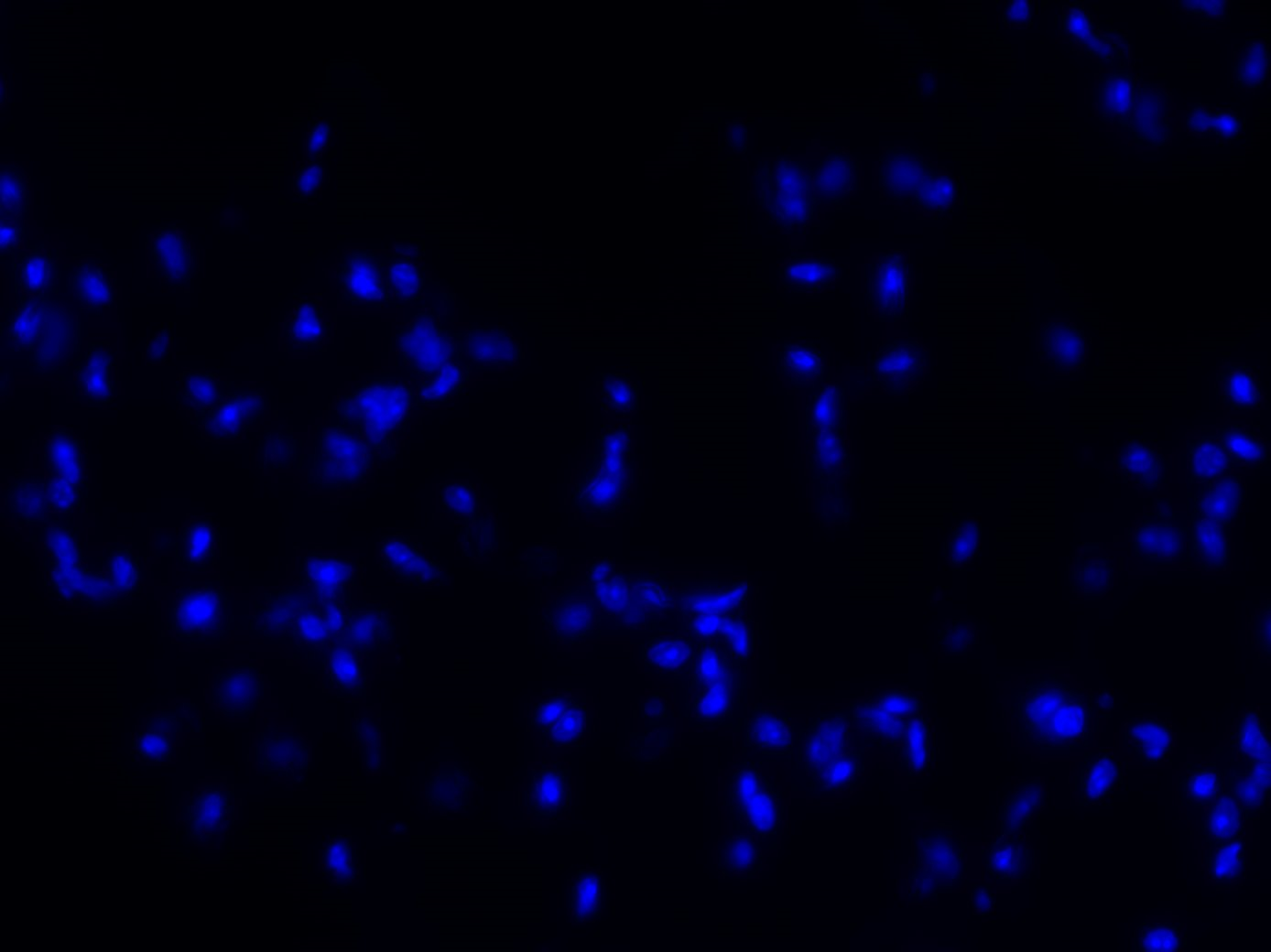

Supplement: Supplementary file 10 — Source Data Fig. 8 [file 44319_2023_41_MOESM10_ESM.zip › Source data Figure 8/8C Image data Micr images/p21+- Dapi.tif]

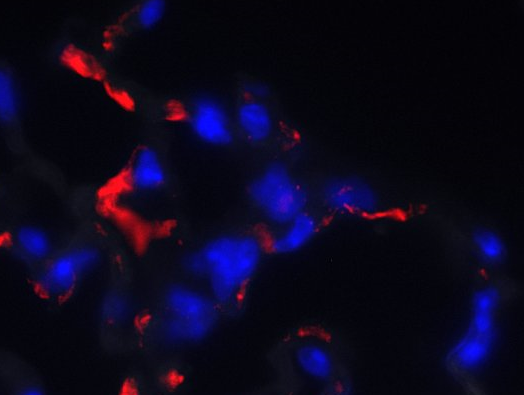

Supplement: Supplementary file 10 — Source Data Fig. 8 [file 44319_2023_41_MOESM10_ESM.zip › Source data Figure 8/8C Image data Micr images/p21+- (Dapi + CD34) ZOOM.tif]

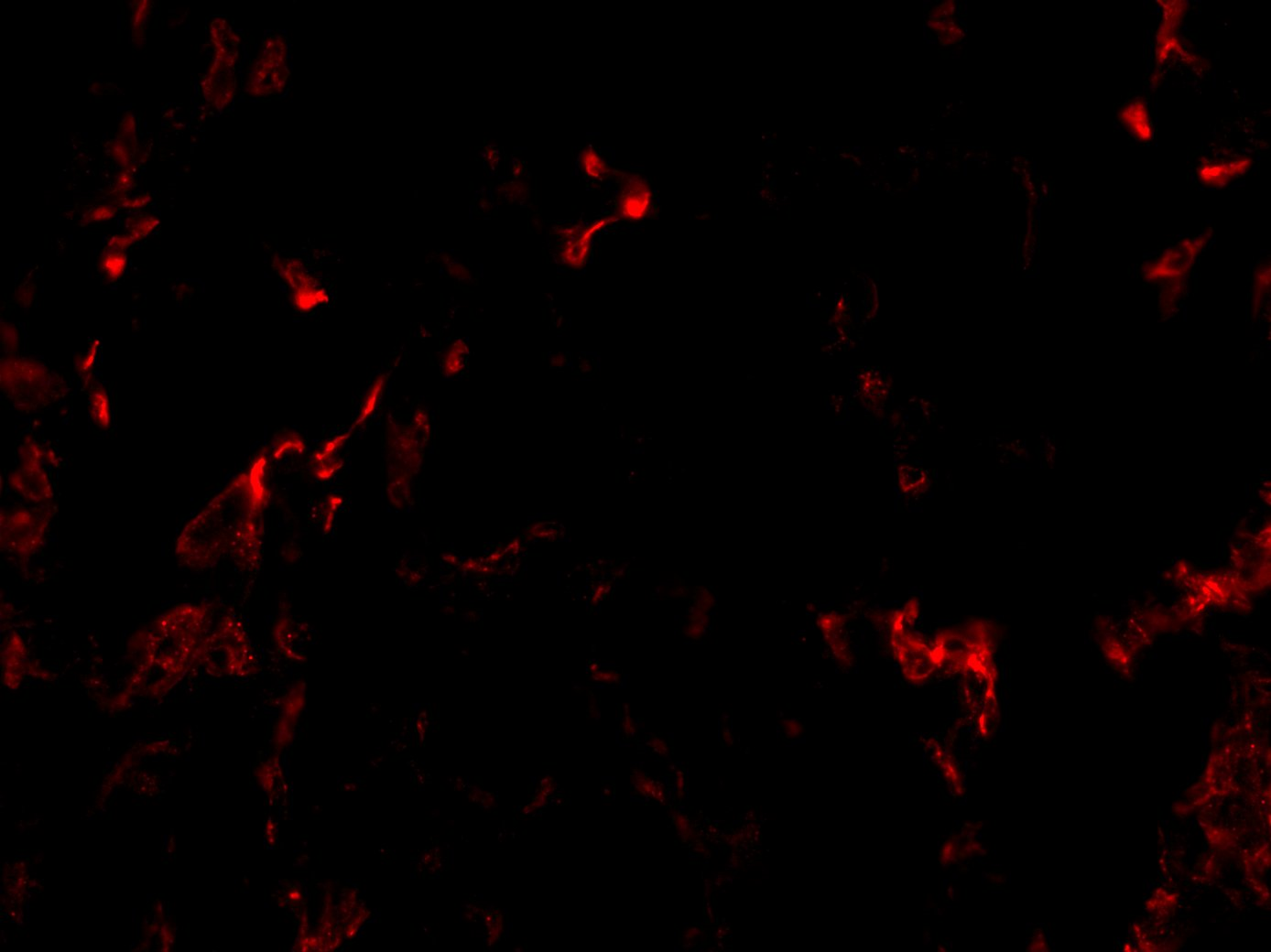

Supplement: Supplementary file 10 — Source Data Fig. 8 [file 44319_2023_41_MOESM10_ESM.zip › Source data Figure 8/8C Image data Micr images/p21++ CD34.tif]

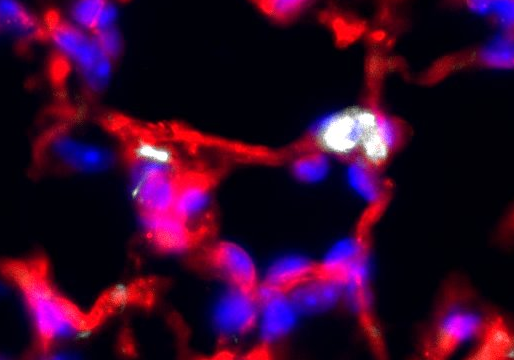

Supplement: Supplementary file 10 — Source Data Fig. 8 [file 44319_2023_41_MOESM10_ESM.zip › Source data Figure 8/8C Image data Micr images/p21+TERT (Dapi + CD34) ZOOM.tif]

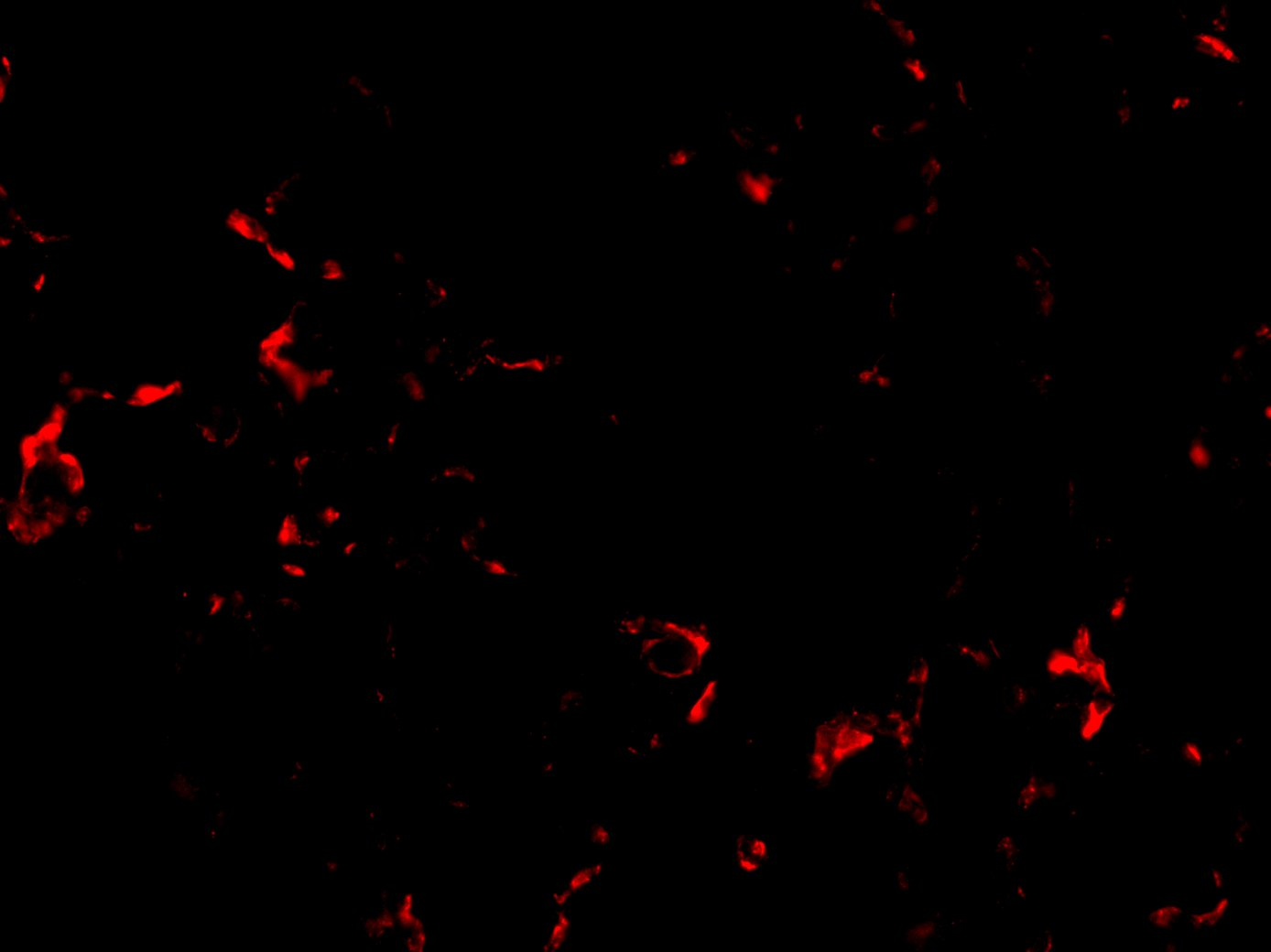

Supplement: Supplementary file 10 — Source Data Fig. 8 [file 44319_2023_41_MOESM10_ESM.zip › Source data Figure 8/8C Image data Micr images/p21+- CD34.tif]

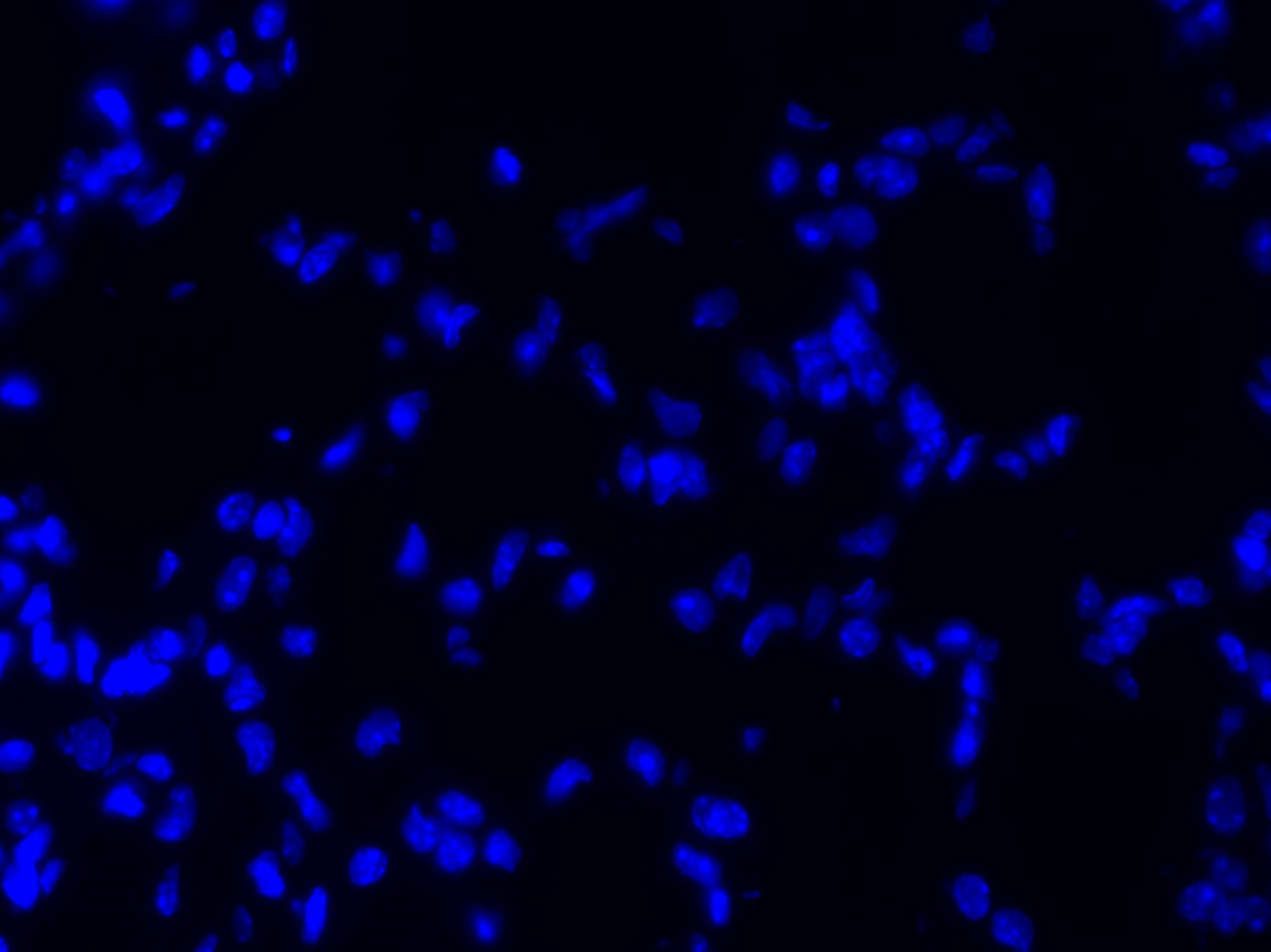

Supplement: Supplementary file 10 — Source Data Fig. 8 [file 44319_2023_41_MOESM10_ESM.zip › Source data Figure 8/8C Image data Micr images/p21++ Dapi.tif]

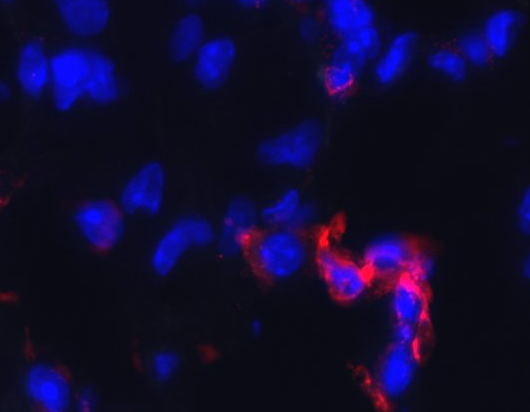

Supplement: Supplementary file 10 — Source Data Fig. 8 [file 44319_2023_41_MOESM10_ESM.zip › Source data Figure 8/8C Image data Micr images/p21++ (Dapi + CD34) ZOOM.tif]

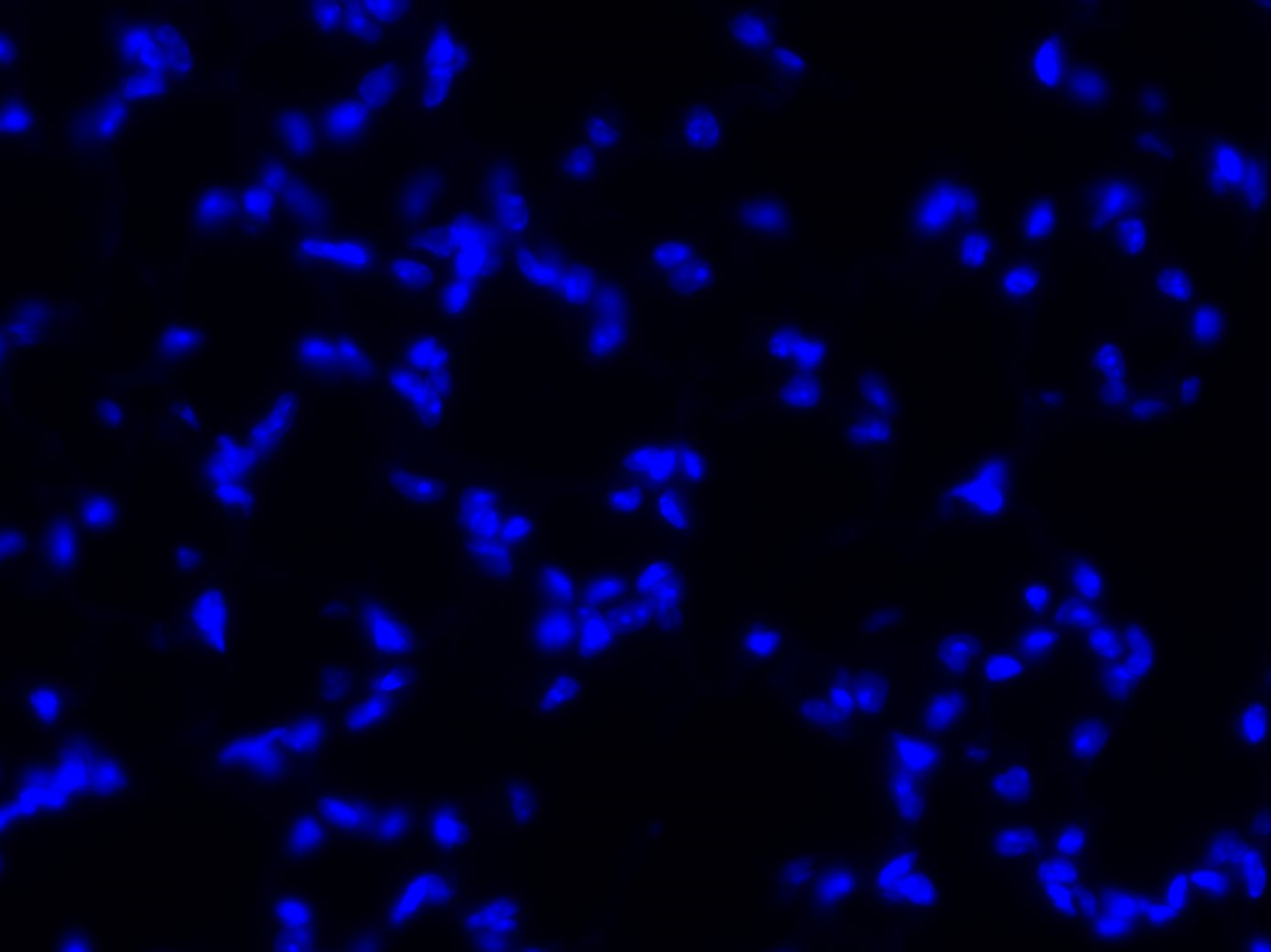

Supplement: Supplementary file 10 — Source Data Fig. 8 [file 44319_2023_41_MOESM10_ESM.zip › Source data Figure 8/8C Image data Micr images/p21+TERT Dapi.tif]

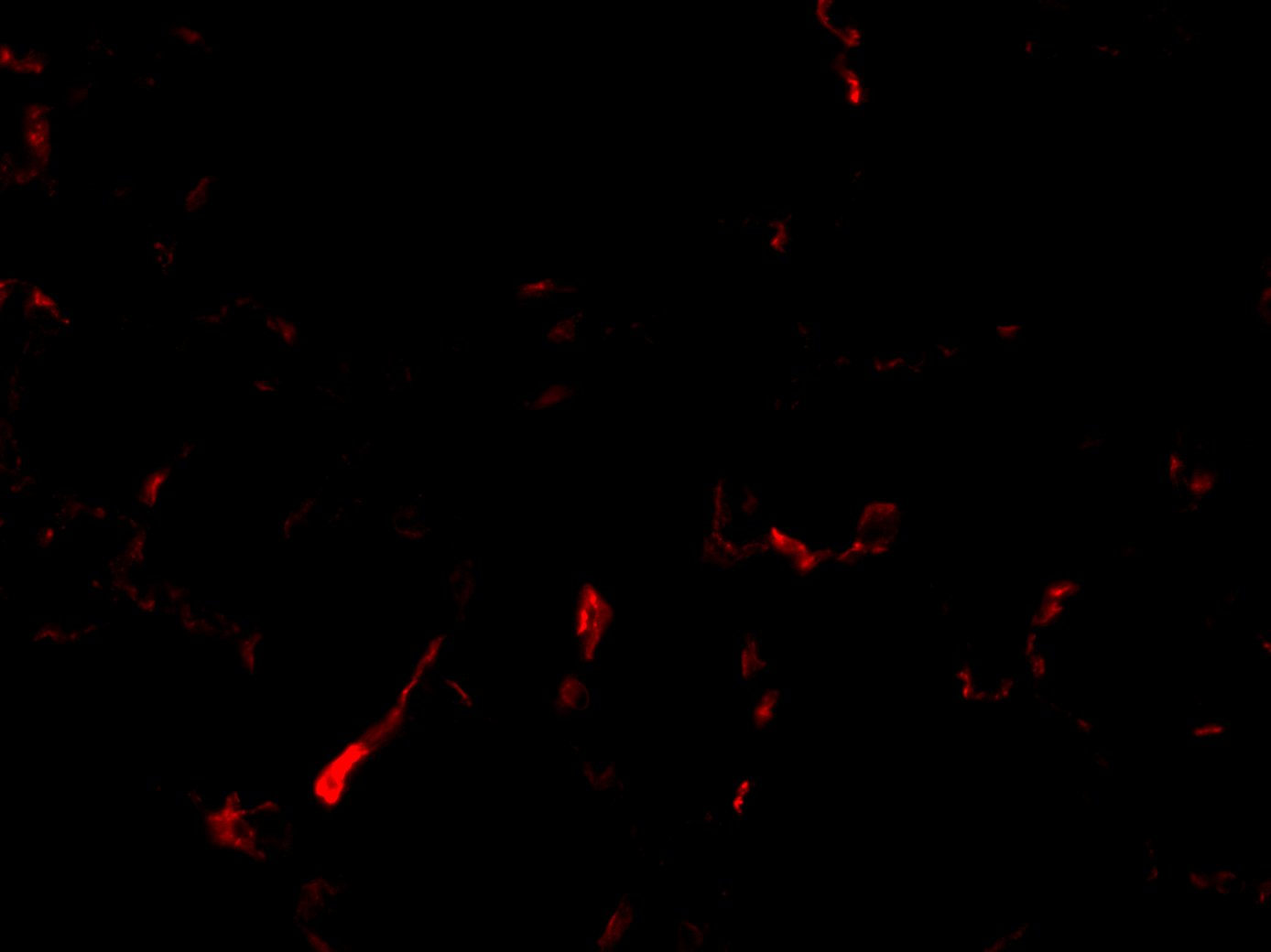

Supplement: Supplementary file 10 — Source Data Fig. 8 [file 44319_2023_41_MOESM10_ESM.zip › Source data Figure 8/8C Image data Micr images/p21+TERT CI CD34.tif]

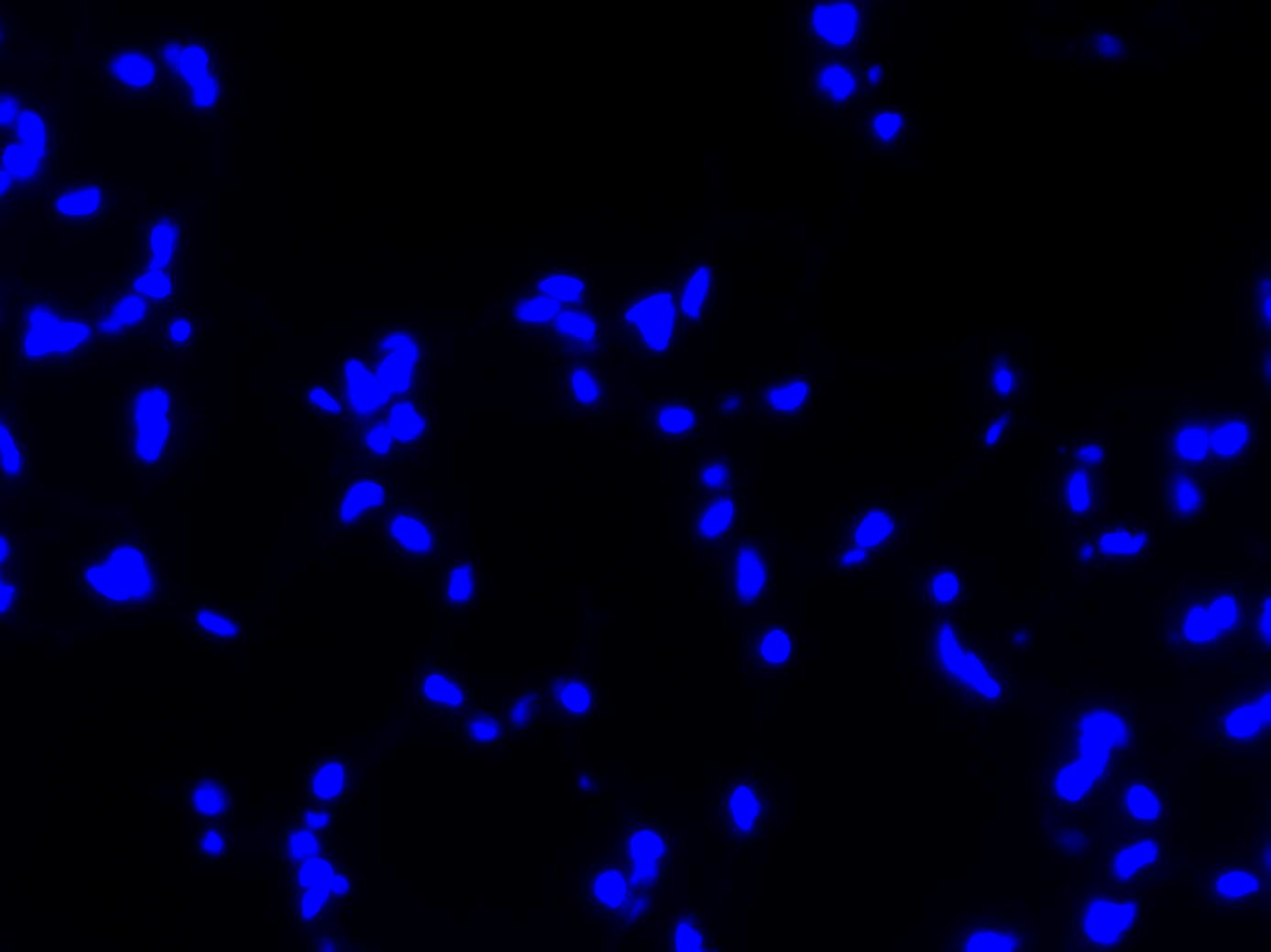

Supplement: Supplementary file 10 — Source Data Fig. 8 [file 44319_2023_41_MOESM10_ESM.zip › Source data Figure 8/8C Image data Micr images/p21+TERT CI Dapi.tif]

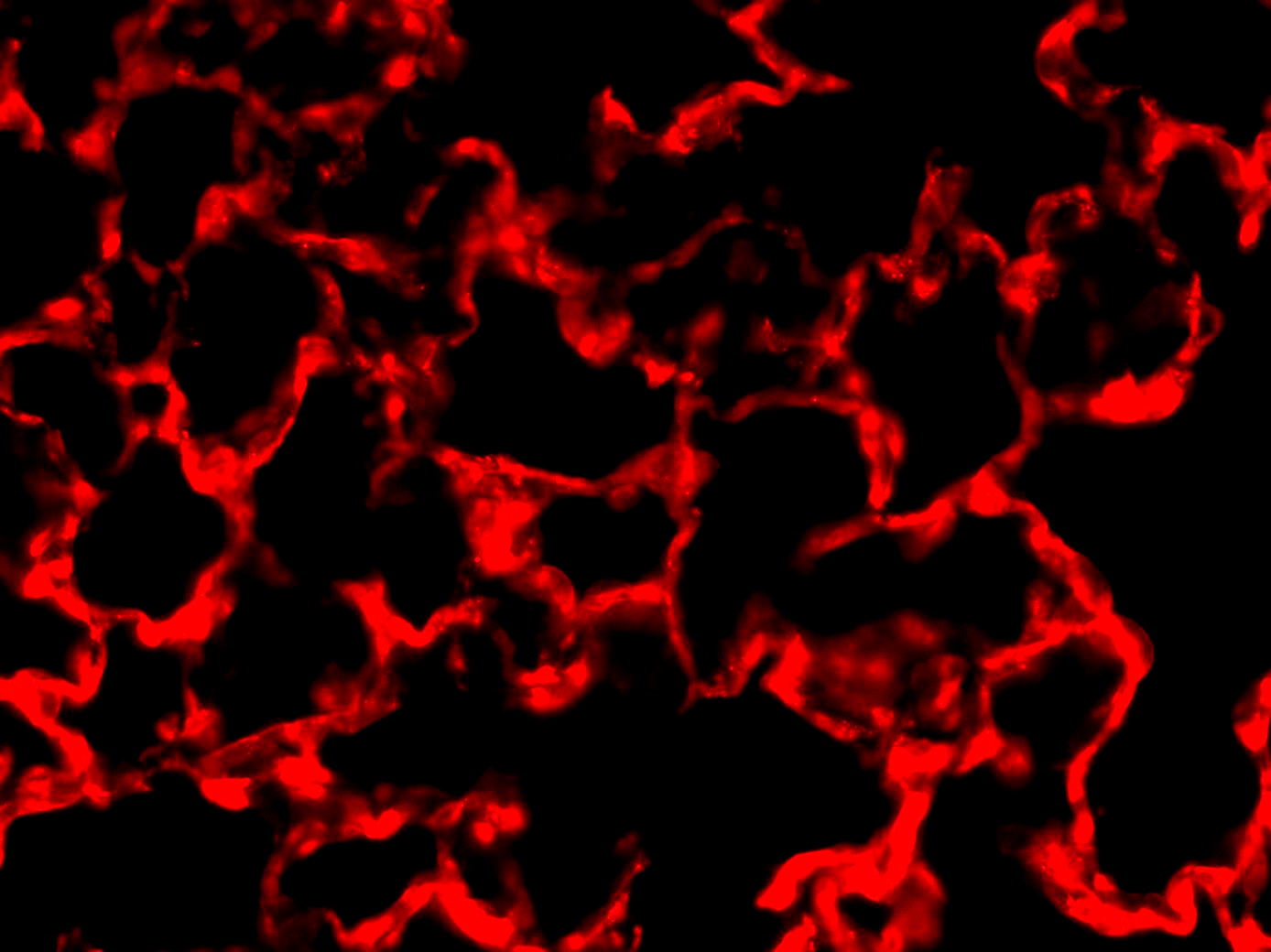

Supplement: Supplementary file 10 — Source Data Fig. 8 [file 44319_2023_41_MOESM10_ESM.zip › Source data Figure 8/8C Image data Micr images/p21+TERT CD34.tif]

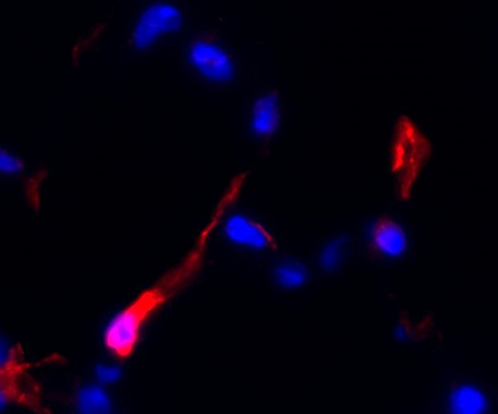

Supplement: Supplementary file 10 — Source Data Fig. 8 [file 44319_2023_41_MOESM10_ESM.zip › Source data Figure 8/8C Image data Micr images/p21+TERT CI (Dapi + CD34) ZOOM.tif]

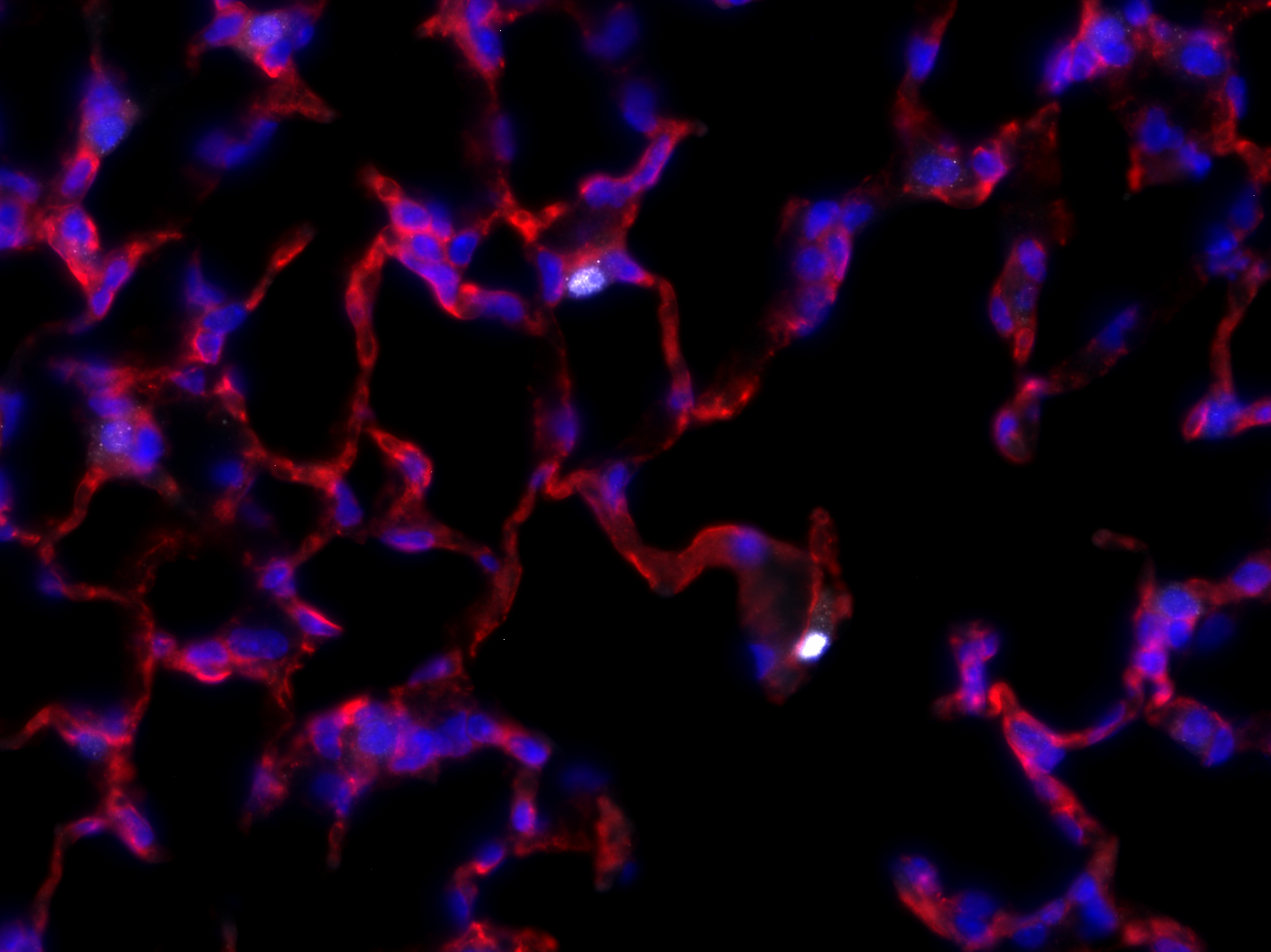

Supplement: Supplementary file 10 — Source Data Fig. 8 [file 44319_2023_41_MOESM10_ESM.zip › Source data Figure 8/8D Image data Micr images/young/p21+TERT CI (p21 + DAPI + CD34).tif]

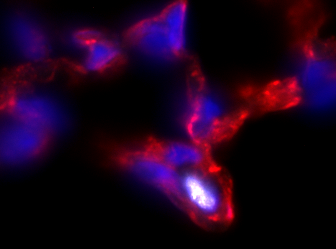

Supplement: Supplementary file 10 — Source Data Fig. 8 [file 44319_2023_41_MOESM10_ESM.zip › Source data Figure 8/8D Image data Micr images/young/p21+TERT (p21 + DAPI + CD34) ZOOM.tif]

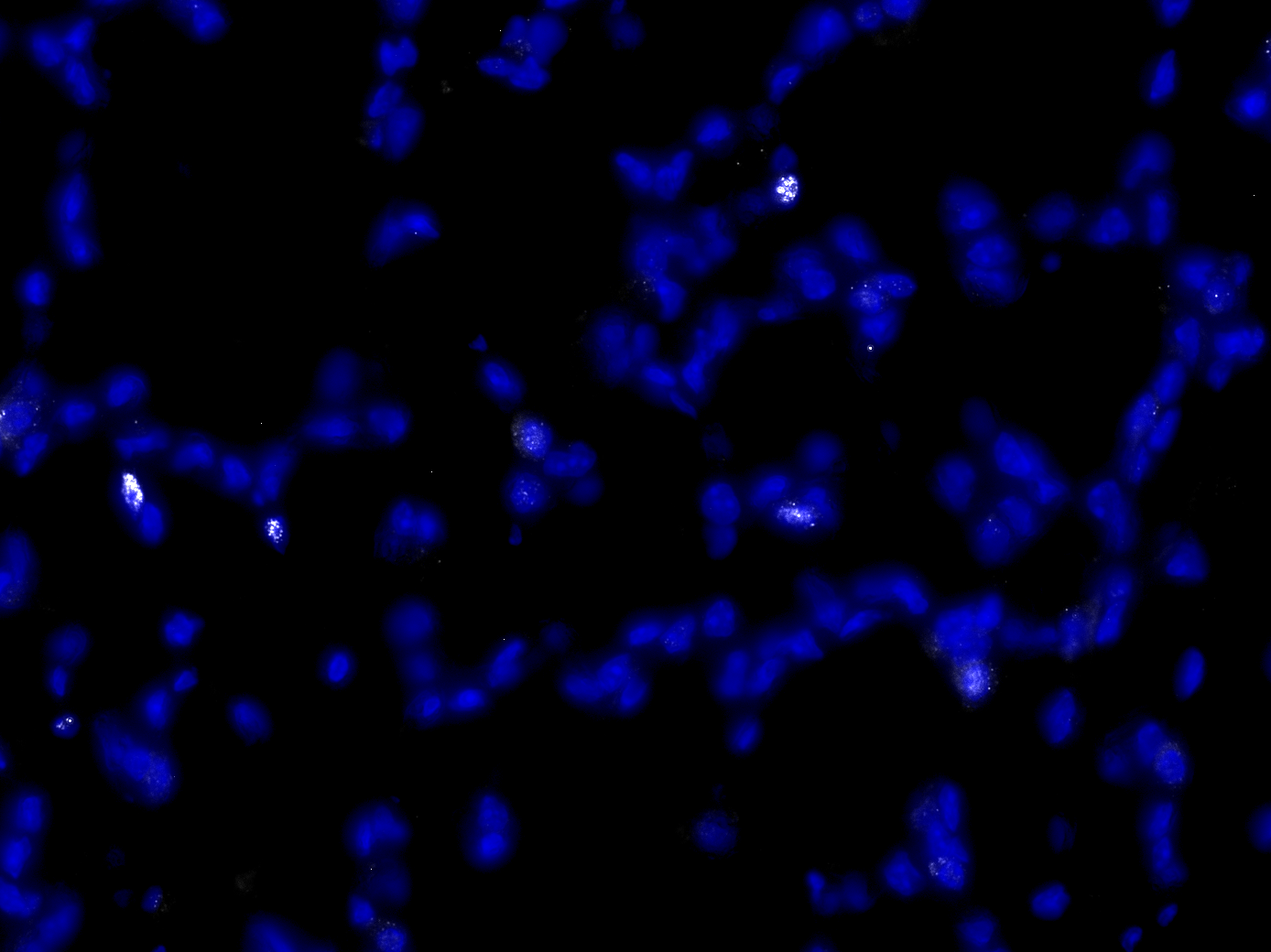

Supplement: Supplementary file 10 — Source Data Fig. 8 [file 44319_2023_41_MOESM10_ESM.zip › Source data Figure 8/8D Image data Micr images/young/p21+- (p21 + DAPI).tif]

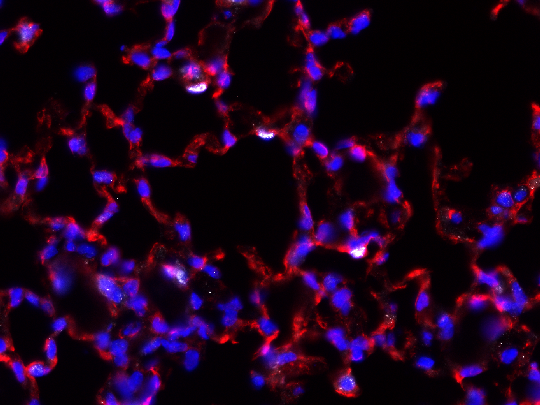

Supplement: Supplementary file 10 — Source Data Fig. 8 [file 44319_2023_41_MOESM10_ESM.zip › Source data Figure 8/8D Image data Micr images/young/p21++ (p21 + DAPI + CD34).tif]

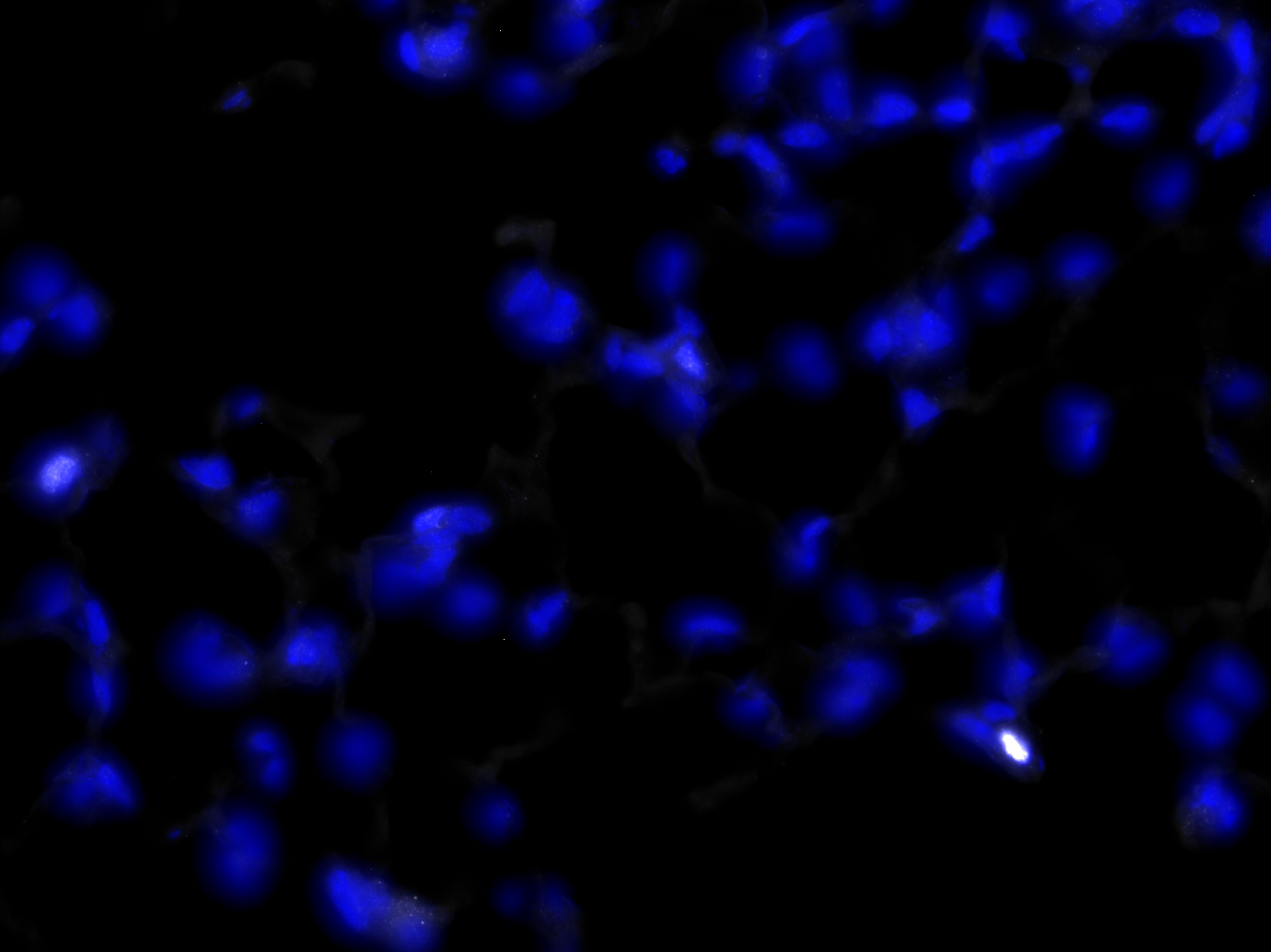

Supplement: Supplementary file 10 — Source Data Fig. 8 [file 44319_2023_41_MOESM10_ESM.zip › Source data Figure 8/8D Image data Micr images/young/p21+TERT (p21 + DAPI).tif]

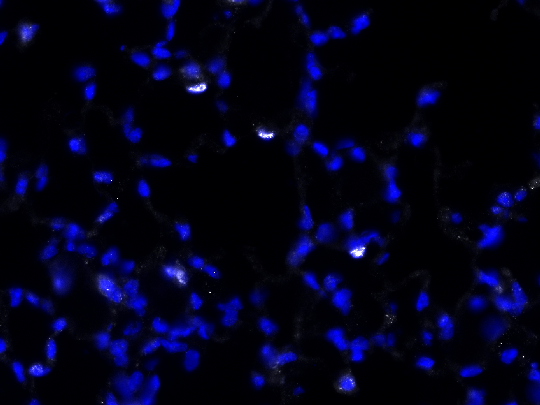

Supplement: Supplementary file 10 — Source Data Fig. 8 [file 44319_2023_41_MOESM10_ESM.zip › Source data Figure 8/8D Image data Micr images/young/p21++ (p21 + DAPI).tif]

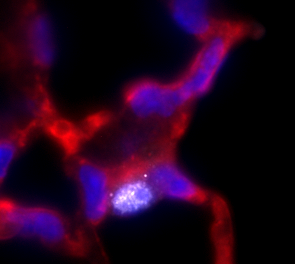

Supplement: Supplementary file 10 — Source Data Fig. 8 [file 44319_2023_41_MOESM10_ESM.zip › Source data Figure 8/8D Image data Micr images/young/p21+TERT CI (p21 + DAPI + CD34) ZOOM.tif]

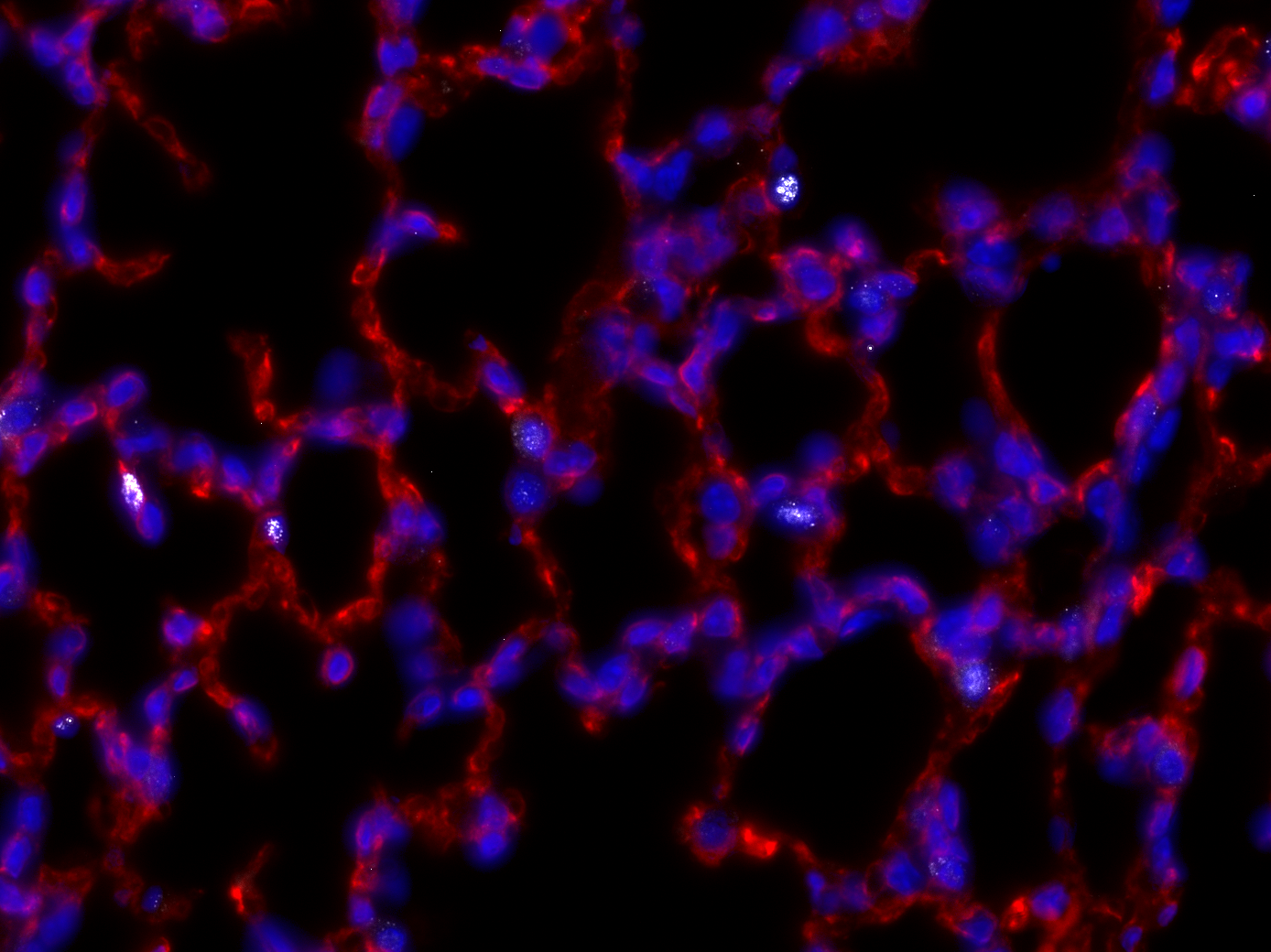

Supplement: Supplementary file 10 — Source Data Fig. 8 [file 44319_2023_41_MOESM10_ESM.zip › Source data Figure 8/8D Image data Micr images/young/p21+- (p21 + DAPI + CD34).tif]

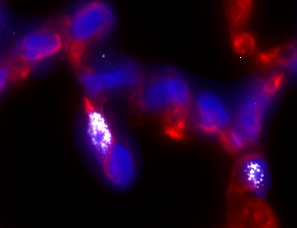

Supplement: Supplementary file 10 — Source Data Fig. 8 [file 44319_2023_41_MOESM10_ESM.zip › Source data Figure 8/8D Image data Micr images/young/p21+- (p21 + DAPI + CD34) ZOOM.tif]

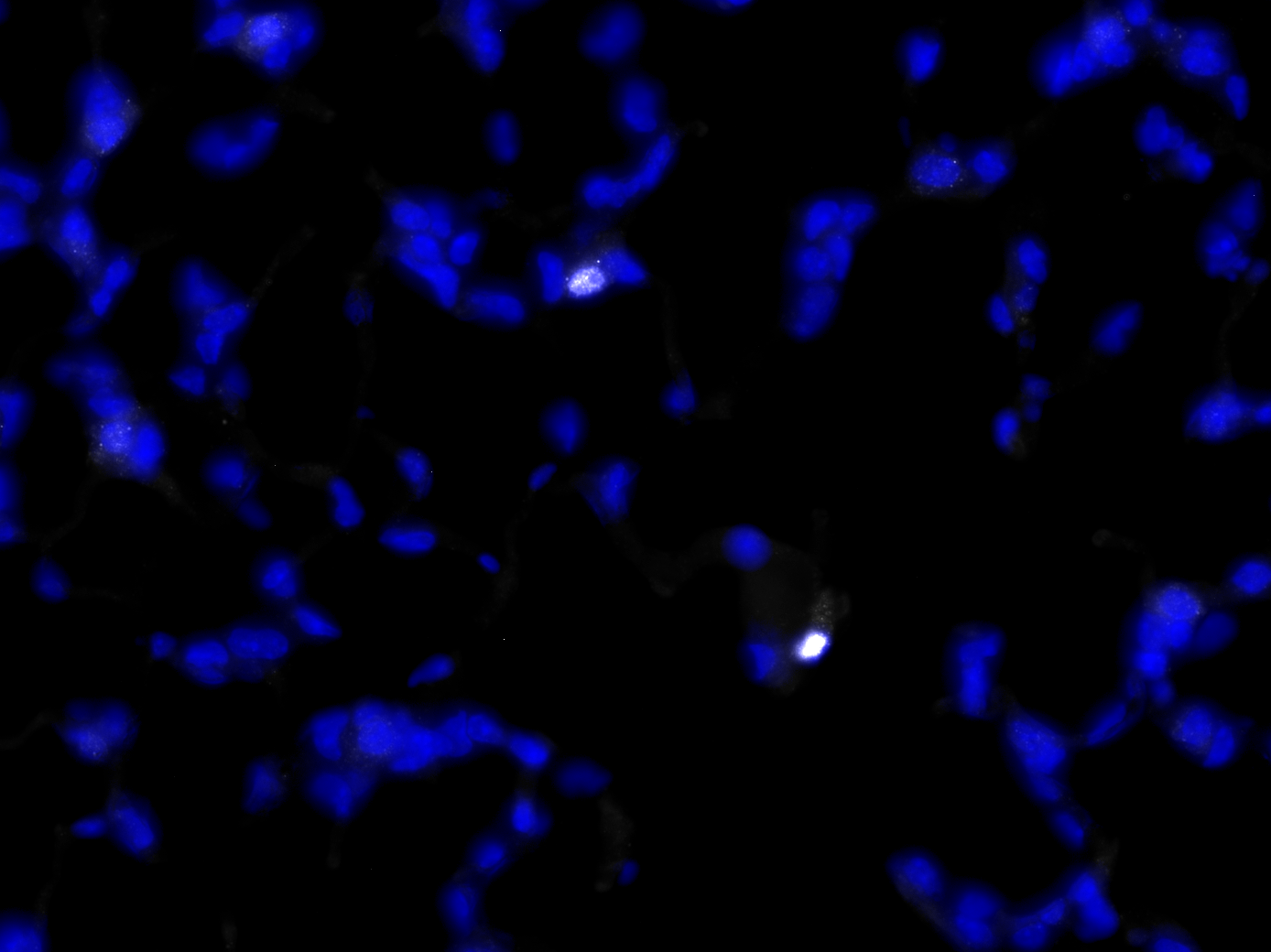

Supplement: Supplementary file 10 — Source Data Fig. 8 [file 44319_2023_41_MOESM10_ESM.zip › Source data Figure 8/8D Image data Micr images/young/p21+TERT CI (p21 + DAPI).tif]

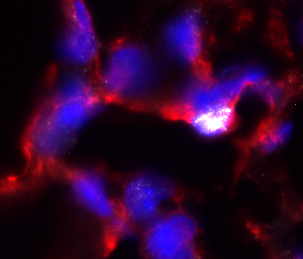

Supplement: Supplementary file 10 — Source Data Fig. 8 [file 44319_2023_41_MOESM10_ESM.zip › Source data Figure 8/8D Image data Micr images/young/p21++ (p21 + DAPI + CD34) ZOOM.tif]

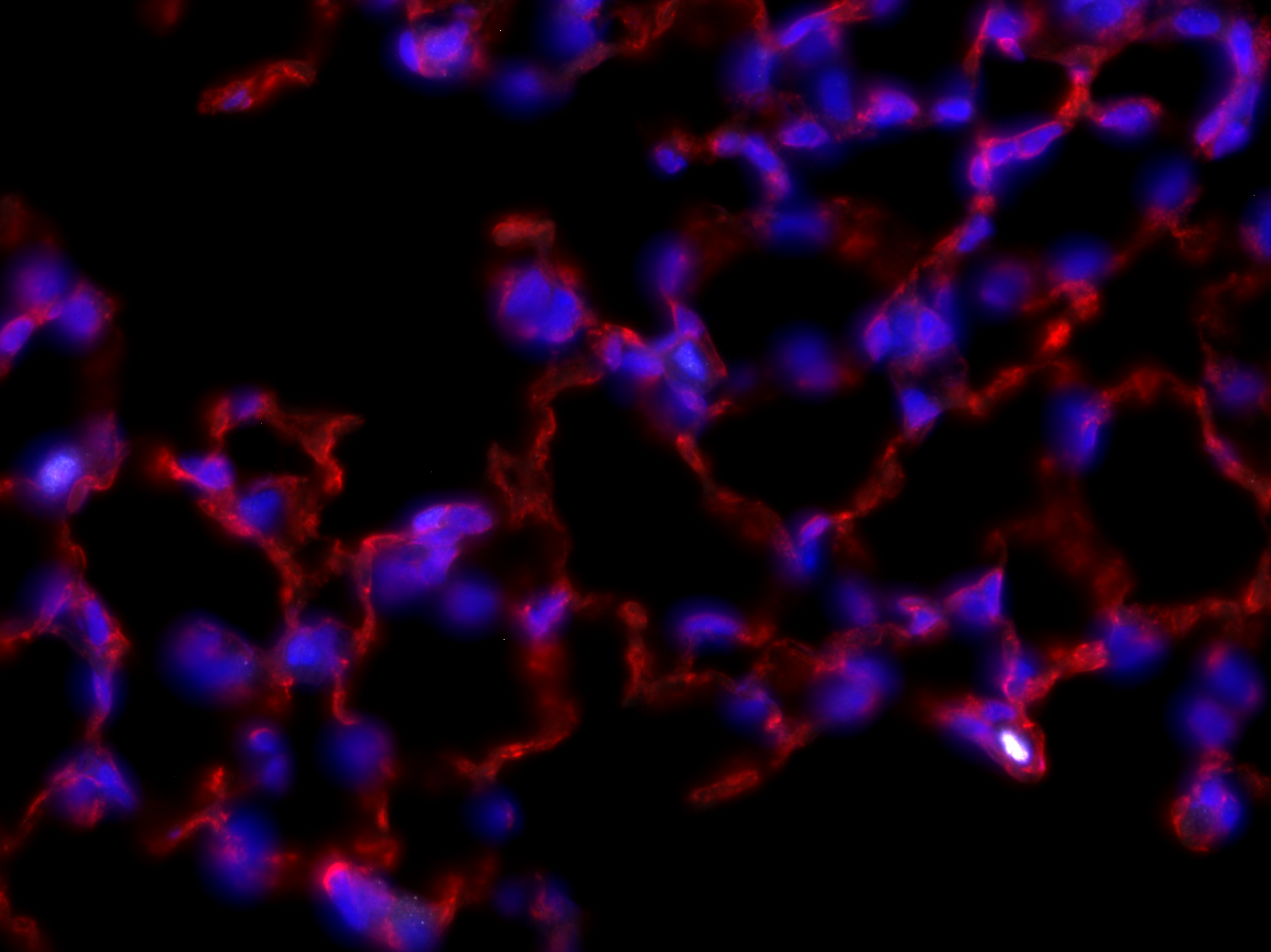

Supplement: Supplementary file 10 — Source Data Fig. 8 [file 44319_2023_41_MOESM10_ESM.zip › Source data Figure 8/8D Image data Micr images/young/p21+TERT (p21 + DAPI + CD34).tif]

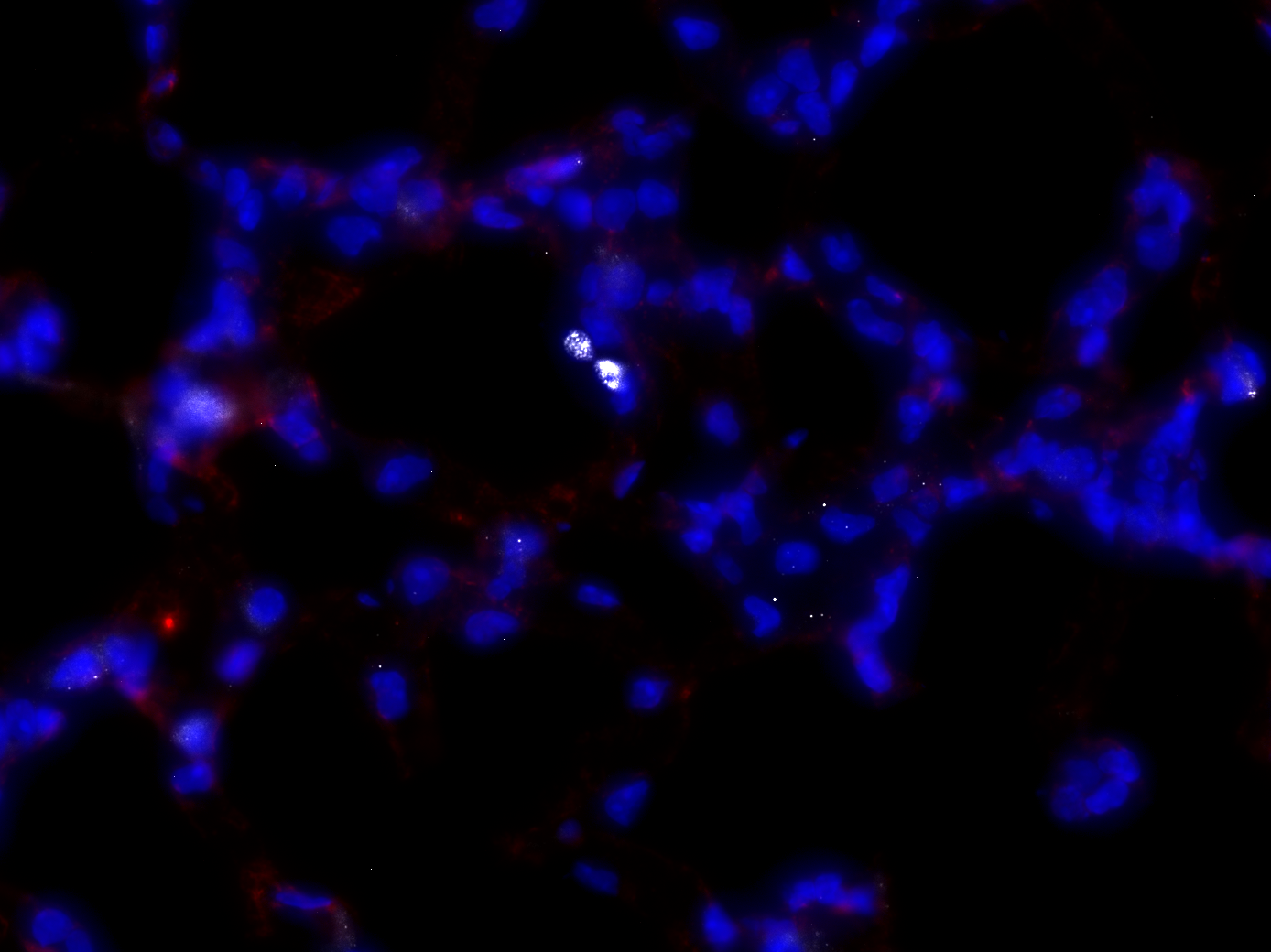

Supplement: Supplementary file 10 — Source Data Fig. 8 [file 44319_2023_41_MOESM10_ESM.zip › Source data Figure 8/8D Image data Micr images/aged/p21+TERT CI (p21 + DAPI + CD34).tif]

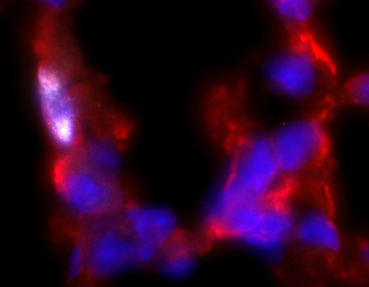

Supplement: Supplementary file 10 — Source Data Fig. 8 [file 44319_2023_41_MOESM10_ESM.zip › Source data Figure 8/8D Image data Micr images/aged/p21+TERT (p21 + DAPI + CD34) ZOOM.tif]

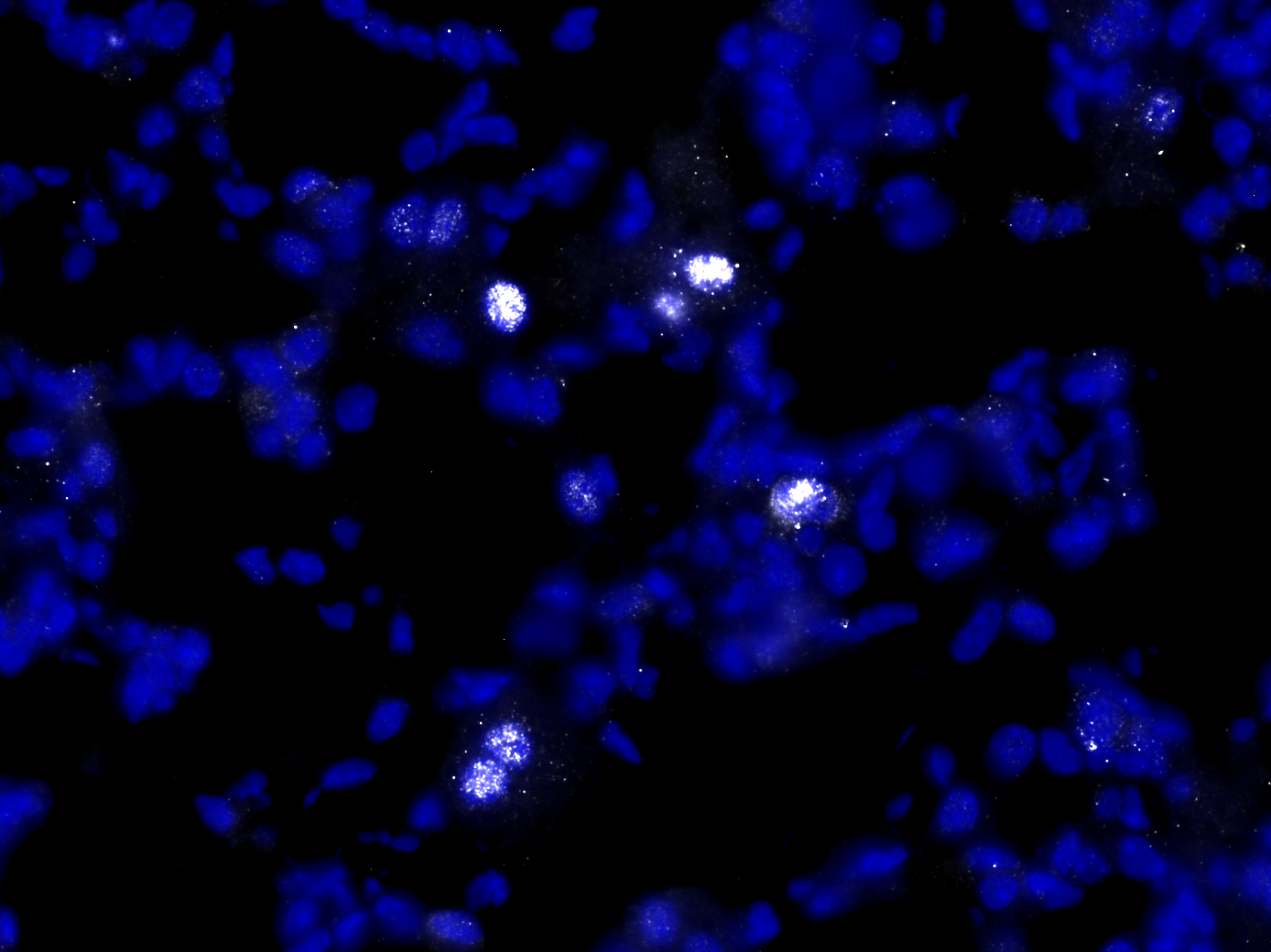

Supplement: Supplementary file 10 — Source Data Fig. 8 [file 44319_2023_41_MOESM10_ESM.zip › Source data Figure 8/8D Image data Micr images/aged/p21+- (p21 + DAPI).tif]

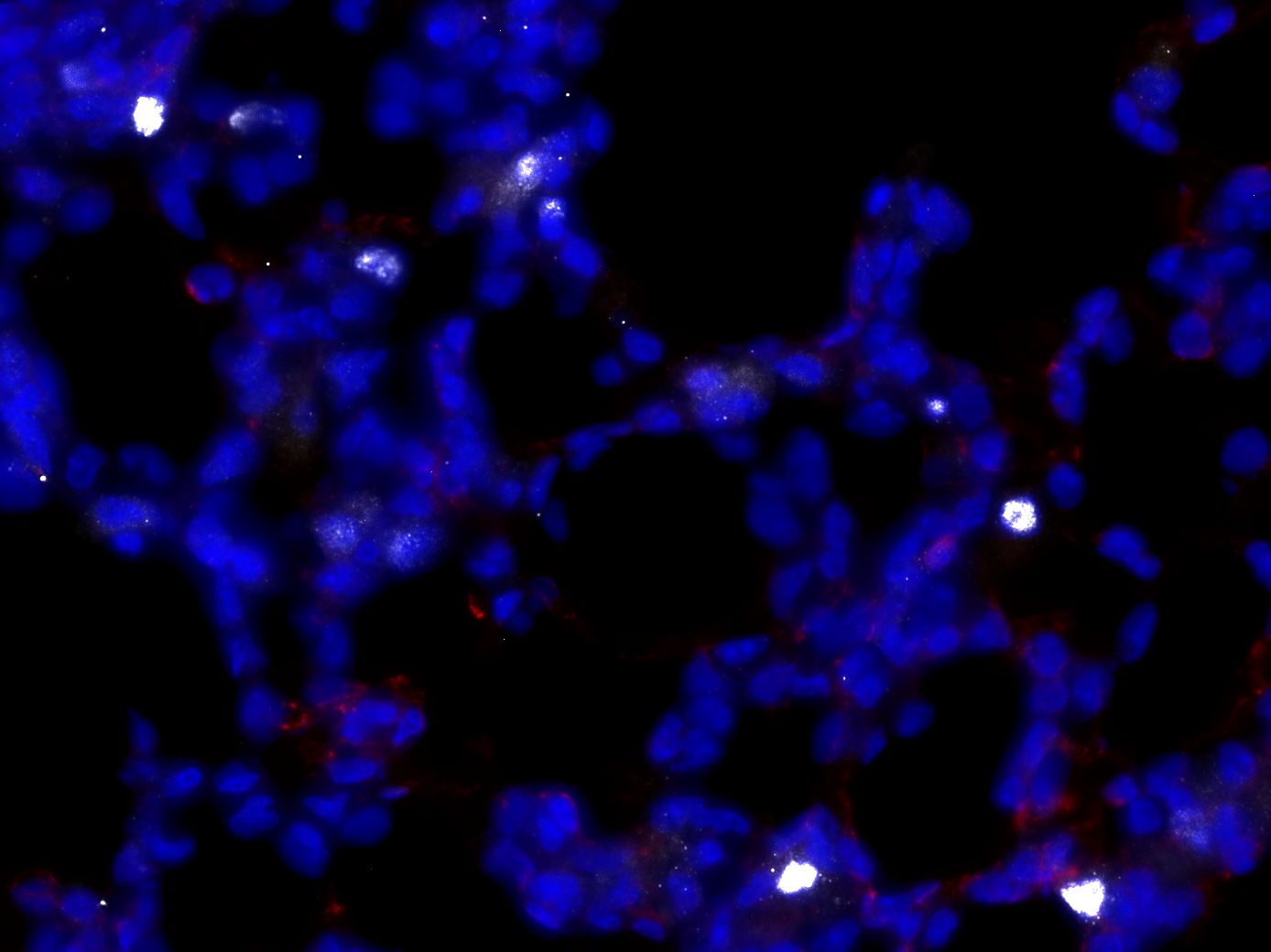

Supplement: Supplementary file 10 — Source Data Fig. 8 [file 44319_2023_41_MOESM10_ESM.zip › Source data Figure 8/8D Image data Micr images/aged/p21++ (p21 + DAPI + CD34).tif]

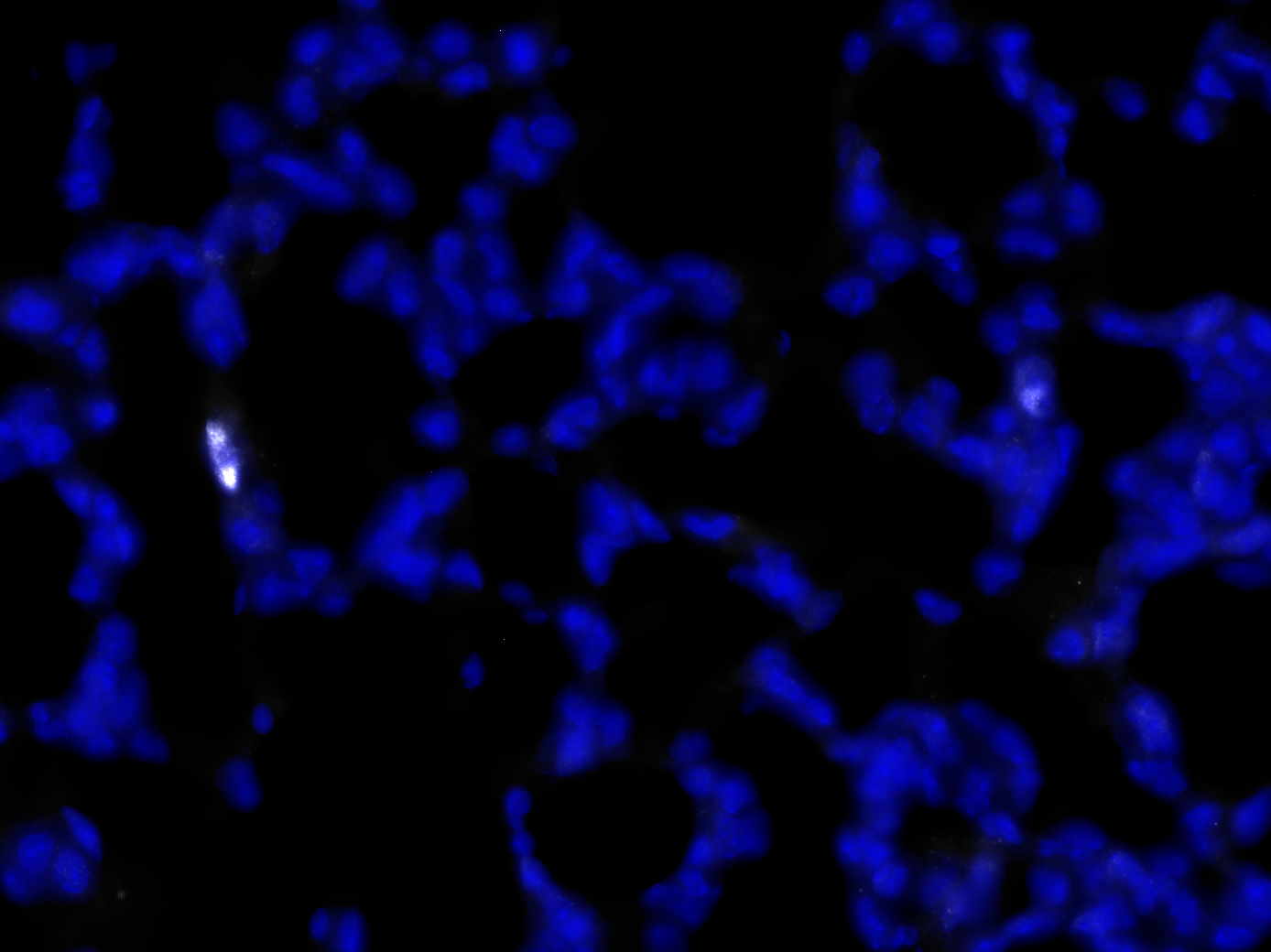

Supplement: Supplementary file 10 — Source Data Fig. 8 [file 44319_2023_41_MOESM10_ESM.zip › Source data Figure 8/8D Image data Micr images/aged/p21+TERT (p21 + DAPI).tif]

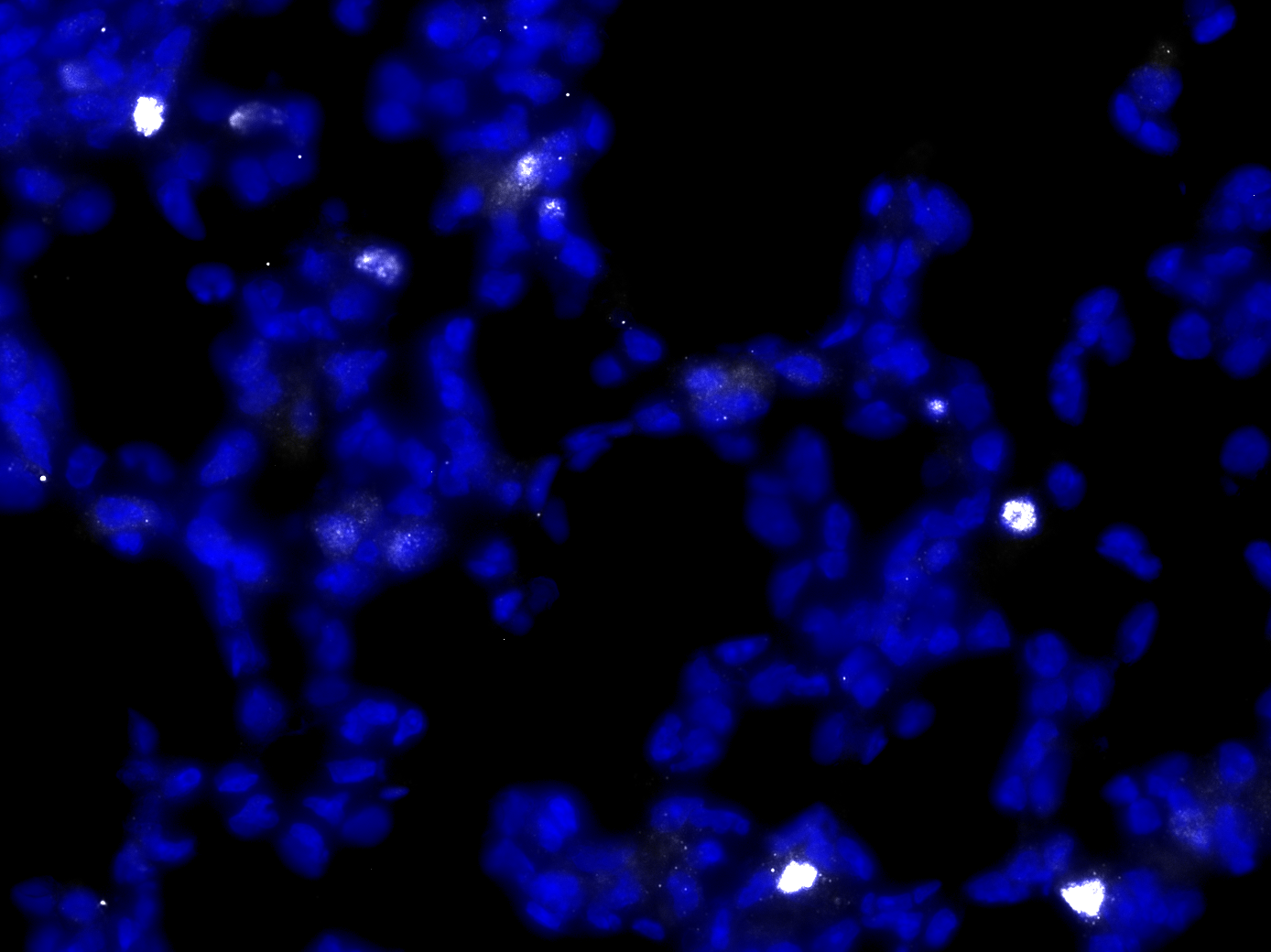

Supplement: Supplementary file 10 — Source Data Fig. 8 [file 44319_2023_41_MOESM10_ESM.zip › Source data Figure 8/8D Image data Micr images/aged/p21++ (p21 + DAPI).tif]

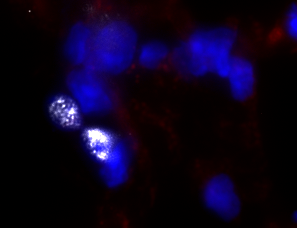

Supplement: Supplementary file 10 — Source Data Fig. 8 [file 44319_2023_41_MOESM10_ESM.zip › Source data Figure 8/8D Image data Micr images/aged/p21+TERT CI (p21 + DAPI + CD34) ZOOM.tif]

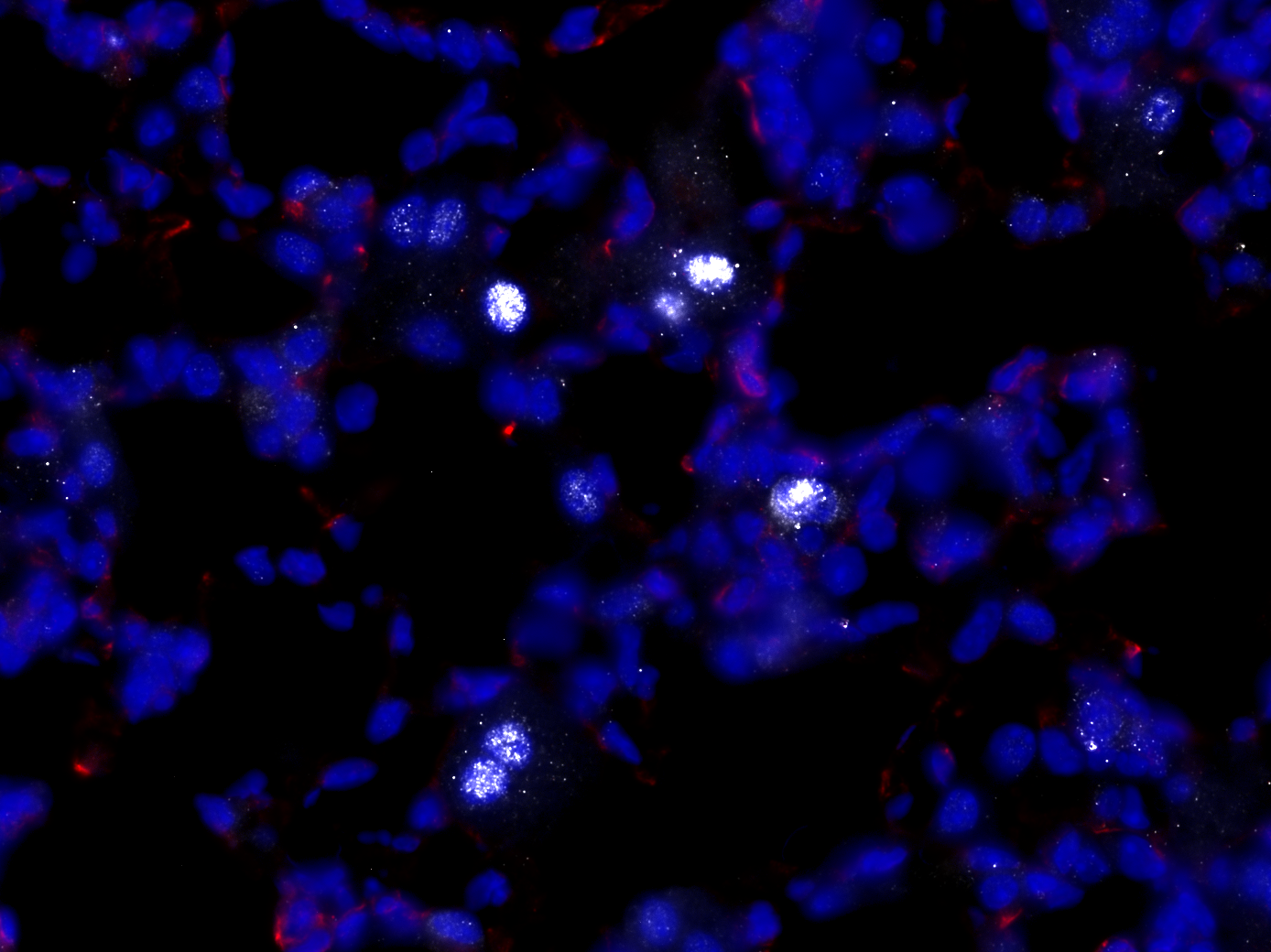

Supplement: Supplementary file 10 — Source Data Fig. 8 [file 44319_2023_41_MOESM10_ESM.zip › Source data Figure 8/8D Image data Micr images/aged/p21+- (p21 + DAPI+ CD34).tif]

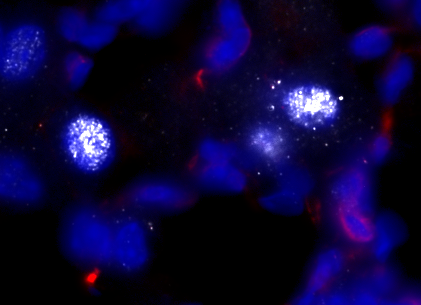

Supplement: Supplementary file 10 — Source Data Fig. 8 [file 44319_2023_41_MOESM10_ESM.zip › Source data Figure 8/8D Image data Micr images/aged/p21+- (p21 + DAPI + CD34) ZOOM.tif]

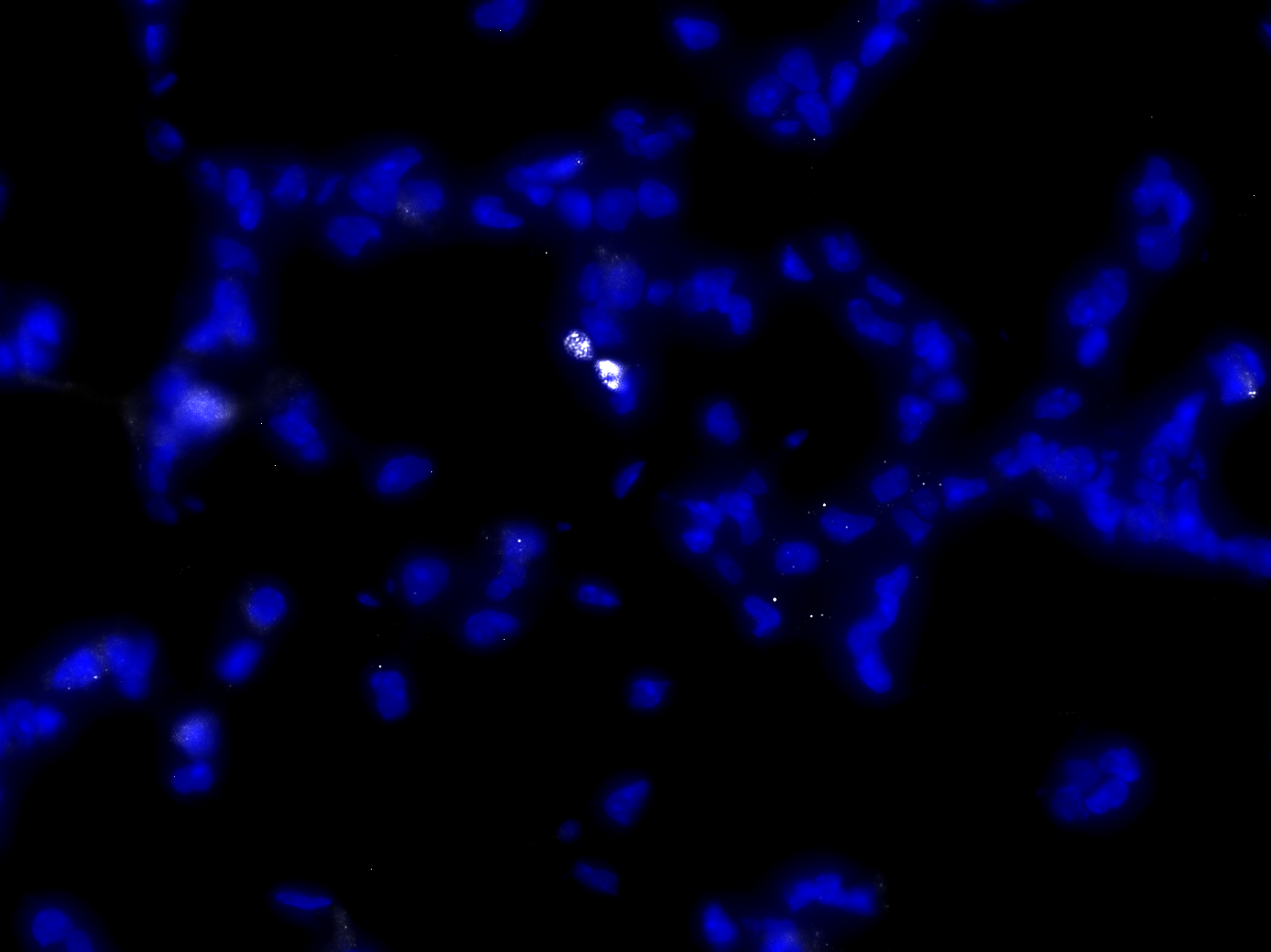

Supplement: Supplementary file 10 — Source Data Fig. 8 [file 44319_2023_41_MOESM10_ESM.zip › Source data Figure 8/8D Image data Micr images/aged/p21+TERT CI (p21 + DAPI).tif]

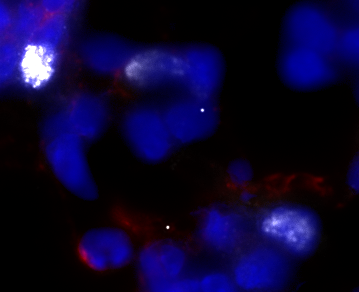

Supplement: Supplementary file 10 — Source Data Fig. 8 [file 44319_2023_41_MOESM10_ESM.zip › Source data Figure 8/8D Image data Micr images/aged/p21++ (p21 + DAPI + CD34) ZOOM.tif]

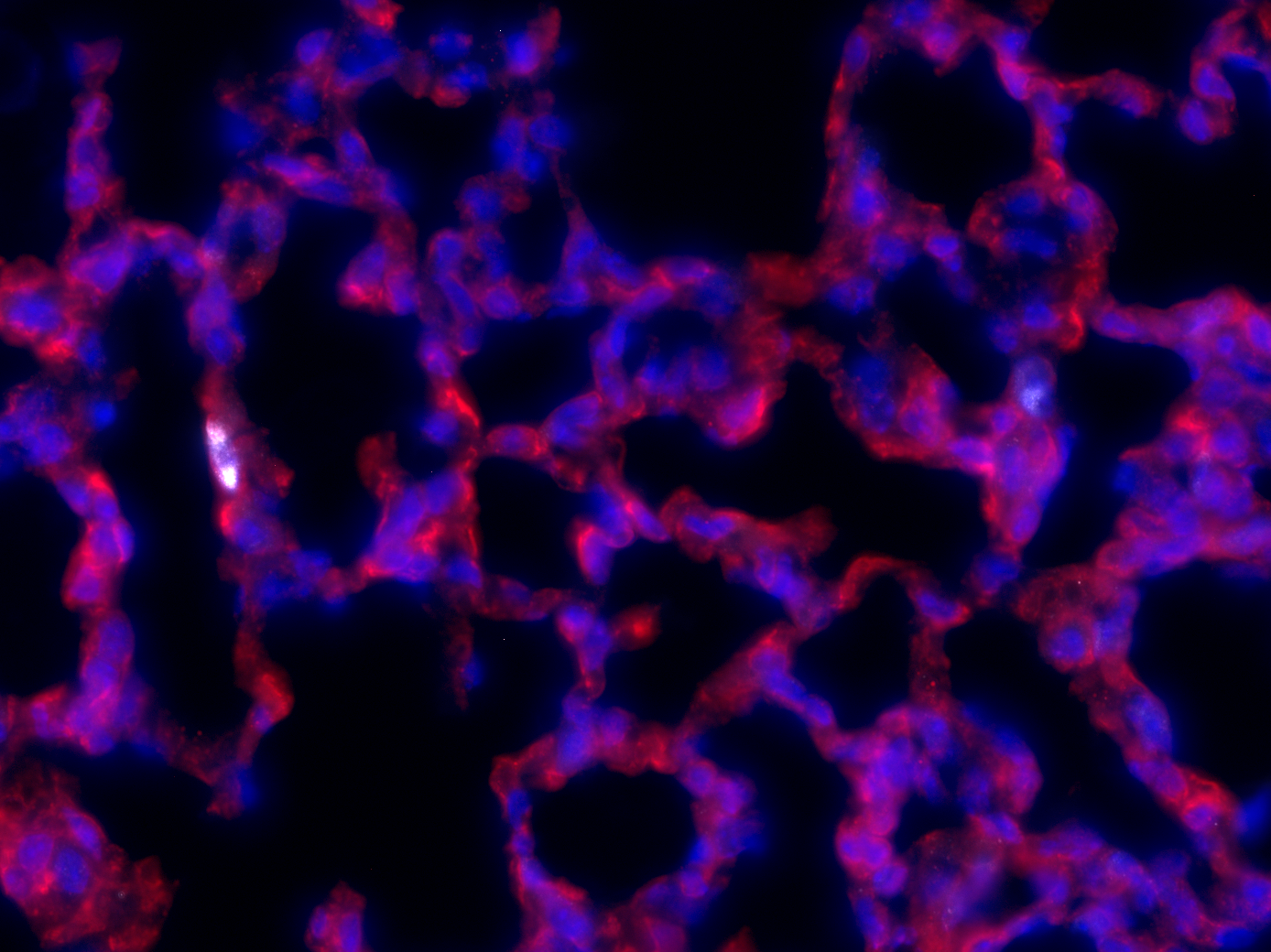

Supplement: Supplementary file 10 — Source Data Fig. 8 [file 44319_2023_41_MOESM10_ESM.zip › Source data Figure 8/8D Image data Micr images/aged/p21+TERT (p21 + DAPI + CD34).tif]

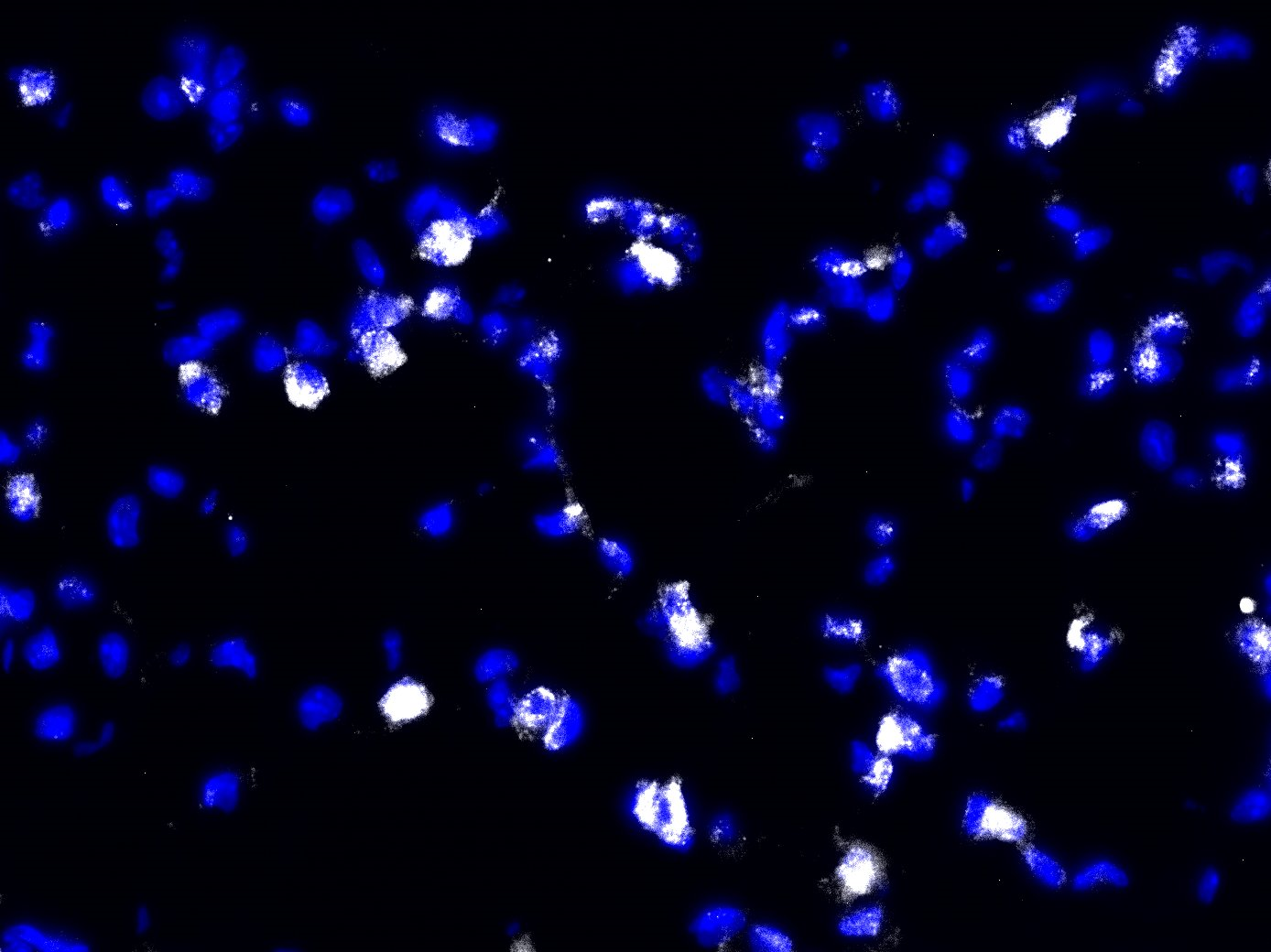

Supplement: Supplementary file 10 — Source Data Fig. 8 [file 44319_2023_41_MOESM10_ESM.zip › Source data Figure 8/8A Image data Micr images/young/p21+TERT young (p16 + DAPI).tif]

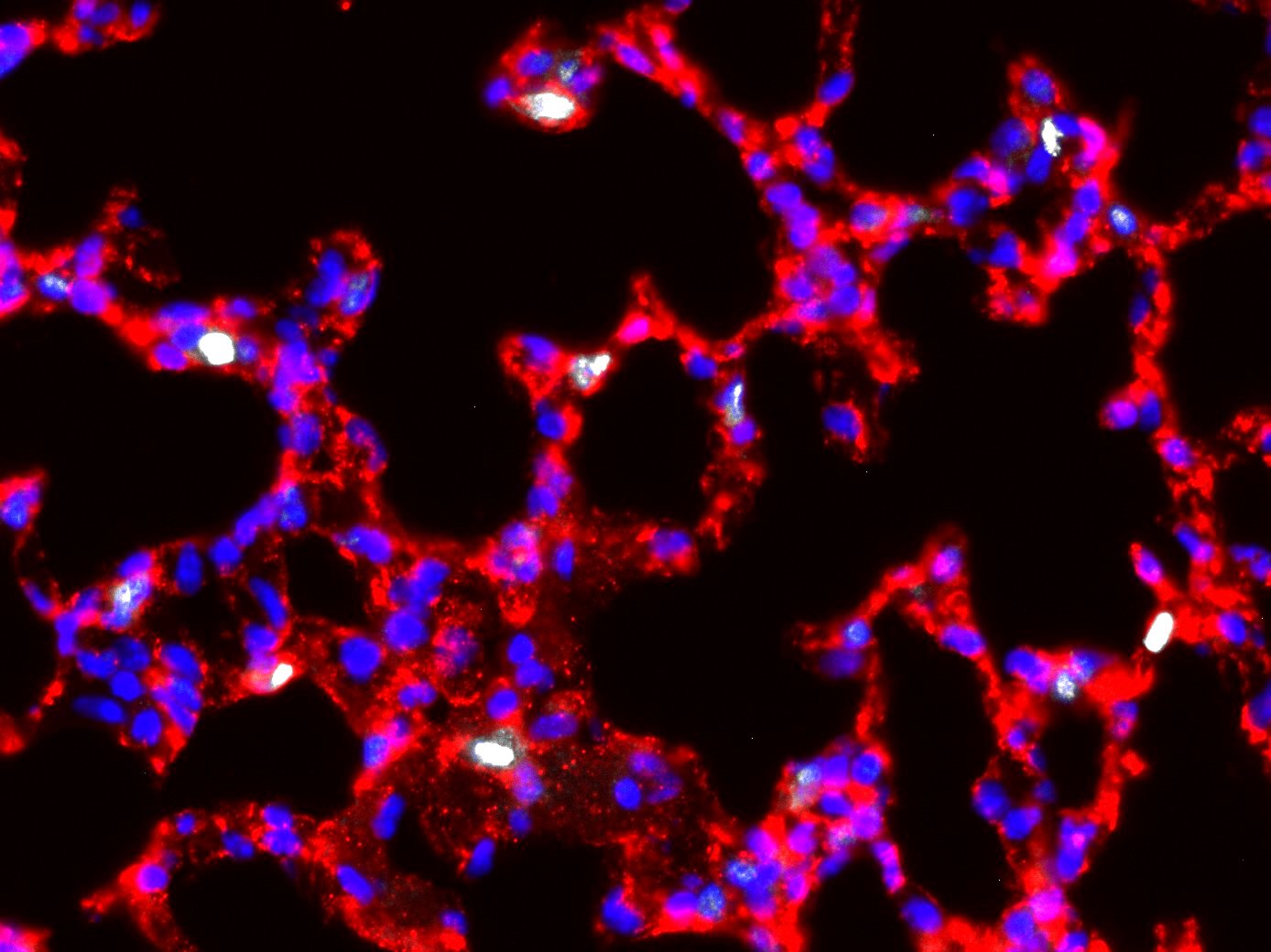

Supplement: Supplementary file 10 — Source Data Fig. 8 [file 44319_2023_41_MOESM10_ESM.zip › Source data Figure 8/8A Image data Micr images/young/p21+TERT CI young (p16 + DAPI + CD34).tif]

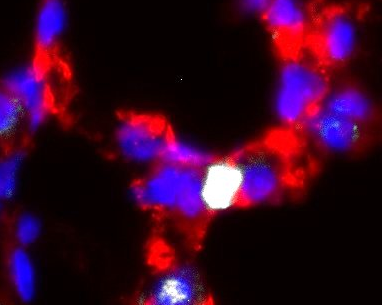

Supplement: Supplementary file 10 — Source Data Fig. 8 [file 44319_2023_41_MOESM10_ESM.zip › Source data Figure 8/8A Image data Micr images/young/p21+- young (p16 + DAPI + CD34) ZOOM.tif]

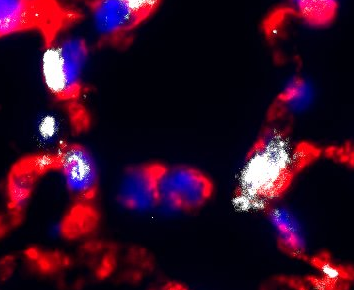

Supplement: Supplementary file 10 — Source Data Fig. 8 [file 44319_2023_41_MOESM10_ESM.zip › Source data Figure 8/8A Image data Micr images/young/p21++ young (p16 + DAPI + CD34) ZOOM.tif]

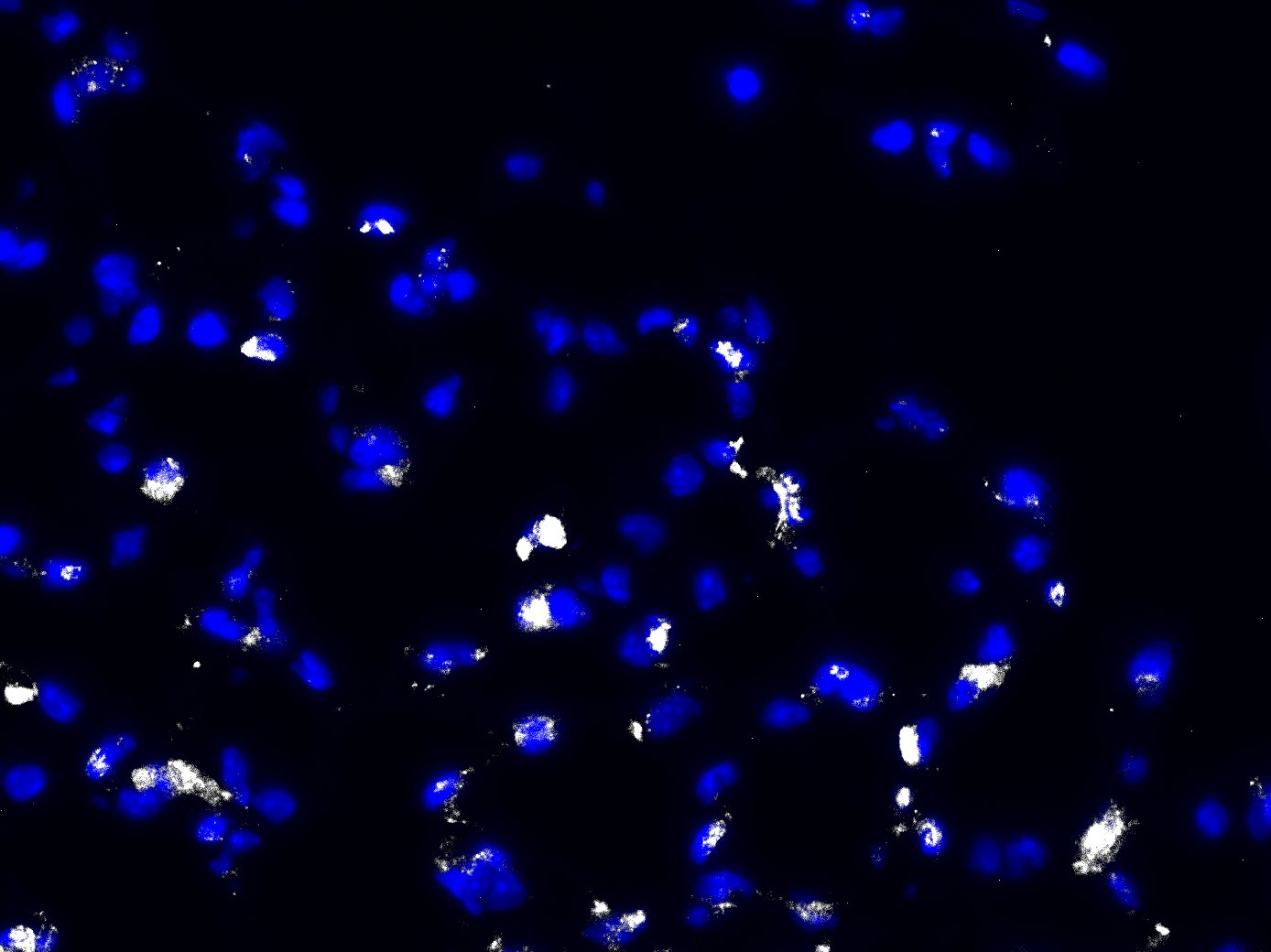

Supplement: Supplementary file 10 — Source Data Fig. 8 [file 44319_2023_41_MOESM10_ESM.zip › Source data Figure 8/8A Image data Micr images/young/p21++ young (p16 + DAPI).tif]

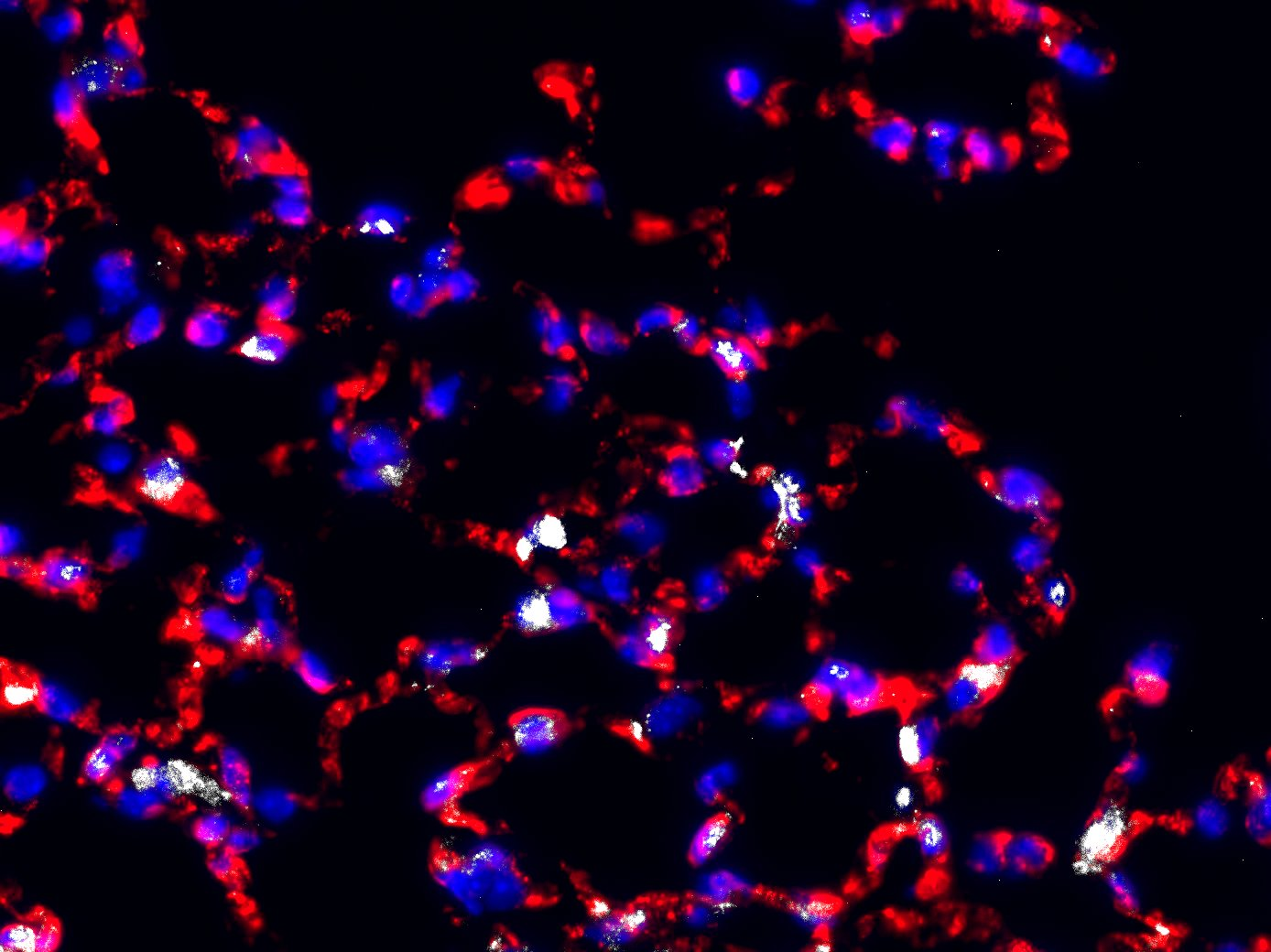

Supplement: Supplementary file 10 — Source Data Fig. 8 [file 44319_2023_41_MOESM10_ESM.zip › Source data Figure 8/8A Image data Micr images/young/p21++ young (p16 + DAPI + CD34).tif]

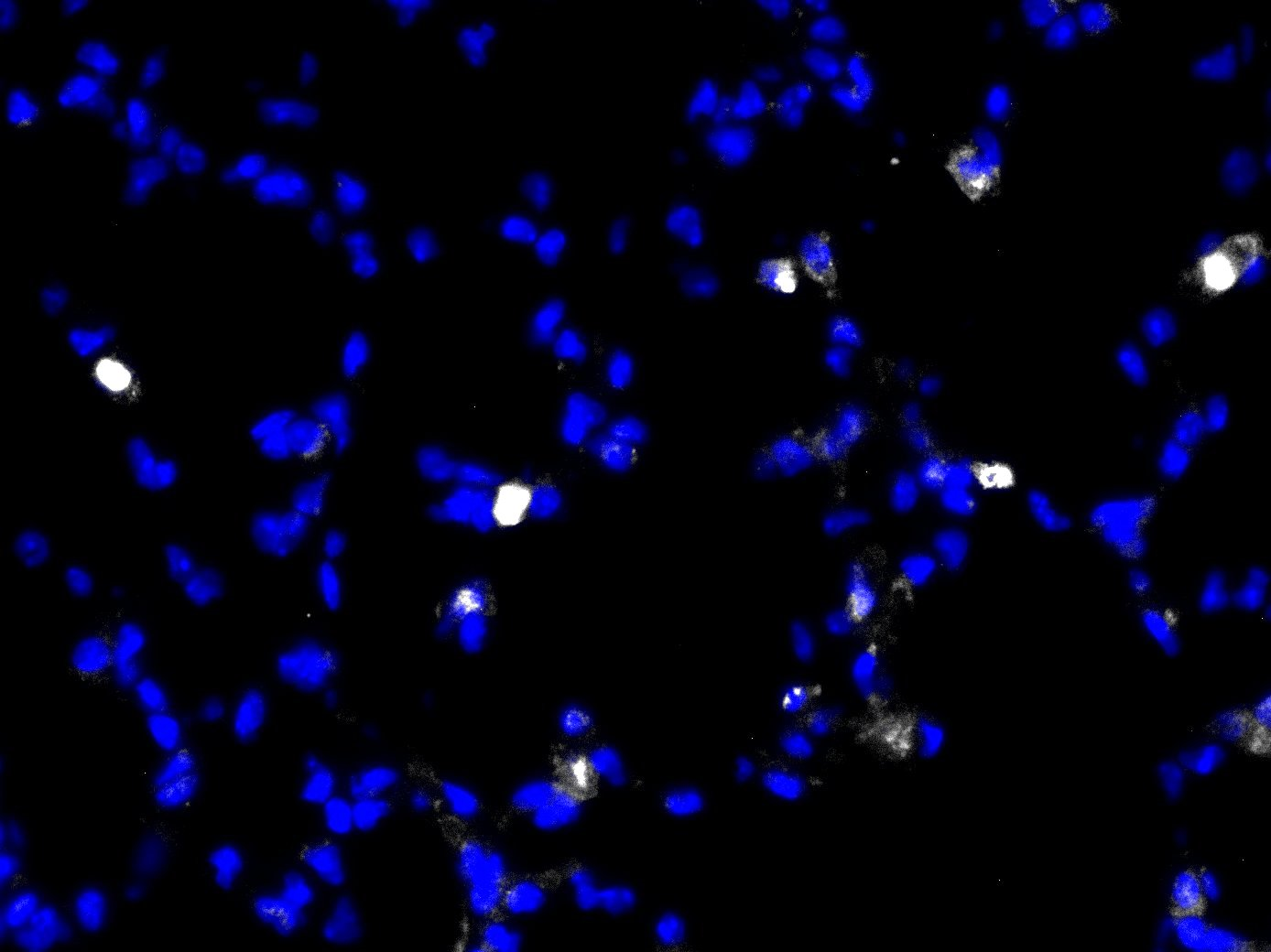

Supplement: Supplementary file 10 — Source Data Fig. 8 [file 44319_2023_41_MOESM10_ESM.zip › Source data Figure 8/8A Image data Micr images/young/p21+- young (p16 + DAPI).tif]

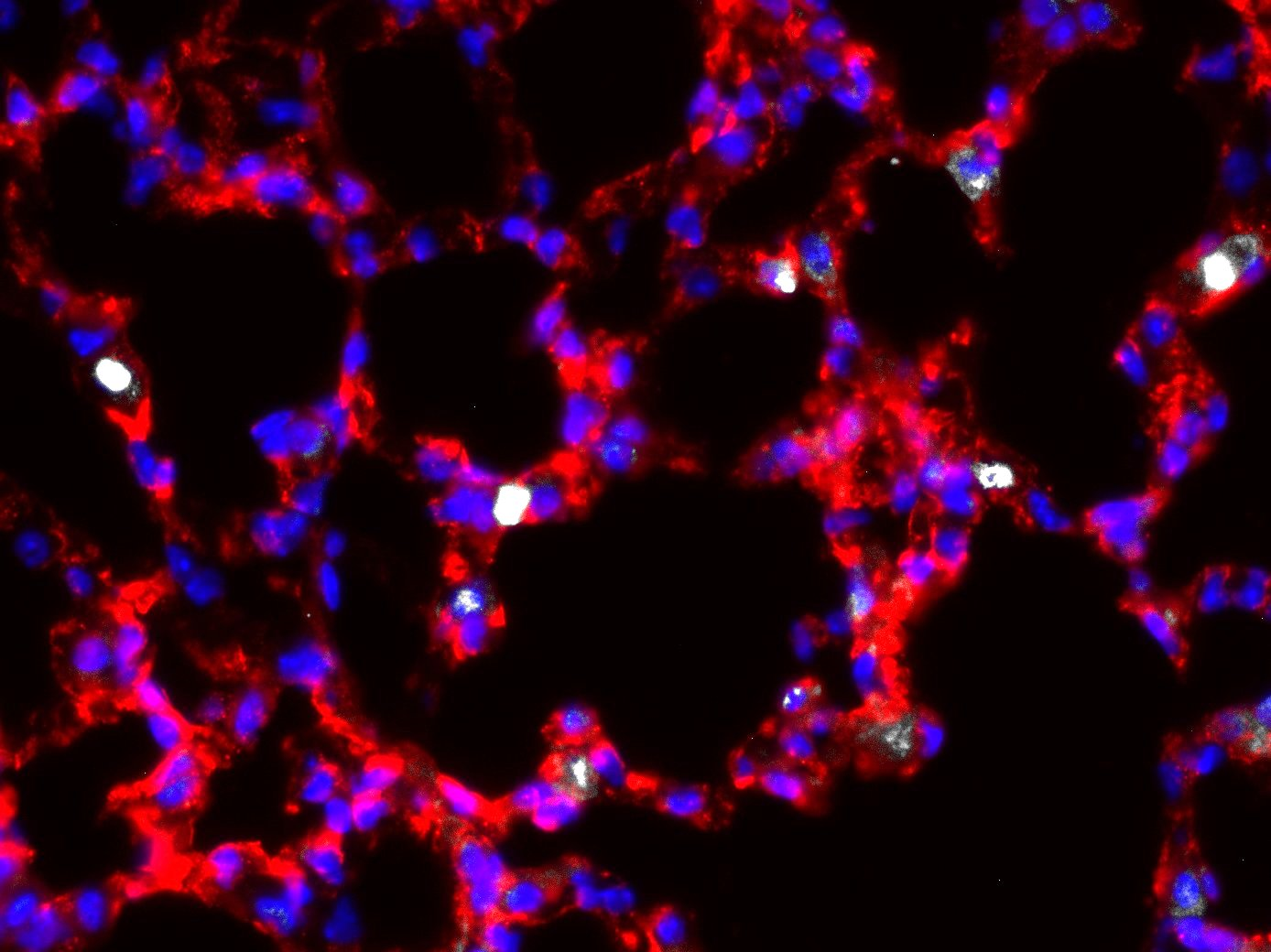

Supplement: Supplementary file 10 — Source Data Fig. 8 [file 44319_2023_41_MOESM10_ESM.zip › Source data Figure 8/8A Image data Micr images/young/p21+- young (p16 + DAPI + CD34).tif]

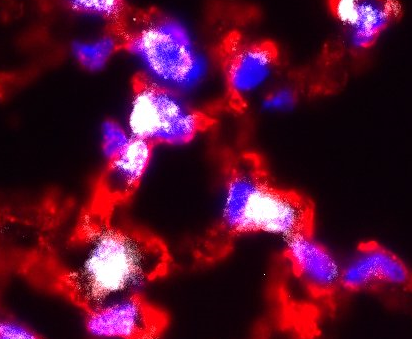

Supplement: Supplementary file 10 — Source Data Fig. 8 [file 44319_2023_41_MOESM10_ESM.zip › Source data Figure 8/8A Image data Micr images/young/p21+TERT young (p16 + DAPI + CD34) ZOOM.tif]

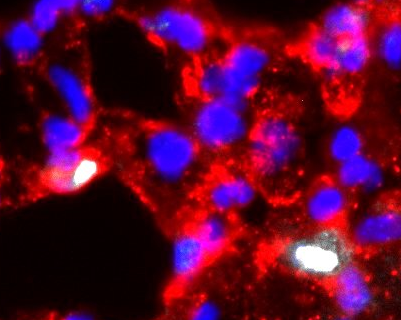

Supplement: Supplementary file 10 — Source Data Fig. 8 [file 44319_2023_41_MOESM10_ESM.zip › Source data Figure 8/8A Image data Micr images/young/p21+TERT CI young (p16 + DAPI + CD34) ZOOM.tif]

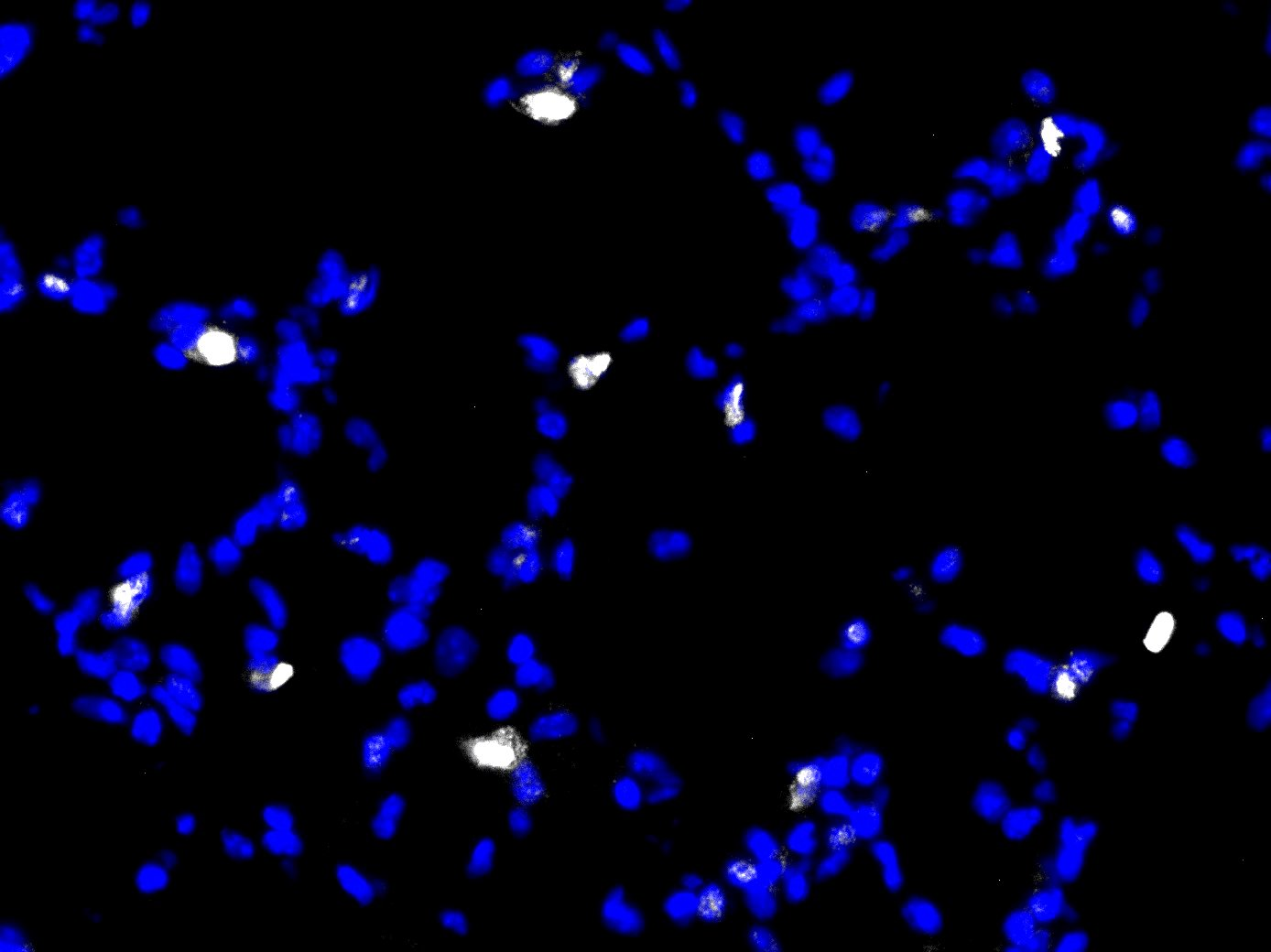

Supplement: Supplementary file 10 — Source Data Fig. 8 [file 44319_2023_41_MOESM10_ESM.zip › Source data Figure 8/8A Image data Micr images/young/p21+TERT CI young (p16 + DAPI).tif]

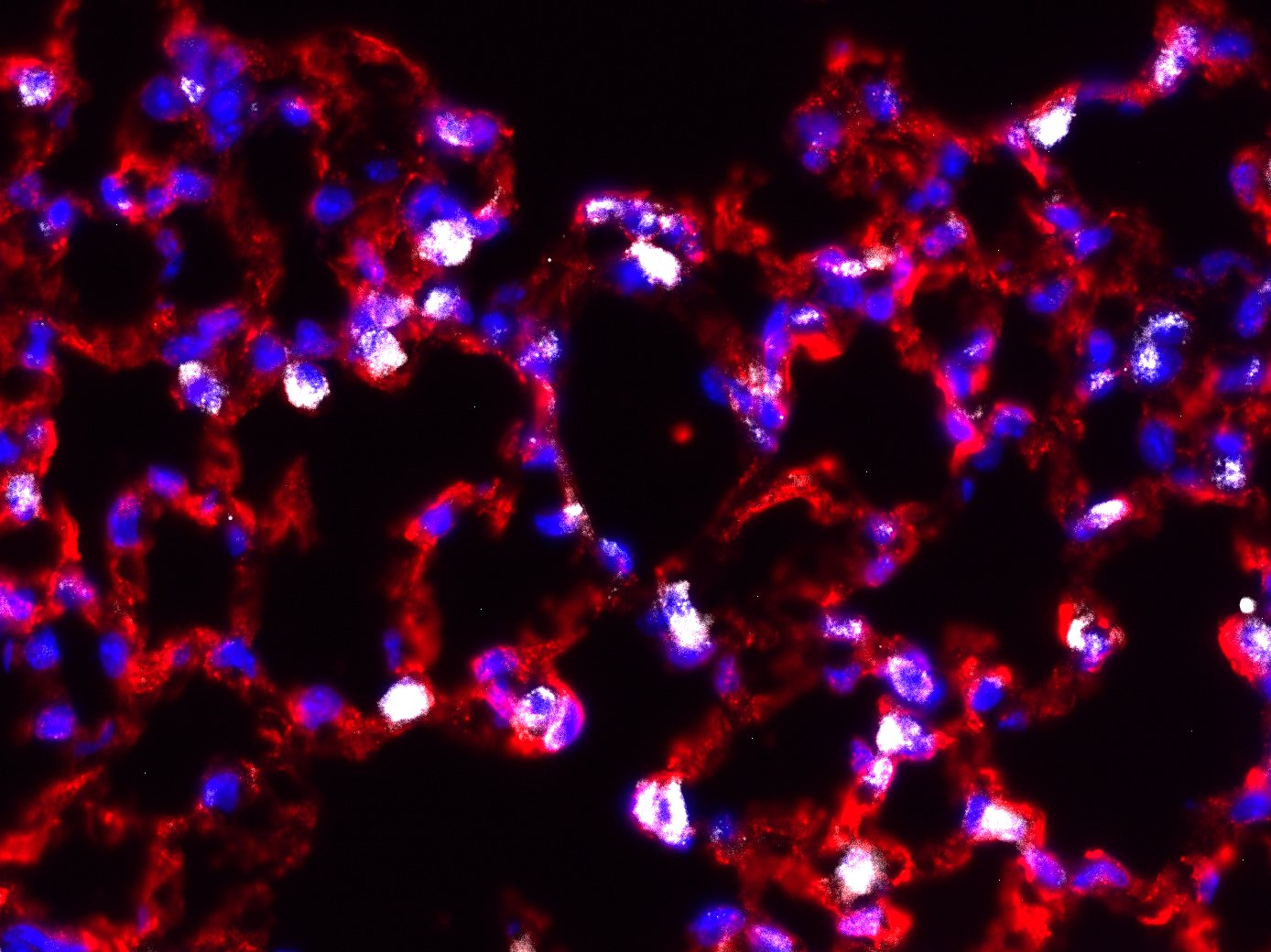

Supplement: Supplementary file 10 — Source Data Fig. 8 [file 44319_2023_41_MOESM10_ESM.zip › Source data Figure 8/8A Image data Micr images/young/p21+TERT young (p16 + DAPI + CD34).tif]

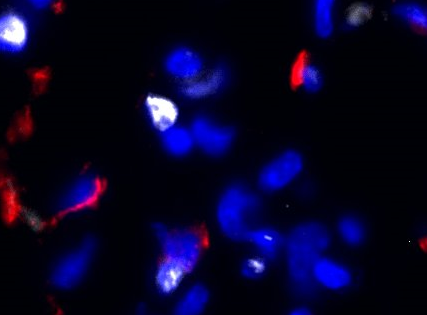

Supplement: Supplementary file 10 — Source Data Fig. 8 [file 44319_2023_41_MOESM10_ESM.zip › Source data Figure 8/8A Image data Micr images/aged/p21+TERT CI aged (p16 + DAPI + CD34) ZOOM.tif]

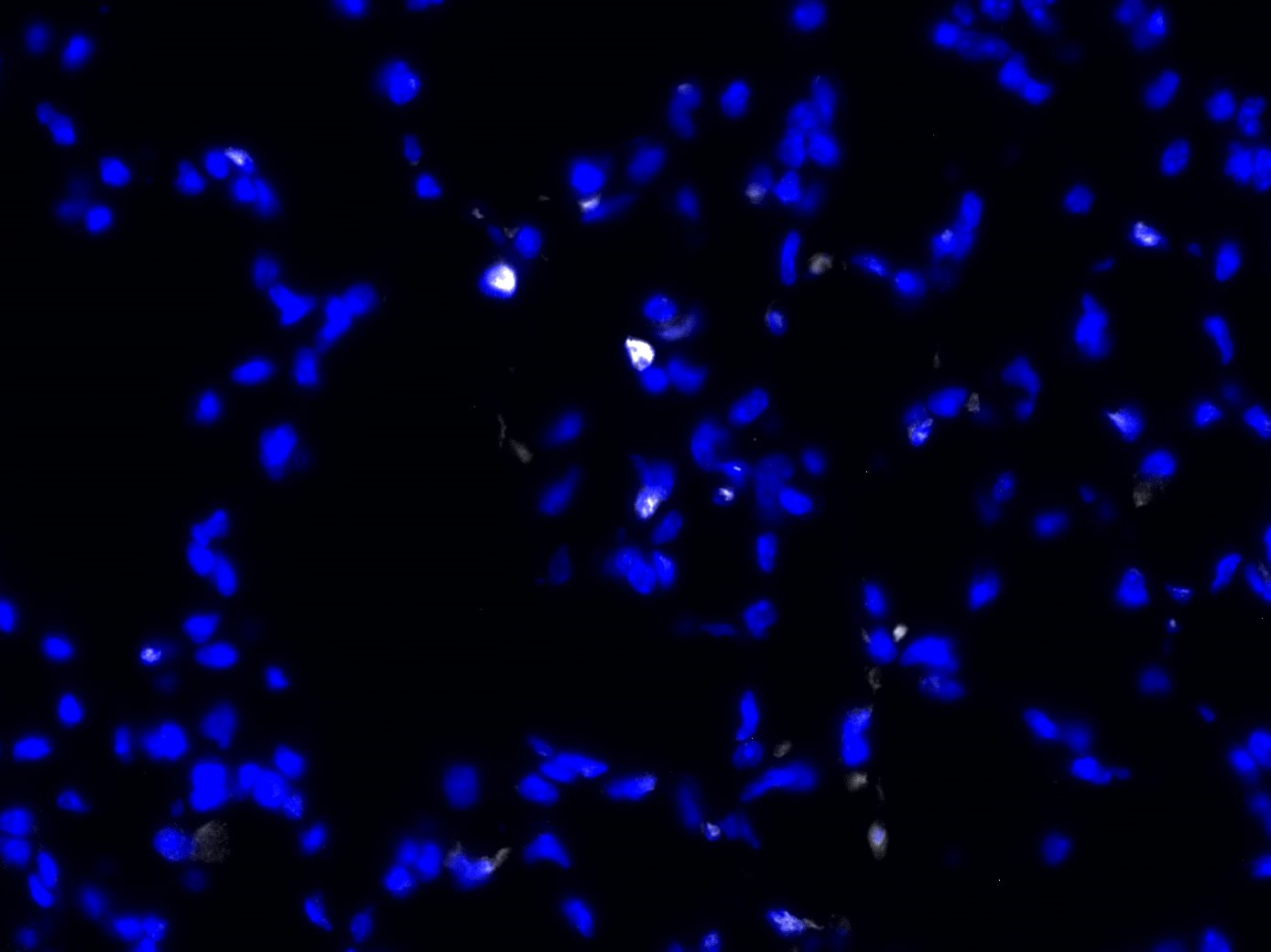

Supplement: Supplementary file 10 — Source Data Fig. 8 [file 44319_2023_41_MOESM10_ESM.zip › Source data Figure 8/8A Image data Micr images/aged/p21+TERT CI aged (p16 + DAPI).tif]

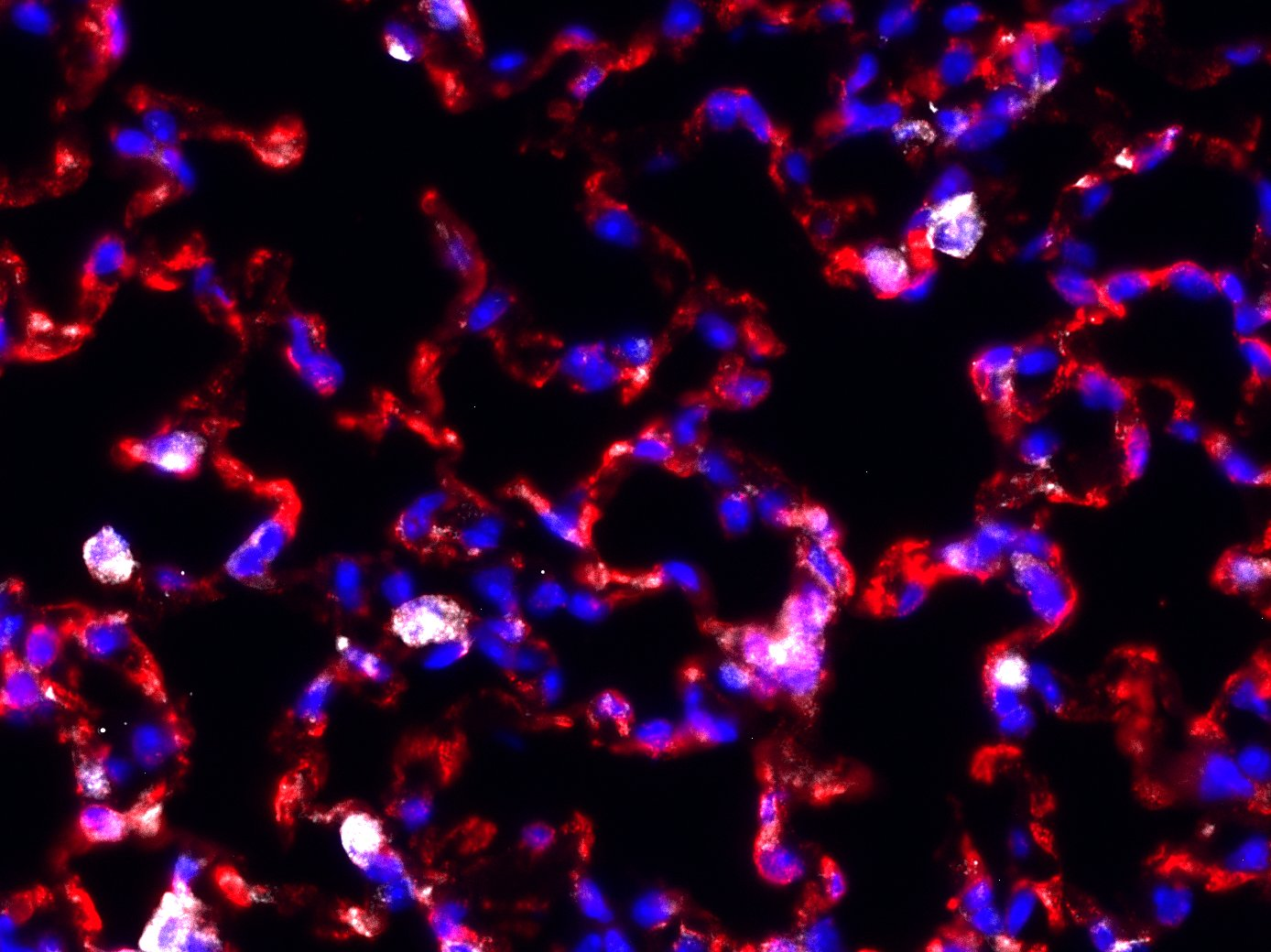

Supplement: Supplementary file 10 — Source Data Fig. 8 [file 44319_2023_41_MOESM10_ESM.zip › Source data Figure 8/8A Image data Micr images/aged/p21+TERT aged (p16 + DAPI + CD34).tif]

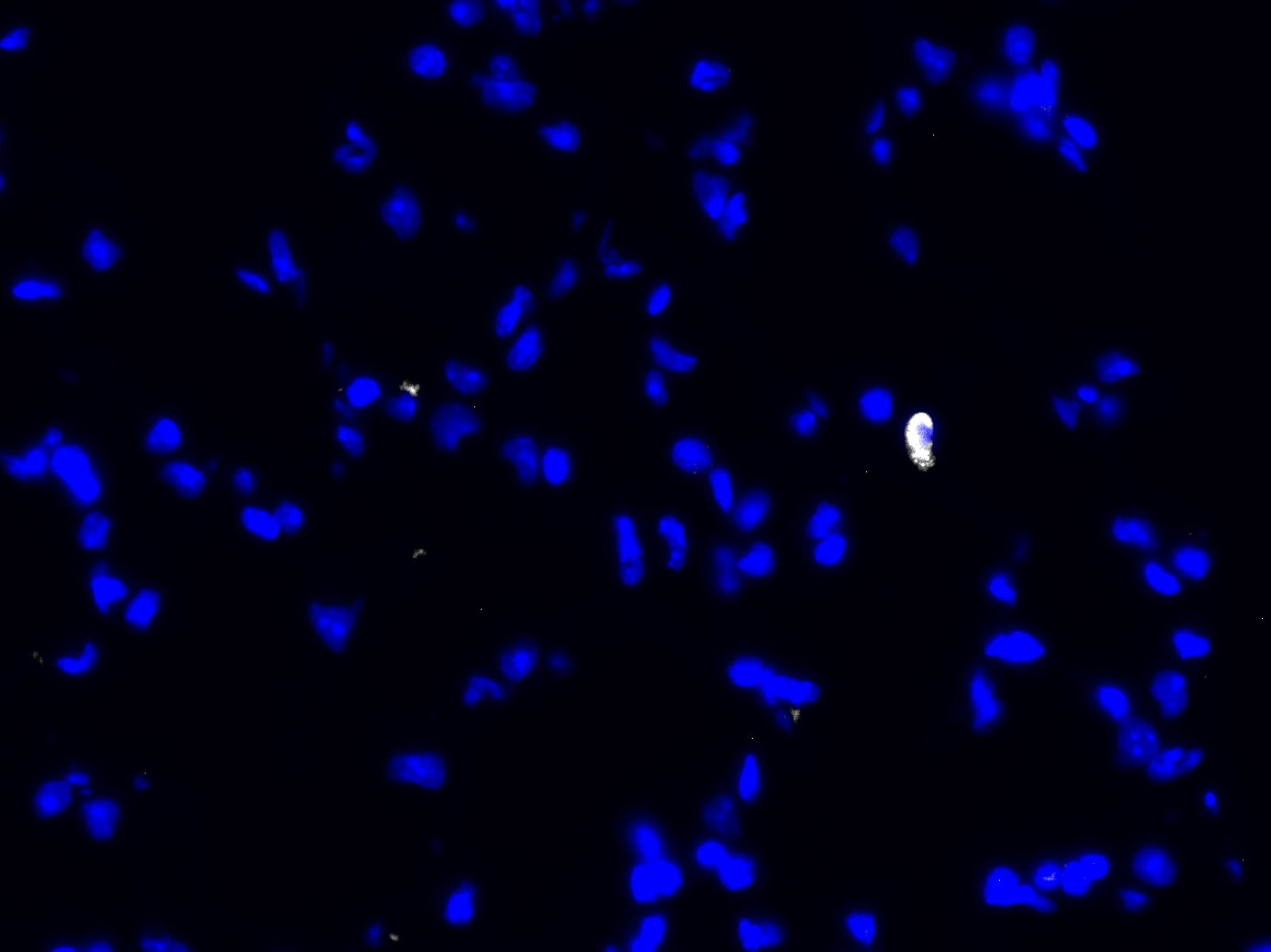

Supplement: Supplementary file 10 — Source Data Fig. 8 [file 44319_2023_41_MOESM10_ESM.zip › Source data Figure 8/8A Image data Micr images/aged/p21+- aged (p16 + DAPI).tif]

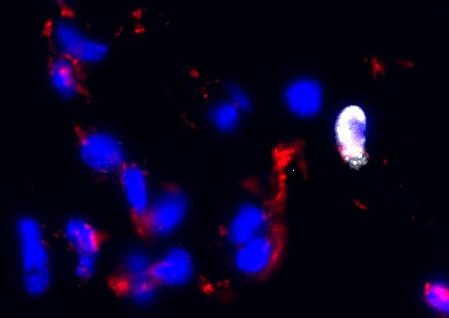

Supplement: Supplementary file 10 — Source Data Fig. 8 [file 44319_2023_41_MOESM10_ESM.zip › Source data Figure 8/8A Image data Micr images/aged/p21+- aged (p16 + DAPI + CD34) ZOOM.tif]

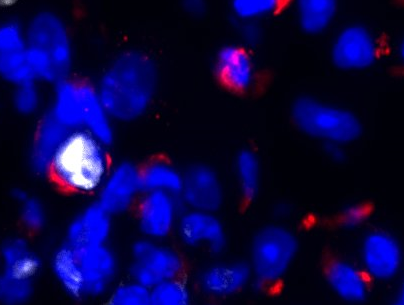

Supplement: Supplementary file 10 — Source Data Fig. 8 [file 44319_2023_41_MOESM10_ESM.zip › Source data Figure 8/8A Image data Micr images/aged/p21++ aged (p16 + DAPI + CD34) ZOOM.tif]

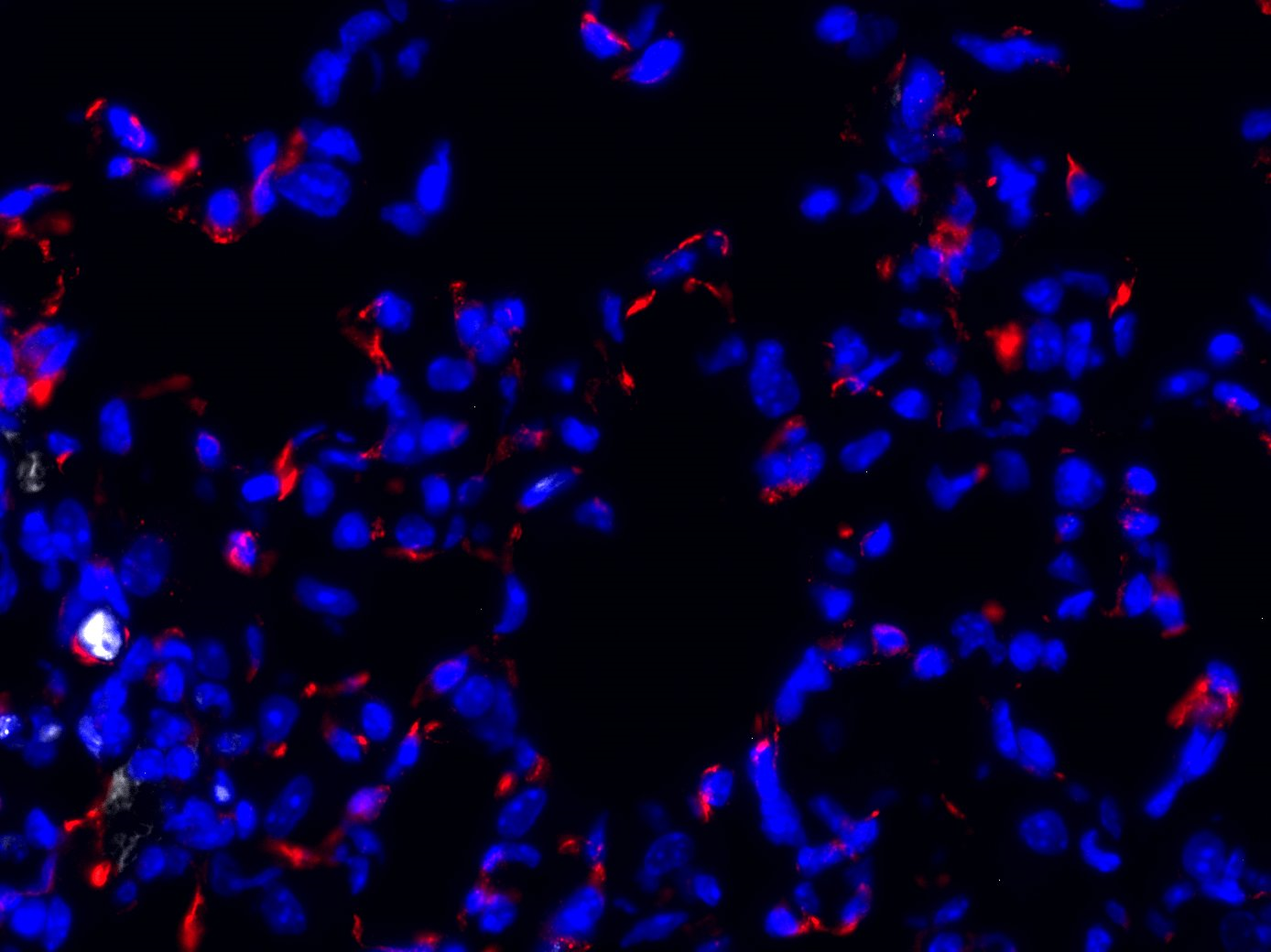

Supplement: Supplementary file 10 — Source Data Fig. 8 [file 44319_2023_41_MOESM10_ESM.zip › Source data Figure 8/8A Image data Micr images/aged/p21++ aged (p16 + DAPI + CD34).tif]

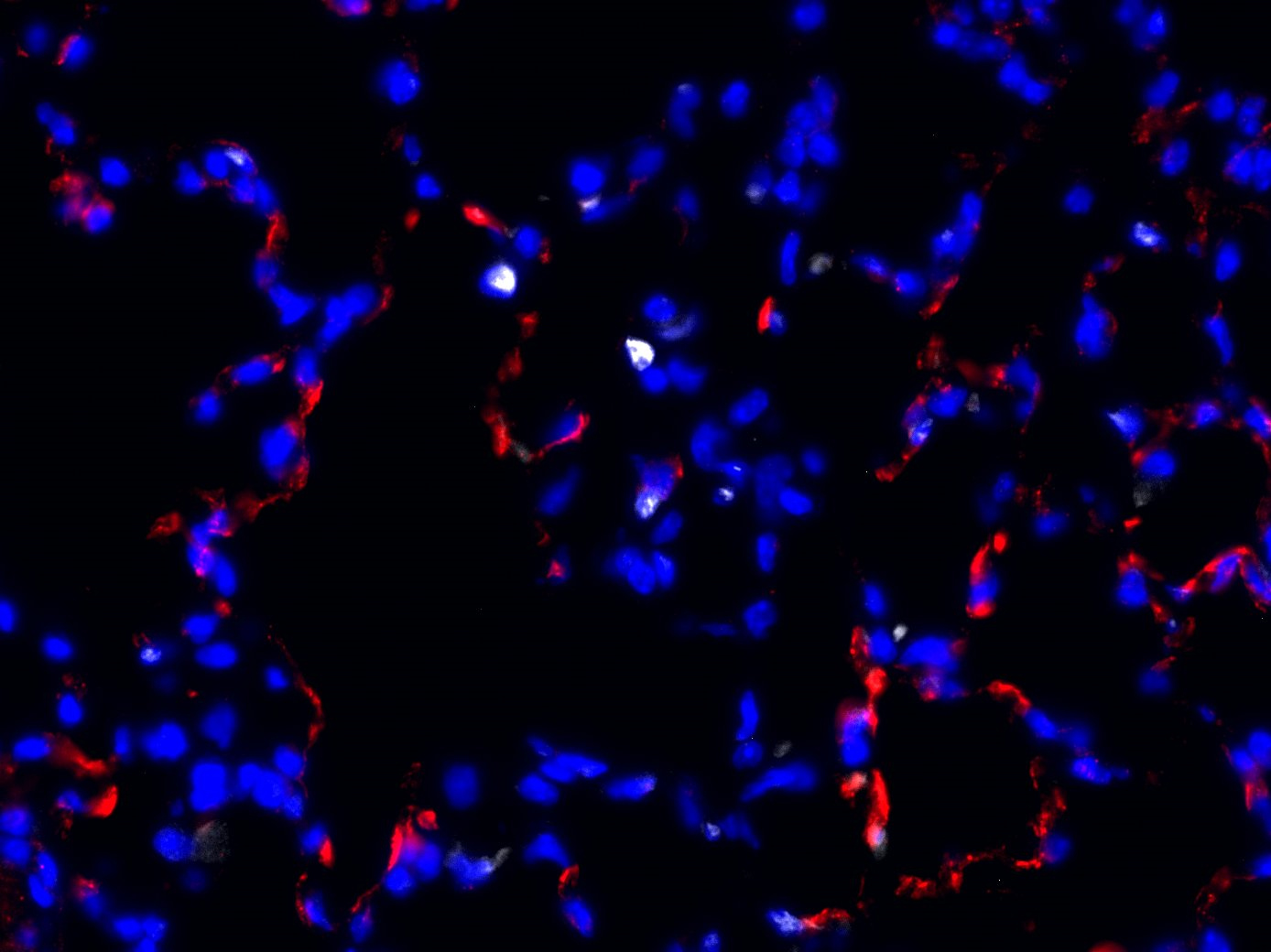

Supplement: Supplementary file 10 — Source Data Fig. 8 [file 44319_2023_41_MOESM10_ESM.zip › Source data Figure 8/8A Image data Micr images/aged/p21+TERT CI aged (p16 + DAPI + CD34).tif]

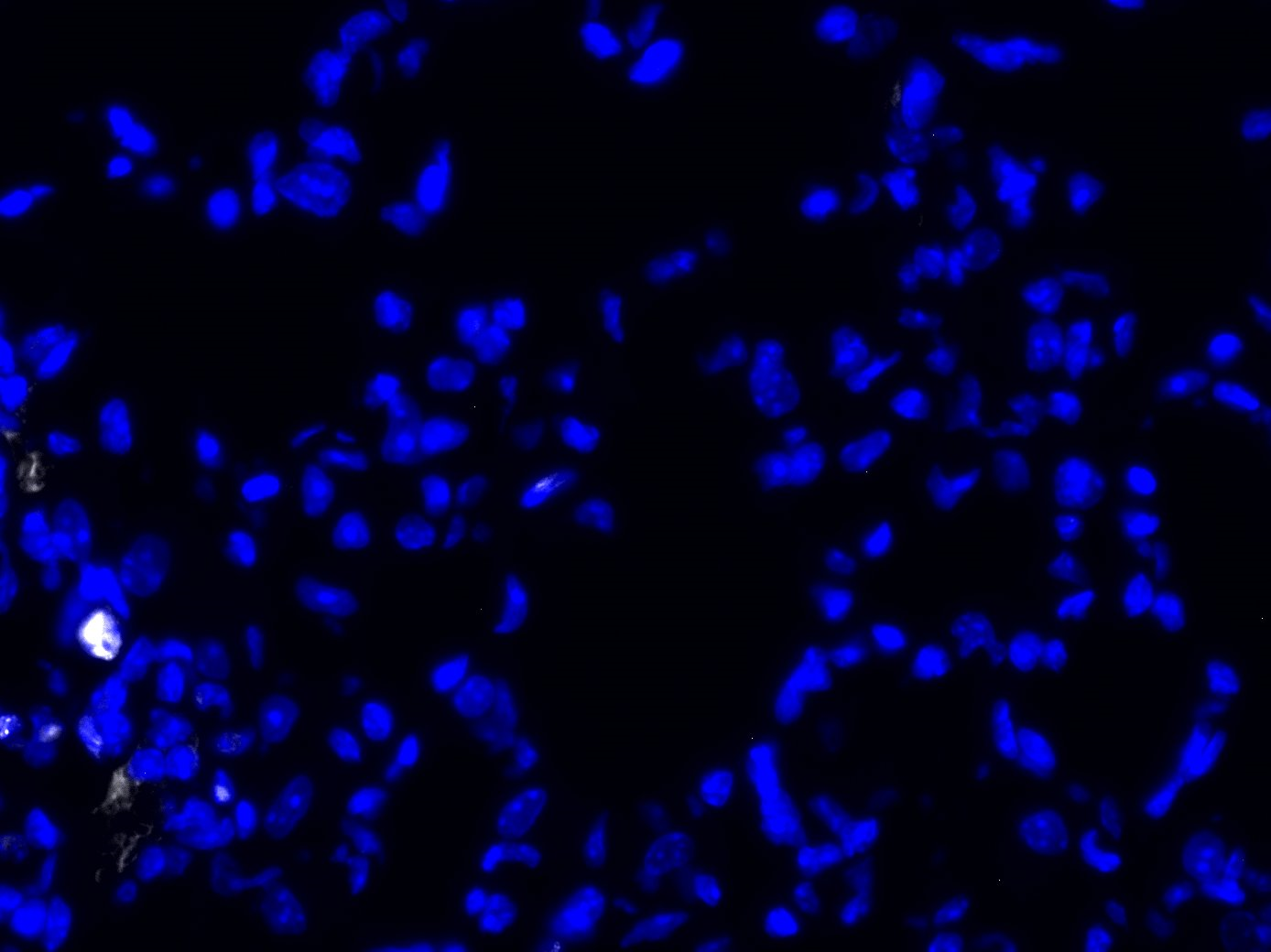

Supplement: Supplementary file 10 — Source Data Fig. 8 [file 44319_2023_41_MOESM10_ESM.zip › Source data Figure 8/8A Image data Micr images/aged/p21++ aged (p16 + DAPI).tif]

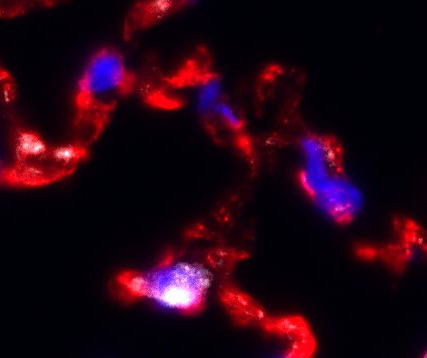

Supplement: Supplementary file 10 — Source Data Fig. 8 [file 44319_2023_41_MOESM10_ESM.zip › Source data Figure 8/8A Image data Micr images/aged/p21+TERT aged (p16 + DAPI + CD34) ZOOM.tif]

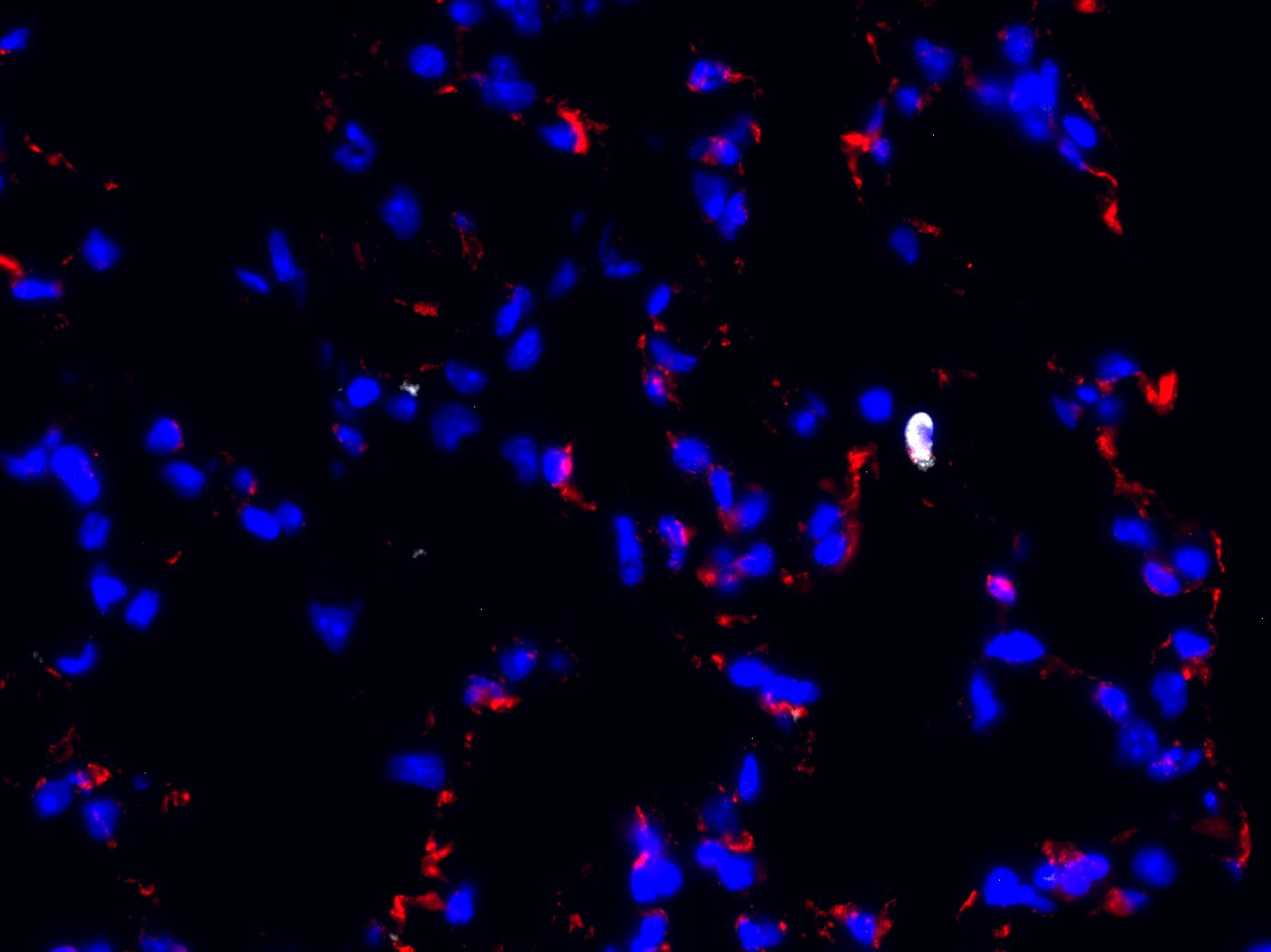

Supplement: Supplementary file 10 — Source Data Fig. 8 [file 44319_2023_41_MOESM10_ESM.zip › Source data Figure 8/8A Image data Micr images/aged/p21+- aged (p16 + DAPI + CD34).tif]

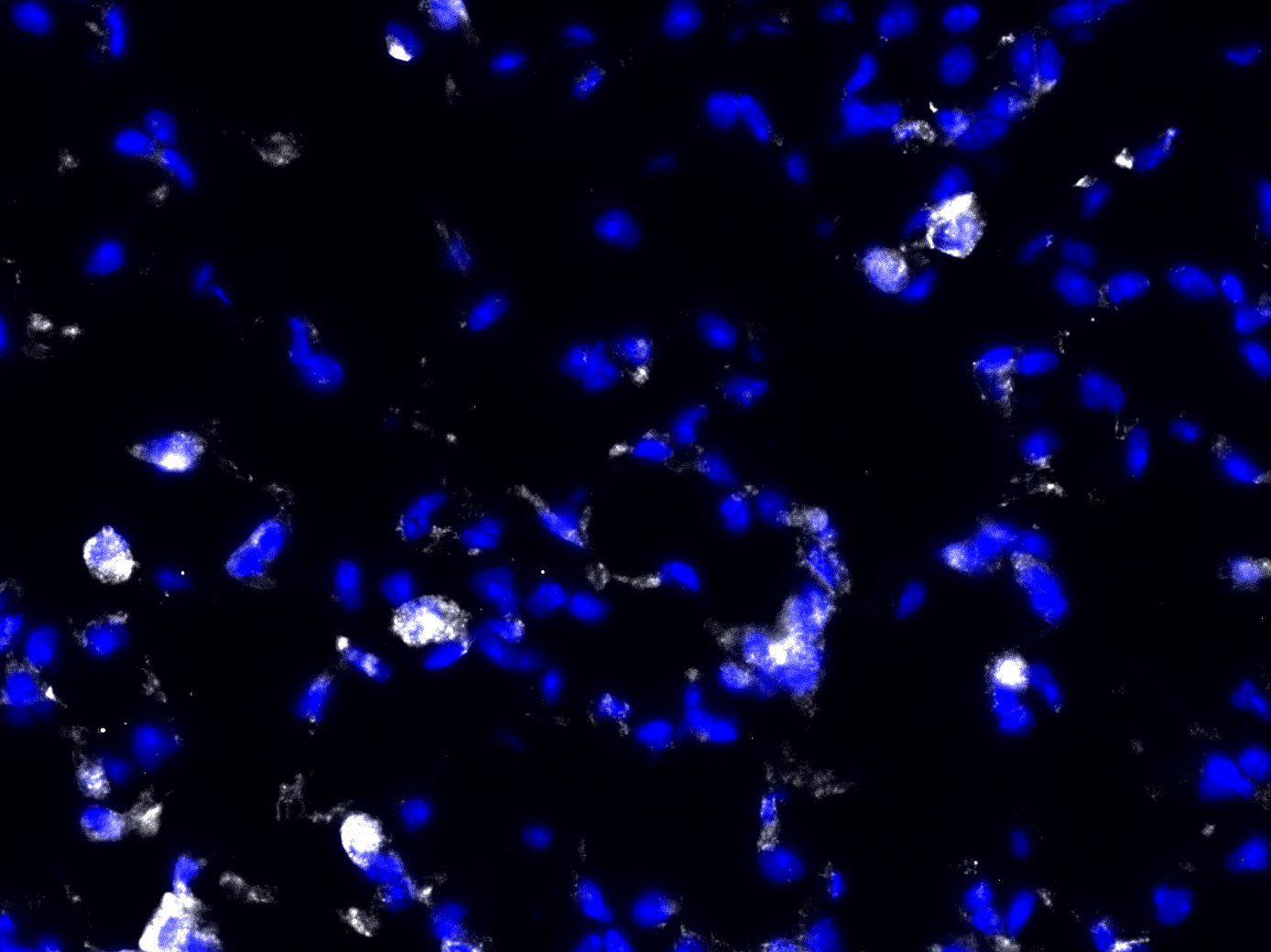

Supplement: Supplementary file 10 — Source Data Fig. 8 [file 44319_2023_41_MOESM10_ESM.zip › Source data Figure 8/8A Image data Micr images/aged/p21+TERT aged (p16 + DAPI).tif]

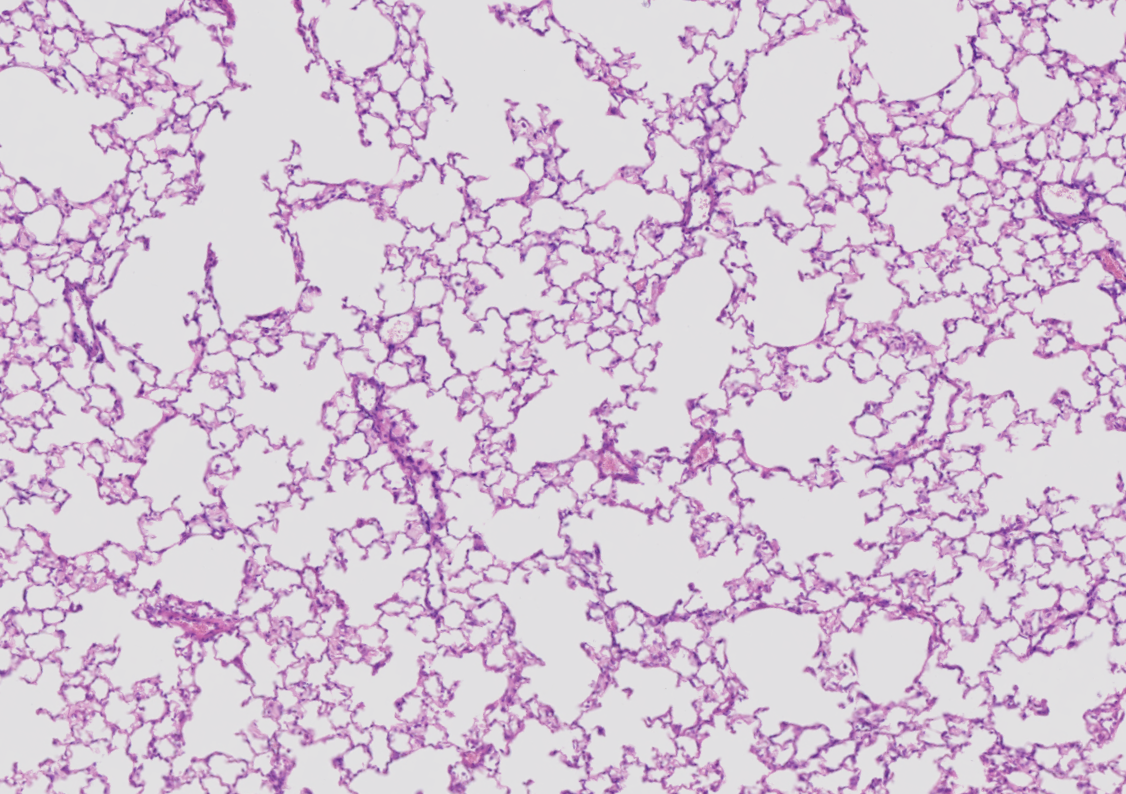

Supplement: Supplementary file 11 — Source Data Fig. 9 [file 44319_2023_41_MOESM11_ESM.zip › Source data Figure 9/9D Image data Micr image/p21 +TERT Hypoxia.tif]

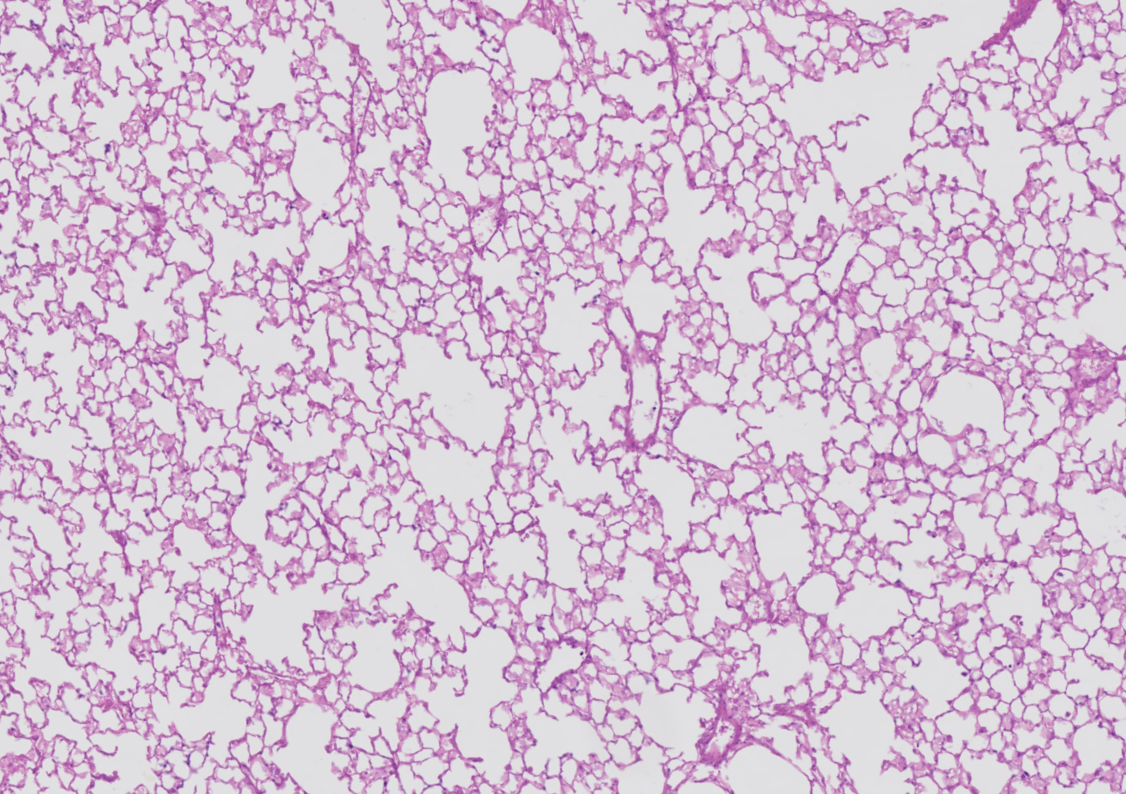

Supplement: Supplementary file 11 — Source Data Fig. 9 [file 44319_2023_41_MOESM11_ESM.zip › Source data Figure 9/9D Image data Micr image/p21+TERT CI Normoxia.tif]

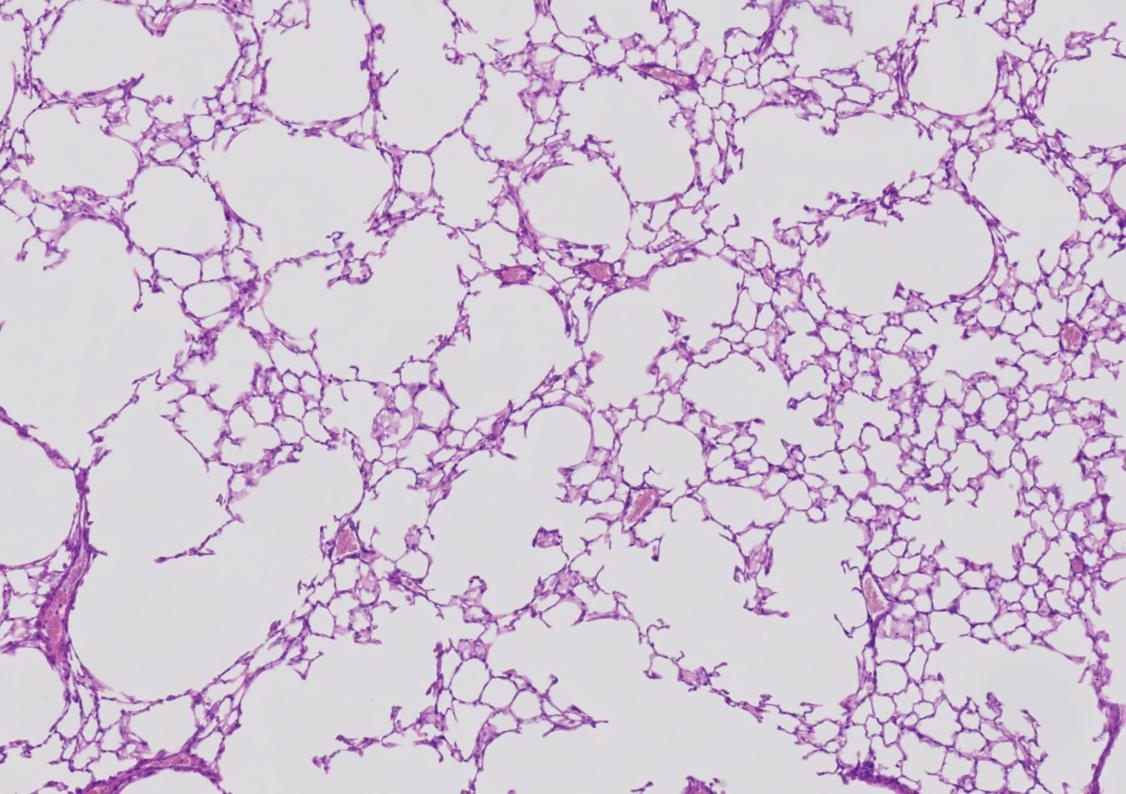

Supplement: Supplementary file 11 — Source Data Fig. 9 [file 44319_2023_41_MOESM11_ESM.zip › Source data Figure 9/9D Image data Micr image/p21+TERT CI Hypoxia + SUGEN.tif]

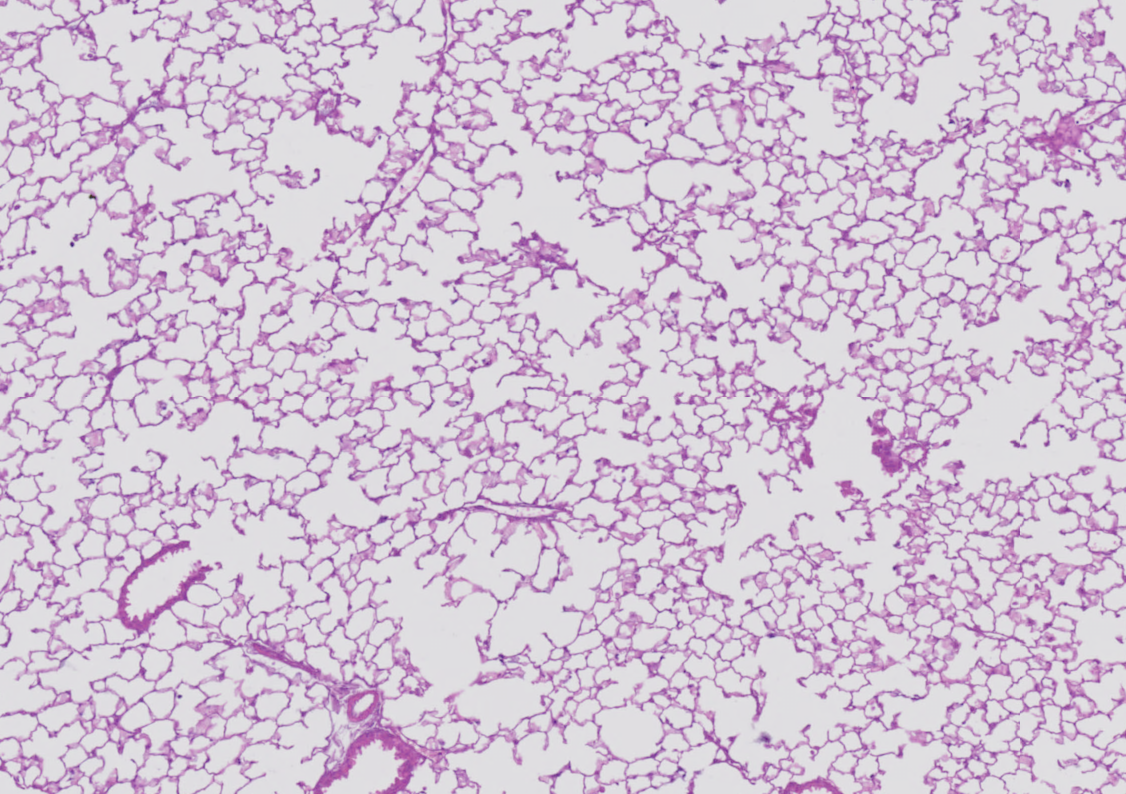

Supplement: Supplementary file 11 — Source Data Fig. 9 [file 44319_2023_41_MOESM11_ESM.zip › Source data Figure 9/9B Image data Micr image/p21+- Normoxia.tif]

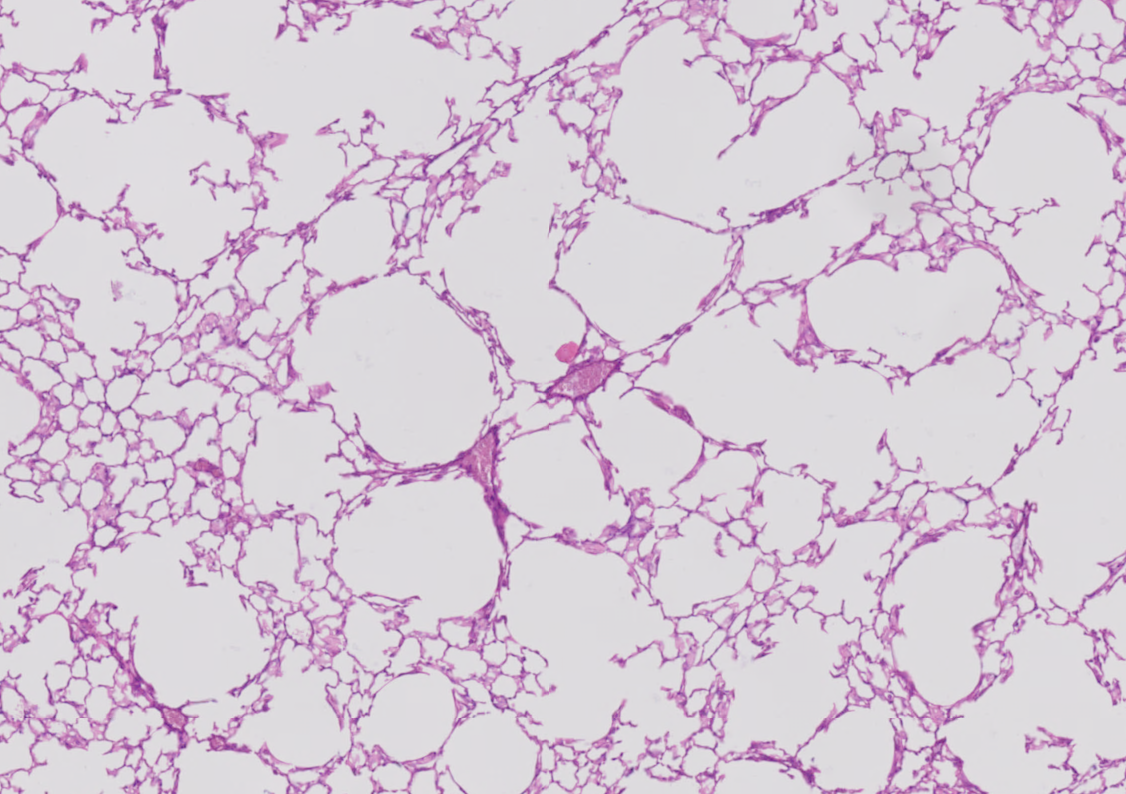

Supplement: Supplementary file 11 — Source Data Fig. 9 [file 44319_2023_41_MOESM11_ESM.zip › Source data Figure 9/9B Image data Micr image/p21+-Hypoxia + SUGEN.tif]

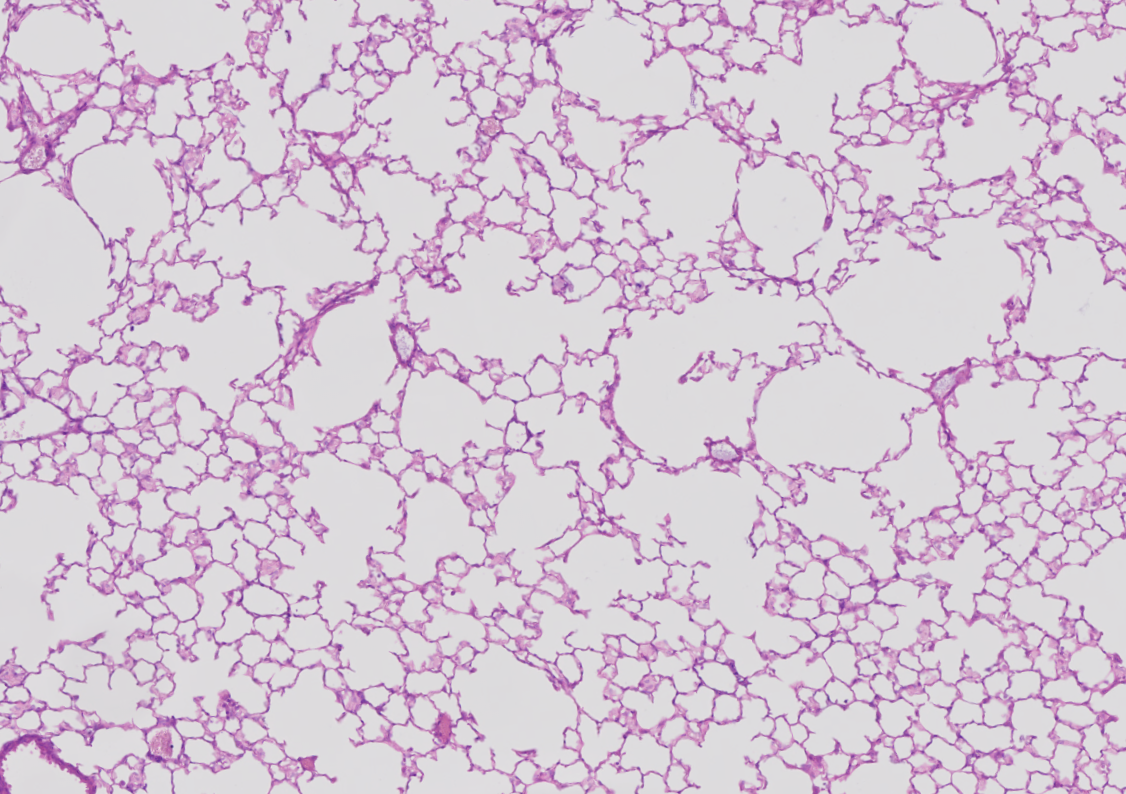

Supplement: Supplementary file 11 — Source Data Fig. 9 [file 44319_2023_41_MOESM11_ESM.zip › Source data Figure 9/9B Image data Micr image/p21+- Hypoxia.tif]

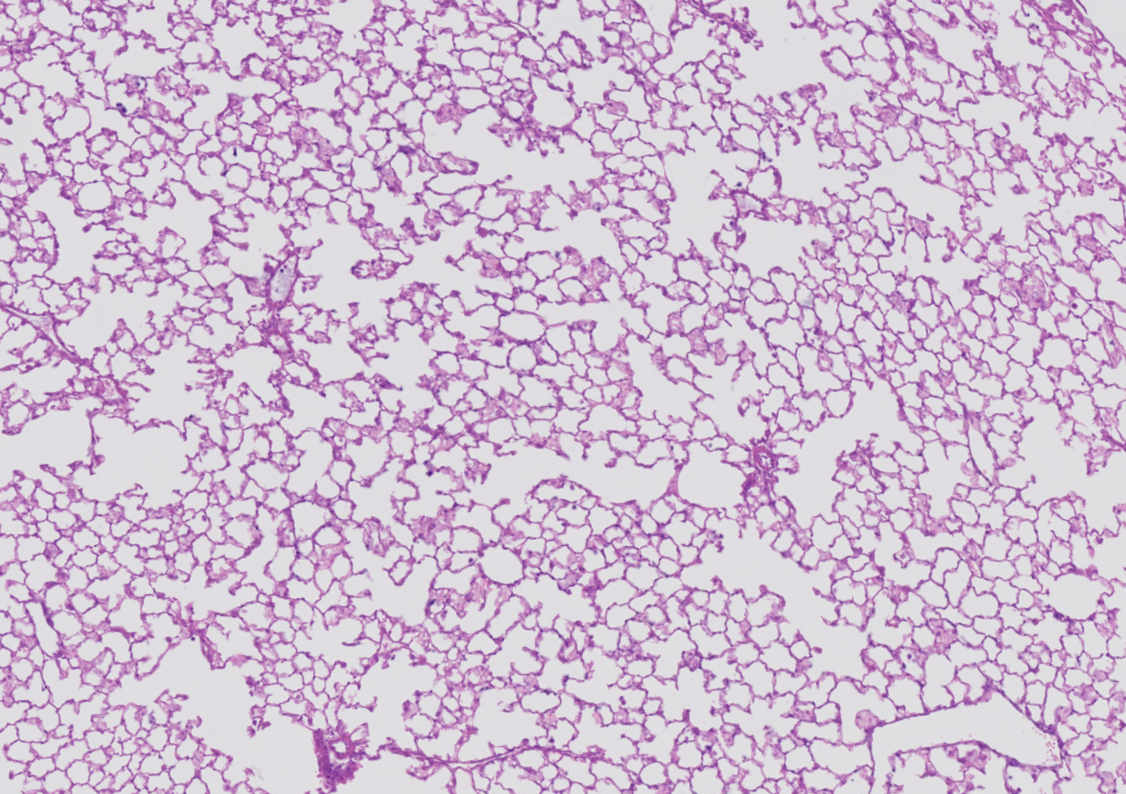

Supplement: Supplementary file 11 — Source Data Fig. 9 [file 44319_2023_41_MOESM11_ESM.zip › Source data Figure 9/9A Image data Micr image/p21++ Normoxia.tif]

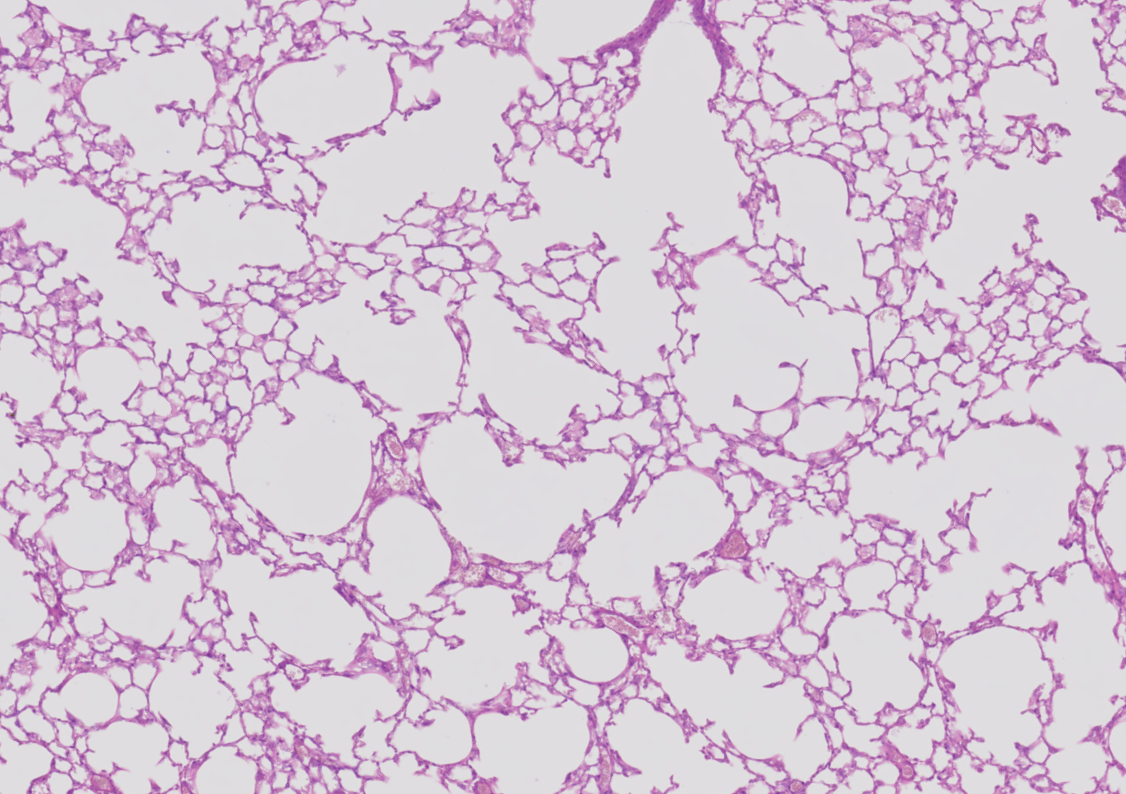

Supplement: Supplementary file 11 — Source Data Fig. 9 [file 44319_2023_41_MOESM11_ESM.zip › Source data Figure 9/9A Image data Micr image/p21++ Hypoxia + SUGEN.tif]

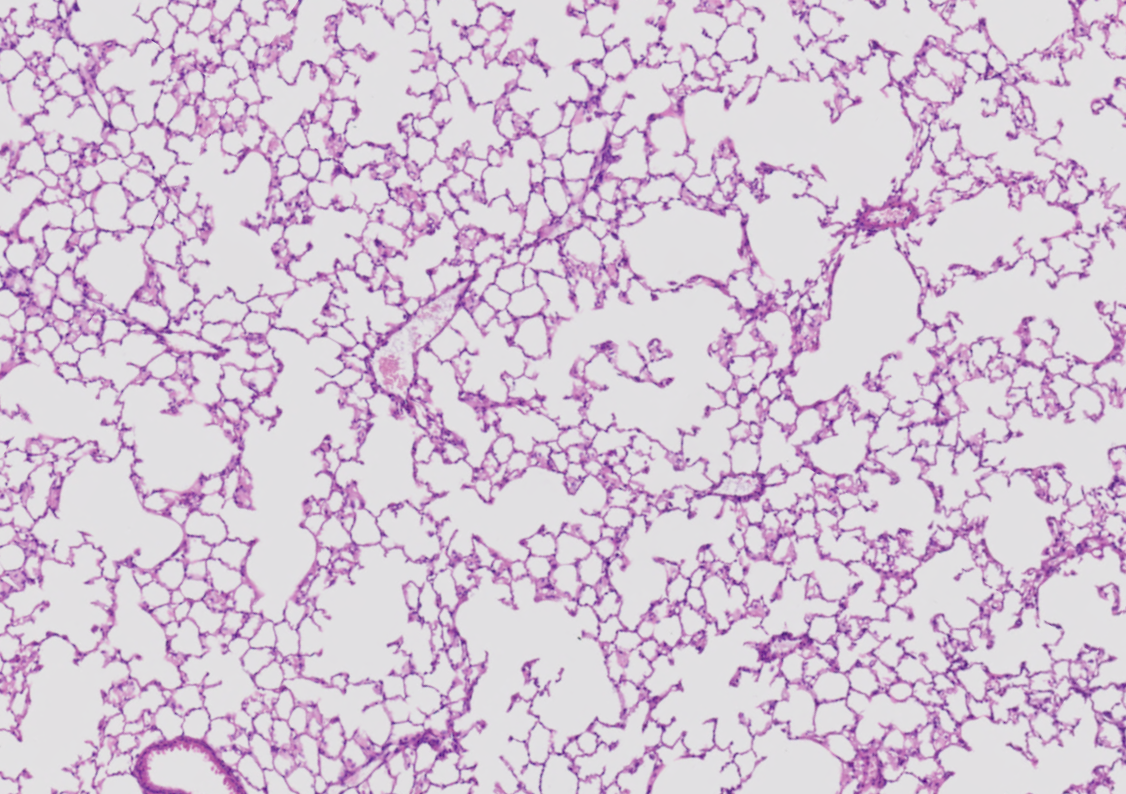

Supplement: Supplementary file 11 — Source Data Fig. 9 [file 44319_2023_41_MOESM11_ESM.zip › Source data Figure 9/9A Image data Micr image/p21++ Hypoxia.tif]

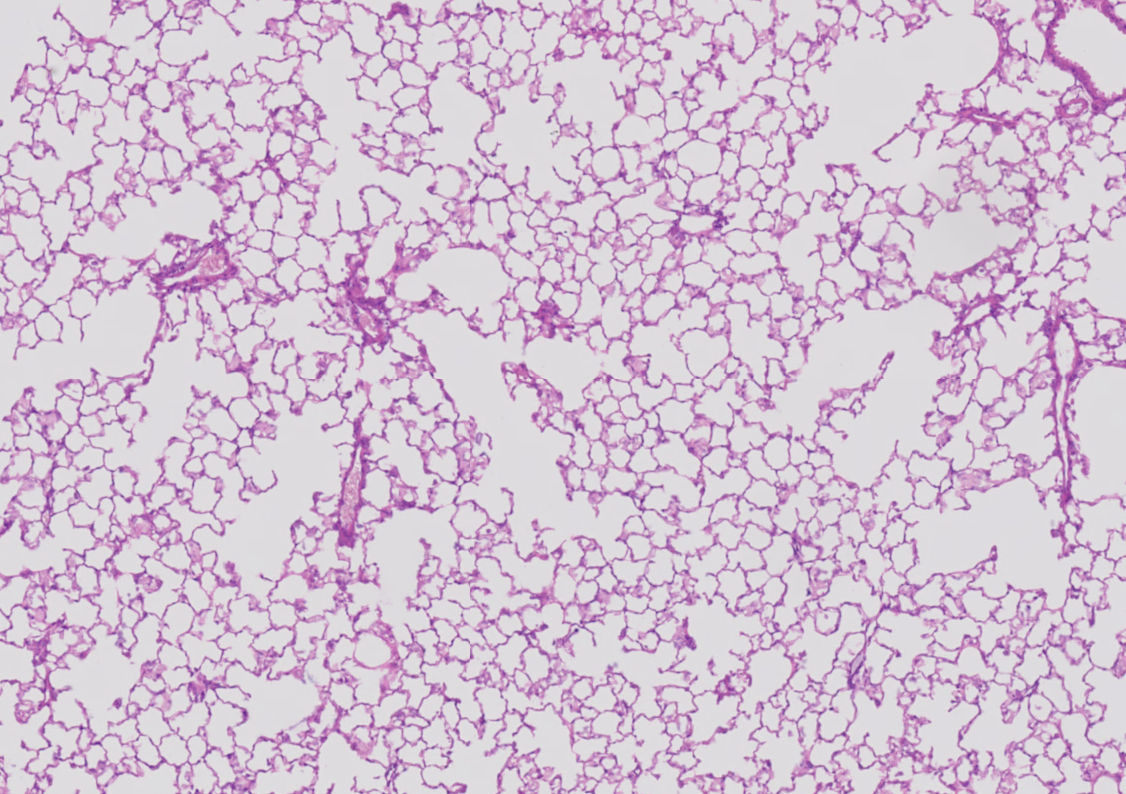

Supplement: Supplementary file 11 — Source Data Fig. 9 [file 44319_2023_41_MOESM11_ESM.zip › Source data Figure 9/9C Image data Micr image/p21+TERT Hypoxia + SUGEN.tif]

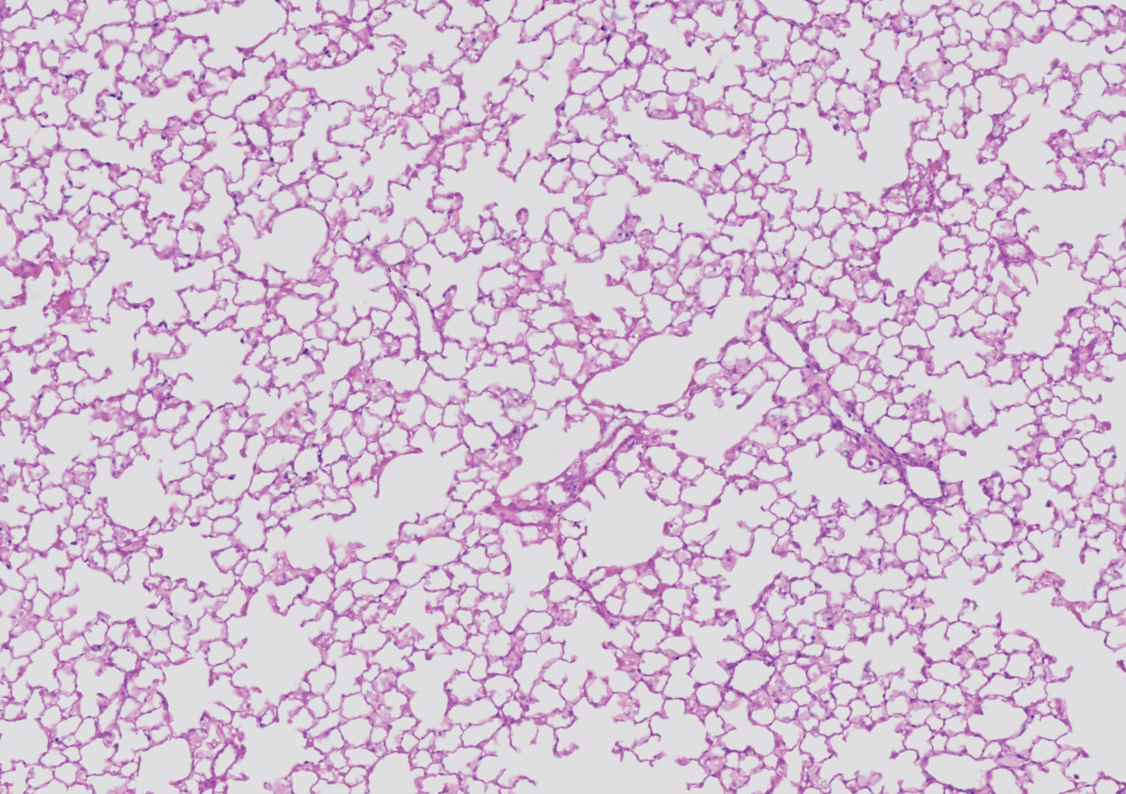

Supplement: Supplementary file 11 — Source Data Fig. 9 [file 44319_2023_41_MOESM11_ESM.zip › Source data Figure 9/9C Image data Micr image/p21+TERT Normoxia.tif]

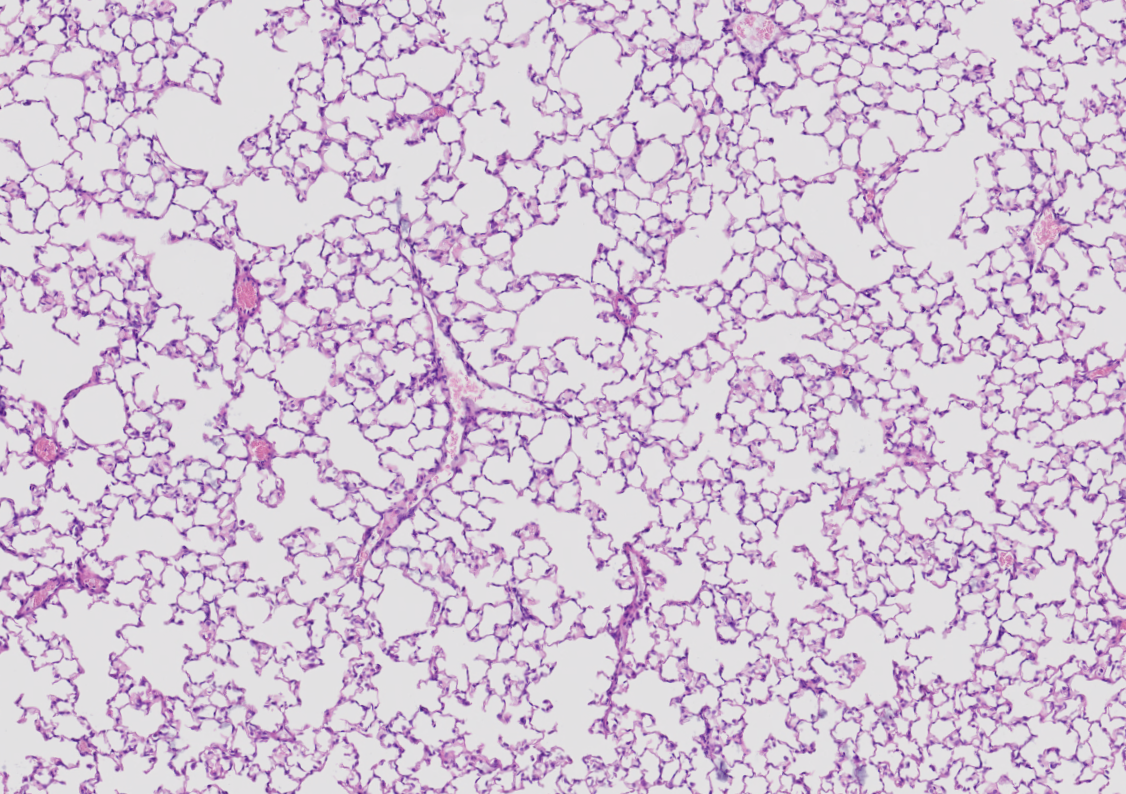

Supplement: Supplementary file 11 — Source Data Fig. 9 [file 44319_2023_41_MOESM11_ESM.zip › Source data Figure 9/9C Image data Micr image/p21+ TERT Hypoxia.tif]

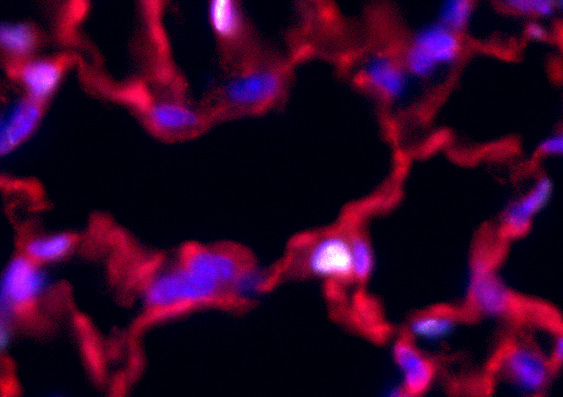

Supplement: Supplementary file 12 — Source Data Fig. 10 [file 44319_2023_41_MOESM12_ESM.zip › Source data Figure 10 /10 Upper panel Image data Micr Images/Normoxia/p21+TERT CI (p16+ DAPI + CD31) ZOOM.tif]

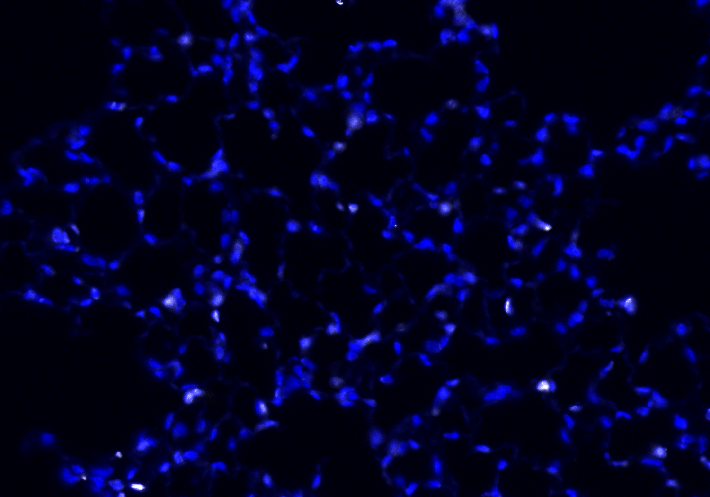

Supplement: Supplementary file 12 — Source Data Fig. 10 [file 44319_2023_41_MOESM12_ESM.zip › Source data Figure 10 /10 Upper panel Image data Micr Images/Normoxia/p21+TERT CI (p16+ DAPI).tif]

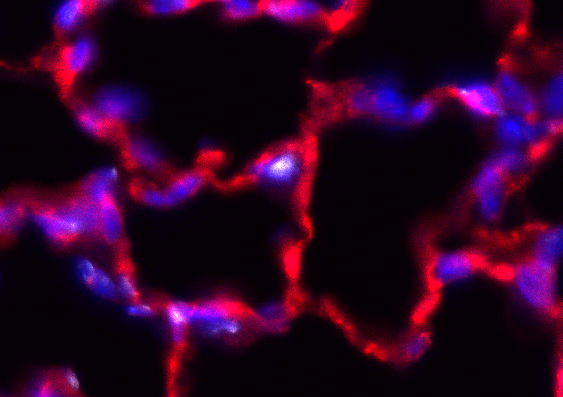

Supplement: Supplementary file 12 — Source Data Fig. 10 [file 44319_2023_41_MOESM12_ESM.zip › Source data Figure 10 /10 Upper panel Image data Micr Images/Normoxia/p21++ (p16+ DAPI + CD31) ZOOM.tif]

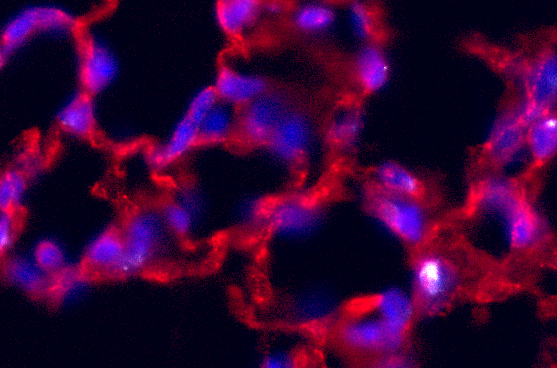

Supplement: Supplementary file 12 — Source Data Fig. 10 [file 44319_2023_41_MOESM12_ESM.zip › Source data Figure 10 /10 Upper panel Image data Micr Images/Normoxia/p21+TERT (p16+ DAPI + CD31) ZOOM.tif]

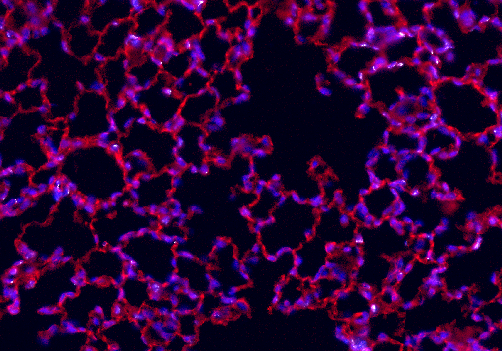

Supplement: Supplementary file 12 — Source Data Fig. 10 [file 44319_2023_41_MOESM12_ESM.zip › Source data Figure 10 /10 Upper panel Image data Micr Images/Normoxia/p21+TERT (p16+ DAPI + CD31).tif]

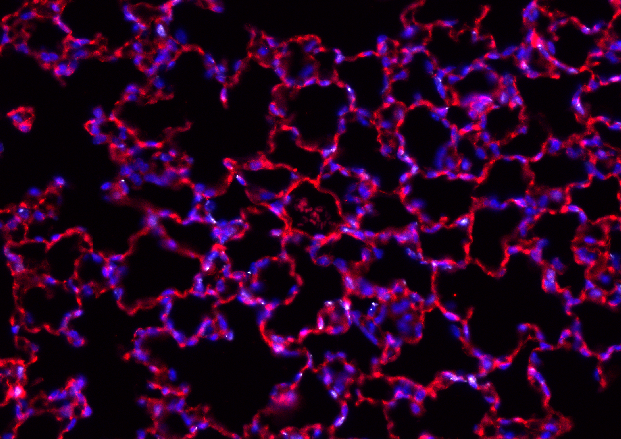

Supplement: Supplementary file 12 — Source Data Fig. 10 [file 44319_2023_41_MOESM12_ESM.zip › Source data Figure 10 /10 Upper panel Image data Micr Images/Normoxia/p21++ (p16+ DAPI+ CD31).tif]

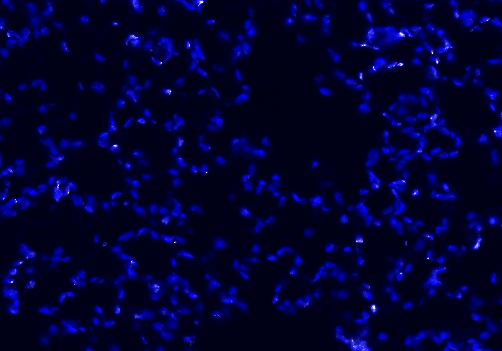

Supplement: Supplementary file 12 — Source Data Fig. 10 [file 44319_2023_41_MOESM12_ESM.zip › Source data Figure 10 /10 Upper panel Image data Micr Images/Normoxia/p21+- (p16+ DAPI).tif]

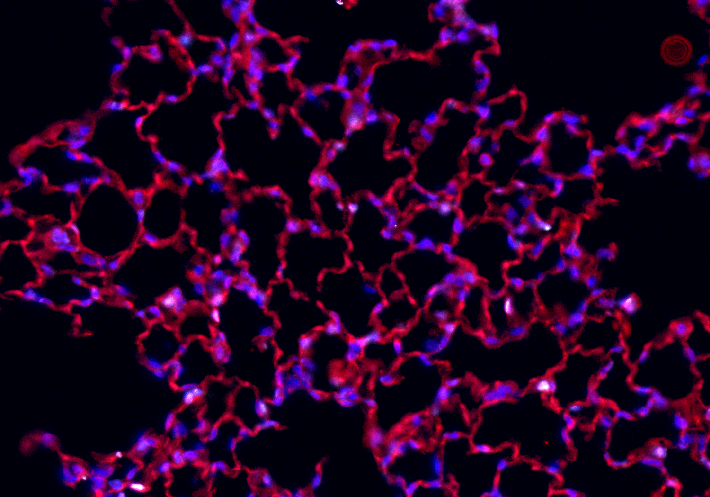

Supplement: Supplementary file 12 — Source Data Fig. 10 [file 44319_2023_41_MOESM12_ESM.zip › Source data Figure 10 /10 Upper panel Image data Micr Images/Normoxia/p21+TERT CI (p16+ DAPI + CD31).tif]
