# Supplementary figures and images for: mTert induction in p21-positive cells counteracts capillary rarefaction and pulmonary emphysema (part 3 of 3)
Source: EMBO Rep. 2024 Feb 29;25(3):36. doi: 10.1038/s44319-023-00041-1 (PMC10933469; doi:10.1038/s44319-023-00041-1)

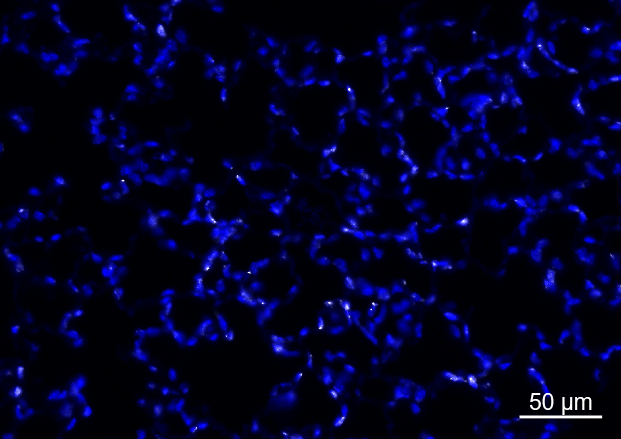

Supplement: Supplementary file 12 — Source Data Fig. 10 [file 44319_2023_41_MOESM12_ESM.zip › Source data Figure 10 /10 Upper panel Image data Micr Images/Normoxia/p21++ (p16+ DAPI).tif]

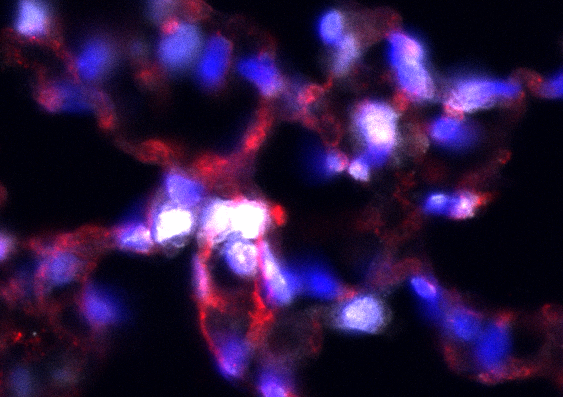

Supplement: Supplementary file 12 — Source Data Fig. 10 [file 44319_2023_41_MOESM12_ESM.zip › Source data Figure 10 /10 Upper panel Image data Micr Images/Hypoxia + SUGEN/p21+TERT CI (p16+ DAPI + CD31) ZOOM.tif]

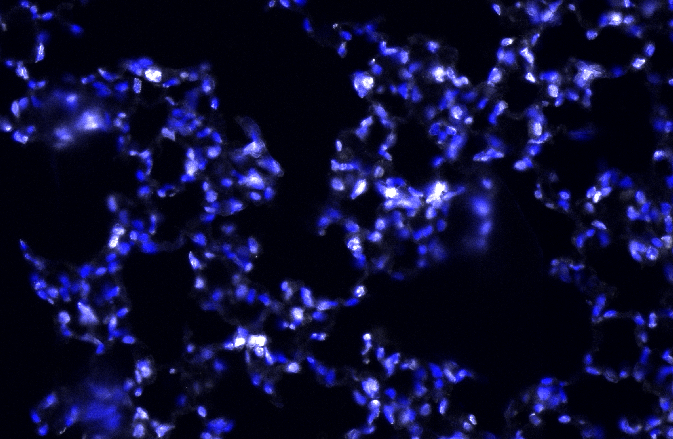

Supplement: Supplementary file 12 — Source Data Fig. 10 [file 44319_2023_41_MOESM12_ESM.zip › Source data Figure 10 /10 Upper panel Image data Micr Images/Hypoxia + SUGEN/p21+TERT CI (p16+ DAPI).tif]

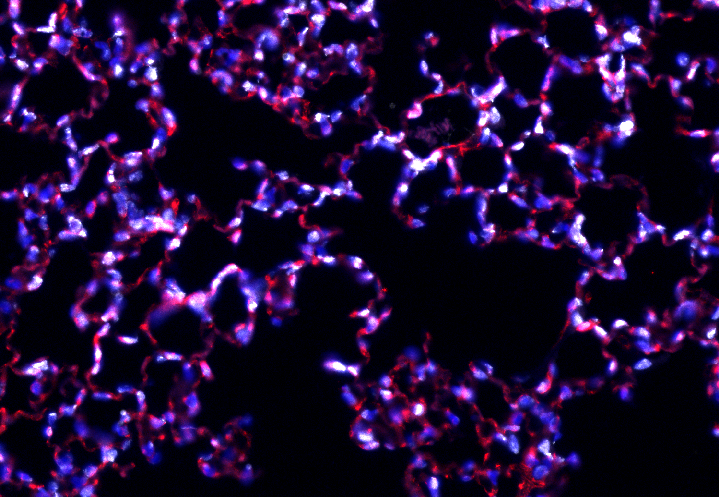

Supplement: Supplementary file 12 — Source Data Fig. 10 [file 44319_2023_41_MOESM12_ESM.zip › Source data Figure 10 /10 Upper panel Image data Micr Images/Hypoxia + SUGEN/p21++ (p16+ DAPI + CD31).tif]

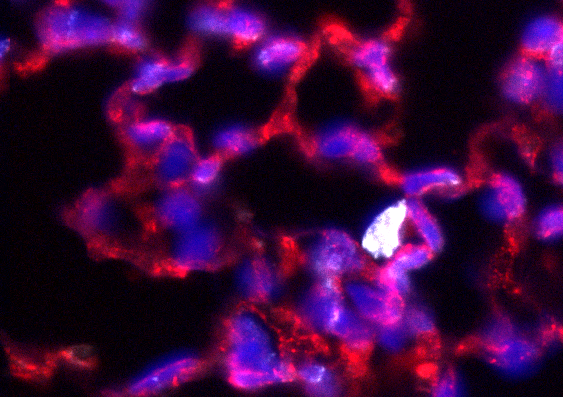

Supplement: Supplementary file 12 — Source Data Fig. 10 [file 44319_2023_41_MOESM12_ESM.zip › Source data Figure 10 /10 Upper panel Image data Micr Images/Hypoxia + SUGEN/p21+TERT (p16+ DAPI + CD31) ZOOM.tif]

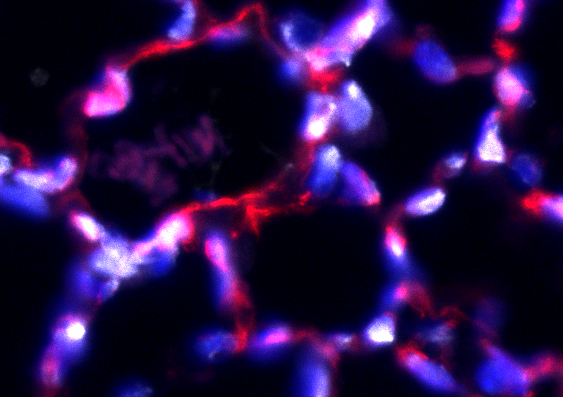

Supplement: Supplementary file 12 — Source Data Fig. 10 [file 44319_2023_41_MOESM12_ESM.zip › Source data Figure 10 /10 Upper panel Image data Micr Images/Hypoxia + SUGEN/p21++(p16+ DAPI + CD31) ZOOM.tif]

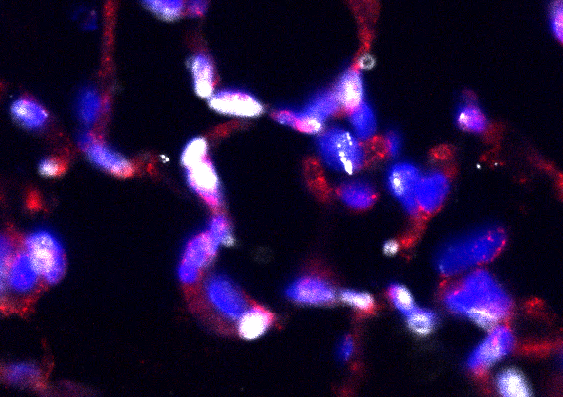

Supplement: Supplementary file 12 — Source Data Fig. 10 [file 44319_2023_41_MOESM12_ESM.zip › Source data Figure 10 /10 Upper panel Image data Micr Images/Hypoxia + SUGEN/p21+- (p16+ DAPI+ CD31) ZOOM.tif]

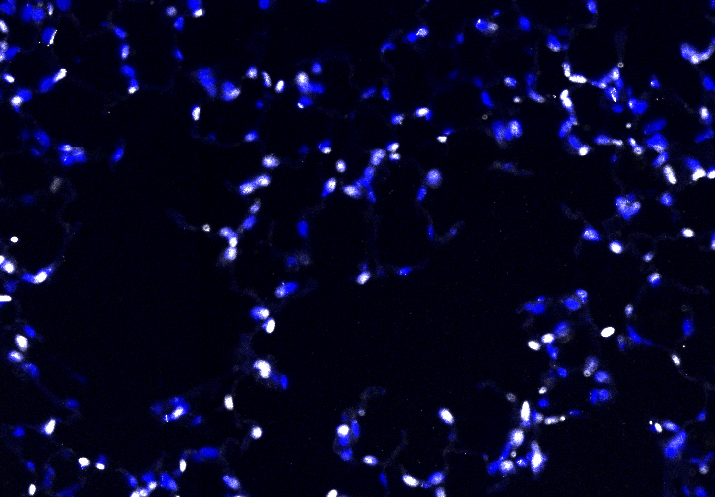

Supplement: Supplementary file 12 — Source Data Fig. 10 [file 44319_2023_41_MOESM12_ESM.zip › Source data Figure 10 /10 Upper panel Image data Micr Images/Hypoxia + SUGEN/p21+- (p16+ DAPI).tif]

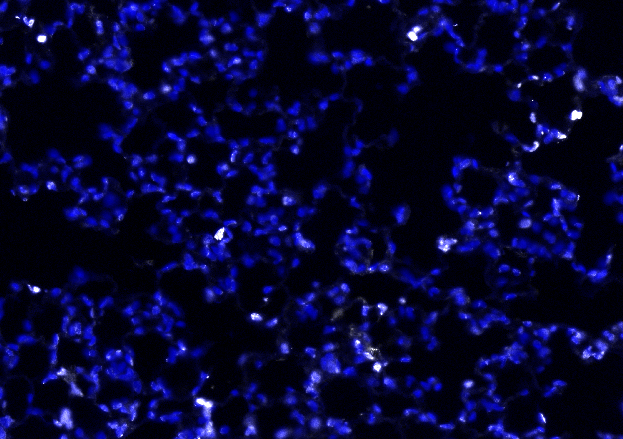

Supplement: Supplementary file 12 — Source Data Fig. 10 [file 44319_2023_41_MOESM12_ESM.zip › Source data Figure 10 /10 Upper panel Image data Micr Images/Hypoxia + SUGEN/p21+TERT (p16+ DAPI).tif]

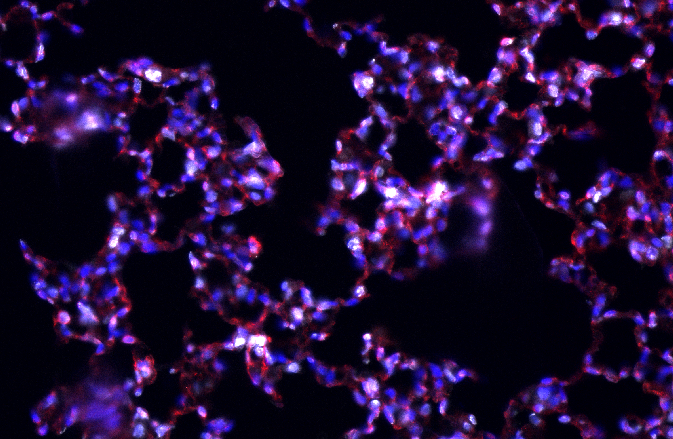

Supplement: Supplementary file 12 — Source Data Fig. 10 [file 44319_2023_41_MOESM12_ESM.zip › Source data Figure 10 /10 Upper panel Image data Micr Images/Hypoxia + SUGEN/p21+TERT CI (p16+ DAPI + CD31).tif]

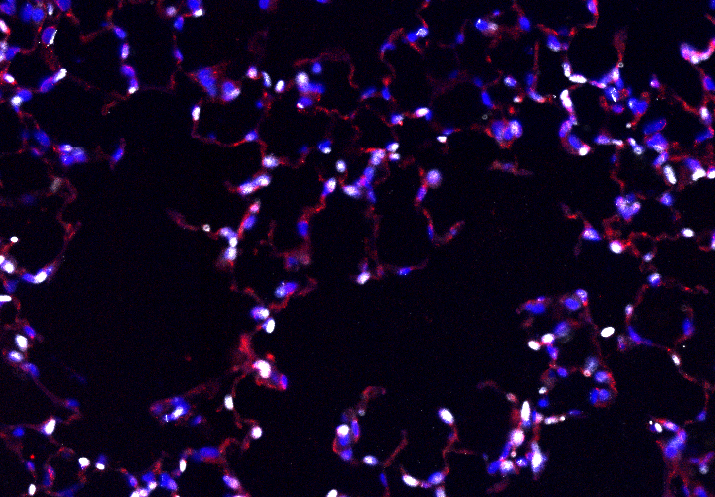

Supplement: Supplementary file 12 — Source Data Fig. 10 [file 44319_2023_41_MOESM12_ESM.zip › Source data Figure 10 /10 Upper panel Image data Micr Images/Hypoxia + SUGEN/p21+- (p16+ DAPI +CD31).tif]

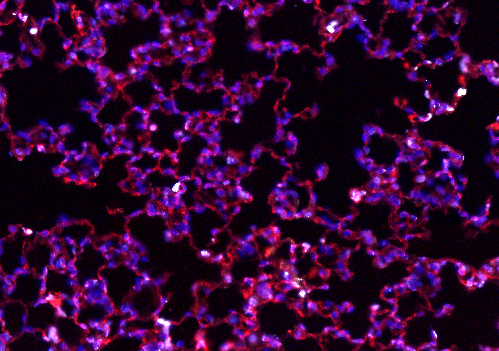

Supplement: Supplementary file 12 — Source Data Fig. 10 [file 44319_2023_41_MOESM12_ESM.zip › Source data Figure 10 /10 Upper panel Image data Micr Images/Hypoxia + SUGEN/p21+TERT (p16+ DAPI + CD31).tif]

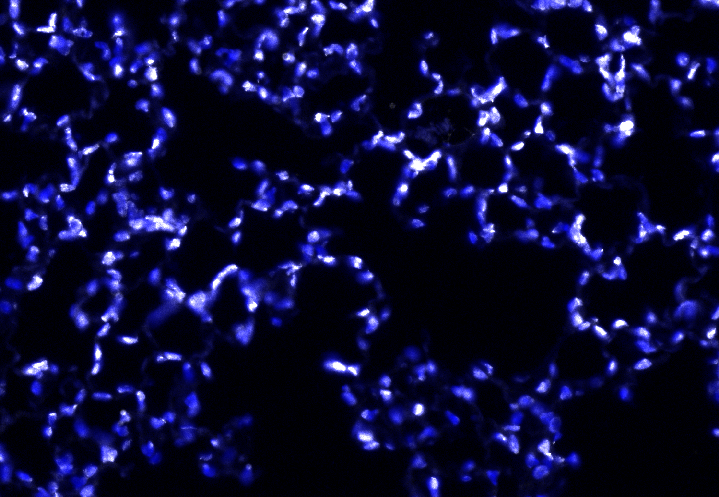

Supplement: Supplementary file 12 — Source Data Fig. 10 [file 44319_2023_41_MOESM12_ESM.zip › Source data Figure 10 /10 Upper panel Image data Micr Images/Hypoxia + SUGEN/p21++ (p16+ DAPI).tif]
