# Supplementary material for: mTert induction in p21-positive cells counteracts capillary rarefaction and pulmonary emphysema
Source: EMBO Rep. 2024 Feb 29;25(3):36. doi: 10.1038/s44319-023-00041-1 (PMC10933469; doi:10.1038/s44319-023-00041-1)
Supplement: Supplementary file 15 — Expanded View Figures [file 44319_2023_41_MOESM15_ESM.pdf]

Expanded View Figures

Figure EV1. Validation of p21$^{+/Tert}$ model.

(**A**) p21$^{+/+}$ and p21$^{+/Tert}$ littermates were subjected to whole-body ionizing radiation (1.5 gray). The fluorescence emitted by the mCherry was followed post-irradiation at the indicated times by in vivo mCherry imaging (Excitation = 545 nm, Background = 495 nm, Emission = 615 nm). (**B**) Left panel, p21 expression and mCherry fluorescence were analyzed in the liver and kidneys after doxorubicin treatments. Right panel, livers and kidneys of p21$^{+/+}$ and p21$^{+/Tert}$ mice were harvested 24 h after doxorubicin treatment. The level of p21 protein was evaluated by semi-quantitative immunoblotting and liver and kidney were imaged for mCherry fluorescence. (**C**) Primers to distinguish transgene expression from total Tert expression are shown. (**D**) *Tert* RT-qPCR experiments were performed using RNA extracted from the harvested organs. The mean and the SEM of the three biological PCR replicates are plotted. For both organs, the difference between the doxorubicin-treated and untreated samples is statistically significant ($p < 0.001$, unpaired Student's t test) for the p21$^{+/Tert}$ but not p21$^{+/+}$ mice.

© The Author(s)

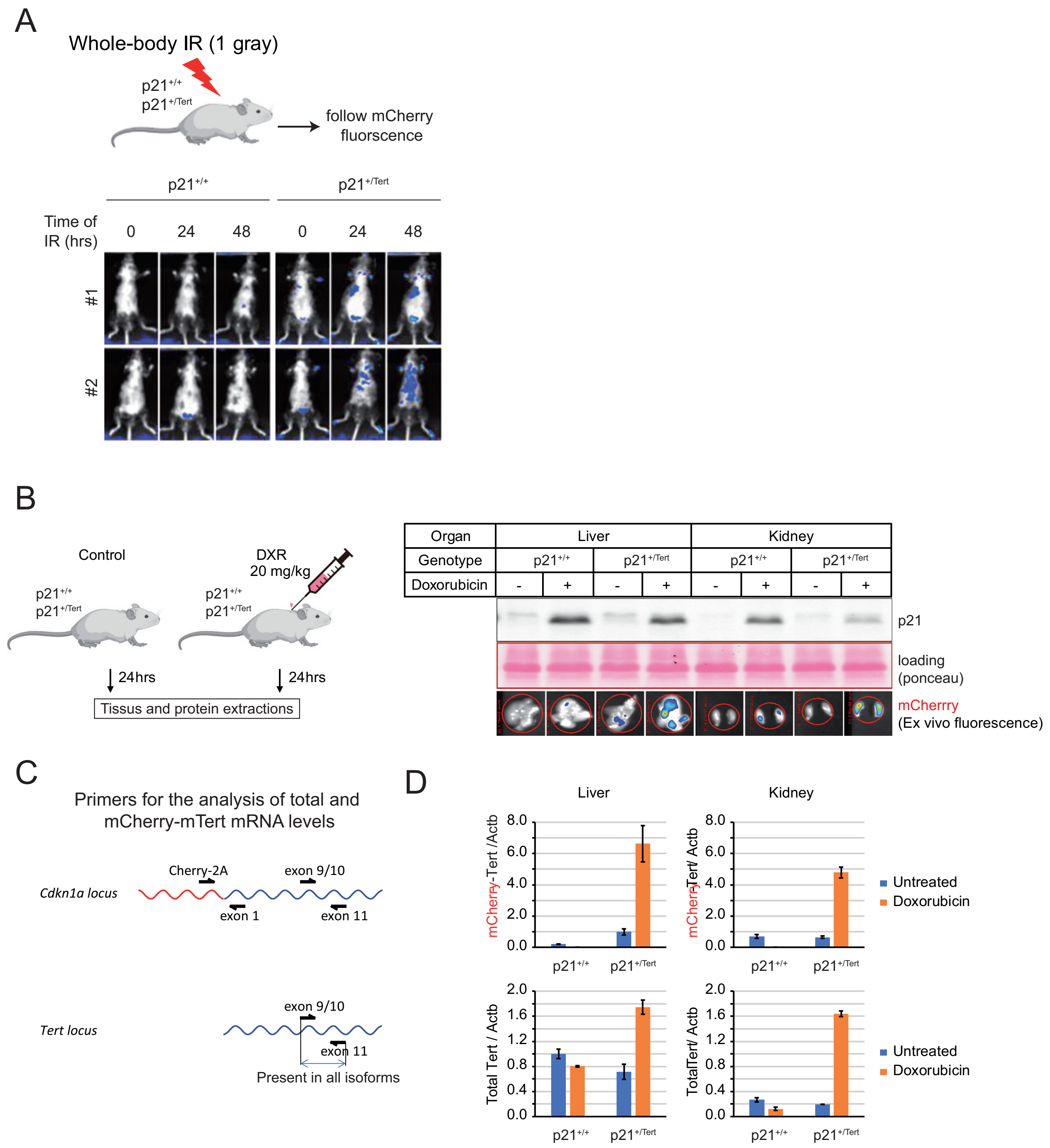
A
Whole-body IR (1 gray)
p21+/+
p21+/Tert
follow mCherry fluorscence
p21+/+
p21+/Tert
Time of IR (hrs)
0
24
48
0
24
48
#1
#2
B
Control
DXR 20 mg/kg
p21+/+
p21+/Tert
p21+/+
p21+/Tert
24hrs
24hrs
Tissus and protein extractions
Organ
Liver
Kidney
Genotype
p21+/+
p21+/Tert
p21+/+
p21+/Tert
Doxorubicin
-
+
-
+
-
+
-
+
p21
loading (ponceau)
mCherrry (Ex vivo fluorescence)
C
Primers for the analysis of total and mCherry-mTert mRNA levels
Cdkn1a locus
Cherry-2A
exon 9/10
exon 1
exon 11
Tert locus
exon 9/10
exon 11
Present in all isoforms
D
Liver
Kidney
mCherry-Tert /Actb
mCherry-Tert/ Actb
8.0
6.0
4.0
2.0
0.0
p21+/+
p21+/Tert
Untreated
Doxorubicin
Total Tert / Actb
TotalTert/ Actb
2.0
1.6
1.2
0.8
0.4
0.0
p21+/+
p21+/Tert
Untreated
Doxorubicin

 © The Author(s)

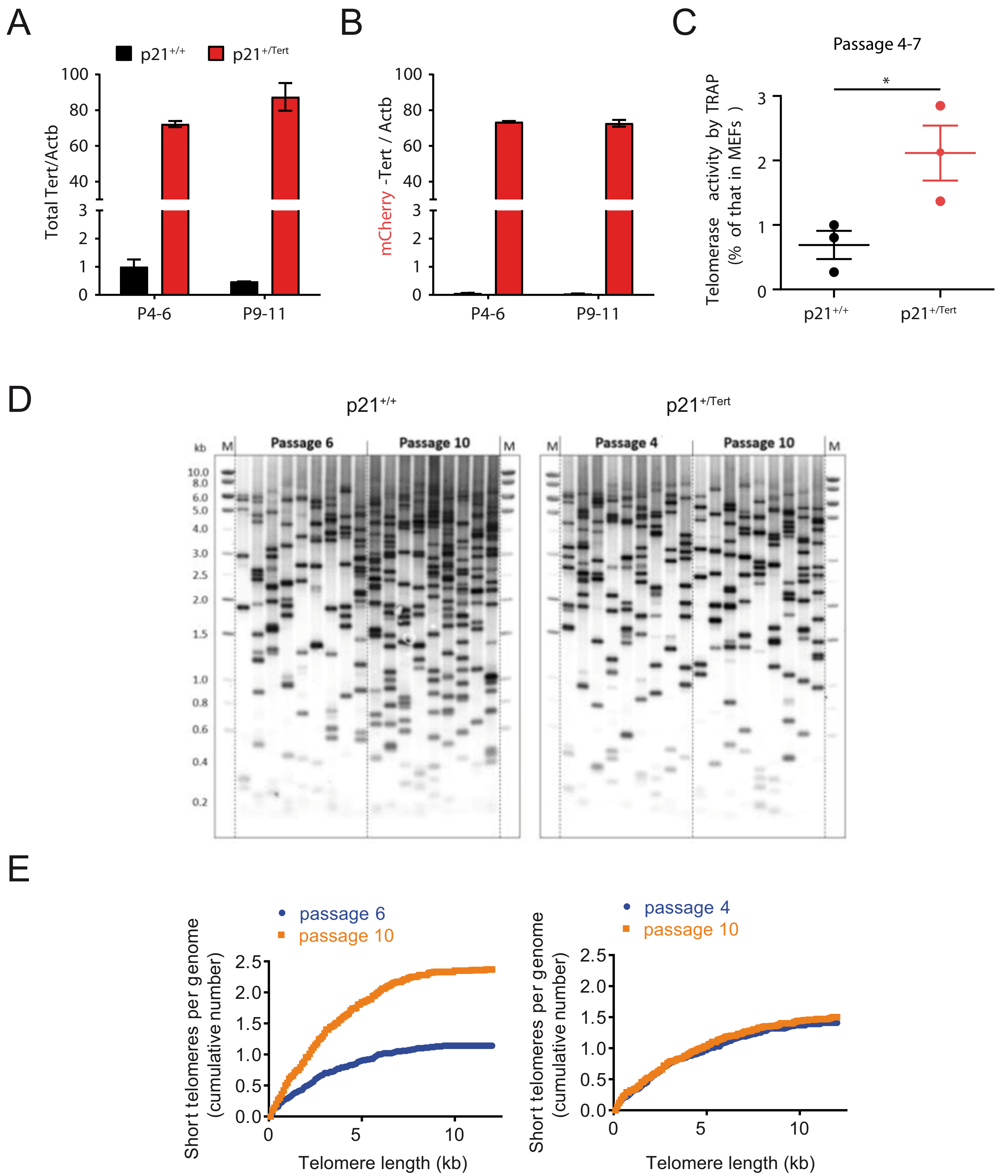
A
p21+/+
p21+/Tert
Total Tert/Actb
100
80
60
40
3
2
1
0
P4-6
P9-11
B
mCherry -Tert / Actb
P4-6
P9-11
C
Passage 4-7
*
Telomerase activity by TRAP (% of that in MEFs)
p21+/+
p21+/Tert
D
p21+/+
p21+/Tert
kb
M
Passage 6
Passage 10
Passage 4
Passage 10
10.0
8.0
6.0
5.0
4.0
3.0
2.5
2.0
1.5
1.0
0.8
0.6
0.4
0.2
E
passage 6
passage 10
passage 4
passage 10
Short telomeres per genome (cumulative number)
Telomere length (kb)
2.5
2.0
1.5
1.0
0.5
0.0
0
5
10

Figure EV2. p21-promoter dependent mTert expression bypasses senescence in PA-SMCs ex vivo (related to Fig. 1C).

(**A**, **B**) Quantification of the m*Tert* mRNA levels in PA-SMCs from the $p21^{+/+}$ and $p21^{+/Tert}$ 4-month-old mice (littermates) at early (p4-6) and late (p9-11) passage. Left panel represents the level of total *Tert* mRNA (transcribed from both the native Tert locus and KI allele), while right panel represents the level of *Tert* mRNA transcribed from KI allele only. Nearly all *Tert* mRNA is transcribed from the KI allele. The means of three independent measurements are plotted, and the error bars are SEs. The difference in the level of *Tert* mRNA between the $p21^{+/+}$ and $p21^{+/Tert}$ cells is highly significant ($p < 0.001$, unpaired Student's t test) for both early and late passages. (**C**) Telomerase activity measured by qTRAP at early passages. The data points correspond to vascular PA-SMC cultures established from individual $p21^{+/+}$ and $p21^{+/Tert}$ 4-month-old mice. The data are expressed as the mean ± SEM. $^{*}p < 0.05$ from the two-sided t test. (**D**, **E**) Analysis of the short telomere fraction by Telomere Shortest Length Assay (TeSLA) in the cultured PA-SMCs from $p21^{+/+}$ and $p21^{+/Tert}$ mice. Southern blots probed for the TTAGGG repeats in (**A**) and quantification of the cumulative number of short telomeres across the telomere length thresholds in (**E**). Source data are available online for this figure.

 © The Author(s)

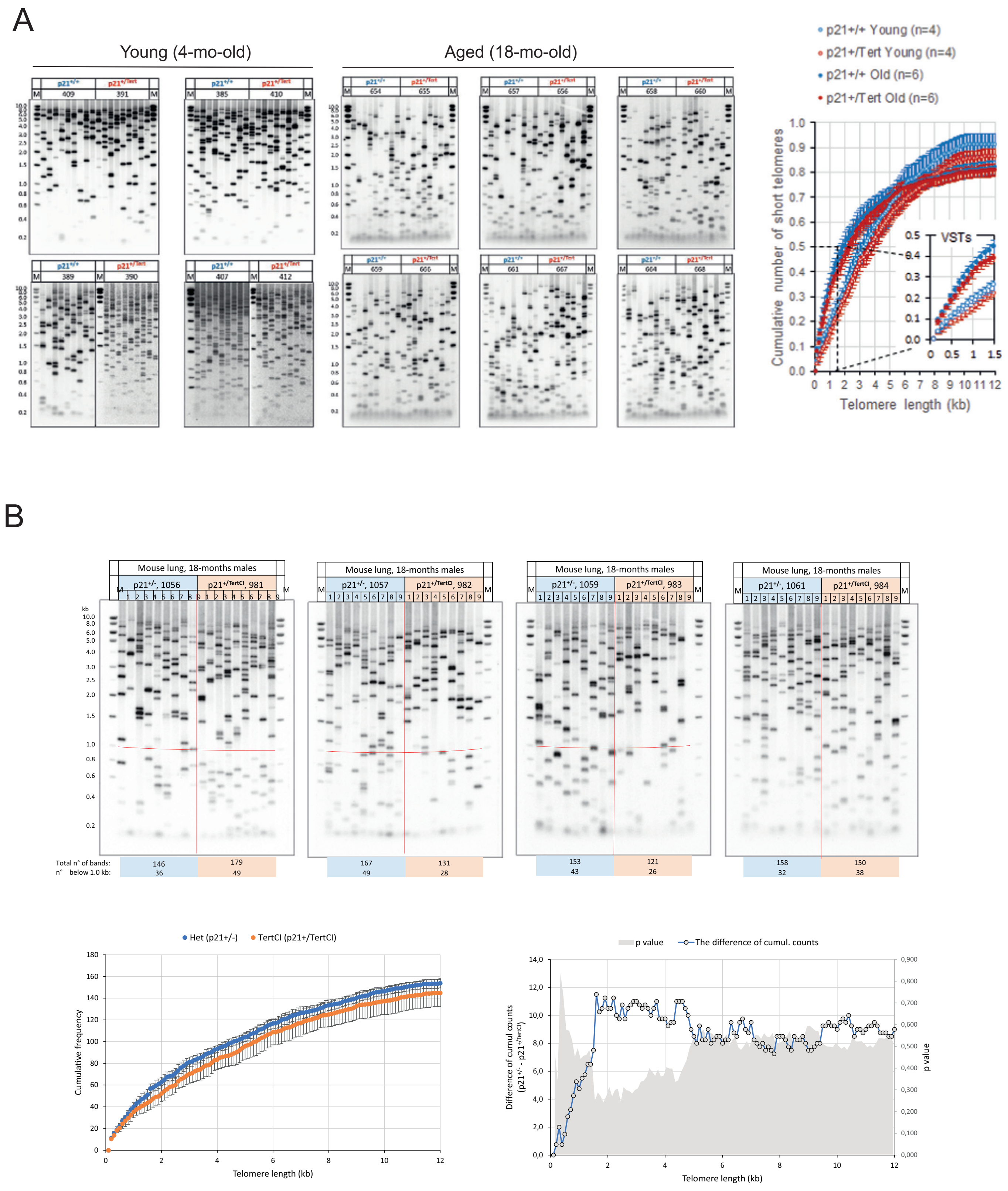
A
Young (4-mo-old)
Aged (18-mo-old)
p21+/+ Young (n=4)
p21+/Tert Young (n=4)
p21+/+ Old (n=6)
p21+/Tert Old (n=6)
Cumulative number of short telomeres
Telomere length (kb)
VSTs
B
Mouse lung, 18-months males
p21+/-, 1056
p21+/TertCI, 981
p21+/-, 1057
p21+/TertCI, 982
p21+/-, 1059
p21+/TertCI, 983
p21+/-, 1061
p21+/TertCI, 984
Total n° of bands:
n° below 1.0 kb:
146 36
179 49
167 49
131 28
153 43
121 26
158 32
150 38
Het (p21+/-)
TertCI (p21+/TertCI)
Cumulative frequency
Telomere length (kb)
p value
The difference of cumul. counts
Difference of cumul counts (p21+/- - p21+/TertCI)
p value
Telomere length (kb)

◀ **Figure EV3. Analysis of the individual short telomeres by TeSLA in the lungs of the young and old mice.**

(**A**) TeSLA Southern blots depicting short telomeres in the lungs of the $p21^{+/+}$ and $p21^{+/Tert}$ littermates (4 and 18-month-old mice). Genomic DNA was extracted from whole lungs and the length of individual short telomeres was determined by TeSLA. Note that young mice, regardless of their genotype, have less telomeres shorter than 1 kb compared to the old mice. The graph depicts cumulative number of short telomeres per genome in the range of 0.2–12 kb. The area corresponding to the very short telomeres (VSTs) is magnified in the inset. The mean values ± SEM are plotted. (**B**) TeSLA Southern blots depicting short telomeres in the lungs of the $p21^{+/-}$ and $p21^{+/TertCI}$ mice (18-month-old). TeSLA Southern blots are shown on top. The graph on the bottom left shows the cumulative frequency of short telomeres in the range of 0.2–12 kb for the mice of two genotypes. The graph on the bottom right depicts the difference of cumulative counts for 0.1 kb bins between the two genotypes (left *y* axis) and the corresponding *p* values from the two-tailed *t* test (right *y* axis). The difference is not significant for any bin indicating that $Tert^{CI}$ is unable to improve the load of the short telomeres in old mice.

 © The Author(s)

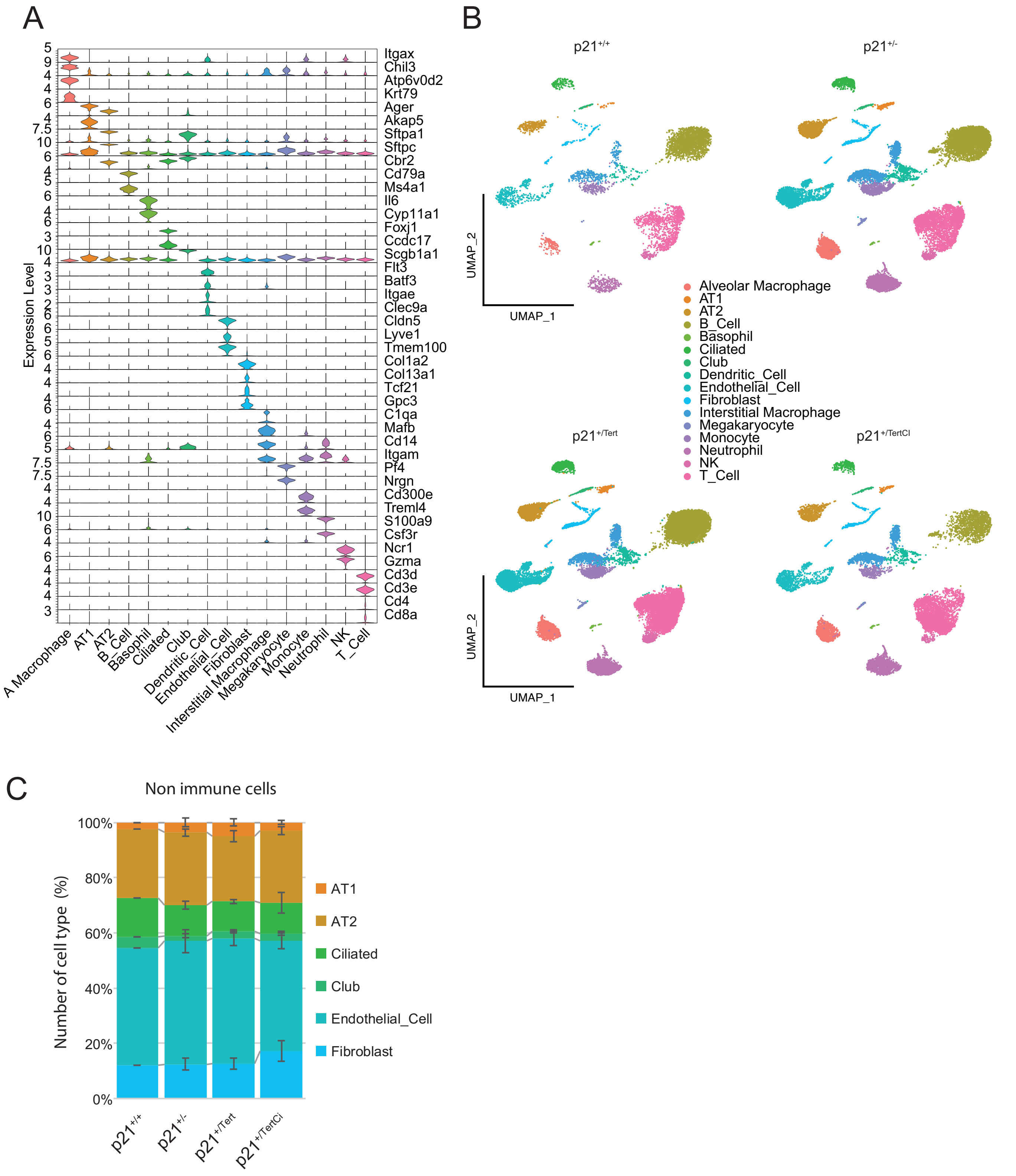

Figure EV4. Lung cell types in the four mouse models.

(**A**) Representative markers use to annotate lung cell types in the four mouse models. (**B**) UMAP clustering of lung cells. Lung cell populations were identified in lung samples from WT ($p21^{+/+}$), $p21^{+/-}$, $p21^{+/Tert}$ and $p21^{+/TertCl}$ 18-month old mice. (**C**) Quantification of the cell types in lungs of the mice of the 4 indicated genotypes. At least 3 mice of each genotype were analyzed. The mean values ± SEM are plotted.

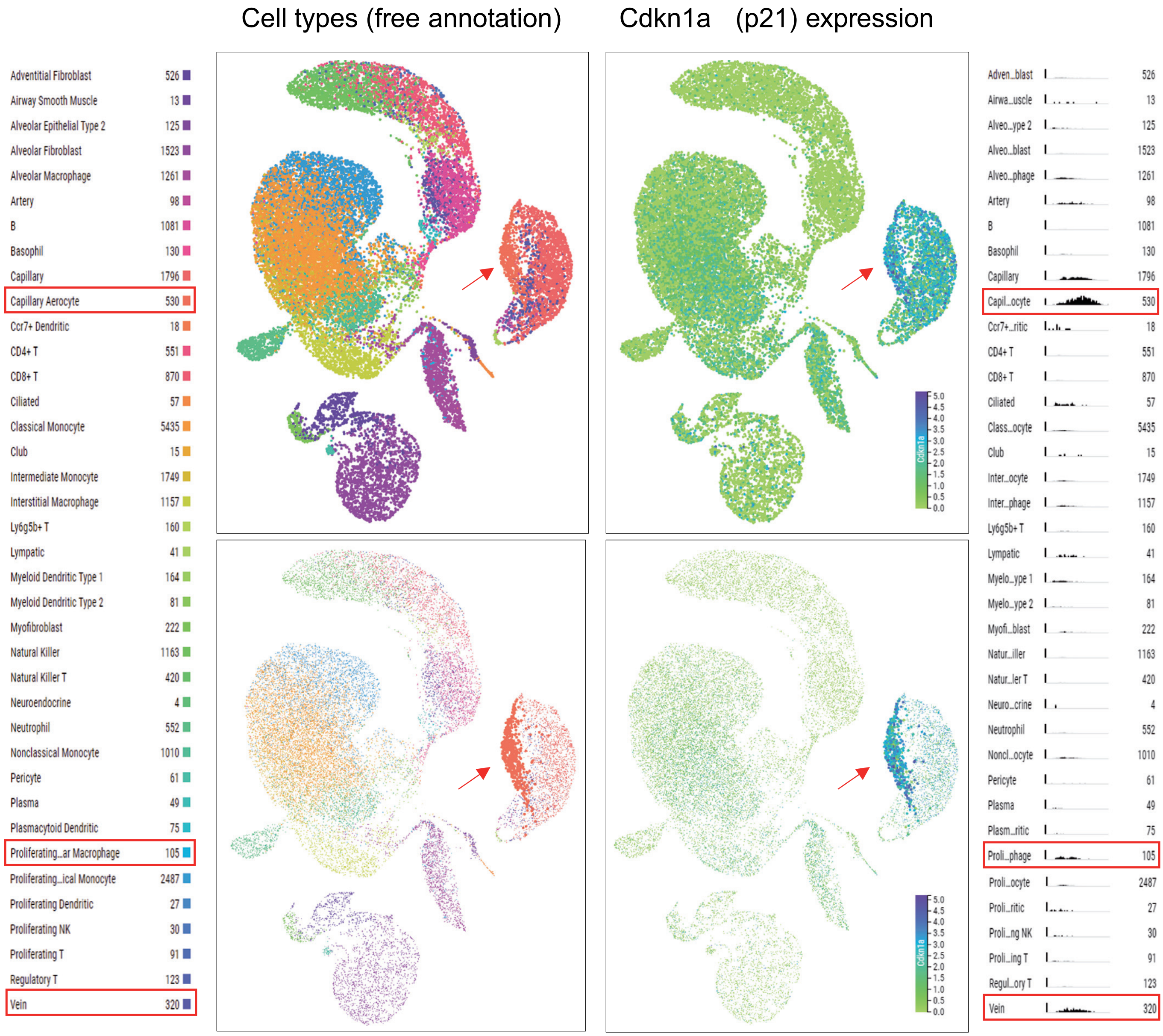

Figure EV5. UMAP plots generated using lung droplet scRNA-seq data in Tabula Muris Senis.

The data for mice of all ages (1–30 months) were included. In the top panels all cell types are shown, while in the bottom panels only the capillary aerocyte population is highlighted. Red arrow points to the capillary aerocytes, and red boxes in the annotation mark cell types with elevated p21 expression level. Histograms (on the right) show scaled number of cells expressing p21 (*y* axis) versus expression level (*x* axis).

 © The Author(s)
